# HMPA-Enabled Direct γ′-Arylation of Cyclic Vinylogous Esters

**DOI:** 10.1021/acs.orglett.6c00380

**Published:** 2026-02-05

**Authors:** Yan-Xun Li, Wei-Ting Zhao, Yen-Ku Wu

**Affiliations:** † Department of Applied Chemistry and Center for Emergent Functional Matter Science, 34914 National Yang Ming Chiao Tung University, 1001 University Road, Hsinchu City 30010, Taiwan; ‡ Department of Chemistry, Chung Yuan Christian University, 200 Zhongbei Road, Taoyuan City 320314, Taiwan

## Abstract

Controlling regioselectivity in deprotonative arylation of conjugated carbonyl systems remains a longstanding challenge. We report a Pd-catalyzed γ′-arylation of cyclic vinylogous esters (CVEs), enabled by Pd­(dba)_2_/cataCXium A and hexamethylphosphoramide (HMPA) as a regioselectivity-governing additive. The method accommodates a broad range of (hetero)­aryl bromides and CVE substrates. The resulting γ′-aryl CVEs were further converted to α-arylcycloalkenones via Stork–Danheiser transposition.

## Graphical abstract

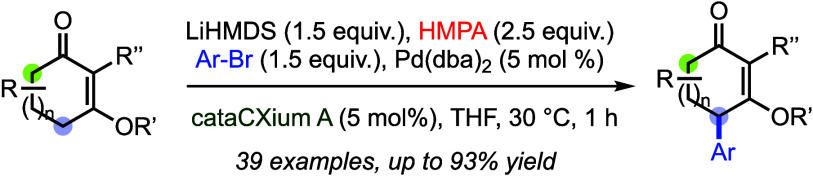

Aryl groups are ubiquitous in functional organic materials and small-molecule drugs, driving sustained interest in arylation methods.^1^ While transition-metal catalysis has made aryl–carbon bond formation routine in many settings,^2^ selective arylation at aliphatic carbon centers remains particularly valuable because it converts readily available carbonyl precursors into three-dimensional aryl-containing architectures. Among these transformations, Pd-catalyzed α-arylation of deprotonated carbonyl derivatives with aryl (pseudo)­halides has become a benchmark strategy for stitching aromatic and alkyl fragments together.^3^

In contrast, catalytic deprotonative α- or γ′-arylation of conjugated carbonyl systems remains comparatively underdeveloped, largely because regioselectivity is difficult to control and competitive conjugate addition can erode efficiency.^4^ Nevertheless, selective arylation at these positions would furnish versatile products that retain both an alkenyl handle and a carbonyl group for downstream diversification.^5^ Achieving reliable regiocontrol in the arylation of conjugated carbonyl compounds would therefore open access to underexplored, three-dimensional arylated scaffolds.

Cyclic vinylogous esters (CVEs) represent a distinctive class of conjugated carbonyl systems that feature both α- and γ′-protons amenable to deprotonation, offering multiple opportunities for site-selective functionalization.^6^ In the context of deprotonative arylation, Zhang,^7^ Lautens,^8^ and our laboratories^9^ have reported Pd-catalyzed α-arylation of CVEs with haloarenes. We subsequently showed that Pd­(OAc)_2_/tris­(1-adamantyl)­phosphine enables polyarylation of CVEs; when α’-alkyl-substituted CVEs were used, γ′-reactivity could be accessed to furnish α,γ′-diarylated and α,α,γ′-triarylated products under appropriate conditions.^10^ Importantly, however, these cascade processes proceed through initial α-arylation and require α’-alkyl substitution to unlock γ′-functionalization. Kapur and co-workers addressed γ′-arylation via a two-step sequence in which γ′-selectivity is preset through formation of conjugated silyl dienol ethers prior to a modified Kuwajima–Urabe arylation (Scheme 1a).^11^ More recently, Shao et al. reported a catalytic γ′-arylation of CVEs in which regioselectivity is proposed to arise from a [1,5]-H shift of organopalladium intermediates; this platform is most effective for α-unblocked CVEs and *ortho*-substituted aryl bromides (Scheme 1b).^12^ Overall, these studies underscore the continuing need for a direct, condition-controlled γ′-arylation of CVEs that is broadly applicable to CVE substrates and bromoarenes, including α,α-disubstituted CVEs and non-*ortho* aryl bromides (Scheme 1c). In addition, we envisioned that γ′-aryl CVEs could be leveraged through Stork–Danheiser transposition^13^ to furnish α-arylcycloalkenones, a motif prevalent in natural products and bioactive small molecules.

**Scheme 1:**
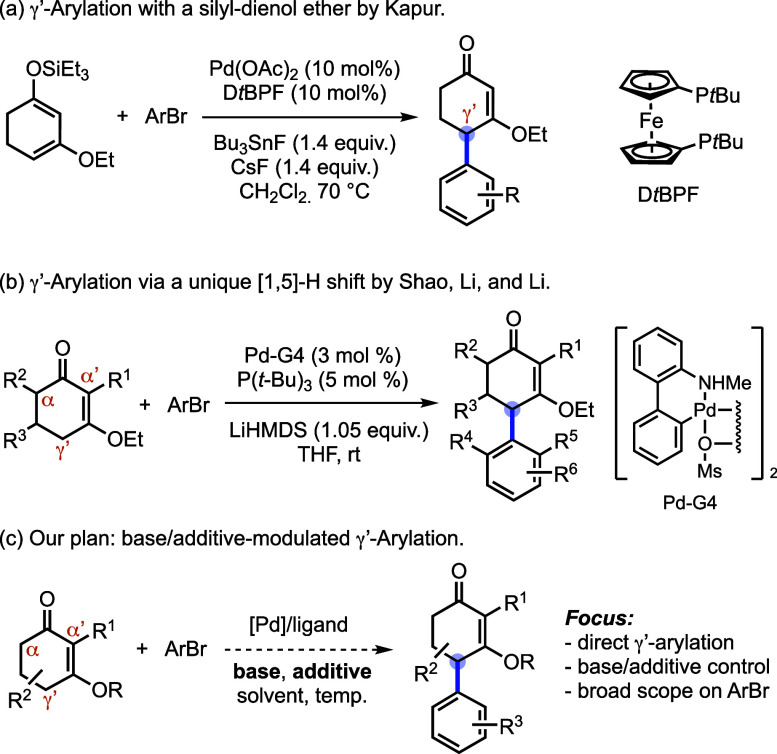
Previous and Our Approaches for γ′-Arylation of Cyclic Vinylogous Esters (CVEs)

We selected 3-ethoxy-2-cyclohexenone (**1a**) and bromobenzene as model substrates to evaluate conditions for Pd-catalyzed γ′-arylation of CVEs. Initial screening focused on bases capable of generating the requisite γ′-dienolate. Under our standard catalytic manifold,^9^ metal alkoxides furnished γ′-arylated product **2a** only in low yield (Scheme 2), and in one case a γ′,γ′-diarylated byproduct **3a** was also observed. Although these results suggested that γ′-selective arylation was feasible, further optimization of alkoxide-based conditions proved unproductive, prompting us to examine alternative base/additive combinations.

**Scheme 2:**
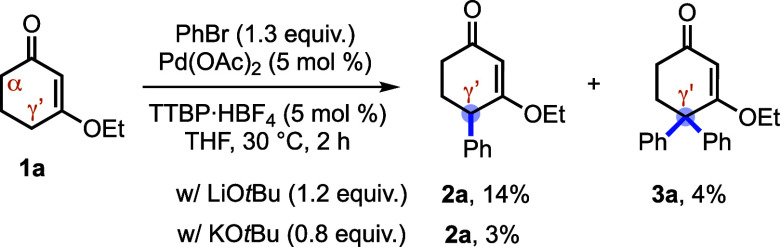
Initial Attempts on Arylation with Alkoxide Bases

Guided by our prior Pd-catalyzed α-arylation of CVEs using lithium amide bases, we evaluated LiHMDS in combination with polar additives to modulate regioselectivity. The additive proved decisive: DMPU afforded γ′-arylated product **2a** in 28% yield but also generated the competing α-arylation product **4a** in 13% yield (Table 1, entry 1), whereas 12-crown-4 largely suppressed productive coupling (entry 2). In contrast, HMPA markedly improved both efficiency and γ′/α selectivity, delivering **2**a in 45% yield with only 5% of **4a** at 2.5 equiv (entry 3). Deviating from this HMPA loading (1.5 or 3.5 equiv) diminished the outcome (entries 4 and 5). With HMPA identified as the optimal additive, we next surveyed Pd sources and ligands. Pd­(dba)_2_ was uniquely effective among the catalysts examined: Pd­(OAc)_2_ was ineffective (entry 6), while common precatalysts provided only modest yields (entries 7–9). Ligand effects were pronounced; BINAP and P­(*o*Tol)_3_ gave only trace conversion (entries 10 and 11), whereas bulky dialkylbiaryl phosphines improved the reaction. Both XPhos and PAd_2_ *n*Bu (cataCXium A)^14^ furnished **2a** in 54% yield, but PAd_2_ *n*Bu further suppressed formation of the α-arylation product **4a** (8% vs 2%; entries 13 and 14). Lowering the catalyst/ligand loading reduced efficiency (entries 15–16). Finally, premixing **1a** with HMPA and LiHMDS to generate the dienolate prior to catalyst addition increased the yield of **2a** to 65% (entry 17); the mass balance is accounted for by intractable byproducts. Under otherwise identical conditions, chlorobenzene was substantially less reactive than bromobenzene (entry 18), highlighting that the current system is optimized for aryl bromides. Control experiments confirmed that both Pd­(dba)_2_ and PAd_2_ *n*Bu are required for C–C bond formation.

**Table 1:**
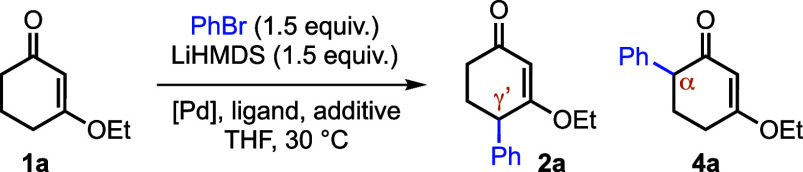
Optimization for the γ′-Arylation^a^

entry	[Pd] (mol %)	ligand (mol %)	additive (equiv)	product (%yield)^b^
1	Pd(dba)_2_ (5)	TTBP·HBF_4_ (5)	DMPU (2.5)	**2a** (28), **4a** (13)
2	Pd(dba)_2_ (5)	TTBP·HBF_4_ (5)	12C4 (2.5)	**2a** (6)
3	Pd(dba)_2_ (5)	TTBP·HBF_4_ (5)	HMPA (2.5)	**2a** (45), **4a** (5)
4	Pd(dba)_2_ (5)	TTBP·HBF_4_ (5)	HMPA (1.5)	**2a** (36), **4a** (10)
5	Pd(dba)_2_ (5)	TTBP·HBF_4_ (5)	HMPA (3.5)	**2a** (29)
6	Pd(OAc)_2_ (5)	TTBP·HBF_4_ (5)	HMPA (2.5)	trace
7	Pd-G2 (5)	TTBP·HBF_4_ (5)	HMPA (2.5)	**2a** (20)
8	Pd-G3 (5)	TTBP·HBF_4_ (5)	HMPA (2.5)	**2a** (12)
9	Pd-G4 (5)	TTBP·HBF_4_ (5)	HMPA (2.5)	**2a** (35), **4a** (6)
10	Pd(dba)_2_ (5)	BINAP (5)	HMPA (2.5)	**2a** (trace)
11	Pd(dba)_2_ (5)	P(*o*Tol)_3_ (5)	HMPA (2.5)	**2a** (trace)
12	Pd(dba)_2_ (5)	PAd_3_ (5)	HMPA (2.5)	**2a** (20), **4a** (4)
13	Pd(dba)_2_ (5)	XPhos (5)	HMPA (2.5)	**2a** (54), **4a** (8)
14	Pd(dba)_2_ (5)	PAd_2_ *n*Bu (5)	HMPA (2.5)	**2a** (54), **4a** (2)
15	Pd(dba)_2_ (3)	PAd_2_ *n*Bu (3)	HMPA (2.5)	**2a** (30)
16	Pd(dba)_2_ (2)	PAd_2_ *n*Bu (2)	HMPA (2.5)	**2a** (trace)
17^c^	Pd(dba)_2_ (5)	PAd_2_ *n*Bu (5)	HMPA (2.5)	**2a** (65)
18^d^	Pd(dba)_2_ (5)	PAd_2_ *n*Bu (5)	HMPA (2.5)	**2a** (31)

a The reactions were evaluated with 0.5 mmol of **1a**. Compound **1a** and other reagents were mixed before adding a solution of LiHMDS.

b Isolated yields.

c Compound **1a**, HMPA, and LiHMDS were premixed to generate dienolate; for more details, see Supporting Information.

d Chlorobenzene was used as the aryl donor.

The scope of aryl bromides was evaluated with **1a**. Using entry 17 (Table 2) as the optimized baseline, we found that a modest temperature increase improved conversion for several substrates. Therefore, the scope studies were conducted at 50 °C unless otherwise noted. Electron-neutral aryl bromides generally performed well, furnishing γ′-arylated products in 53–79% yield (**2a**–**g**). Electron-rich aryl bromides were similarly effective, typically providing **2h**–**n** and **2p**–**u** in 40–78% yield. Steric effects were well tolerated for mono-*ortho*-substituted aryl bromides (**2e**, **2g**, **2j**, **2p**, **2q**, **2r**, **2t**, and **2u**); however, the doubly *ortho*-substituted 2,6-dimethoxy aryl bromide led to a pronounced drop in yield (**2o**, 21%). Notably, the reaction displayed useful chemoselectivity: despite the increased steric congestion at the C–Br bond (*ortho* to the methoxy group), 2-bromo-4-chloroanisole underwent coupling exclusively at bromine, leaving the more accessible aryl chloride intact for further functionalization and furnishing **2r** in 40% yield. In contrast, aryl bromides bearing strongly electron-withdrawing substituents were less competent: *para*-F, *para*-CF_3_, and *para*-CN substrates delivered products **2v**–**x** in 13–44% yield; crude NMR analysis suggested appreciable aromatization of the CVE scaffold, producing 3-alkoxyphenol byproducts from direct CVE dehydrogenation and via γ′-arylation followed by aromatization. Additional variations in Pd source, ligand, or temperature did not substantially improve this subset. The protocol also enabled γ′-heteroarylation to afford products **2y**–**2bb** in moderate yields. Finally, the reaction was readily scalable, delivering **2q** (3.6 mmol) and **2u** (1.0 mmol) in synthetically useful yields.

**Table 2:**
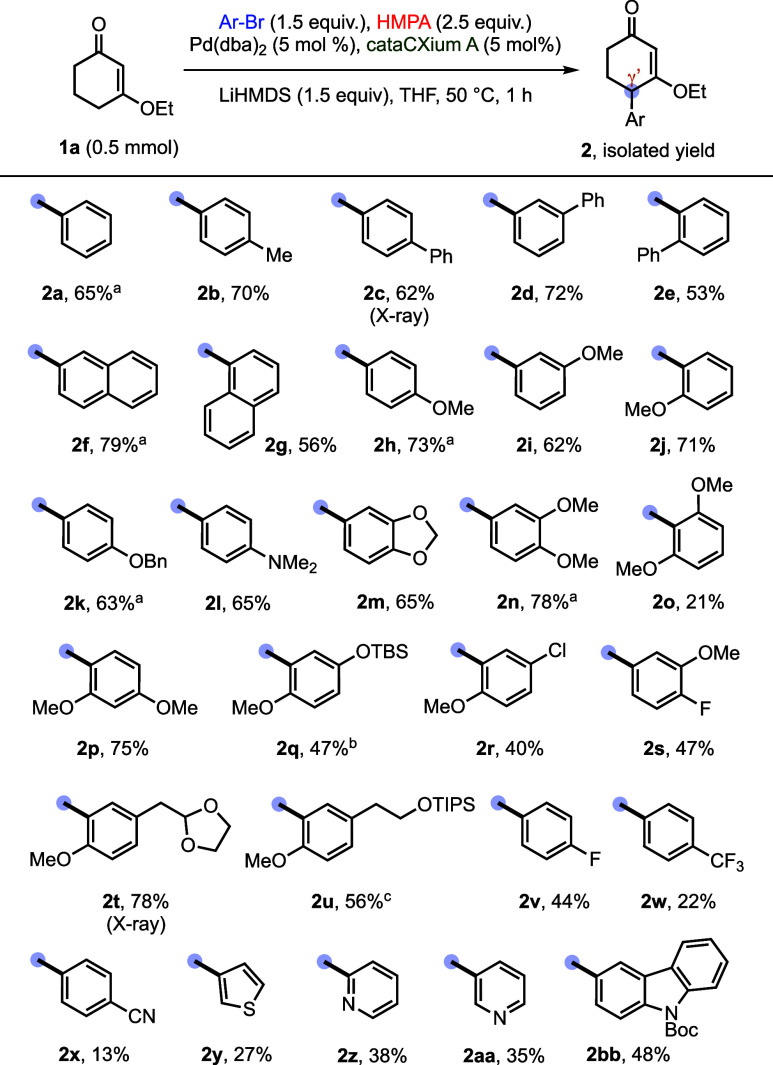
Substrate Scope on Aryl Bromides

a The reactions were conducted at 30 °C.

b The experiment was based on using 3.6 mmol of **1a**.

c The experiment was based on using 1.0 mmol of **1a**.

We next evaluated the scope of cyclic vinylogous esters (CVEs) using 4-bromoanisole and 2-bromoanisole as representative aryl donors (Table 3). Variation of the alkoxy substituent on the CVE was tolerated: *i*-propyloxy and *n*-propyloxy derivatives afforded **2cc** and **2dd** in good yields, whereas the more bulky cyclohexyloxy analogue gave diminished efficiency (**2ee**, 33%) and was accompanied by substantial decomposition/intractable material, consistent with increased steric congestion proximal to the γ′-position. Notably, a cyclic vinylogous amide also proved compatible, delivering **2ff** in 85% yield. The influence of ring size was then examined. A seven-membered CVE underwent γ′-arylation smoothly to provide **2gg** in 76% yield, while a 3-ethoxy-2-cyclopentenone substrate resulted in a complex mixture under the standard conditions. The protocol was further applicable to 1,8-dioxo-octahydroxanthene, furnishing the monoarylation product **2hh** in moderate yield. We further probed substitution effects on the CVE scaffold. An α′-methyl-substituted substrate furnished **2ii**, and an α-methyl CVE gave **2jj** as a pair of diastereomers (dr = 4:1). In contrast, a β-methyl-substituted CVE provided **2kk** with higher diastereoselectivity, plausibly reflecting a closer steric influence of the stereocontrol element. As illustrated by **2ll**, a neighboring all-carbon quaternary center reduced the efficiency of γ′-arylation. Importantly, γ′-arylation of α,α-disubstituted CVEs was achieved to afford **2 mm** (42%) and **2nn** (31%). This substrate class (lacking an α-H) notably differs from the scope reported by Shao et al., in which α,α-disubstituted CVEs were not compatible (Scheme 1b).^12^ Therefore, these results are consistent with a mechanism in which regioselectivity is established at the deprotonation stage to form a γ′-dienolate that subsequently undergoes Pd-catalyzed γ′-arylation.

**Table 3:**
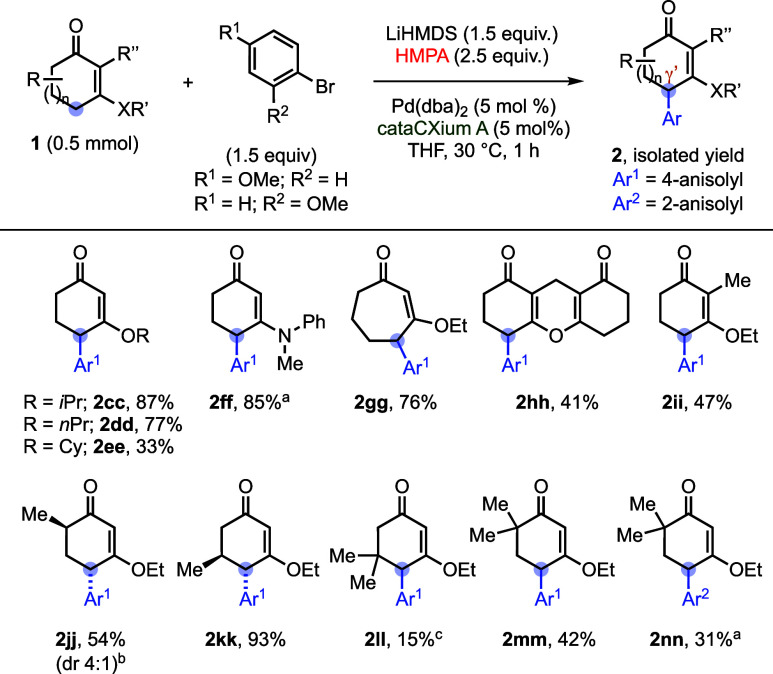
Substrate Scope on Cyclic Vinylogous Esters

a The reactions were conducted at 50 °C.

b The major diastereomer is shown.

c The reactions were conducted at 65 °C.

To probe whether regioselectivity is established at the deprotonation stage, we performed D_2_O-quenching experiments on **1a** in the absence of Pd catalyst and bromoarene. When a mixture of **1a**, LiHMDS, and HMPA was quenched with D_2_O at different time points, deuterium incorporation occurred predominantly at the γ′-position, with ∼80% γ′-deuterium incorporation maintained across 60 min. (Scheme 3a). This time-independent deuteration profile is consistent with direct and rapid γ′-selective deprotonation under HMPA-containing conditions.^15^ In contrast, in the absence of HMPA, an analogous experiment afforded primarily α-deuterated **1a**, in line with the prevailing view that lithium amides preferentially deprotonate CVEs at the α-position via a six-membered, coordination-assisted transition structure (**TS1**) to generate the kinetic dienolate.^16^ In a complementary experiment, **1a** was first reacted with LiHMDS in the absence of HMPA for 1 h, followed by addition of HMPA and subsequent D_2_O quenching at various time points (Scheme 3b). Under these conditions, the initial α-enriched deuteration pattern gradually evolved, and γ′-deuterium incorporation increased steadily, becoming dominant only after prolonged aging (>150 min; see Supporting Information). This time-dependent drift indicates that, once HMPA is introduced after initial deprotonation, interconversion among dienolate manifolds becomes feasible but proceeds on a comparatively slow time scale. Importantly, these results contrast sharply with the experiment in which HMPA is present from the outset, where the γ′-deuteration level is established rapidly and remains essentially unchanged.

**Scheme 3:**
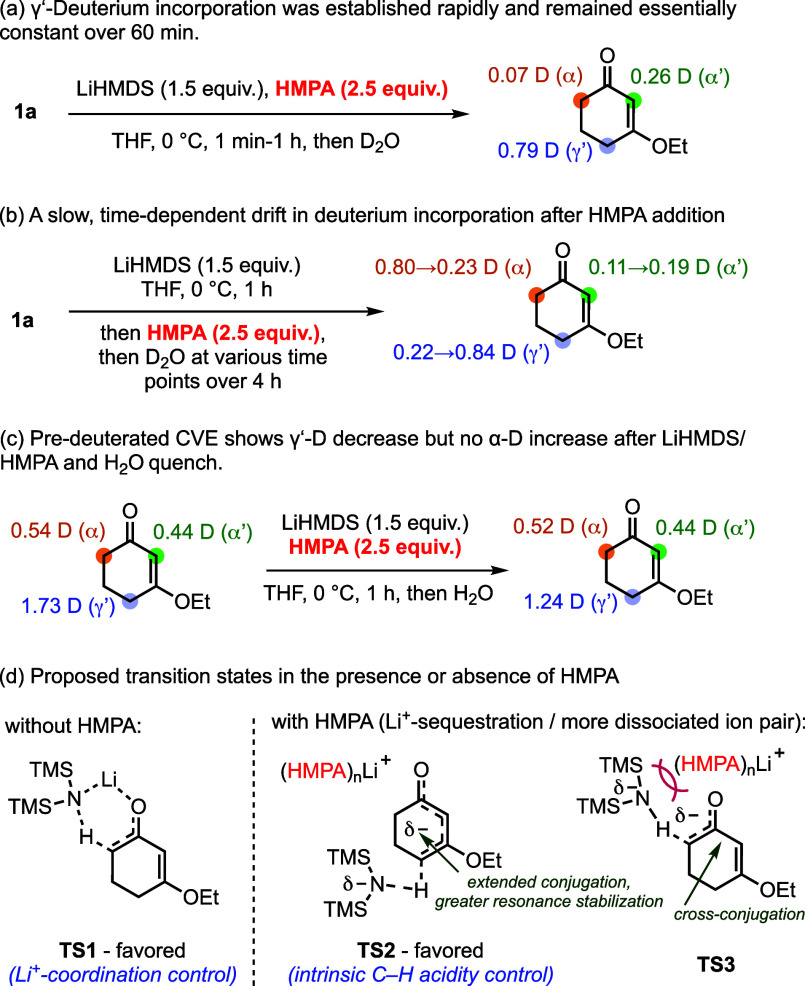
Dienolate Formation-Quenching Experiments and Proposed Transition States for the Deprotonation Events

To further assess possible H/D scrambling under the deprotonation conditions, a predeuterated CVE (γ′ = 1.73 D, α = 0.54 D) was subjected to LiHMDS/HMPA and then quenched with H_2_O (Scheme 3c). The recovered material retained essentially the same α-deuterium content (0.54 to 0.52 D), while the γ′-deuterium content decreased (1.73 to 1.24 D), consistent with γ′-selective deprotonation/reprotonation. Notably, no measurable increase of deuterium at the α position was observed, arguing against significant intramolecular 1,5-H­(D) transfer under these conditions and supporting deprotonation-controlled regioselectivity in our system. Taken together, these results suggest that HMPA governs the site of deprotonation and thereby controls the γ′/α selectivity observed in the catalytic arylation.^17^ We propose that HMPA solvates Li^+^ to promote a more dissociated ion pair,^18^ disrupting the pathway leading to **TS1**. Under such conditions, deprotonation at the γ′-position (**TS2**) becomes preferred to α-deprotonation (**TS3**), as the transition structure leading to an extended conjugated dienolate is better stabilized than that leading to a cross-conjugated dienolate.^19^ Thus, when Li^+^ sequestration overrides carbonyl coordination, deprotonation proceeds primarily under the substrate’s intrinsic C–H acidity control, shifting the kinetic deprotonation manifold from **TS1** to γ′-deprotonation via **TS2**.

As shown in Scheme 4, the present γ′-arylation can be readily combined with an established α-arylation protocol,^9,10^ enabling regioselective installation of two different aryl groups on the CVE core. This two-step sequence furnished the diarylated products **5a** (anti/syn) in good yield (dr = 4:1). Notably, the order of operations is critical: the sequence must begin with γ′-arylation and be followed by α-arylation, because preliminary experiments showed that the α-monoarylated CVE is not competent under the γ′-arylation manifold. By comparison, our previously reported cascade polyarylation is restricted to α′-alkyl-substituted CVEs and provides reduced control over regioselectivity in the second arylation step.^10a^ These results further highlight that the present protocol enables complete and programmable control over site selectivity in deprotonative arylation of CVEs.

**Scheme 4:**
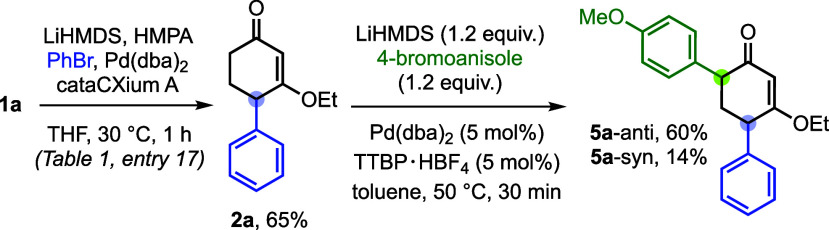
Sequential γ′- and α-Arylation

To further expand synthetic utility, we explored Stork–Danheiser transposition of γ′-arylated CVEs as an entry to α-aryl cycloalkenones. This strategy is synthetically attractive because direct α-arylation of cycloalkenones remains challenging and underdeveloped. In this sequence, carbon and hydride nucleophiles, such as MeLi, a Grignard reagent, PhCCLi, NaBH_4_, and DIBAL-H, added to the CVE carbonyl, and the resulting intermediates underwent acid-mediated hydrolysis to furnish the corresponding α-aryl cycloalkenones (Table 4). Overall, the combined γ′-arylation/Stork–Danheiser transposition provides a modular route to versatile carbocyclic building blocks **6a**–**6f**.

**Table 4:**
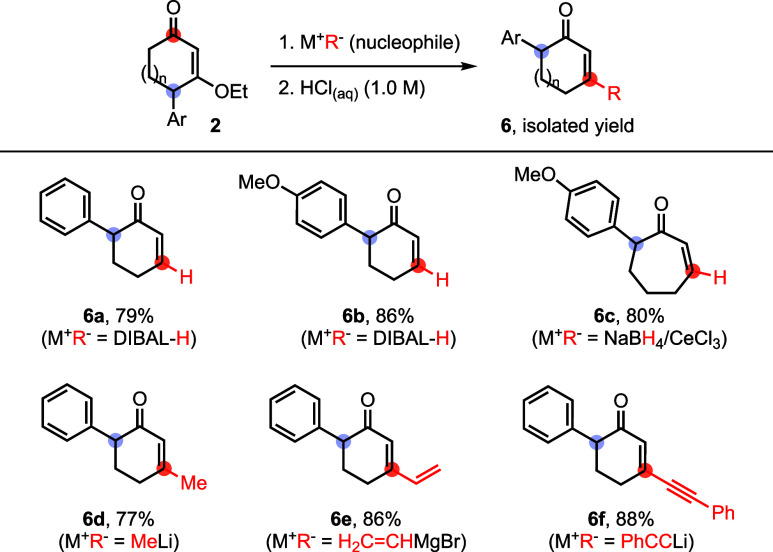
Stork–Danheiser Transposition of γ′-Arylated CVEs^a^

a For detailed procedures and conditions, see the Supporting Information.

In summary, we have developed a condition-controlled, γ′-selective monoarylation of cyclic vinylogous esters. The method can be sequenced with α-arylation to enable programmable installation of distinct aryl groups on the CVE scaffold, and it also provides access to α-aryl cycloalkenones via Stork–Danheiser transposition. Ongoing studies are focused on elucidating the role of HMPA and related polar additives in remote deprotonative arylation of conjugated carbonyl systems.

## Supplementary Material



## Data Availability

The data underlying this study are available in the published article and its Supporting Information.

## Notes

Experimental procedures, characterization data, and ORTEP structure for **2c** and **2t** (PDF)
