# Supplementary material for: HMPA-Enabled Direct γ′-Arylation of Cyclic Vinylogous Esters
Source: Org Lett. 2026 Feb 5;28(7):2534–9. doi: 10.1021/acs.orglett.6c00380 (PMC12930496; doi:10.1021/acs.orglett.6c00380)
Supplement: Supplementary file 1 [file ol6c00380_si_001.pdf]

HMPA-Enabled Direct γ'-Arylation of Cyclic Vinylogous Esters

Yan-Xun Li,[a] Wei-Ting Zhao,[a] and Yen-Ku Wu*[a,b]

[a] *Department of Applied Chemistry and Center for Emergent Functional Matter Science, National Yang Ming Chiao Tung University, 1001 University Road, Hsinchu City 30010, Taiwan*

[b] *Department of Chemistry, Chung Yuan Christian University, 200 Zhongbei Road, Taoyuan City 320314, Taiwan*

E-mail: yenkuwu@nycu.edu.tw

Supporting Information

Table of Contents

General Information……………………………………………………………… S-1

Synthesis and Characterization Data of 2, 3a, 4a, 5a, and 6………………S-2–S-35

Enolate Formation-Quenching Experiments……………………………S-36–S-40

ORTEP Structure for 2c……………………………………………………….S-41

ORTEP Structure for 2t……………………………………………………….S-42

References……………………………………………………………………S-43

NMR Spectra…………………………………………………………...S-44–S-168

General Information

All air-sensitive reactions were carried out with flame-dried glassware under a N_2 atmosphere with the Schlenk line technique. Tetrahydrofuran, toluene, 1,4-dioxane, and diethyl ether were purified via a commercial solvent purification system. Hexamethylphosphoramide (HMPA) and dimethyl sulfoxide (DMSO) were dried by 4 Å molecular sieves and stored under a N_2 atmosphere. All other solvents (ACS grade) and commercially obtained reagents were used as received. A solution of hexamethyldisilazane (HMDS) was freshly distilled over potassium hydroxide. Lithium hexamethyldisilazide (LiHMDS) was prepared before use by deprotonating HMDS in THF with *n*-BuLi (2.5M in hexanes) at 0 ºC for 10 min. Reactions were monitored by thin layer chromatography (TLC) on Merck silica gel 60 Å F254 plates and visualized via 254 nm UV and $KMnO_4$ as a color development agent. Reaction residues were purified via flash column chromatography with silica gel (230-400 mesh). Melting points were uncorrected. NMR spectra were measured on Agilent-MR DD2 (400 MHz for ^{1}H NMR spectra; 100 MHz for ^{13}C NMR spectra), JEOL JNM-ECZ400S/L1 (400 MHz for ^{1}H NMR spectra; 100 MHz for ^{13}C NMR spectra), JEOL ECZ500R/S1 (500 MHz for ^{1}H NMR spectra; 125 MHz for ^{13}C NMR spectra) or Varian VNMRS-600 NMR spectrometer (600 MHz for ^{1}H NMR spectra; 150 MHz for ^{13}C NMR spectra) and calibrated from residual solvent signals such as chloroform-d$_1$ (δ_H = 7.26 ppm; δ_C = 77.00 ppm). Chemical shifts were denoted in ppm (δ), and the following abbreviations were used to explain multiplicities: s = singlet, br s = broad singlet, d = doublet, t = triplet, q = quartet, m = multiplet. Coupling constants (*J*) were mentioned in Hertz. Infrared (IR) spectra were measured on Thermo Nicolet iS5 FT-IR spectrometer with ATR sampling system and were reported as wavenumber (cm^{-1}). High-resolution mass spectroscopy (HRMS) was performed on a TOF instrument with ESI or EI in positive ionization mode. X-ray diffraction was measured on Rigaku

XtaLAB Synergy DW. Structural assignments were made with additional information from NOESY, gHSQC, and gHMBC experiments.

Synthesis and Characterization Data of 2, 3a, 4a, 5a, and 6.

General Procedure for the γ'-Arylation.

To a round-bottom flask containing cyclic vinylogous ester (0.5 mmol), HMPA (2.5 equiv., 1.25 mmol, 220 μL) was added THF (2.5 mL) at room temperature, and the solution was cooled to 0 °C (an ice bath). A solution of LiHMDS (0.62 M, 1.5 equiv., 1.21 mL) was slowly added to the mixture at 0 °C and then stirred for 1 hour. At the same time, another round-bottom flask containing aryl bromide (1.3 equiv., 0.65 mmol), $Pd(dba)_2$ (5 mol%, 14 mg), $P(Ad)_2Bu$ (5 mol%, 9 mg) was evacuated and backfilled with N_2 3 times. After adding THF (1.5 mL), the mixture was stirred at room temperature for 30 minutes. Subsequently, the solution of enolate was transferred to the flask containing the catalyst, and it was heated to the indicated temperature (30 °C or 50 °C in oil bath). After stirring for the indicated time, the reaction was quenched with water, and the mixture was diluted with ethyl acetate and extracted with saturated brine. The organic layer was filtered through a pad of celite, dried over anhydrous $MgSO_{4(s)}$, and concentrated with a rotary evaporator. The crude residue was purified by flash column chromatography

6-Ethoxy-2,3-dihydro-[1,1'-biphenyl]-4(1*H*)-one (2a)[1]

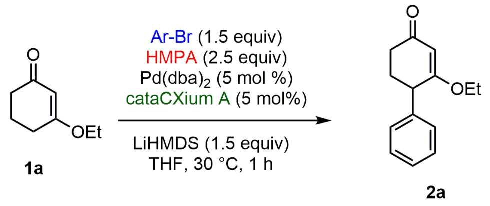

The reaction was conducted with 0.5 mmol of **1a** following the general procedure

(reaction temperature = 30 ºC; reaction time = 1 hour). The crude product was purified by flash column chromatography (EtOAc/hexanes/DCM = 1/3/1) to afford **2a** (70 mg, 65%) as a pale-yellow solid (m.p. 63-64 ºC). R*f*: 0.25 (EtOAc/hexanes/DCM = 1/3/1). **^{1}H NMR** (400 MHz, $CDCl_3$): δ 7.35-7.29 (m, 2H), 7.28-7.22 (m, 1H), 7.20-7.15 (m, 2H), 5.57 (s, 1H), 4.01-3.84 (m, 2H), 3.75 (t, *J* = 4.8 Hz, 1H), 2.44-2.22 (m, 3H), 2.08-2.00 (m, 1H), 1.25 (t, *J* = 7.0 Hz, 3H); **^{13}C NMR** (100 MHz, $CDCl_3$): δ 199.7, 177.2, 139.7, 128.6, 127.7, 126.9, 104.4, 64.4, 44.7, 33.2, 30.2, 13.9. ^{1}H NMR data of **2a** are consistent with those reported in the literature.[1]

6-Ethoxy-4'-methyl-2,3-dihydro-[1,1'-biphenyl]-4(1*H*)-one (2b)

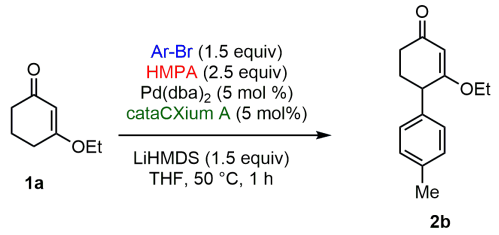

The reaction was conducted with 0.5 mmol of **1a** following the general procedure (reaction temperature = 50 ºC; reaction time = 1 hour). The crude product was purified by flash column chromatography (EtOAc/hexanes/DCM = 1/3/1) to afford **2b** (81 mg, 70%) as a colorless solid (m.p. 62-63 ºC). R*f*: 0.3 (EtOAc/hexanes/DCM = 1/3/1). **IR** (cast): 2942, 1650, 1597, 1511 cm^{-1}; **^{1}H NMR** (400 MHz, $CDCl_3$): δ 7.14 (d, *J* = 8.1 Hz, 2H), 7.08 (d, *J* = 8.1 Hz, 2H), 5.57 (s, 1H), 4.00-3.85 (m, 2H), 3.72 (t, *J* = 4.5 Hz, 1H), 2.41-2.22 (m, 6H), 2.05-1.96 (m, 1H), 1.27 (t, *J* = 7.0 Hz, 3H); **^{13}C NMR** (100 MHz, $CDCl_3$): δ 199.8, 177.5, 136.6, 136.6, 129.3, 127.5, 104.3, 64.4, 44.3, 33.2, 30.2, 20.9, 13.9; **HRMS** (ESI) *m/z*: $[M+H]^+$ calcd. for $C_{15}H_{19}O_2$ 231.1380, found: 231.1383.

6-Ethoxy-2,3-dihydro-[1,1':4',1''-terphenyl]-4(1*H*)-one (2c)

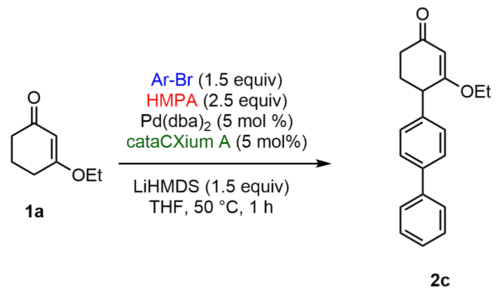

The reaction was conducted with 0.5 mmol of **1a** following the general procedure (reaction temperature = 50 ºC; reaction time = 1 hour). The crude product was purified by flash column chromatography (EtOAc/hexanes/DCM = 1/3/1) to afford **2c** (90 mg, 62%) as white powder (m.p. 117-118 ºC). R_f: 0.25 (EtOAc/hexanes/DCM = 1/3/1). **IR** (cast): 2931, 1651, 1596, 1486 cm^{-1}; **^{1}H NMR** (400 MHz, $CDCl_3$): δ 7.60-7.55 (m, 4H), 7.47-7.41 (m, 2H), 7.37-7.32 (m, 1H), 7.28-7.26 (m, 2H), 5.62 (s, 1H), 4.04-3.90 (m, 2H), 3.81 (t, J = 4.6 Hz, 1H), 2.47-2.26 (m, 3H), 2.12-2.05 (m, 1H), 1.30 (t, J = 7.0 Hz, 3H); **^{13}C NMR** (100 MHz, $CDCl_3$): δ 199.7, 177.2, 140.6, 139.9, 138.7, 128.7, 128.1, 127.3, 127.0, 104.4, 64.5, 44.4, 33.2, 30.2, 13.9 [One sp^2 carbon is missing due to peak overlapping]; **HRMS** (ESI) *m/z*: $[M+H]^+$ calcd. for $C_{20}H_{21}O_2$ 293.1536, found: 293.1536.

6-Ethoxy-2,3-dihydro-[1,1':3',1''-terphenyl]-4(1*H*)-one (2d)

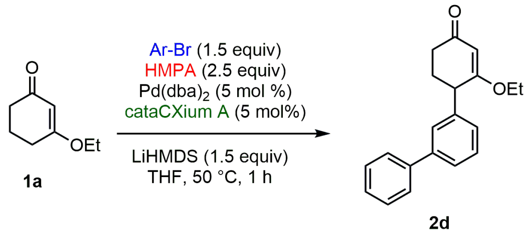

The reaction was conducted with 0.5 mmol of **1a** following the general procedure (reaction temperature = 50 ºC; reaction time = 1 hour). The crude product was purified by flash column chromatography (EtOAc/hexanes/DCM = 1/3/1) to afford **2d** (105 mg, 72%) as a yellow viscous oil. R_f: 0.23 (EtOAc/hexanes/DCM = 1/3/1). **IR** (film): 2934, 1651, 1595, 1478 cm^{-1}; **^{1}H NMR** (400 MHz, $CDCl_3$): δ 7.58-7.54 (m, 2H), 7.51-7.48

(m, 1H), 7.47-7.39 (m, 4H), 7.38-7.33 (m, 1H), 7.19-7.16 (m, 1H), 5.62 (s, 1H), 4.04-3.88 (m, 2H), 3.83 (t, *J* = 4.8 Hz, 1H), 2.48-2.27 (m, 3H), 2.14-2.07 (m, 1H), 1.29 (t, *J* = 7.0 Hz, 3H); **^{13}C NMR** (100 MHz, $CDCl_3$): δ 199.7, 177.1, 141.6, 140.8, 140.2, 128.9, 128.8, 127.4, 127.1, 126.6, 126.5, 125.9, 104.5, 64.4, 44.7, 33.2, 30.2, 13.9; **HRMS** (ESI) *m/z*: $[M+H]^+$ calcd. for $C_{20}H_{21}O_2$ 293.1536 found: 293.1532.

6-Ethoxy-2,3-dihydro-[1,1':2',1''-terphenyl]-4(1*H*)-one (2e)[1]

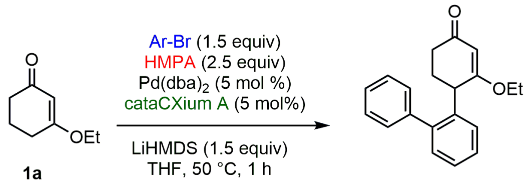

2e

The reaction was conducted with 0.5 mmol of **1a** following the general procedure (reaction temperature = 50 ºC; reaction time = 1 hour). The crude product was purified by flash column chromatography (EtOAc/hexanes/DCM = 1/3/1) to afford **2e** (78 mg, 53%) as a pale-yellow solid (m.p. 120-121 ºC). R*f*: 0.25 (EtOAc/hexanes/DCM = 1/3/1). **^{1}H NMR** (400 MHz, $CDCl_3$): δ 7.46-7.41 (m, 2H), 7.40-7.37 (m, 1H), 7.37-7.33 (m, 2H), 7.33-7.29 (m, 2H), 7.27-7.25 (m, 1H), 7.21-7.18 (m, 1H), 5.56 (s, 1H), 4.00 (t, *J* = 6.0 Hz, 1H), 3.97-3.84 (m, 2H), 2.41-2.32 (m, 1H), 2.23-2.15 (m, 1H), 2.07-1.99 (m, 1H), 1.89-1.80 (m, 1H), 1.26 (t, *J* = 7.0 Hz, 3H). ^{1}H NMR data of **2e** are consistent with those reported in the literature.[1]

3-Ethoxy-4-(naphthalen-2-yl)cyclohex-2-en-1-one (2f)[1]

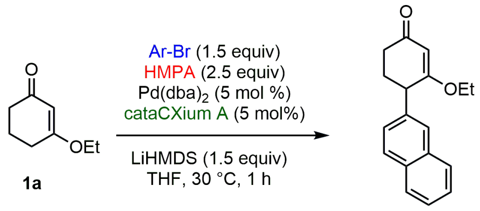

2f

The reaction was conducted with 0.5 mmol of **1a** following the general procedure

(reaction temperature = 30 ºC; reaction time = 1 hour). The crude product was purified by flash column chromatography (EtOAc/hexanes/DCM = 1/3/1) to afford **2f** (105 mg, 79%) as a white solid (m.p. 79-80 ºC). R_f: 0.25 (EtOAc/hexanes/DCM = 1/3/1). **^{1}H NMR** (400 MHz, $CDCl_3$): δ 7.86-7.77 (m, 3H), 7.62 (br s, 1H), 7.51-7.44 (m, 2H), 7.33 (dd, J = 8.5, 1.9 Hz, 1H), 5.66 (s, 1H), 4.04-3.90 (m, 3H), 2.50-2.27 (m, 3H), 2.18-2.10 (m, 1H), 1.26 (t, J = 7.0 Hz, 3H). ^{1}H NMR data of **2f** are consistent with those reported in the literature.[1]

3-Ethoxy-4-(naphthalen-1-yl)cyclohex-2-en-1-one (2g)

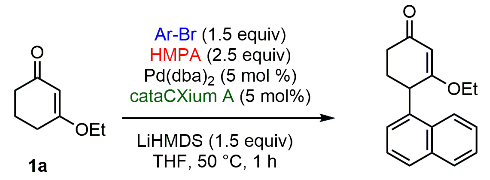

The reaction was conducted with 0.5 mmol of **1a** following the general procedure (reaction temperature = 50 ºC; reaction time = 1 hour). The crude product was purified by flash column chromatography (EtOAc/hexanes/DCM = 1/3/1) to afford **2g** (74 mg, 56%) as a yellow viscous oil. R_f: 0.23 (EtOAc/hexanes/DCM = 1/3/1). **IR** (film): 2937, 1650, 1599, 1339 cm^{-1}; **^{1}H NMR** (400 MHz, $CDCl_3$): δ 8.05 (d, J = 8.4 Hz, 1H), 7.91 (dd, J = 7.8, 1.6 Hz, 1H), 7.80 (d, J = 8.2 Hz, 1H), 7.59-7.50 (m, 2H), 7.41 (dd, J = 8.2, 7.2 Hz, 1H), 7.28 (br s, 1H), 5.73 (s, 1H), 4.56 (dd, J = 5.7, 3.4 Hz, 1H), 4.07-3.90 (m, 2H), 2.54-2.43 (m, 1H), 2.31-2.19 (m, 3H), 1.25 (t, J = 7.0 Hz, 3H); **^{13}C NMR** (100 MHz, $CDCl_3$): δ 199.9, 177.6, 134.6, 134.3, 131.3, 129.1, 127.8, 126.3, 125.7, 125.1, 124.4, 122.9, 105.2, 64.53, 41.0, 33.3, 28.1, 13.9; **HRMS** (ESI) *m/z*: $[M+H]^+$ calcd. for $C_{18}H_{19}O_2$ 267.1380, found: 267.1379.

6-Ethoxy-4'-methoxy-2,3-dihydro-[1,1'-biphenyl]-4(1*H*)-one (2h)[1]

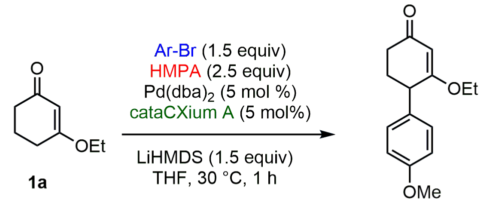

The reaction was conducted with 0.5 mmol of **1a** following the general procedure (reaction temperature = 30 ºC; reaction time = 1 hour). The crude product was purified by flash column chromatography (EtOAc/hexanes/DCM = 1/2/1) to afford **2h** (90 mg, 73%) as a yellow viscous oil. R_f: 0.23 (EtOAc/hexanes/DCM = 1/3/1). **^{1}H NMR** (400 MHz, $CDCl_3$): δ 7.10 (d, J = 8.7 Hz, 2H), 6.85 (d, J = 8.7 Hz, 2H), 5.55 (s, 1H), 4.00-3.84 (m, 2H), 3.78 (s, 3H), 3.70 (t, J = 4.3 Hz, 1H), 2.38-2.21 (m, 3H), 2.04-1.95 (m, 1H), 1.26 (t, J = 7.0 Hz, 3H). ^{1}H NMR data of **2h** are consistent with those reported in the literature.[1]

6-Ethoxy-3'-methoxy-2,3-dihydro-[1,1'-biphenyl]-4(1*H*)-one (2i)

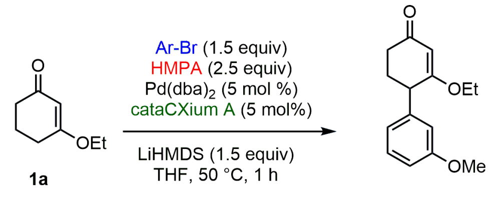

The reaction was conducted with 0.5 mmol of **1a** following the general procedure (reaction temperature = 50 ºC; reaction time = 1 hour). The crude product was purified by flash column chromatography (EtOAc/hexanes/DCM = 1/3/1) to afford **2i** (76 mg, 62%) as a yellow viscous oil. R_f: 0.2 (EtOAc/hexanes/DCM = 1/3/1). **IR** (film): 2948, 1652, 1595, 1486 cm^{-1}; **^{1}H NMR** (400 MHz, $CDCl_3$): δ 7.27-7.23 (m, 1H), 6.83-6.77 (m, 2H), 6.74 (t, J = 2.1 Hz, 1H), 5.58 (s, 1H), 4.02-3.86 (m, 2H), 3.80 (s, 3H), 3.73 (t, J = 4.5 Hz, 1H), 2.43-2.23 (m, 3H), 2.08-2.01 (m, 1H), 1.28 (t, J = 7.0 Hz, 3H); **^{13}C NMR** (100 MHz, $CDCl_3$): δ 199.7, 177.1, 159.7, 141.2, 129.5, 120.0, 113.9, 111.8, 104.4, 64.4, 55.1, 44.6, 33.2, 30.1, 13.9; **HRMS** (ESI) *m/z*: $[M+H]^+$ calcd. for $C_{15}H_{19}O_3$

247.1329, found: 247.1327.

6-Ethoxy-2'-methoxy-2,3-dihydro-[1,1'-biphenyl]-4(1*H*)-one (2j)[1]

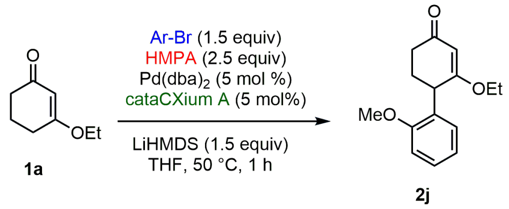

The reaction was conducted with 0.5 mmol of **1a** following the general procedure (reaction temperature = 50 ºC; reaction time = 1 hour). The crude product was purified by flash column chromatography (EtOAc/hexanes/DCM = 1/3/1) to afford **2j** (87 mg, 71%) as a pale-yellow viscous oil. R*f*: 0.25 (EtOAc/hexanes/DCM = 1/3/1). **^{1}H NMR** (400 MHz, $CDCl_3$): δ 7.28-7.22 (m, 1H), 7.02 (dd, *J* = 7.5, 1.8 Hz, 1H), 6.93-6.86 (m, 2H), 5.60 (s, 1H), 4.14-4.12 (m, 1H), 4.00-3.86 (m, 2H), 3.85 (s, 3H), 2.30-2.18 (m, 3H), 2.10-2.04 (m, 1H), 1.26 (t, *J* = 7.0 Hz, 3H). ^{1}H NMR data of **2j** are consistent with those reported in the literature.[1]

4'-(Benzyloxy)-6-ethoxy-2,3-dihydro-[1,1'-biphenyl]-4(1*H*)-one (2k)

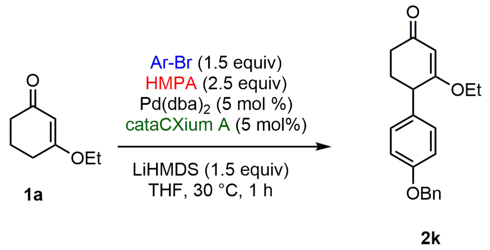

The reaction was conducted with 0.5 mmol of **1a** following the general procedure (reaction temperature = 30 ºC; reaction time = 1 hour). The crude product was purified by flash column chromatography (EtOAc/hexanes/DCM = 1/3/1) to afford **2k** (101 mg, 63%) as a white solid (m.p. 127-128 ºC). R*f*: 0.28 (EtOAc/hexanes/DCM = 1/3/1). **IR** (cast): 2937, 1647, 1597, 1507 cm^{-1}; **^{1}H NMR** (400 MHz, $CDCl_3$): δ 7.48-7.34 (m, 4H),

7.38-7.28 (m, 1H), 7.11 (d, J = 8.6 Hz, 2H), 6.94 (d, J = 8.6 Hz, 2H), 5.57 (s, 1H), 5.05 (s, 2H), 4.03-3.83 (m, 2H), 3.71 (t, J = 4.4 Hz, 1H), 2.40-2.21 (m, 3H), 2.03-1.97 (m, 1H), 1.28 (t, J = 7.0 Hz, 3H); **^{13}C NMR** (100 MHz, $CDCl_3$): δ 199.9, 177.6, 157.8, 136.9, 131.9, 128.7, 128.6, 128.0, 127.5, 114.9, 104.2, 70.0, 64.4, 43.9, 33.2, 30.3, 13.9; **HRMS** (ESI) *m/z*: $[M+H]^+$ calcd. for $C_{21}H_{23}O_3$ 323.1642, found: 323.1640.

4'-(Dimethylamino)-6-ethoxy-2,3-dihydro-[1,1'-biphenyl]-4(1*H*)-one (2l)

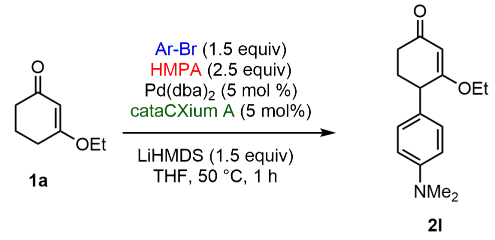

The reaction was conducted with 0.5 mmol of **1a** following the general procedure (reaction temperature = 50 ºC; reaction time = 1 hour). The crude product was purified by flash column chromatography (EtOAc/hexanes = 1/3) to afford **2l** (85 mg, 65%) as a pale-yellow viscous oil. R*f*: 0.25 (EtOAc/hexanes/DCM = 1/3/1). **IR** (film): 2941, 2890, 1653, 1647, 1519 cm^{-1}; **^{1}H NMR** (400 MHz, $CDCl_3$): δ 7.06 (d, J = 8.6 Hz, 2H), 6.70 (d, J = 8.6 Hz, 2H), 5.55 (s, 1H), 4.00-3.85 (m, 2H), 3.67 (t, J = 4.3 Hz, 1H), 2.94 (s, 6H), 2.39-2.21 (m, 3H), 2.01-1.94 (m, 1H), 1.28 (t, J = 7.0 Hz, 3H); **^{13}C NMR** (100 MHz, $CDCl_3$): δ 200.1, 178.1, 149.5, 128.3, 127.2, 112.7, 104.1, 64.3, 43.7, 40.6, 33.1, 30.3, 13.9; **HRMS** (ESI) *m/z*: $[M+H]^+$ calcd. for $C_{16}H_{22}NO_2$ 260.1645, found: 260.1672.

4-(Benzo[*d*][1,3]dioxol-5-yl)-3-ethoxycyclohex-2-en-1-one (2m)

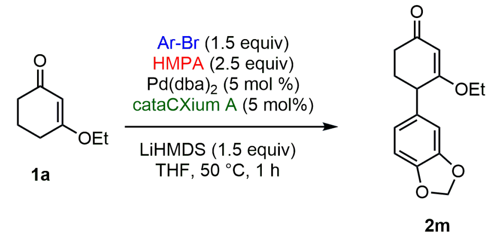

The reaction was conducted with 0.5 mmol of **1a** following the general procedure

(reaction temperature = 50 ºC; reaction time = 1 hour). The crude product was purified by flash column chromatography (EtOAc/hexanes/DCM = 1/3/1) to afford **2m** (84 mg, 65%) as a yellow viscous oil. R*f*: 0.2 (EtOAc/hexanes/DCM = 1/3/1). **IR** (film): 2894, 1650, 1595, 1503 cm^{-1}; **^{1}H NMR** (400 MHz, $CDCl_3$): δ 6.77 (d, *J* = 7.9 Hz, 1H), 6.68 (d, *J* = 1.7 Hz, 1H), 6.65 (dd, *J* = 7.9, 1.7 Hz, 1H), 5.96-5.94 (m, 2H), 5.55 (s, 1H), 4.01-3.85 (m, 2H), 3.69-3.65 (m, 1H), 2.39-2.22 (m, 3H), 2.05-1.93 (m, 1H), 1.29 (t, *J* = 7.0 Hz, 3H); **^{13}C NMR** (100 MHz, $CDCl_3$): δ 199.7, 177.2, 147.8, 146.5, 133.4, 120.7, 108.3, 108.1, 104.3, 101.0, 64.4, 44.3, 33.17, 30.3, 13.9; **HRMS** (ESI) *m/z*: $[M+H]^+$ calcd. for $C_{15}H_{17}O_4$ 261.1121, found: 261.1124.

6-Ethoxy-3',4'-dimethoxy-2,3-dihydro-[1,1'-biphenyl]-4(1*H*)-one (2n)[1]

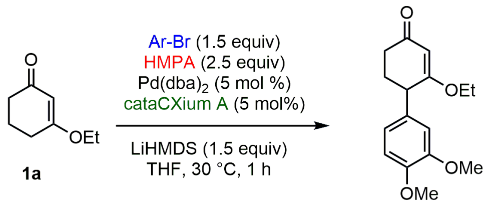

The reaction was conducted with 0.5 mmol of **1a** following the general procedure (reaction temperature = 30 ºC; reaction time = 1 hour). The crude product was purified by flash column chromatography (EtOAc/hexanes/DCM = 2/2/1) to afford **2n** (108 mg, 78%) as a yellow viscous oil. R*f*: 0.28 (EtOAc/hexanes/DCM = 2/2/1). **^{1}H NMR** (400 MHz, $CDCl_3$): δ 6.82 (d, *J* = 8.0 Hz, 1H), 6.75-6.70 (m, 2H), 5.57 (s, 1H), 4.00-3.88 (m, 2H), 3.87 (s, 3H), 3.86 (s, 3H), 3.71-3.69 (m, 1H), 2.39-2.25 (m, 3H), 2.07-1.98 (m, 1H), 1.29 (t, *J* = 7.1 Hz, 3H). ^{1}H NMR data of **2n** are consistent with those reported in the literature.[1]

6-Ethoxy-2',6'-dimethoxy-2,3-dihydro-[1,1'-biphenyl]-4(1*H*)-one (2o)[1]

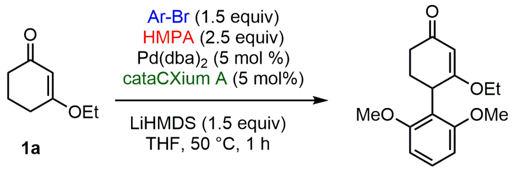

The reaction was conducted with 0.5 mmol of **1a** following the general procedure (reaction temperature = 50 ºC; reaction time = 1 hour). The crude product was purified by flash column chromatography (EtOAc/hexanes/DCM = 1/2/1 to 1/1/1) to afford **2o** (29 mg, 21%) as a white solid (m.p. 120-121 ºC). R_f: 0.20 (EtOAc/hexanes/DCM = 1/1/1). **^{1}H NMR** (400 MHz, $CDCl_3$): δ 7.19 (t, J = 8.3 Hz, 1H), 6.54 (br s, 2H), 5.43 (d, J = 1.7 Hz, 1H), 4.40 (ddd, J = 11.0, 5.0, 1.7 Hz, 1H), 3.97-3.64 (m, 8H), 2.48-2.44 (m, 2H), 2.37-2.26 (m, 1H), 1.99-1.93 (m, 1H), 1.16 (t, J = 7.0 Hz, 3H). ^{1}H NMR data of **2o** are consistent with those reported in the literature.[1]

6-Ethoxy-2',4'-dimethoxy-2,3-dihydro-[1,1'-biphenyl]-4(1*H*)-one (2p)[1]

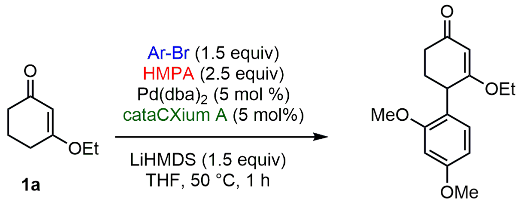

The reaction was conducted with 0.5 mmol of **1a** following the general procedure (reaction temperature = 50 ºC; reaction time = 1 hour). The crude product was purified by flash column chromatography (EtOAc/hexanes/DCM = 1/3/1) to afford **2p** (104 mg, 75%) as a pale-yellow viscous oil. R_f: 0.18 (EtOAc/hexanes/DCM = 1/3/1). **^{1}H NMR** (400 MHz, $CDCl_3$): δ 6.92 (d, J = 8.3 Hz, 1H), 6.49 (d, J = 2.4 Hz, 1H), 6.41 (dd, J = 8.3, 2.4 Hz, 1H), 5.60 (s, 1H), 4.04 (t, J = 4.7 Hz, 1H), 3.99-3.86 (m, 2H), 3.82 (s, 3H), 3.80 (s, 3H), 2.31-2.15 (m, 3H), 2.07-2.00 (m, 1H), 1.27 (t, J = 7.0 Hz, 3H). ^{1}H NMR data of **2p** are consistent with those reported in the literature.[1]

5'-((tert-Butyldimethylsilyl)oxy)-6-ethoxy-2'-methoxy-2,3-dihydro-[1,1'-bipheny-l]-4(1*H*)-one (2q)

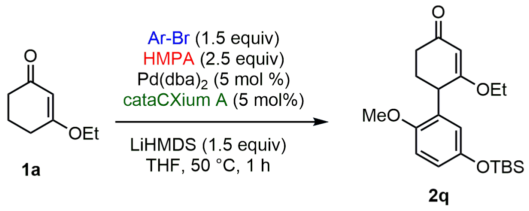

The reaction was conducted with 3.6 mmol of **1a** following the general procedure (reaction temperature = 50 ºC; reaction time = 1 hour). The crude product was purified by flash column chromatography (EtOAc/hexanes/DCM = 1/5/1) to afford **2q** (638 mg, 47%) as a brown solid (m.p. 81-82 ºC). R_f: 0.52 (EtOAc/hexanes/DCM =1/3/1). **IR** (cast): 2926, 2855, 1661, 1604, 1494 cm^{-1}; ^{1}**H NMR** (400 MHz, $CDCl_3$): δ 6.75 (d, *J* = 8.7 Hz, 1H), 6.70 (dd, *J* = 8.7, 2.8 Hz, 1H), 6.52 (d, *J* = 2.8 Hz, 1H), 5.59 (s, 1H), 4.08-4.05 (m, 1H), 3.99-3.83 (m, 2H), 3.79 (s, 3H), 2.29-2.19 (m, 3H), 2.09-2.02 (m, 1H), 1.26 (t, *J* = 7.0 Hz, 3H), 0.93 (s, 9H), 0.12 (s, 6H); ^{13}**C NMR** (100 MHz, $CDCl_3$): δ 200.2, 177.9, 151.7, 148.9, 128.3, 119.8, 118.7, 111.4, 104.7, 64.3, 55.7, 38.8, 33.6, 27.6, 25.7, 18.2, 13.9, -4.5; **HRMS** (ESI) *m/z*: $[M+H]^+$ calcd. for $C_{21}H_{33}O_4Si$ 377.2143, found: 377.2155.

5'-Chloro-6-ethoxy-2'-methoxy-2,3-dihydro-[1,1'-biphenyl]-4(1*H*)-one (2r)

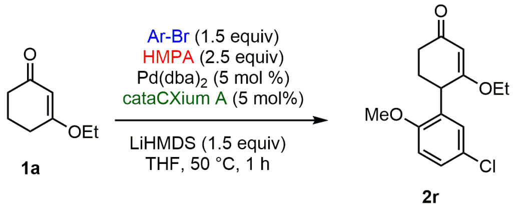

The reaction was conducted with 0.5 mmol of **1a** following the general procedure (reaction temperature = 50 ºC; reaction time = 1 hour). The crude product was purified by flash column chromatography (EtOAc/hexanes/DCM = 1/3/1) to afford **2r** (56 mg, 40%) as a pale-yellow solid (m.p. 129-130 ºC). R_f: 0.23 (EtOAc/hexanes/DCM = 1/3/1).

IR (cast): 2939, 1652, 1599, 1486 cm^{-1}; ^{1}**H NMR** (400 MHz, $CDCl_3$): δ 7.21 (dd, *J* = 8.7, 2.6 Hz, 1H), 6.98 (d, *J* = 2.6 Hz, 1H), 6.82 (d, *J* = 8.7 Hz, 1H), 5.61 (s, 1H), 4.08 (t, *J* = 4.9 Hz, 1H), 3.99-3.89 (m, 2H), 3.84 (s, 3H), 2.31-2.18 (m, 3H), 2.10-2.01 (m, 1H), 1.28 (t, *J* = 7.0 Hz, 3H); ^{13}**C NMR** (100 MHz, $CDCl_3$): δ 199.8, 177.1, 155.7, 129.5, 127.8, 127.7, 125.3, 111.8, 104.8, 64.4, 55.6, 38.9, 33.7, 27.4, 13.9; **HRMS** (ESI) *m/z*: $[M+H]^+$ calcd. for $C_{15}H_{18}ClO_3$ 281.0939, found: 281.0942.

6-Ethoxy-4'-fluoro-3'-methoxy-2,3-dihydro-[1,1'-biphenyl]-4(1*H*)-one (2s)

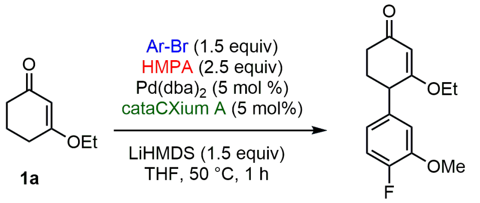

The reaction was conducted with 0.5 mmol of **1a** following the general procedure (reaction temperature = 50 ºC; reaction time = 1 hour). The crude product was purified by flash column chromatography (EtOAc/hexanes/DCM = 1/3/1 to 1/2/1) to afford **2s** (62 mg, 47%) as a yellow viscous oil. R_f: 0.2 (EtOAc/hexanes/DCM = 1/3/1). **IR** (film): 2939, 1651, 1595, 1515 cm^{-1}; ^{1}**H NMR** (400 MHz, $CDCl_3$): δ 7.02 (dd, *J* = 11.1, 8.3 Hz, 1H), 6.79 (dd, *J* = 8.0, 2.2 Hz, 1H), 6.71 (ddd, *J* = 8.3, 4.2, 2.2 Hz, 1H), 5.57 (s, 1H), 4.02-3.89 (m, 2H), 3.87 (s, 3H), 3.72 (t, *J* = 4.7 Hz, 1H), 2.41-2.28 (m, 3H), 2.06-1.97 (m, 1H), 1.28 (t, *J* = 7.0 Hz, 3H); ^{13}**C NMR** (100 MHz, $CDCl_3$): δ 199.5, 176.8, 151.5 (d, J_{C-F} = 245.6 Hz), 147.6 (d, J_{C-F} = 10.9 Hz), 135.9, 119.8 (d, J_{C-F}= 6.8 Hz), 115.9 (d, J_{C-F} = 18.3 Hz), 113.1, 104.4, 64.5, 56.3, 44.4, 33.3, 30.2, 13.9; ^{19}**F NMR** (376 MHz, $CDCl_3$): δ -137.4; **HRMS** (ESI) *m/z*: $[M+H]^+$ calcd. for $C_{15}H_{18}FO_3$ 265.1234 found: 265.1237.

5'-((1,3-Dioxolan-2-yl)methyl)-6-ethoxy-2'-methoxy-2,3-dihydro-[1,1'-biphenyl]-

4(1*H*)-one (2t)

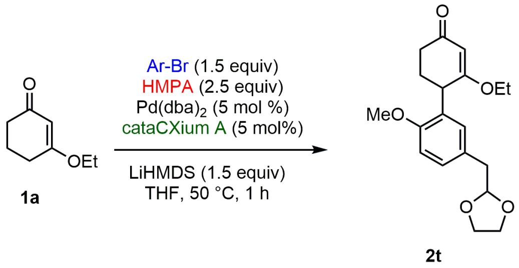

The reaction was conducted with 0.5 mmol of **1a** following the general procedure (reaction temperature = 50 ºC; reaction time = 1 hour). The crude product was purified by flash column chromatography (EtOAc/hexanes = 1/1) to afford **2t** (130 mg, 78%) as a yellow solid (m.p. 97-98 ºC). R_f: 0.25 (EtOAc/hexanes = 1/1). **IR** (cast): 2954, 2889, 1649, 1600, 1500 cm^{-1}; **^{1}H NMR** (400 MHz, $CDCl_3$): δ 7.14 (dd, *J* = 8.3, 2.2 Hz, 1H), 6.92 (d, *J* = 2.2 Hz, 1H), 6.84 (d, *J* = 8.3 Hz, 1H), 5.63 (s, 1H), 4.96 (t, *J* = 4.8 Hz, 1H), 4.10 (t, *J* = 4.8 Hz, 1H), 4.01-3.91 (m, 2H), 3.91-3.87 (m, 2H), 3.84-3.77 (m, 5H), 2.86 (d, *J* = 4.8 Hz, 2H), 2.35-2.18 (m, 3H), 2.10-2.02 (m, 1H), 1.26 (t, *J* = 7.0 Hz, 3H); **^{13}C NMR** (100 MHz, $CDCl_3$): δ 200.4, 178.1, 155.9, 129.3, 129.1, 127.7, 127.3, 110.5, 104.7, 104.6, 64.9, 64.3, 55.3, 39.8, 38.9, 33.7, 27.6, 13.9 [One sp^2 carbon is missing due to peak overlapping]; **HRMS** (ESI) *m/z*: $[M+H]^+$ calcd. for $C_{19}H_{25}O_5$ 333.1697, found: 333.1709.

6-Ethoxy-2'-methoxy-5'-(2-((triisopropylsilyl)oxy)ethyl)-2,3-dihydro-[1,1'-biphenyl]-4(1*H*)-one (2u)

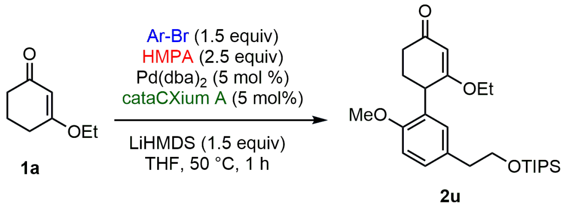

The reaction was conducted with 1.0 mmol of **1a** following the general procedure at (reaction temperature = 50 ºC; reaction time = 1 hour). The crude product was purified by flash column chromatography (EtOAc/hexanes = 1/4) to afford **2u** (252 mg,

56%) as a pale-yellow viscous oil. R_f: 0.25 (EtOAc/hexanes = 1/4). **IR** (film): 2948, 2869, 1659, 1602, 1498 cm^{-1}; ^{1}**H NMR** (400 MHz, $CDCl_3$): δ 7.09 (dd, J = 8.3, 2.3 Hz, 1H), 6.84 (d, J = 2.3 Hz, 1H), 6.81 (d, J = 8.3 Hz, 1H), 5.58 (s, 1H), 4.08 (t, J = 5.0 Hz, 1H), 3.99-3.86 (m, 2H), 3.81-3.76 (m, 5H), 2.75 (t, J = 7.2 Hz, 2H), 2.31-2.16 (m, 3H), 2.10-2.02 (m, 1H), 1.24 (t, J = 7.0 Hz, 3H), 1.04-0.98 (m, 21H); ^{13}**C NMR** (100 MHz, $CDCl_3$): δ 200.3, 178.1, 155.5, 130.9, 128.6, 128.5, 127.4, 110.5, 104.5, 65.0, 64.2, 55.3, 39.1, 38.9, 33.9, 27.8, 17.9, 13.9, 11.8; **HRMS** (ESI) *m/z*: $[M+H]^+$ calcd. for $C_{26}H_{43}O_4Si$ 447.2925, found: 447.2931.

6-Ethoxy-4'-fluoro-2,3-dihydro-[1,1'-biphenyl]-4(1*H*)-one (2v)

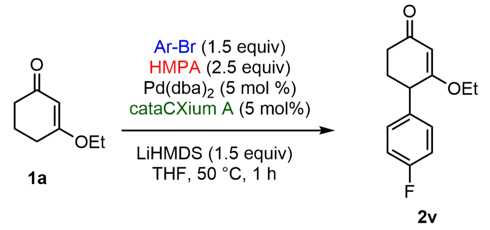

The reaction was conducted with 0.5 mmol of **1a** following the general procedure (reaction temperature = 50 ºC; reaction time = 1 hour). The crude product was purified by flash column chromatography (EtOAc/hexanes/DCM = 1/3/1 to 1/2/1) to afford **2v** (51 mg, 44%) as a pale-yellow viscous oil. R_f: 0.25 (EtOAc/hexanes/DCM = 1/3/1). **IR** (film): 2939, 1651, 1596, 1507 cm^{-1}; ^{1}**H NMR** (400 MHz, $CDCl_3$): δ 7.18-7.12 (m, 2H), 7.05-6.99 (m, 2H), 5.57 (s, 1H), 4.00-3.85 (m, 2H), 3.74 (t, J = 4.9 Hz, 1H), 2.42-2.28 (m, 3H), 2.04-1.95 (m, 1H), 1.27 (t, J = 7.0 Hz, 3H); ^{13}**C NMR** (100 MHz, $CDCl_3$): δ 199.4, 176.9, 161.8 (d, J_{C-F} = 245.3 Hz), 135.4, 129.2 (d, J_{C-F} = 8.0 Hz), 115.4 (d, J_{C-F} = 21.5 Hz), 104.3, 64.5, 43.9, 33.2, 30.2, 13.9; ^{19}**F NMR** (376 MHz, $CDCl_3$): δ -115.7; **HRMS** (ESI) *m/z*: $[M+H]^+$ calcd. for $C_{14}H_{16}FO_2$ 235.1129, found: 235.1128.

6-Ethoxy-4'-(trifluoromethyl)-2,3-dihydro-[1,1'-biphenyl]-4(1*H*)-one (2w)

2-Ethoxy-4'-(trifluoromethyl)-[1,1'-biphenyl]-4-ol (S1)

3-Ethoxyphenol (S2)[2]

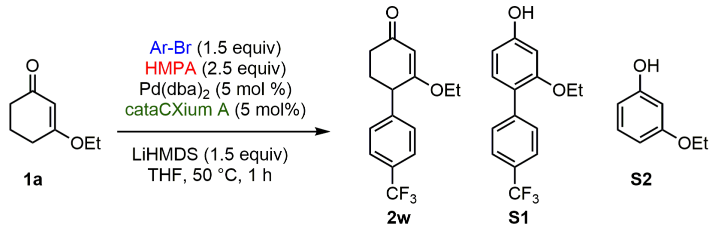

The reaction was conducted with 0.5 mmol of **1a** following the general procedure (reaction temperature = 50 ºC; reaction time = 1 hour). The crude product was purified by flash column chromatography (EtOAc/hexanes/DCM = 1/4/1 to 1/2/1) to afford **2w** (31 mg, 22%), **S1** (12 mg, 9%), **S2** (6 mg, 9%). **2w**: Yellow viscous oil. R*f*: 0.25 (EtOAc/hexanes/DCM = 1/3/1). **IR** (film): 2942, 1655, 1600, 1322 cm^{-1}; **^{1}H NMR** (400 MHz, $CDCl_3$): δ 7.60 (d, *J* = 8.0 Hz, 2H), 7.32 (d, *J* = 8.0 Hz, 2H), 5.61 (s, 1H), 4.01-3.87 (m, 2H), 3.82 (t, *J* = 5.2 Hz, 1H), 2.46-2.36 (m, 1H), 2.33-2.29 (m, 2H), 2.08-1.99 (m, 1H), 1.27 (t, *J* = 6.9 Hz, 3H); **^{13}C NMR** (100 MHz, $CDCl_3$): δ 199.1, 176.2, 144.0, 129.4 (q, *J* = 32.6 Hz), 128.1, 125.6 (q, *J* = 3.8 Hz), 124.1 (q, *J* = 270.7 Hz), 104.7, 64.6, 44.7, 33.3, 30.0, 13.9; **^{19}F NMR** (376 MHz, $CDCl_3$): δ -62.38; **HRMS** (ESI) *m/z*: $[M+H]^+$ calcd. for $C_{15}H_{16}F_3O_2$ 285.1097, found: 285.1101. **S1**: Yellow solid (m.p. 120-121 ºC). R*f*: 0.22 (EtOAc/hexanes = 1/4). **IR** (film): 3365 (broad), 2923, 2855, 1605, 1458 cm^{-1} ; **^{1}H NMR** (400 MHz, $CDCl_3$): δ 7.63 (s, 4H), 7.19 (d, *J* = 8.1 Hz, 1H), 6.52 (d, *J* = 2.3 Hz, 1H), 6.49 (dd, *J* = 8.1, 2.3 Hz, 1H), 4.02 (q, *J* = 7.0 Hz, 2H), 1.36 (t, *J* = 7.0 Hz, 3H); **^{13}C NMR** (100 MHz, $CDCl_3$): δ 157.03, 156.74, 142.08, 131.46, 129.58, 128.25 (q, *J* = 32.2 Hz), 124.73 (q, *J* = 3.8 Hz),124.45 (q, *J* = 270.4 Hz), 122.08, 107.47, 100.41, 64.05, 14.59; **^{19}F NMR** (376 MHz,$CDCl_3$): δ -62.31; **HRMS** (ESI) *m/z*: $[M+H]^+$ calcd. for $C_{15}H_{15}F_3O_2$ calcd. 283.0940, found: 283.0939. **S2**: Pale-red liquid. R*f*: 0.25 (EtOAc/hexanes = 1/4). **^{1}H NMR** (400 MHz, $CDCl_3$): δ 7.12 (t, *J* = 8.4 Hz, 1H), 6.50-6.46 (m, 1H), 6.42-6.39 (m, 2H), 4.00 (q, *J* = 7.0 Hz, 2H), 1.40 (t, *J* = 7.0 Hz,

3H). ^{1}H NMR data of **S2** are consistent with those reported in the literature.[2]

6'-Ethoxy-4'-oxo-1',2',3',4'-tetrahydro-[1,1'-biphenyl]-4-carbonitrile (2x)

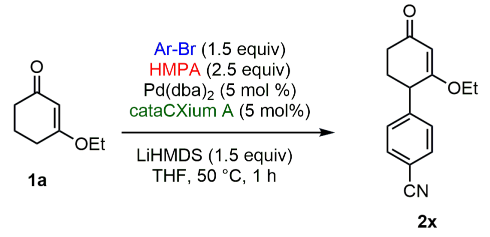

The reaction was conducted with 0.5 mmol of **1a** following the general procedure (reaction temperature = 50 ºC; reaction time = 1 hour). The crude product was purified by flash column chromatography (EtOAc/hexanes/DCM = 1/3/1 to 1/2/1) to afford **2x** (16 mg, 13%) as a pale-yellow viscous oil. R_f: 0.13 (EtOAc/hexanes/DCM = 1/3/1). **IR** (film): 2939, 2229, 1651, 1599 cm^{-1}; **^{1}H NMR** (400 MHz, $CDCl_3$): δ 7.64 (d, *J* = 8.4 Hz, 2H), 7.31 (d, *J* = 8.4 Hz, 2H), 5.60 (s, 1H), 4.00-3.86 (m, 2H), 3.81 (t, *J* = 5.4 Hz, 1H), 2.45-2.23 (m, 3H), 2.07-1.98 (m, 1H), 1.26 (t, *J* = 7.0 Hz, 3H); **^{13}C NMR** (100 MHz, $CDCl_3$): δ 1988, 175.6, 145.5, 132.5, 128.6, 118.6, 111.1, 104.8, 64.7, 44.9, 33.4, 29.9, 13.9; **HRMS** (ESI) *m/z*: $[M+H]^+$ calcd. for $C_{15}H_{16}NO_2$ 242.1176, found: 242.1176.

3-Ethoxy-4-(thiophen-3-yl)cyclohex-2-en-1-one (2y)

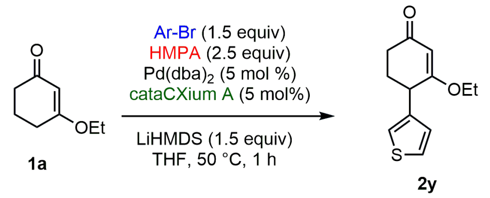

The reaction was conducted with 0.5 mmol of **1a** following the general procedure (reaction temperature = 50 ºC; reaction time = 1 hour). The crude product was purified by flash column chromatography (EtOAc/hexanes/DCM = 1/3/1 to 1/2/1) to afford **2y** (30 mg, 27%) as a brown viscous oil. R_f: 0.28 (EtOAc/hexanes/DCM = 1/3/1). **IR** (film): 2937, 1645, 1595, 1342 cm^{-1}; **^{1}H NMR** (400 MHz, $CDCl_3$): δ 7.31 (dd, *J* = 5.0, 3.0 Hz,

1H), 7.02-7.00 (m, 1H), 6.98 (dd, J = 5.0, 1.4 Hz, 1H), 5.49 (s, 1H), 4.02-3.87 (m, 2H), 3.82 (t, J = 4.1 Hz, 1H), 2.39-2.27 (m, 3H), 2.16-2.07 (m, 1H), 1.32 (t, J = 7.0 Hz, 3H); **^{13}C NMR** (100 MHz, $CDCl_3$): δ 199.6, 177.6, 139.7, 127.3, 125.9, 121.3, 103.3, 64.5, 40.1, 33.3, 29.1, 13.9; **HRMS** (ESI) *m/z*: $[M+H]^+$ calcd. for $C_{12}H_{15}O_2S$ calcd. 223.0787, found: 223.0785.

3-Ethoxy-4-(pyridin-2-yl)cyclohex-2-en-1-one (2z)

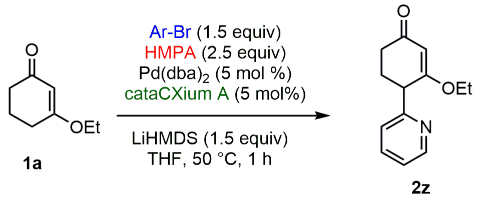

The reaction was conducted with 0.5 mmol of **1a** following the general procedure (reaction temperature = 50 ºC; reaction time = 1 hour). The crude product was purified by flash column chromatography (EtOAc/hexanes = 2/1) to afford **2z** (41 mg, 38%) as a pale-yellow viscous oil. R$_f$: 0.25 (EtOAc/hexanes = 2/1). **IR** (film): 3444 (broad), 2990, 2925, 1651 cm^{-1}; **^{1}H NMR** (400 MHz, $CDCl_3$):) δ 8.58 (ddd, J = 4.9, 1.9, 1.0 Hz, 1H), 7.64 (td, J = 7.7, 1.9 Hz, 1H), 7.19 (ddd, J = 7.6, 4.9, 1.1 Hz, 1H), 7.16 (dt, J = 7.8, 1.1 Hz, 1H), 5.57 (s, 1H), 4.00-3.87 (m, 3H), 2.44-2.28 (m, 4H), 1.26 (t, J = 7.0 Hz, 3H); **^{13}C NMR** (100 MHz, $CDCl_3$): δ 199.6, 176.5, 159.6, 149.6, 136.6, 122.2, 122.0, 104.4, 64.5, 47.0, 33.9, 28.5, 13.9; **HRMS** (ESI) *m/z*: $[M+H]^+$ calcd. for $C_{13}H_{16}NO_2$ 218.1176, found: 218.1172.

3-Ethoxy-4-(pyridin-3-yl)cyclohex-2-en-1-one (2aa)

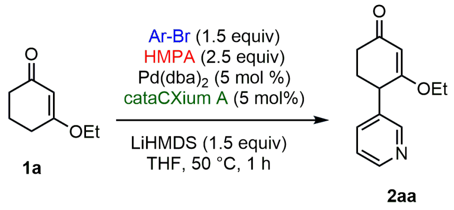

The reaction was conducted with 0.5 mmol of **1a** following the general procedure (reaction temperature = 50 ºC; reaction time = 1 hour). The crude product was purified by flash column chromatography (Acetone/hexanes = 2/1) to afford **2aa** (38 mg, 35%) as a pale-yellow viscous oil. R_f: 0.3 (acetone/hexanes = 2/1). **IR** (film): 3453 (broad), 2939, 2985, 2945, 1647 cm^{-1}; **^{1}H NMR** (400 MHz, $CDCl_3$): δ 8.52 (dd, J = 4.9, 1.7 Hz, 1H), 8.49 (br s, 1H), 7.51-7.47 (m, 1H), 7.30-7.25 (m, 1H), 5.59 (s, 1H), 4.00-3.85 (m, 2H), 3.79 (t, J = 5.3 Hz, 1H), 2.45-2.30 (m, 3H), 2.09-2.01 (m, 1H), 1.25 (t, J = 7.0 Hz, 3H); **^{13}C NMR** (100 MHz, $CDCl_3$): δ 198.9, 175.9, 149.5, 148.4, 135.6, 134.9, 123.5, 104.6, 64.7, 42.5, 33.5, 29.9, 13.9; **HRMS** (ESI) *m/z*: $[M+H]^+$ calcd. for $C_{13}H_{16}NO_2$ 218.1176, found: 218.1176.

tert-Butyl 3-(2-ethoxy-4-oxocyclohex-2-en-1-yl)-9*H*-carbazole-9-carboxylate (2bb)

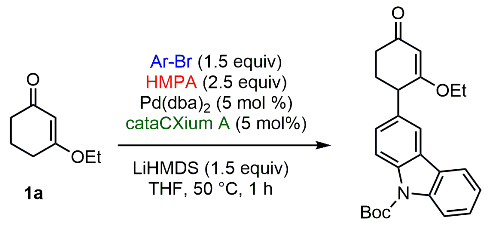

The reaction was conducted with 0.5 mmol of **1a** following the general procedure (reaction temperature = 50 ºC; reaction time = 1 hour). The crude product was purified by flash column chromatography (EtOAc/hexanes/DCM = 1/3/1) to afford **2bb** (97 mg, 48%) as a yellow viscous oil. R_f: 0.3 (EtOAc/hexanes/DCM = 1/3/1). **IR** (film): 2982, 1724, 1652, 1600 cm^{-1}; **^{1}H NMR** (400 MHz, $CDCl_3$):) δ 8.30 (d, J = 8.4 Hz, 1H), 8.26 (d, J = 8.6 Hz, 1H), 7.97-7.93 (m, 1H), 7.79 (d, J = 1.8 Hz, 1H), 7.47 (ddd, J = 8.4, 7.4, 1.3 Hz, 1H), 7.35 (td, J = 7.4, 1.0 Hz, 1H), 7.30 (dd, J = 8.6, 1.8 Hz, 1H), 5.66 (s, 1H), 4.02-3.93 (m, 3H), 2.51-2.28 (m, 3H), 2.18-2.10 (m, 1H), 1.76 (s, 9H), 1.27 (t, J = 7.0 Hz, 3H); **^{13}C NMR** (100 MHz, $CDCl_3$): δ 199.8, 177.5, 150.9, 138.8, 137.5, 134.5,

127.3, 126.7, 126.1, 125.4, 123.0, 119.6, 118.5, 116.4, 116.3, 104.4, 83.9, 64.5, 44.7, 33.3, 30.6, 28.3, 13.9; **HRMS** (ESI) *m/z*: $[M+H]^+$ calcd. for $C_{25}H_{28}NO_4$ 406.2013, found: 406.2016.

6-Isopropoxy-4'-methoxy-2,3-dihydro-[1,1'-biphenyl]-4(1*H*)-one (2cc)

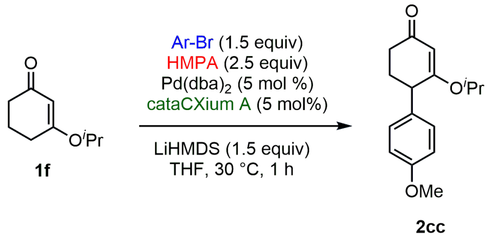

The reaction was conducted with 0.5 mmol of **1f**[3] following the general procedure (reaction temperature = 30 ºC; reaction time = 1 hour). The crude product was purified by flash column chromatography (EtOAc/hexanes/DCM = 1/3/1) to afford **2cc** (113 mg, 87%) as a pale-yellow viscous oil. R_f: 0.23 (EtOAc/hexanes/DCM = 1/3/1). **IR** (film): 2979, 1650, 1593, 1509 cm^{-1}; ^{1}**H NMR** (400 MHz, $CDCl_3$): δ 7.10 (d, *J* = 8.8 Hz, 2H), 6.86 (d, *J* = 8.8 Hz, 2H), 5.56 (s, 1H), 4.47 (septet, *J* = 6.1 Hz, 1H), 3.80 (s, 3H), 3.67 (t, *J* = 4.4 Hz, 1H), 2.38-2.21 (m, 3H), 2.01-1.95 (m, 1H), 1.27 (d, *J* = 6.1 Hz, 3H), 1.19 (d, *J* = 6.1 Hz, 3H); ^{13}**C NMR** (100 MHz, $CDCl_3$): δ 199.9, 176.6, 158.4, 131.7, 128.6, 113.9, 104.5, 71.0, 55.2, 44.1, 32.9, 30.2, 21.6, 20.8; **HRMS** (ESI) *m/z*: $[M+H]^+$ calcd. for $C_{16}H_{21}O_3$ 261.1485, found: 261.1482.

4'-Methoxy-6-propoxy-2,3-dihydro-[1,1'-biphenyl]-4(1*H*)-one (2dd)

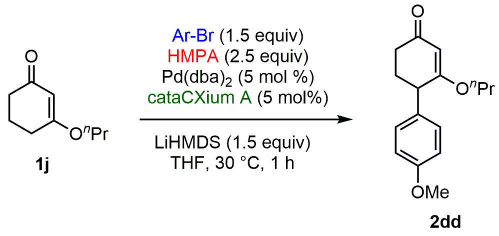

The reaction was conducted with 0.5 mmol of **1j**[4] following the general procedure (reaction temperature = 30 ºC; reaction time = 1 hour). The crude product was purified

by flash column chromatography (EtOAc/hexanes/DCM = 1/3/1) to afford **2dd** (100 mg, 77%) as a white solid (m.p. 70-71 ºC). R*f*: 0.2 (EtOAc/hexanes/DCM = 1/3/1). **IR** (cast): 2965, 2875, 1649, 1596 cm^{-1}; **^{1}H NMR** (400 MHz, $CDCl_3$): δ 7.11 (d, *J* = 8.7 Hz, 2H), 6.86 (d, *J* = 8.7 Hz, 2H), 5.55 (s, 1H), 3.87-3.80 (m, 1H), 3.81-3.74 (m, 4H), 3.73-3.70 (m, 1H), 2.39-2.24 (m, 3H), 2.05-1.98 (m, 1H), 1.70-1.61 (m, 2H), 0.85 (t, *J* = 7.4 Hz, 3H); **^{13}C NMR** (100 MHz, $CDCl_3$): δ 199.7, 177.8, 158.5, 131.7, 128.7, 113.9, 104.1, 70.1, 55.8, 43.9, 33.4, 30.2, 21.7, 10.3; **HRMS** (ESI) *m/z*: [M+H]$^+$ calcd. for $C_{16}H_{21}O_3$ 261.1485, found: 261.1480.

6-(Cyclohexyloxy)-4'-methoxy-2,3-dihydro-[1,1'-biphenyl]-4(1*H*)-one (2ee)

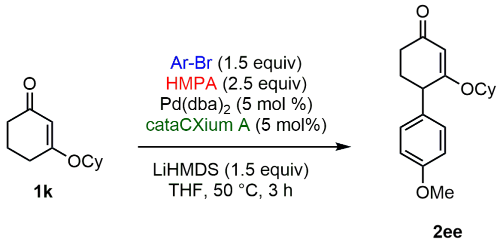

The reaction was conducted with 0.5 mmol of **1k**[5] following the general procedure (reaction temperature = 50 ºC; reaction time = 3 hours). The crude product was purified by flash column chromatography (EtOAc/hexanes/DCM = 1/4/1 to 1/3/1) to afford **2ee** (50 mg, 33%) as a colorless viscous oil. R*f*: 0.75 (EtOAc/DCM = 1/3). **IR** (film): 2934, 2863, 1648, 1595 cm^{-1}; **^{1}H NMR** (400 MHz, $CDCl_3$): δ 7.11 (d, *J* = 8.7 Hz, 2H), 6.86 (d, *J* = 8.7 Hz, 2H), 5.57 (s, 1H), 4.23-4.16 (m, 1H), 3.80 (s, 3H), 3.68 (t, *J* = 4.5 Hz, 1H), 2.40-2.21 (m, 3H), 2.03-1.96 (m, 1H), 1.91-1.81 (m, 2H), 1.71-1.63 (m, 1H), 1.61-1.58 (m, 1H), 1.53-1.40 (m, 2H), 1.38-1.16 (m, 4H); **^{13}C NMR** (100 MHz, $CDCl_3$): δ 199.9, 176.6, 158.4, 131.9, 128.7, 113.9, 104.4, 76.2, 55.2, 44.1, 33.1, 31.2, 30.3, 30.1, 25.2, 23.0, 23.3; **HRMS** (ESI) *m/z*: [M+H]$^+$ calcd. for $C_{19}H_{25}O_3$ 301.1798, found: 301.1794.

4'-Methoxy-6-(methyl(phenyl)amino)-2,3-dihydro-[1,1'-biphenyl]-4(1*H*)-one (2ff)

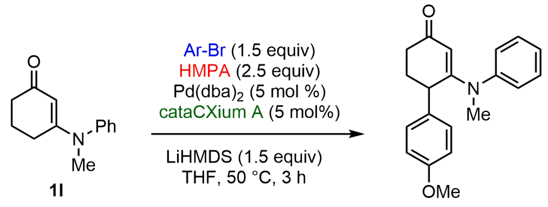

The reaction was conducted with 0.5 mmol of **1l**[6] following the general procedure (reaction temperature = 50 ºC; reaction time = 3 hours). The crude product was purified by flash column chromatography (EtOAc/hexanes = 3/1 to 10/1) to afford **2ff** (130 mg, 85%) as a yellow viscous oil. R_f: 0.23 (EtOAc/hexanes = 3/1). **IR** (film): 2942, 1609, 1545, 1507 cm^{-1}; **^{1}H NMR** (400 MHz, $CDCl_3$): δ 7.25-7.22 (m, 3H), 6.98 (d, *J* = 8.7 Hz, 2H), 6.87-6.82 (m, 2H), 6.80 (d, *J* = 8.7 Hz, 2H), 5.45 (s, 1H), 3.80 (s, 3H), 3.72-3.69 (m, 1H), 3.18 (s, 3H), 2.39-2.29 (m, 1H), 2.21-2.14 (m, 2H), 1.90-1.84 (m, 1H); **^{13}C NMR** (100 MHz, $CDCl_3$): δ 197.7, 165.5, 158.5, 144.9, 131.2, 129.3, 128.8, 127.5, 127.4, 113.9, 101.9, 55.2, 41.2, 40.8, 31.2, 30.7; **HRMS** (ESI) *m/z*: $[M+H]^+$ calcd. for $C_{20}H_{22}NO_2$ 308.1645, found: 308.1645.

3-Ethoxy-4-(4-methoxyphenyl)cyclohept-2-en-1-one (2gg)

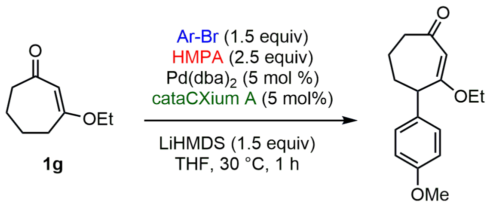

The reaction was conducted with 0.5 mmol of **1g**[7] following the general procedure (reaction temperature = 30 ºC; reaction time = 1 hour). The crude product was purified by flash column chromatography (EtOAc/hexanes/DCM = 1/3/1) to afford **2gg** (99 mg, 76%) as a white solid (m.p. 49-50 ºC). R_f: 0.33 (EtOAc/hexanes/DCM = 1/3/1). **IR** (cast): 2937, 2877, 1602, 1508 cm^{-1}; **^{1}H NMR** (400 MHz, $CDCl_3$): δ 7.10 (d, *J* = 8.6 Hz, 2H), 6.85 (d, *J* = 8.6 Hz, 2H), 5.60 (s, 1H), 3.92 (dd, *J* = 7.0, 4.1 Hz, 1H), 3.87-

3.76 (m, 5H), 2.64-2.46 (m, 2H), 2.22-2.07 (m, 2H), 1.74-1.67 (m, 2H), 1.23 (t, J = 7.0 Hz, 3H); **^{13}C NMR** (100 MHz, $CDCl_3$): δ 201.9, 174.6, 158.1, 132.7, 128.5, 113.8, 107.6, 64.1, 55.2, 49.8, 43.1, 32.2, 18.9, 14.0; **HRMS** (ESI) *m/z*: $[M+H]^+$ calcd. for $C_{16}H_{21}O_3$ 261.1485, found: 261.1483.

4-(4-Methoxyphenyl)-3,4,5,6,7,9-hexahydro-1H-xanthene-1,8(2*H*)-dione (2hh)

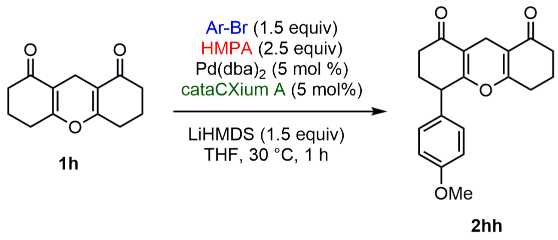

The reaction was conducted with 0.5 mmol of **1h**[8] following the general procedure (reaction temperature = 30 ºC; reaction time = 1 hour). The crude product was purified by flash column chromatography (EtOAc/hexanes/DCM = 1/2/1) to afford **2hh** (66 mg, 41%) as a yellow viscous oil. R_f: 0.23 (EtOAc/hexanes/DCM = 1/2/1). **IR** (film): 2951, 2838, 1657, 1510 cm^{-1}; **^{1}H NMR** (400 MHz, $CDCl_3$): δ 7.14 (d, J = 8.6 Hz, 2H), 6.89 (d, J = 8.6 Hz, 2H), 3.80 (s, 3H), 3.79-3.75 (m, 1H), 3.07-2.90 (m, 2H), 2.47-2.30 (m, 7H), 2.03-1.94 (m, 3H); **^{13}C NMR** (100 MHz, $CDCl_3$): δ 197.6, 197.6, 164.8, 164.4, 158.8, 130.8, 128.7, 114.2, 113.9, 112.2, 55.3, 42.2, 36.6, 33.3, 29.7, 26.8, 20.3, 15.9; **HRMS** (ESI) *m/z*: $[M+H]^+$ calcd. for $C_{20}H_{21}O_4$ 325.1434, found: 325.1438.

6-Ethoxy-4'-methoxy-5-methyl-2,3-dihydro-[1,1'-biphenyl]-4(1*H*)-one (2ii)

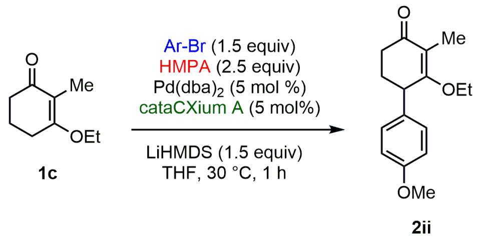

The reaction was conducted with 0.5 mmol of **1c**[8] following the general procedure (reaction temperature = 30 ºC; reaction time = 1 hour). The crude product was purified

by flash column chromatography (EtOAc/hexanes = 1/3) to afford **2ii** (61 mg, 47%) as a pale-yellow viscous oil. R$_f$: 0.25 (EtOAc/hexanes = 1/3). **IR** (film): 2982, 2931, 1643, 1607 cm^{-1}; **^{1}H NMR** (400 MHz, $CDCl_3$): δ 7.14 (d, *J* = 8.7 Hz, 2H), 6.88 (d, *J* = 8.7 Hz, 2H), 4.03-3.93 (m, 2H), 3.80 (s, 3H), 3.76-3.57 (m, 1H), 2.42-2.33 (m, 1H), 2.30-2.18 (m, 2H), 1.98-1.92 (m, 1H), 1.83 (s, 3H), 1.20 (t, *J* = 7.0 Hz, 3H); **^{13}C NMR** (100 MHz, $CDCl_3$): δ 198.9, 171.1, 158.7, 130.2, 128.8, 116.5, 114.3, 63.4, 55.2, 39.9, 31.8, 30.2, 15.1, 7.6; **HRMS** (ESI) *m/z*: $[M+H]^+$ calcd. for $C_{16}H_{21}O_3$ 261.1485, found: 261.1483.

(1S,3R)-6-Ethoxy-4'-methoxy-3-methyl-2,3-dihydro-[1,1'-biphenyl]-4(1*H*)-one (2jj) and (1R,3R)-6-Ethoxy-4'-methoxy-3-methyl-2,3-dihydro-[1,1'-biphenyl]-4(1*H*)-one (2jj')

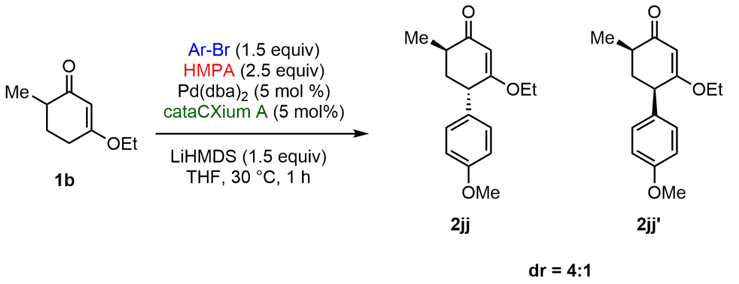

The reaction was conducted with 0.5 mmol of **1b**[9] following the general procedure (reaction temperature = 30 ºC; reaction time = 1 hour). The crude product was purified by flash column chromatography (EtOAc/hexanes = 1/3) to afford **2jj** (40 mg) and a mixture of **2jj** and **2jj'** (31mg). The combined yield of the two diastereomers is 54%. Subsequently, the mixture of isomers was further purified by flash column chromatography (EtOAc/hexanes = 1/3) to obtain a small amount of pure **2jj'** (7 mg). **2jj**: Pale-yellow viscous oil. R$_f$: 0.53 (EtOAc/hexanes/DCM = 1/1/1). **IR** (film): 2928, 1654, 1605, 1508 cm^{-1}; **^{1}H NMR** (400 MHz, $CDCl_3$): δ 7.12 (d, *J* = 8.7 Hz, 2H), 6.86 (d, *J* = 8.7 Hz, 2H), 5.56 (s, 1H), 4.01-3.85 (m, 2H), 3.80 (s, 3H), 3.72 (dd, *J* = 5.4, 2.9 Hz, 1H), 2.31 (dqd, *J* = 13.4, 6.8, 4.6 Hz, 1H), 2.18-2.09 (m, 1H), 2.00 (ddd, *J* = 13.1,

4.6, 2.9 Hz, 1H), 1.28 (t, J = 7.1 Hz, 3H), 1.07 (d, J = 6.8 Hz, 3H); **^{13}C NMR** (100 MHz, $CDCl_3$): δ 201.9, 176.5, 158.5, 131.7, 128.6, 113.9, 103.9, 64.4, 55.2, 43.6, 38.5, 35.3, 15.0, 14.0; **HRMS** (ESI) *m/z*: $[M+H]^+$ calcd. for $C_{16}H_{21}O_3$ 261.1485, found: 261.1478.

2jj': Colorless viscous oil. R_f: 0.51 (EtOAc/hexanes/DCM = 1/1/1). **IR** (film): 2931, 1656, 1597, 1512 cm^{-1}; **^{1}H NMR** (400 MHz, $CDCl_3$): δ 7.06 (d, J = 8.7 Hz, 2H), 6.85 (d, J = 8.7 Hz, 2H), 5.50 (d, J = 1.7 Hz, 1H), 3.86 (q, J = 7.0 Hz, 2H), 3.80 (s, 3H), 3.77 (ddd, J = 11.9, 4.9, 1.7 Hz, 1H), 2.44 (dqd, J = 13.4, 6.7, 4.5 Hz, 1H), 2.23-2.16 (m, 1H), 1.85-1.75 (m, 1H), 1.18 (t, J = 7.0 Hz, 3H), 1.14 (d, J = 6.7 Hz, 3H); **^{13}C NMR** (100 MHz, $CDCl_3$): δ 201.3, 177.2, 158.3, 133.3, 128.9, 113.8, 103.4, 64.4, 55.2, 46.1, 40.6, 40.4, 14.8, 13.8; **HRMS** (ESI) *m/z*: $[M+H]^+$ calcd. for $C_{16}H_{21}O_3$ 261.1485, found: 261.1482.

(1S,2S)-6-Ethoxy-4'-methoxy-2-methyl-2,3-dihydro-[1,1'-biphenyl]-4(1*H*)-one (2kk)

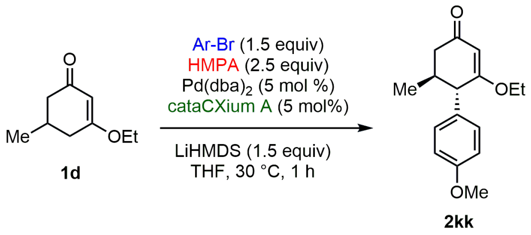

The reaction was conducted with 0.5 mmol of **1d**[9] following the general procedure (reaction temperature = 30 ºC; reaction time = 1 hour). The crude product was purified by flash column chromatography (EtOAc/hexanes/DCM = 2/3/1) to afford **2kk** (122 mg, 93%) as a pale-yellow viscous oil. R_f: 0.33 (EtOAc/hexanes/DCM = 2/3/1). **IR** (film): 2959, 2835, 1651, 1596 cm^{-1}; **^{1}H NMR** (400 MHz, $CDCl_3$): δ 7.06 (d, J = 8.7 Hz, 2H), 6.85 (d, J = 8.7 Hz, 2H), 5.53 (s, 1H), 3.95-3.83 (m, 2H), 3.80 (s, 3H), 3.34 (d, J = 6.3 Hz, 1H), 2.49 (dd, J = 16.3, 4.4 Hz, 1H), 2.30-2.19 (m, 1H), 2.13 (dd, J = 16.3, 7.9 Hz, 1H), 1.21 (t, J = 7.0 Hz, 3H), 1.04 (d, J = 6.7 Hz, 3H); **^{13}C NMR** (100

MHz, $CDCl_3$): δ 199.03 176.7, 158.4, 131.9, 128.9, 113.8, 103.4, 64.4, 55.1, 52.1, 42.1, 36.4, 19.9, 13.8; **HRMS** (ESI) *m/z*: $[M+H]^+$ calcd. for $C_{16}H_{21}O_3$ 261.1485, found: 261.1491.

6-Ethoxy-4'-methoxy-2,2-dimethyl-2,3-dihydro-[1,1'-biphenyl]-4(1*H*)-one (2ll)

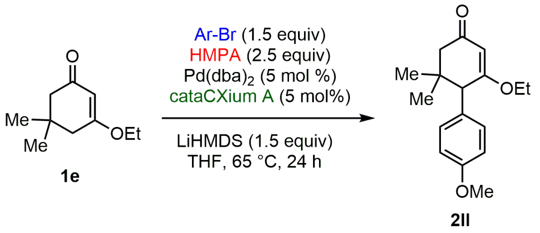

The reaction was conducted with 0.5 mmol of **1e**[3] following the general procedure (reaction temperature = 65 ºC; reaction time = 24 hours). The crude product was purified by flash column chromatography (EtOAc/hexanes/DCM = 1/1/7) to afford **2ll** (21 mg, 15%) as a pale-yellow viscous oil. R_f: 0.43 (EtOAc/hexanes/DCM = 1/1/7). **IR** (film): 2968, 2908, 1653, 1601 cm^{-1}; ^{1}**H NMR** (400 MHz, $CDCl_3$): δ 7.08 (d, J = 8.6 Hz, 2H), 6.84 (d, J = 8.6 Hz, 2H), 5.56 (s, 1H), 4.00-3.91 (m, 1H), 3.87-3.77 (m, 4H), 3.25 (s, 1H), 2.38 (d, J = 17.1 Hz, 1H), 2.03 (d, J = 17.1 Hz, 1H), 1.23 (t, J = 7.0 Hz, 3H), 1.20 (s, 3H), 0.72 (s, 3H); ^{13}**C NMR** (100 MHz, $CDCl_3$): δ 199.7, 177.6, 158.8, 129.8, 129.2, 113.7, 102.7, 64.5, 56.3, 55.2, 46.7, 35.6, 29.2, 27.6, 13.9; **HRMS** (ESI) *m/z*: $[M+H]^+$ calcd. for $C_{17}H_{23}O_3$ 275.1642 found: 275.1640.

6-Ethoxy-4'-methoxy-3,3-dimethyl-2,3-dihydro-[1,1'-biphenyl]-4(1*H*)-one (2mm)

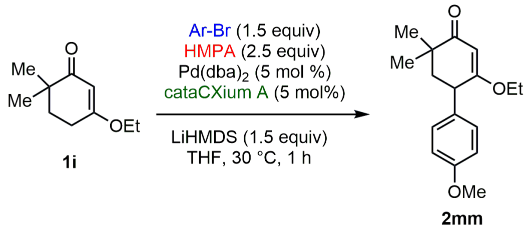

The reaction was conducted with 0.5 mmol of **1i**[10] following the general procedure (reaction temperature = 30 ºC; reaction time = 1 hour). The crude product was purified

by flash column chromatography (EtOAc/hexanes = 1/5) to afford **2mm** (58 mg, 42%) as a pale-yellow viscous oil. R*f*: 0.33 (EtOAc/hexanes = 1/5). **IR** (film): 2962, 2925, 1651, 1597 cm^{-1}; **^{1}H NMR** (400 MHz, $CDCl_3$): δ 7.07 (d, *J* = 8.7 Hz, 2H), 6.86 (d, *J* = 8.7 Hz, 2H), 5.41 (d, *J* = 1.5 Hz, 1H), 3.88 (q, *J* = 7.0 Hz, 2H), 3.81-3.76 (m, 4H), 1.97-1.94 (m, 2H), 1.21-1.17 (m, 6H), 1.10 (s, 3H); **^{13}C NMR** (100 MHz, $CDCl_3$): δ 204.4, 175.9, 158.2, 133.6, 129.0, 113.8, 102.1, 64.3, 55.2, 45.9, 42.8, 41.1, 24.9, 24.6, 13.8; **HRMS** (ESI) *m/z*: $[M+H]^+$ calcd. for $C_{17}H_{23}O_3$ 275.1642, found: 275.1640.

6-Ethoxy-2'-methoxy-3,3-dimethyl-2,3-dihydro-[1,1'-biphenyl]-4(1*H*)-one (2nn)

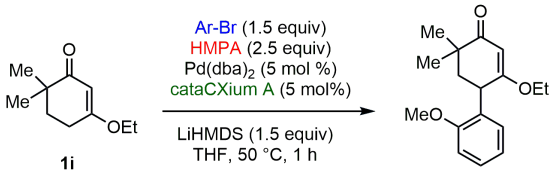

The reaction was conducted with 0.5 mmol of **1i**[10] following the general procedure (reaction temperature = 50 ºC; reaction time = 1 hour). The crude product was purified by flash column chromatography (EtOAc/hexanes/DCM = 1/6) to afford **2nn** (43 mg, 31%) as a colorless, viscous oil. R*f*: 0.23 (EtOAc/hexanes = 1/5). **IR** (film): 2962, 2928, 1650 1597 cm^{-1}; **^{1}H NMR** (400 MHz, $CDCl_3$): δ 7.22 (ddd, *J* = 8.2, 7.3, 1.8 Hz, 1H), 7.08-6.98 (broad, 1H), 6.91 (td, *J* = 7.3, 1.1 Hz, 1H), 6.87 (d, *J* = 8.2 Hz, 1H), 5.40 (s, 1H), 4.70-4.00 (broad, 1H), 3.91-3.84 (m, 2H), 3.81 (s, 3H), 2.30-1.78 (broad, 2H), 1.22 (s, 3H), 1.18 (t, *J* = 7.0 Hz, 3H), 1.10 (s, 3H); **^{13}C NMR** (100 MHz, $CDCl_3$): δ 204.7, 176.8, 157.1, 129.7, 127.8, 120.6, 110.7, 102.0, 64.2, 55.3, 43.6, 41.0, 25.1, 24.7, 13.8 [One sp^2 and one sp^3 carbons are missing due to peak broadening or overlapping]; **HRMS** (ESI) *m/z*: $[M+H]^+$ calcd. for $C_{17}H_{23}O_3$ 275.1642, found: 275.1642. Note: The ^{1}H and ^{13}C NMR spectra of **2nn** shows line broadening at room temperature, likely due to restricted rotation about the aryl–C bond caused by the *ortho*-OMe aryl substituent

and the CVE core carrying the α-gem-dimethyl group. At –45 °C, two sets of ^{1}H and ^{13}C NMR resonances are observed, consistent with two slowly interconverting diastereomeric conformers. Accordingly, the ^{1}H and ^{13}C NMR spectra of **2nn** acquired at –45 °C are provided below for reference.

6'-Ethoxy-2',3'-dihydro-4'*H*-[1,1':1',1''-terphenyl]-4'-one (3a)

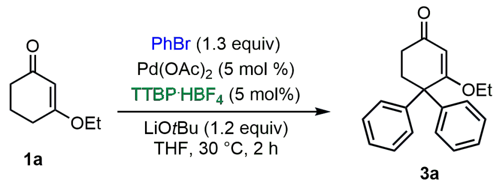

To a round-bottom flask containing vinylogous ester **1a** (1 mmol), bromobenzene (1.2 equiv., 188 mg), $Pd(OAc)_2$ (5 mol %, 11 mg), and TTBP·HBF_4 (5 mol %, 15 mg), was added DMSO (2 mL) at rt. After stirring for 10 minutes, a solution of LiO^{t}Bu (0.66 M in THF, 1.2 equiv., 1.8 mL) was slowly added into the flask, and the mixture was placed in an aluminum heating block (70 ºC). After stirring for 5 hours at 70 ºC, the reaction was quenched with water, and the mixture was diluted with ethyl acetate and extracted with brine. The organic layer was filtered through a pad of celite, dried over anhydrous $MgSO_{4(s)}$, and concentrated with a rotary evaporator. The crude residue was purified by flash column chromatography (EtOAc/hexanes/DCM = 1/5/1 to 1/3/1) to afford **3a** (11 mg, 4%) as a yellow solid (m.p. 133-134 ºC) and **2a** (30 mg, 14%). R_f : 0.28 (EtOAc/hexanes /DCM = 1/3/1). **IR** (cast): 3064, 2985, 1654, 1591 cm^{-1}; **^{1}H NMR** (400 MHz, $CDCl_3$): δ 7.33-7.27 (m, 6H), 7.20-7.17 (m, 4H), 5.61 (s, 1H), 3.96 (q, *J* = 7.0 Hz, 2H), 2.70 (t, *J* = 6.3 Hz, 2H), 2.27 (t, *J* = 6.3 Hz, 2H), 1.20 (t, *J* = 7.0 Hz, 3H); **^{13}C NMR** (100 MHz, $CDCl_3$): δ 199.1, 179.1, 142.9, 128.6, 127.9, 126.9, 104.8, 64.7, 54.1, 36.8, 34.4, 13.8; **HRMS** (ESI) *m/z*: $[M+H]^+$ calcd. for $C_{20}H_{21}O_2$ 293.1536, found: 293.1536.

4-Ethoxy-5,6-dihydro-[1,1'-biphenyl]-2(1H)-one (4a)

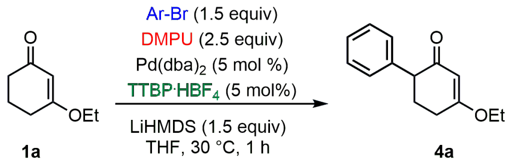

To a round-bottom flask containing **1a** (70 mg, 0.5 mmol), DMPU (2.5 equiv., 1.25 mmol, 0.2 mL) was added THF (2.5 mL) at room temperature, and the solution was cooled to 0 ºC (an ice bath). A solution of LiHMDS (0.62 M, 1.5 equiv., 1.2 mL) was slowly added to the mixture at 0 °C and then stirred for 1 hour. At the same time, another round-bottom flask containing aryl bromide (1.3 equiv.), $Pd(dba)_2$ (5 mol%, 14 mg), TTBP • HBF_4 (5 mol%, 7 mg) was evacuated and backfilled with N_2 3 times. After adding THF (1.5 mL), the mixture was stirred at room temperature for 30 minutes. Subsequently, the solution of enolate was transferred to the flask containing the catalyst, and it was heated to 30 ºC. After stirring for 1 hour, the reaction was quenched with water, and the mixture was diluted with ethyl acetate and extracted with brine. The organic layer was filtered through a pad of celite, dried over anhydrous $MgSO_{4(s)}$, and concentrated with a rotary evaporator. The crude residue was purified by flash column chromatography (EtOAc/hexanes = 1/3) to afford **4a** (14 mg, 13%) as a yellow solid (m.p. 53-54 ºC) and **2a** (29 mg, 28%). R_f : 0.2 (EtOAc/hexanes = 1/3). **IR** (film): 3058, 3030, 2981, 2939, 1651, 1600, 1496 cm^{-1}; **^{1}H NMR** (400 MHz, $CDCl_3$): δ 7.36-7.29 (m, 2H), 7.27-7.21 (m, 1H), 7.20-7.15 (m, 2H), 5.51 (s, 1H), 3.95 (qd, J = 7.0, 1.7 Hz, 2H), 3.53 (dd, J = 9.6, 5.3 Hz, 1H), 2.61-2.40 (m, 2H), 2.33-2.18 (m, 2H), 1.39 (t, J = 7.0 Hz, 3H); **^{13}C NMR** (100 MHz, $CDCl_3$): δ 199.2, 177.3, 139.9, 128.5, 128.3, 126.8, 103.2, 64.4, 51.9, 29.4, 28.2, 14.2; **HRMS** (EI) *m/z*: $[M]^+$ calcd. for $C_{14}H_{17}O_2$ 216.1145, found: 216.1147.

(1'S,3'S)-6'-Ethoxy-4''-methoxy-2',3'-dihydro-[1,1':3',1''-terphenyl]-4'(1'*H*)-one (5a-anti) and (1'R,3'S)-6'-Ethoxy-4''-methoxy-2',3'-dihydro-[1,1':3',1''-terphenyl]-4'(1'*H*)-one (5a-syn)

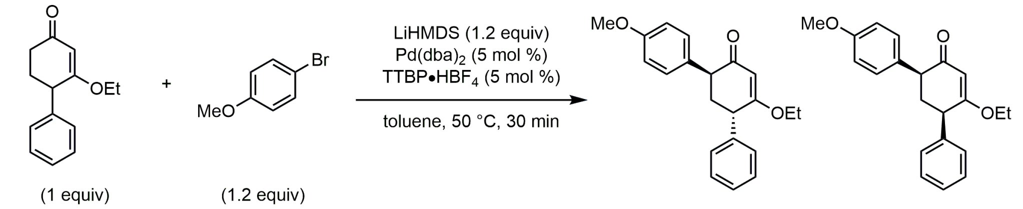

(1 equiv) (1.2 equiv)

2a **5a-anti** **5a-syn**

To a round-bottom flask containing **2a** (50 mg, 0.23 mmol) was sequentially added 4-bormoanisole (1.2 equiv., 52 mg), Pd(dba)$_2$ (5 mol%, 7 mg) and TTBP·HBF4 (5 mol%, 3 mg), and toluene (2 mL). After stirring for 10 minutes, LiHMDS (0.62 M, 1.2 equiv., 0.45 mL) was slowly added into the flask at room temperature, and the mixture was moved to an oil bath of 50 ºC. After stirring for 30 minutes at 50 ºC, the reaction was quenched with water and diluted with ethyl acetate. The organic layer was separated, washed with brine, dried over anhydrous $MgSO_{4(s)}$, filtered through a pad of celite, and concentrated with a rotary evaporator. The crude residue was purified by flash column chromatography (EtOAc/hexanes = 1/3) to afford **5a**-anti (45 mg, 60%) and **5a**-syn (10 mg, 14%). **5a-**anti: Yellow solid (m.p. 128-129 ºC). R$_f$: 0.3 (EtOAc/hexanes = 1/3). **IR** (cast): 2993, 2934, 1654, 1607 cm^{-1}; **^{1}H NMR** (400 MHz, $CDCl_3$): δ 7.39-7.34 (m, 2H), 7.32-7.28 (m, 1H), 7.25-7.23 (m, 2H), 7.01 (d, *J* = 8.7 Hz, 2H), 6.83 (d, *J* = 8.7 Hz, 2H), 5.74 (s, 1H), 4.07-3.92 (m, 2H), 3.86 (dd, *J* = 5.3, 3.5 Hz, 1H), 3.77 (s, 3H), 3.44 (dd, *J* = 12.2, 4.5 Hz, 1H), 2.67 (ddd, *J* = 13.3, 12.2, 5.3 Hz, 1H), 2.27 (ddd, *J* = 13.3, 4.5, 3.5 Hz, 1H), 1.32 (t, *J* = 7.0 Hz, 3H); **^{13}C NMR** (100 MHz, $CDCl_3$): δ 199.4, 176.4, 158.4, 139.4, 131.8, 129.5, 128.7, 127.7, 127.1, 113.9, 104.7, 64.6, 55.2, 47.1, 44.4, 38.4, 14.0; **HRMS** (ESI) *m/z*: $[M+H]^+$ calcd. for $C_{21}H_{23}O_3$ 323.1642, found: 323.1642. **5a**-syn: Colorless viscous oil. R$_f$: 0.28 (EtOAc/hexanes = 1/3); **IR** (film): 2979, 2920, 1654, 1596 cm^{-1}; **^{1}H NMR** (400 MHz, $CDCl_3$): δ 7.35-7.30

(m, 2H), 7.28-7.25 (m, 1H), 7.19 (d, *J* = 7.2 Hz, 2H), 7.09 (d, *J* = 8.6 Hz, 2H), 6.86 (d, *J* = 8.6 Hz, 2H), 5.66 (s, 1H), 3.98-3.89 (m, 3H), 3.78 (s, 3H), 3.62 (dd, *J* = 12.7, 5.6 Hz, 1H), 2.41-2.29 (m, 2H), 1.20 (t, *J* = 7.0 Hz, 3H); **^{13}C NMR** (100 MHz, $CDCl_3$): δ 198.9, 176.8, 158.5, 141.1, 131.5, 129.6, 128.5, 128.1, 126.8, 113.9, 104.2, 64.6, 55.2, 52.2, 47.3, 40.3, 13.8; **HRMS** (ESI) *m/z*: $[M+H]^+$ calcd. for $C_{21}H_{23}O_3$ 323.1642, found: 323.1636.

5,6-Dihydro-[1,1'-biphenyl]-2(1*H*)-one (6a)[11]

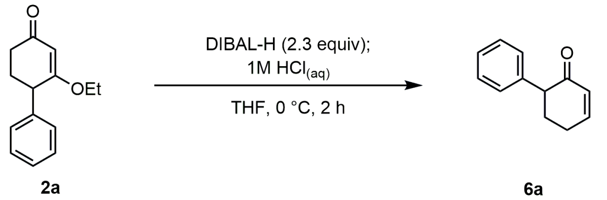

To a round-bottom flask containing **2a** (50 mg, 0.23 mmol) and THF (2.3 mL) at 0 ºC was added a solution of DIBAL-H (1.0 M, 2.3 equiv., 0.53 mL). After stirring at 0 °C for 1.5 hours, 1.0 M $HCl_{(aq)}$ (0.46 mL) was added to the flask. After stirring at 0 °C for 30 minutes, the reaction mixture was diluted with ethyl acetate. The organic layer was sequentially washed with saturated $NaHCO_{3(aq)}$ and brine, dried over anhydrous $MgSO_{4(s)}$, and concentrated with a rotary evaporator. The crude residue was purified by flash column chromatography (EtOAc/hexanes = 1/5) to afford **6a** (31 mg, 79%) as a colorless oil. R_f : 0.33 (EtOAc/hexanes = 1/5). **^{1}H NMR** (400 MHz, $CDCl_3$): δ 7.36-7.30 (m, 2H), 7.29-7.24 (m, 1H), 7.18-7.15 (m, 2H), 7.04 (ddd, *J* = 10.1, 4.6, 3.5 Hz, 1H), 6.17 (ddd, *J* = 10.1, 2.2, 1.7 Hz, 1H), 3.66-3.58 (m, 1H), 2.52-2.45 (m, 2H), 2.32-2.27 (m, 2H). ^{1}H NMR data of **6a** are consistent with those reported in the literature.[11]

4'-Methoxy-5,6-dihydro-[1,1'-biphenyl]-2(1*H*)-one (6b)

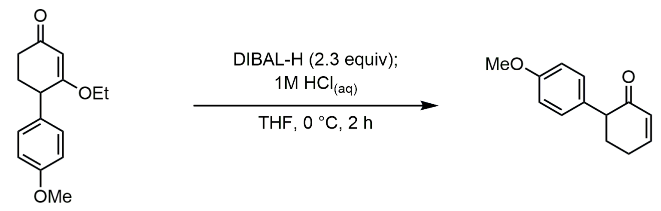

2h **6b**

To a round-bottom flask containing **2h** (41 mg, 0.166 mmol) and THF (1.6 mL) at 0 °C was added a solution of DIBAL-H (1.0 M, 2.3 equiv., 0.38 mL). After stirring for 1.5 hours, 1.0 M $HCl_{(aq)}$ (0.33 mL) was added to the flask. After stirring at 0 °C for 30 minutes, the reaction mixture was diluted with ethyl acetate. The organic layer was sequentially washed with saturated $NaHCO_{3(aq)}$ and brine, dried over anhydrous $MgSO_{4(s)}$, and concentrated with a rotary evaporator. The crude residue was purified by flash column chromatography (EtOAc/hexanes = 1/4) to afford **6b** (29 mg, 86%) as a white solid (m.p. 76-77 ºC). R_f : 0.25 (EtOAc/hexanes = 1/5). **IR** (cast): 2934, 2832, 1671, 1611 cm^{-1}; **^{1}H NMR** (400 MHz, $CDCl_3$): δ 7.09 (d, J = 8.7 Hz, 2H), 7.03 (ddd, J = 10.1, 4.6, 3.4 Hz, 1H), 6.88 (d, J = 8.7 Hz, 2H), 6.15 (ddd, J = 10.1, 2.3, 1.7 Hz, 1H), 3.80 (s, 3H), 3.60-3.53 (m, 1H), 2.51-2.45 (m, 2H), 2.29-2.23 (m, 2H); **^{13}C NMR** (100 MHz, $CDCl_3$):δ 199.7, 158.5, 149.9, 131.3, 130.2, 129.2, 113.9, 55.2, 52.6, 30.8, 25.6; **HRMS** (ESI) *m/z*: $[M+H]^+$ calcd. for $C_{13}H_{15}O_2$ 203.1067, found: 203.1065.

7-(4-Methoxyphenyl)cyclohept-2-en-1-one (6c)

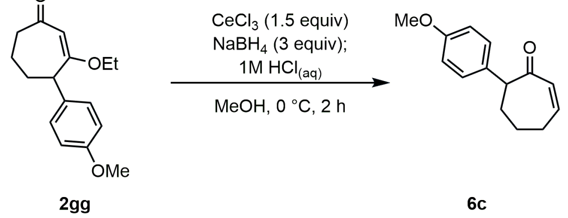

2gg **6c**

To a round-bottom flask containing **2gg** (45 mg, 0.173 mmol), $CeCl_3$ (1.5 equiv., 64 mg), and MeOH (5 mL) at 0 ºC was added $NaBH_4$ (3.0 equiv., 20 mg). After stirring at 0 °C for 1.5 hours, 1.0 M $HCl_{(aq)}$ (0.9 mL) was added to the flask. After stirring at 0 °C for another 30 minutes, the reaction mixture was diluted with ethyl acetate. The

organic layer was sequentially washed with saturated $NaHCO_{3(aq)}$ and brine, dried over anhydrous $MgSO_{4(s)}$, and concentrated with a rotary evaporator. The crude residue was purified by flash column chromatography (EtOAc/hexanes = 1/4) to afford **6c** (30 mg, 80%) as a colorless liquid. R*f* : 0.2 (EtOAc/hexanes = 1/5). **IR** (film): 2937, 2863, 1664, 1610 cm^{-1}; **^{1}H NMR** (400 MHz, $CDCl_3$): δ 7.14 (d, *J* = 8.8 Hz, 2H), 6.86 (d, *J* = 8.8 Hz, 2H), 6.60 (dt, *J* = 12.2, 5.1 Hz, 1H), 6.10 (dt, *J* = 12.2, 1.8 Hz, 1H), 3.84 (dd, *J* = 11.0, 5.8 Hz, 1H), 3.79 (s, 3H), 2.54-2.48 (m, 2H), 2.17-1.98 (m, 2H), 1.93-1.86 (m, 2H); **^{13}C NMR** (100 MHz, $CDCl_3$): δ 204.2, 158.4, 145.2, 132.7, 132.3, 129.4, 113.8, 58.1, 55.2, 31.1, 30.7, 25.0; **HRMS** (EI) *m/z*: $[M]^+$ calcd. for $C_{14}H_{17}O_2$ 216.1156, found: 216.1150.

4-Methyl-5,6-dihydro-[1,1'-biphenyl]-2(1*H*)-one (6d)[12]

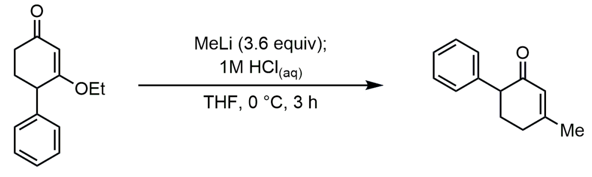

2a 6d

To a round-bottom flask containing **2a** (50 mg, 0.23 mmol) and THF (2 mL) at 0 ºC was added MeLi (3.1 M, 3.6 equiv., 0.27 mL), and the resulting mixture was stirred at 0 °C for 2 hours. 1.0 M $HCl_{(aq)}$ (0.7 mL) was added to the flask at 0 °C. After stirring at 0 °C for 1 hour, the reaction mixture was diluted with ethyl acetate. The organic layer was sequentially washed with saturated $NaHCO_{3(aq)}$ and brine, dried over anhydrous $MgSO_{4(s)}$, and concentrated with a rotary evaporator. The crude residue was purified by flash column chromatography (EtOAc/hexanes = 1/6) to afford **6d** (33 mg, 77%) as a white solid (m.p. 58-59 ºC). R*f* : 0.3 (EtOAc/hexanes = 1/6). **^{1}H NMR** (400 MHz, $CDCl_3$): δ 7.35-7.30 (m, 2H), 7.27-7.23 (m, 1H), 7.17-7.14 (m, 2H), 6.04-6.02 (m, 1H),

3.52 (dd, J = 9.1, 6.5 Hz, 1H), 2.50-2.23 (m, 4H), 2.00 (s, 3H). ^{1}H NMR data of **6d** are consistent with those reported in the literature.[12]

4-Vinyl-5,6-dihydro-[1,1'-biphenyl]-2(1*H*)-one (6e)

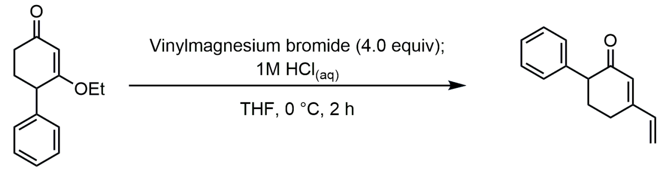

2a 6e

To a round-bottom flask containing **2a** (40 mg, 0.19 mmol) and THF (2 mL) at 0 ºC was added a solution of vinylmagnesium bromide (0.7 M in THF, 4.0 equiv., 1.1 mL). After stirring at 0 °C for 1 hour, 1.0 M $HCl_{(aq)}$ (2 mL) was added to the flask. After stirring at 0 °C for 1 hour, the reaction mixture was diluted with ethyl acetate. The organic layer was sequentially washed with saturated $NaHCO_{3(aq)}$ and brine, dried over anhydrous $MgSO_{4(s)}$, and concentrated with a rotary evaporator. The crude residue was purified by flash column chromatography (EtOAc/hexanes = 1/10) to afford **6e** (32 mg, 86%) as a white oil. R_f: 0.38 (EtOAc/hexanes = 1/6). **IR** (film): 3033, 2931, 1660, 1577 cm^{-1}; **^{1}H NMR** (400 MHz, $CDCl_3$): δ 7.35-7.30 (m, 2H), 7.28-7.22 (m, 1H), 7.17-7.14 (m, 2H), 6.54 (dd, J = 17.5, 10.7 Hz, 1H), 6.10 (s, 1H), 5.70 (d, J = 17.5 Hz, 1H), 5.49 (d, J = 10.7 Hz, 1H), 3.60 (dd, J = 10.6, 5.2 Hz, 1H), 2.67-2.59 (m, 1H), 2.57-2.48 (m, 1H), 2.39-2.23 (m, 2H); **^{13}C NMR** (100 MHz, $CDCl_3$): δ 200.0, 156.4, 139.4, 137.7, 128.5, 128.3, 126.9, 120.9, 52.9, 30.2, 23.8 [One sp^2 carbon is missing due to peak overlapping]; **HRMS** (ESI) *m/z*: [M+H]$^+$ calcd. for $C_{14}H_{15}O$ 199.1117, found: 199.1112.

4-(Phenylethynyl)-5,6-dihydro-[1,1'-biphenyl]-2(1*H*)-one (6f)

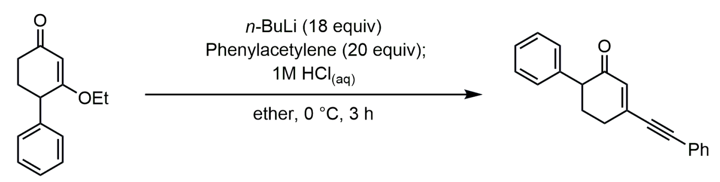

2a **6f**

To a round-bottom flask containing phenylacetylene (20 equiv., 470 mg) and ether (7.3 mL) at −78 °C was slowly added a solution of *n*-BuLi (2.5 M in hexane, 18 equiv., 1.65 mL), and the temperature was kept at −78 °C for 1 hour. The solution was then warmed to 0 °C and then transferred to another round-bottom flask containing **2a** (50 mg, 0.23 mmol) and ether (1 mL) at 0 ºC. After stirring at 0 ºC for 2 hours, THF (2 mL) and 1.0 M $HCl_{(aq)}$ (4 mL) was added to the flask. After stirring at 0 °C for 1 hour, the reaction mixture was diluted with ethyl acetate. The organic layer was sequentially washed with saturated $NaHCO_{3(aq)}$ and brine, dried over anhydrous $MgSO_{4(s)}$, and concentrated with a rotary evaporator. The crude residue was purified by flash column chromatography (EtOAc/hexanes = 1/10) to afford **6f** (55 mg, 88%) as a yellow solid (m.p. 104-105 ºC). R_f: 0.48 (EtOAc/hexanes = 1/6). **IR** (cast): 3066, 2942, 2197, 1664 cm^{-1}; **^{1}H NMR** (400 MHz, $CDCl_3$): δ 7.51-7.48 (m, 2H), 7.38-7.31 (m, 5H), 7.29-7.24 (m, 1H), 7.18-7.15 (m, 2H), 6.43 (t, *J* = 1.6 Hz, 1H), 3.66-3.58 (m, 1H), 2.70-2.61 (m, 2H), 2.37-2.31 (m, 2H); **^{13}C NMR** (100 MHz, $CDCl_3$): δ 198.5, 143.0, 139.1, 132.7, 131.9, 129.5, 128.56, 128.5, 128.2, 127.0, 121.9, 99.9, 88.3, 52.5, 30.6, 29.9; **HRMS** (ESI) *m/z*: $[M+H]^+$ calcd. for $C_{20}H_{17}O$ 273.1274, found: 273.1275.

Enolate Formation-Quenching Experiments

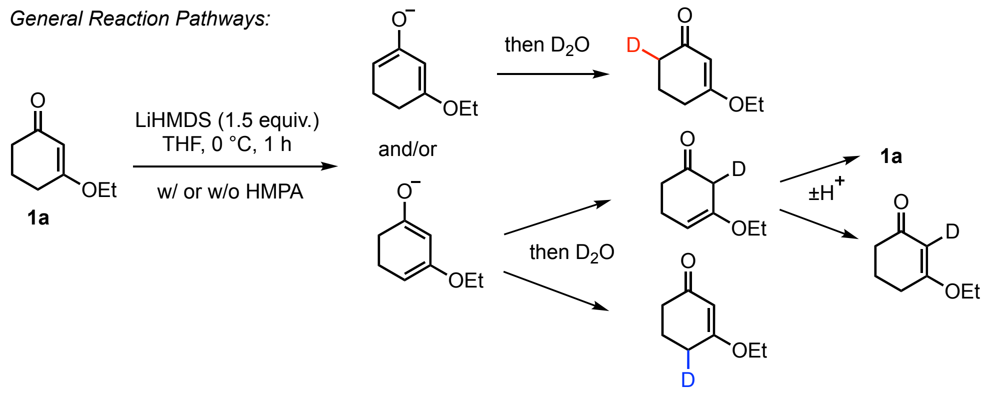

(a) D_2O-Quenching Experiments for Probing Enolate Site Selectivity

To a round-bottom flask containing **1a** (1.0 mmol) and THF (5 mL), in the presence or absence of HMPA (2.5 equiv.) was added a solution of LiHMDS (1.5 equiv., 0.62 M in THF) at 0 ºC. After stirring at 0 °C for the indicated time, an aliquot of the solution (1 mL) was transferred to another flame-dried vial, and D_2O (0.1 mL) was added. The mixture was diluted with a small amount of ethyl acetate. The organic layer was washed with brine and concentrated under vacuum. ^{1}H NMR spectrum of the crude residue was analyzed without further purification.

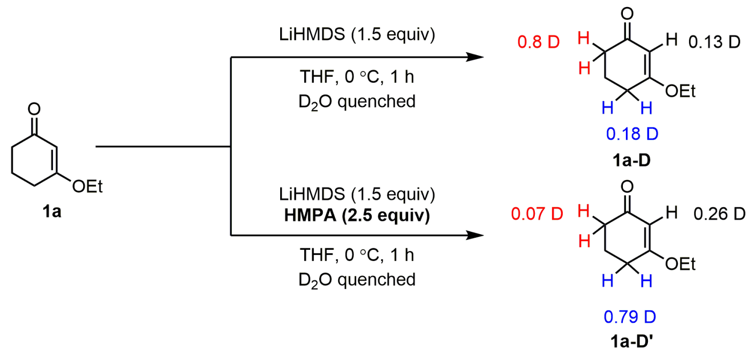

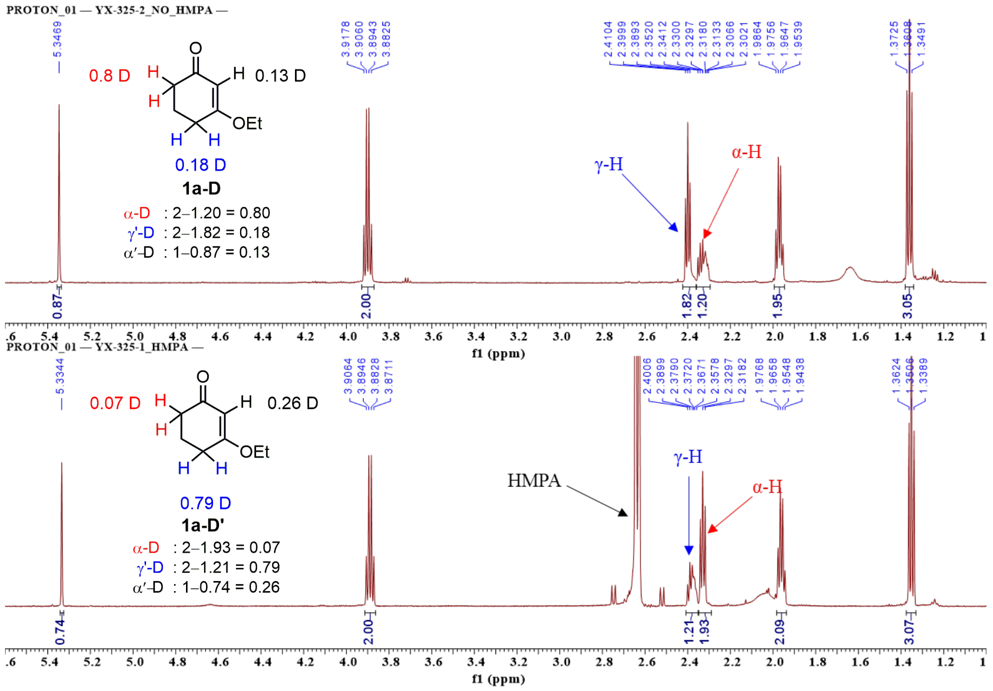

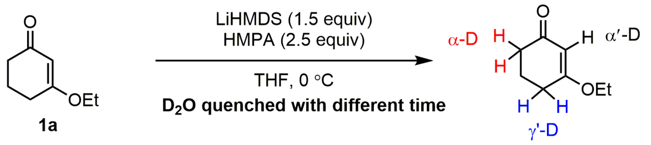

time (min)	α-D	γ'-D	α'-D
<1*	0.1	0.79	0.29
10	0.09	0.79	0.27
20	0.1	0.79	0.28
40	0.11	0.8	0.29
60	0.09	0.81	0.29

*The sample transfer and D_2O-quenching was conducted after the addition of LiHMDS.

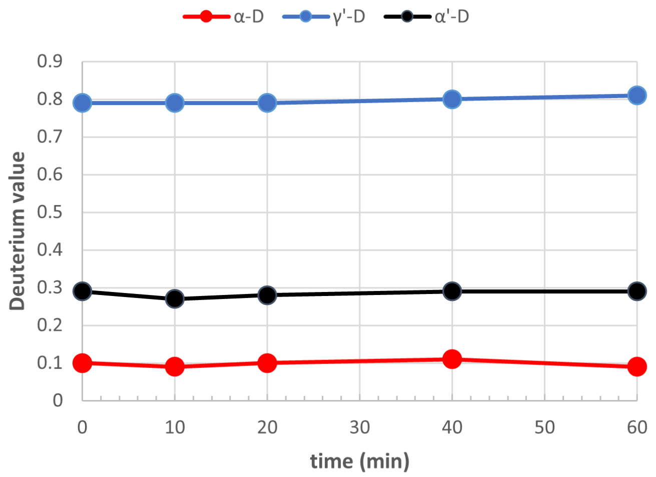

(b) Post-Addition HMPA, D_2O-Quenching Experiment (Time-Dependent Drift Study)

To a round-bottom flask containing **1a** (2.0 mmol) and THF (10 mL) was added a solution of LiHMDS (1.5 equiv., 0.62 M in THF) at 0 ºC for 1 h. To the stirring mixture was slowly added HMPA (2.5 equiv.). After stirring at 0 °C for the indicated time, an aliquot of the solution (0.5 mL) was transferred to another flame-dried vial, and D_2O

(0.1 mL) was added. The mixture was diluted with a small amount of ethyl acetate. The organic layer was washed with brine and concentrated under vacuum. ^{1}H NMR spectrum of the crude residue was analyzed without further purification.

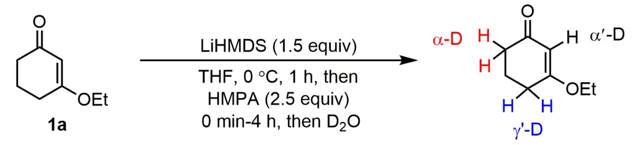

time (min)	α-D	γ'-D	α′-D
<1*	0.8	0.22	0.11
30	0.49	0.43	0.14
60	0.46	0.52	0.15
90	0.35	0.64	0.17
120	0.32	0.75	0.16
150	0.26	0.8	0.21
180	0.26	0.86	0.16
210	0.24	0.86	0.22
240	0.23	0.84	0.19

*The sample transfer and D_2O-quenching was conducted after the addition of LiHMDS.

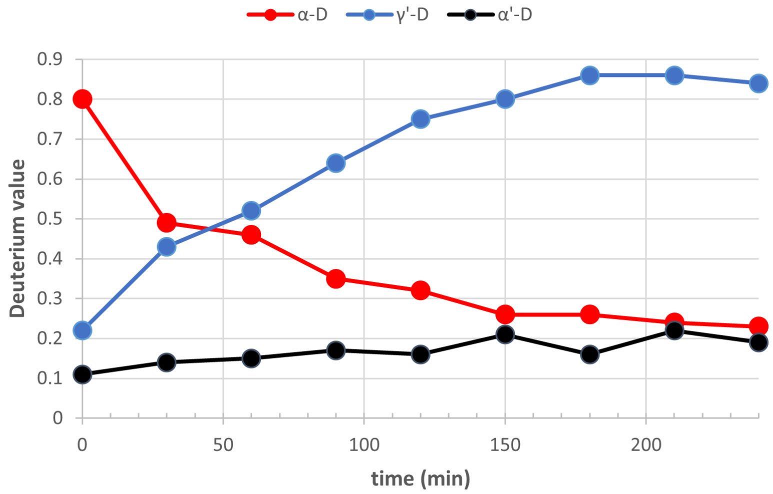

(c) Iterative γ′-Deuteration of **1a** via LiHMDS/HMPA-Mediated Enolate Formation and D_2O Quench

*First-round deuteration (preparation of **1a-Dx1**).*

To a flame-dried round-bottom flask equipped with a stir bar were added **1a**, HMPA (2.5 equiv.), and THF (0.1 M). The resulting solution was stirred at room temperature for 30 min and then cooled to 0 °C for 10 min. A solution of LiHMDS (1.0 M in THF, 1.5 equiv.) was added dropwise, and the reaction mixture was stirred at 0 °C for 1 h. An aliquot of the enolate mixture was transferred to a separate flame-dried vial, quenched with D_2O, diluted with EtOAc, and washed with saturated brine. The

organic layer was filtered through a pad of Celite, dried over anhydrous $MgSO_4$, and concentrated under reduced pressure. The crude residue was purified by flash column chromatography (EtOAc/hexanes = 1:3) to afford the first-round deuterated product **1a-Dx1**.

*Subsequent rounds (preparation of **1a-Dx2–1a-Dx4**)*

Compounds **1a-Dx2**, **1a-Dx3**, and **1a-Dx4** were prepared by repeating the above deprotonation/D_2O-quench sequence using **1a-Dx1**, **1a-Dx2**, and **1a-Dx3**, respectively, as the starting material. In each iteration, the crude residue was purified by flash column chromatography (EtOAc/hexanes = 1:3) to afford the corresponding deuterated products, resulting in progressively higher deuterium incorporation at the γ’ position. Deuterium incorporation levels (α and γ’) were determined by ^{1}H NMR analysis as described.

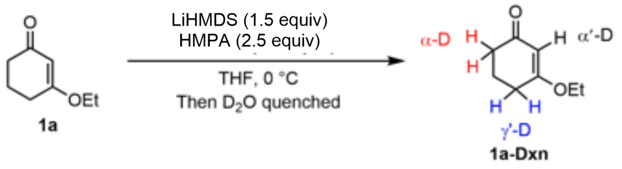

Number of Deuteration	α-D	γ'-D	α'-D
1	0.07	0.73	0.21
2	0.10	1.38	0.35
3	0.54	1.73	0.44
4	0.93	1.86	0.53

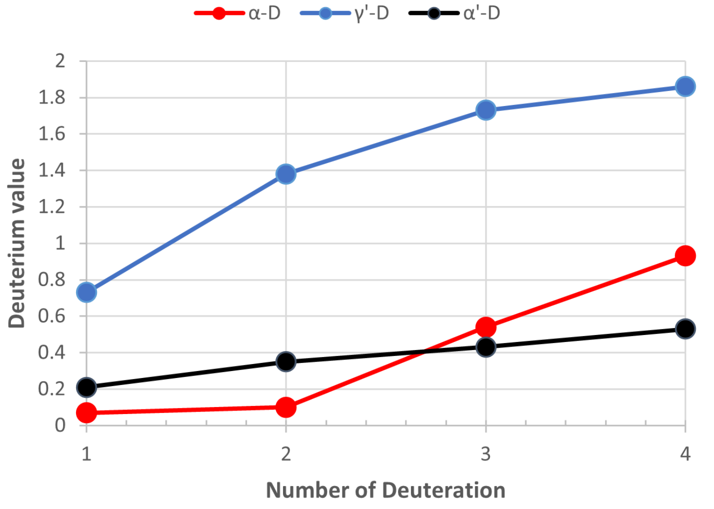

(d) H/D Exchange Control via H_2O Quench of **1a-Dx3** Enolate

A flame-dried round-bottom flask charged with **1a-Dx3** (1.0 equiv.), HMPA (2.5 equiv.), and THF (0.1 M) was stirred at room temperature for 30 min and then cooled to 0 °C for 10 min. A solution of LiHMDS (1.0 M in THF, 1.5 equiv.) was added dropwise, and the mixture was stirred at 0 °C for 1 h. An aliquot of the resulting enolate solution was then transferred to a separate flame-dried vial and quenched with H_2O. The vial was shaken thoroughly, then EtOAc and brine were added to aid phase separation. The organic layer was transferred to an NMR tube and concentrated under reduced pressure to afford **1a-Dx3+H** (used directly for NMR analysis).

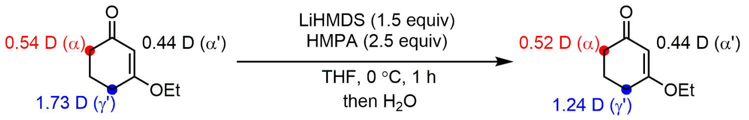

1a-Dx3 **1a-Dx3+H**

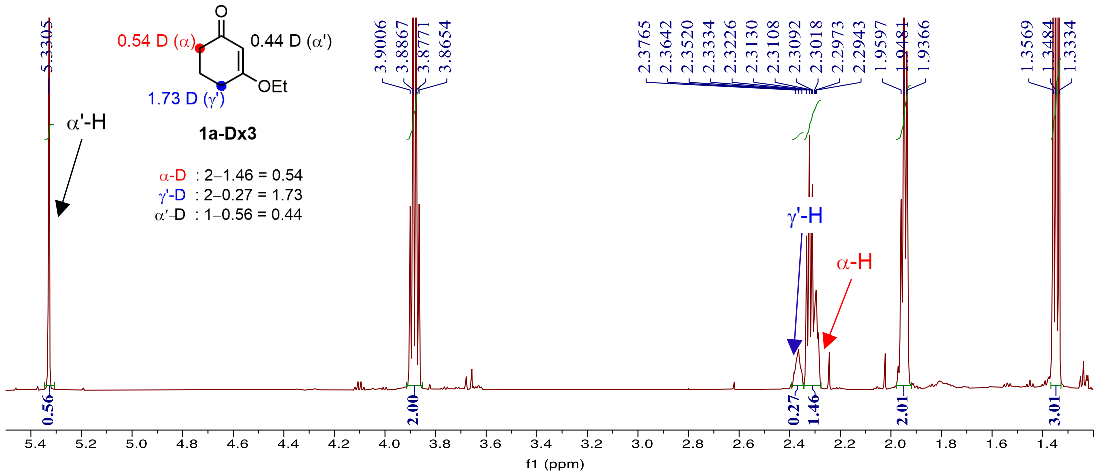

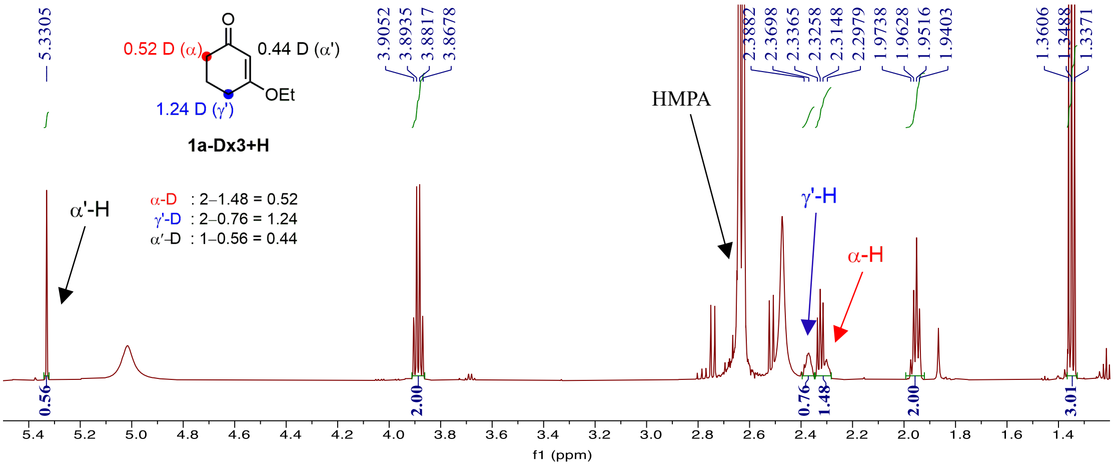

ORTEP Diagram of 2c. Single crystals of $C_{20}H_{20}O_2$ were obtained by slow evaporation in chloroform. A suitable crystal was selected and measured on a XtaLAB Synergy R, DW system, HyPix-Arc 150 diffractometer. The crystal was kept at 100.01(10) K during data collection. Using Olex2, the structure was solved with the SHELXT structure solution program using Intrinsic Phasing and refined with the SHELXL refinement package using Least Squares minimisation. Crystal Data for $C_{20}H_{20}O_2$ (M =292.36 g/mol): monoclinic, space group P2$_1$/n (no. 14), a = 5.53771(19) Å, b = 8.3080(3) Å, c = 33.2914(12) Å, β = 94.561(3)°, V = 1526.80(9) Å^3, Z = 4, T = 100.01(10) K, μ(Cu Kα) = 0.634 mm^{-1}, $Dcalc$ = 1.272 g/cm^3, 16347 reflections measured (5.326° ≤ 2Θ ≤ 134.146°), 2712 unique (R_{int} = 0.0477, R_{sigma} = 0.0361) which were used in all calculations. The final R_1 was 0.0397 (I > 2σ(I)) and wR_2 was 0.1069 (all data). CCDC 2286421 contains the supplementary crystallographic data for this paper. These data can be obtained free of charge via www.ccdc.cam.ac.uk/data_request/cif.

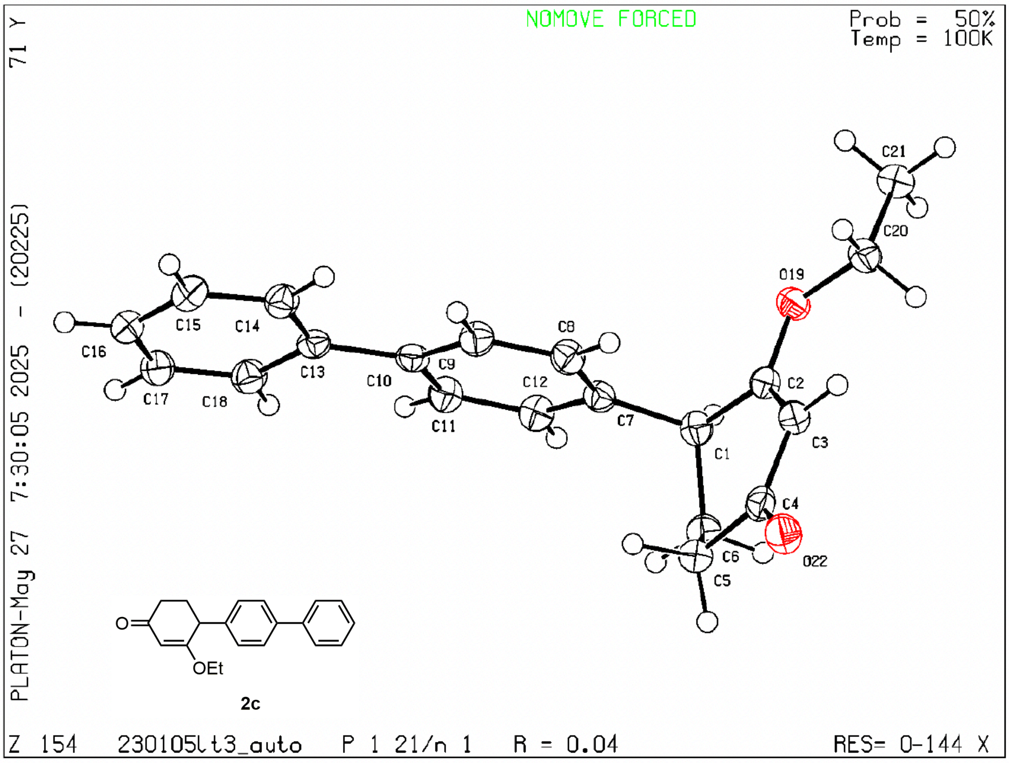

ORTEP Diagram of 2t. Single crystals of $C_{19}H_{24}O_5$ were obtained by slow evaporation in chloroform. A suitable crystal was selected and measured on a XtaLAB Synergy R, DW system, HyPix-Arc 150 diffractometer. The crystal was kept at 100.0(2) K during data collection. Using Olex2, the structure was solved with the SHELXT structure solution program using Intrinsic Phasing and refined with the SHELXL refinement package using Least Squares minimisation. Crystal Data for $C_{19}H_{24}O_5$ (M =332.38 g/mol): monoclinic, space group $P2_1/c$ (no. 14), a = 8.95493(17) Å, b = 23.3404(4) Å, c = 8.9134(2) Å, β = 114.973(3)°, V = 1688.83(6) Å^3, Z = 4, T = 100.0(2) K, μ(Cu Kα) = 0.768 mm^{-1}, $Dcalc$ = 1.307 g/cm^3, 20911 reflections measured (7.576° ≤ 2Θ ≤ 134.15°), 2982 unique (R_{int} = 0.0321, R_{sigma} = 0.0164) which were used in all calculations. The final R_1 was 0.0366 (I > 2σ(I)) and wR_2 was 0.1019 (all data). CCDC 2286420 contains the supplementary crystallographic data for this paper. These data can be obtained free of charge via www.ccdc.cam.ac.uk/data_request/cif.

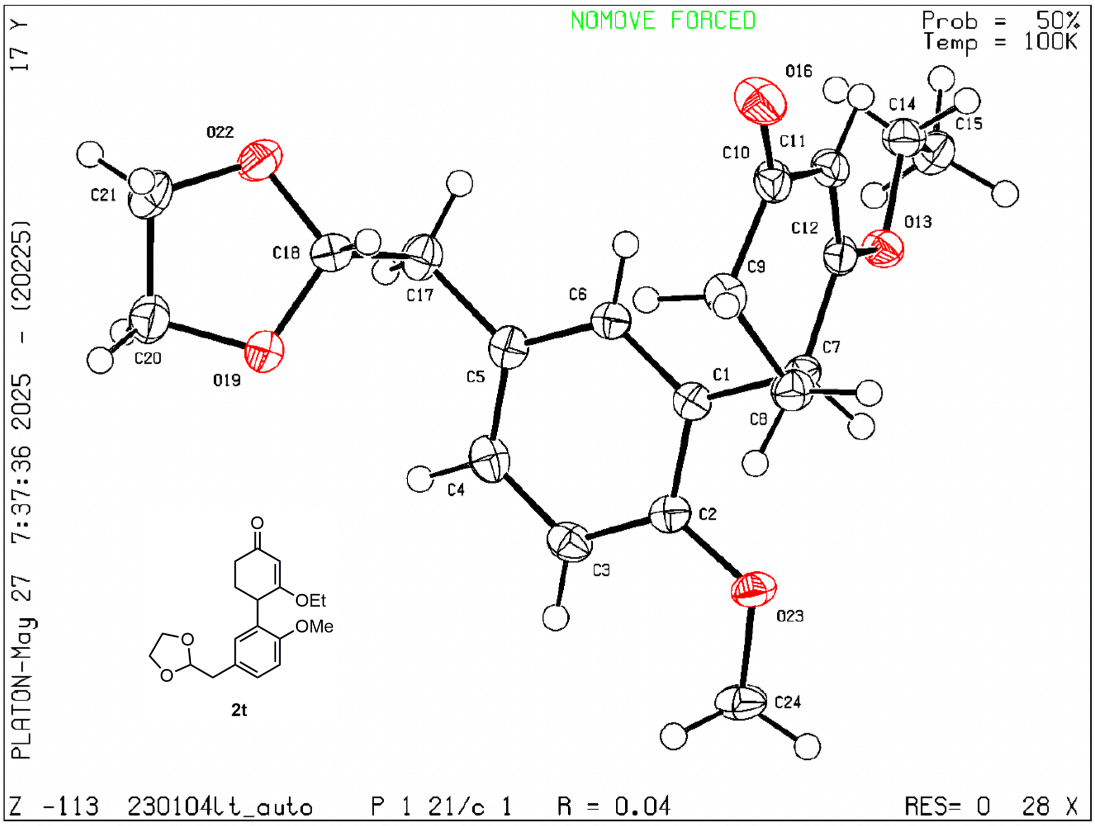

References

[1] Shao, L.-D.; Chen, Y.; Wang, M.; Xiao, N.; Zhang, Z.-J.; Li, D.; Li, R.-T. *Org. Chem. Front.* 2022, *9*, 2308-2315.

[2] Nakamura, H.; Sugiyama, K.; Ohta, K.; Yasutake, M. *J. Mater. Chem. C Mater. Opt. Electron. Devices* **2017**, *5*, 7297–7306.

[3] Graßl, R.; Jandl, C.; Bach, T. *J. Org. Chem.* 2020, *85*, 11426–11439.

[4] Parvathalu, N.; Agalave, S. G.; Mohanta, N.; Gnanaprakasam, B. *Org. Biomol. Chem.* 2019, *17*, 3258-3266.

[5] Curini, M.; Epifano, F.; Genovese, S. *Tetrahedron Lett.* 2006, *47*, 4697-4700.

[6] Szymor-Pietrzak, D.; Khan, M. N.; Pagès, A.; Kumar, A.; Depner, N.; Clive, D. L. J. *J. Org. Chem.* 2021, *86*, 619-631.

[7] Blouin, S.; Pertschi, R.; Schoenfelder, A.; Suffert, J.; Blond, G. *Adv. Synth. Catal Catal.* 2018, *360*, 2166–2171.

[8] Handy, S. T.; Omune, D. *Org. Lett.* 2005, *7*, 1553–1555.

[9] Johnson, T.; Pultar, F.; Menke, F.; Lautens, M. *Org. Lett.* 2016, *18*, 6488–6491.

[10] Foote, K. M.; Hayes, C. J.; John, M. P.; Pattenden, G. *Org. Biomol. Chem.* 2003, *1*, 3917–3948.

[11] List, B.; Shevchenko, G.; Dehn, S. *Synlett* 2018, *29*, 2298-2300.

[12] Han, X.; Wang, X.; Pei, T.; Widenhoefer, R. A. *Chem. Eur. J.* 2004, *10*, 6333-6342.

PROTON_01 — — exp182-pure —

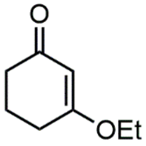

1a

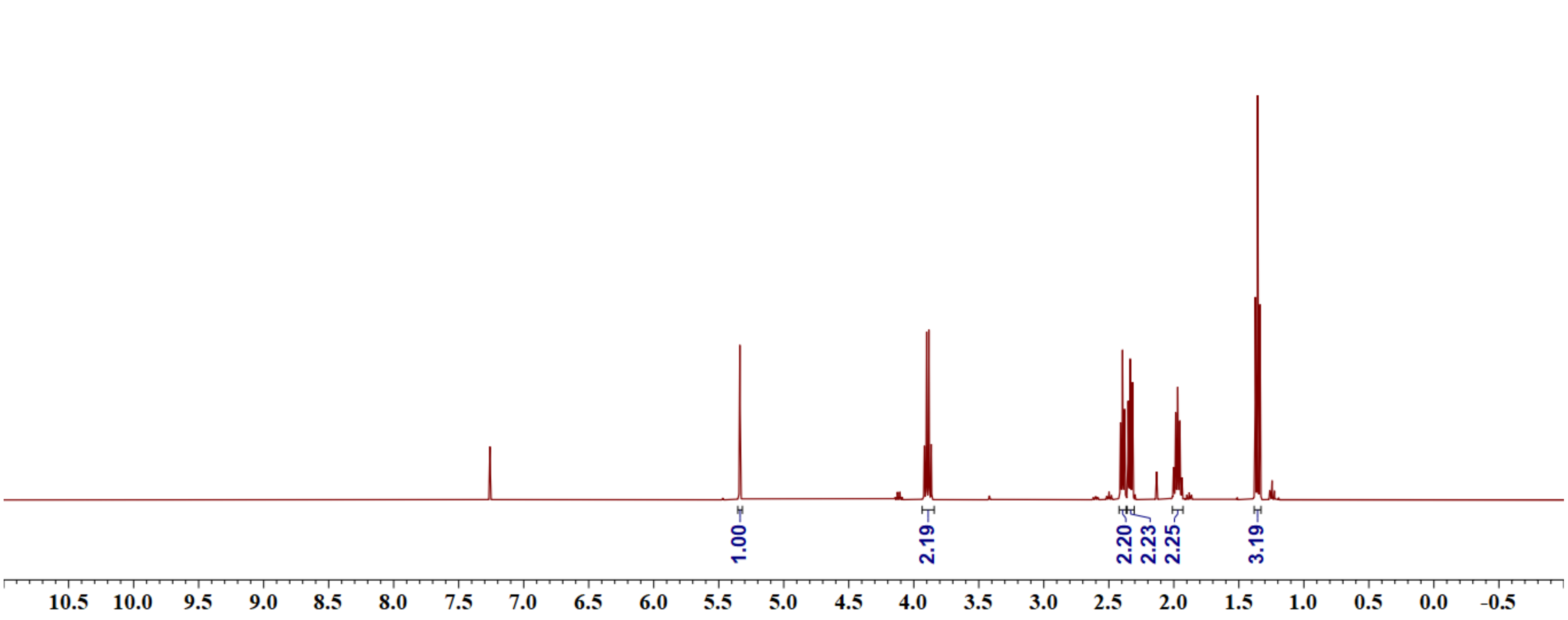

^{1}H NMR Spectrum of Compound 1a

PROTON_01 — KLC-01-009-p —

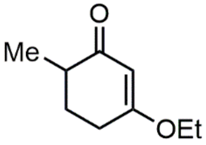

1b

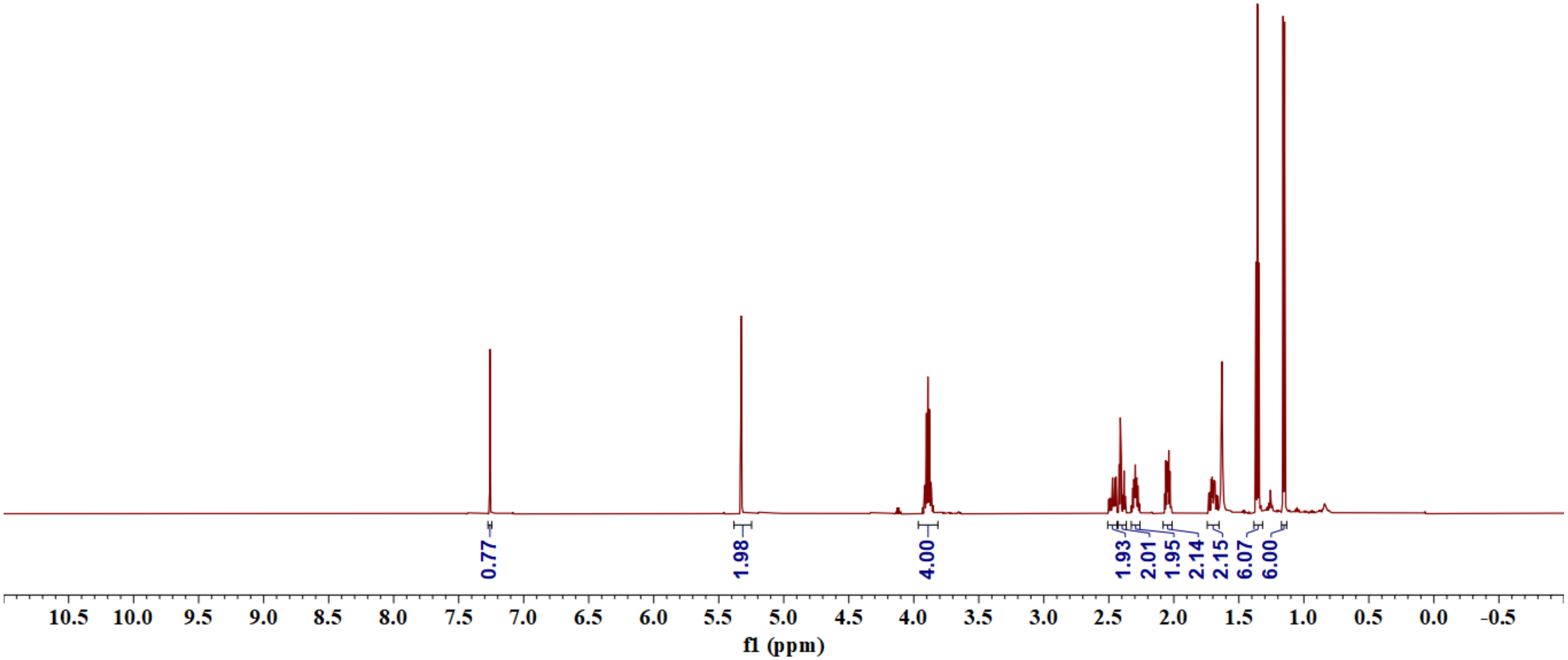

^{1}H NMR Spectrum of Compound 1b

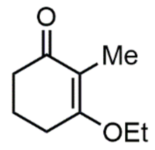

1c

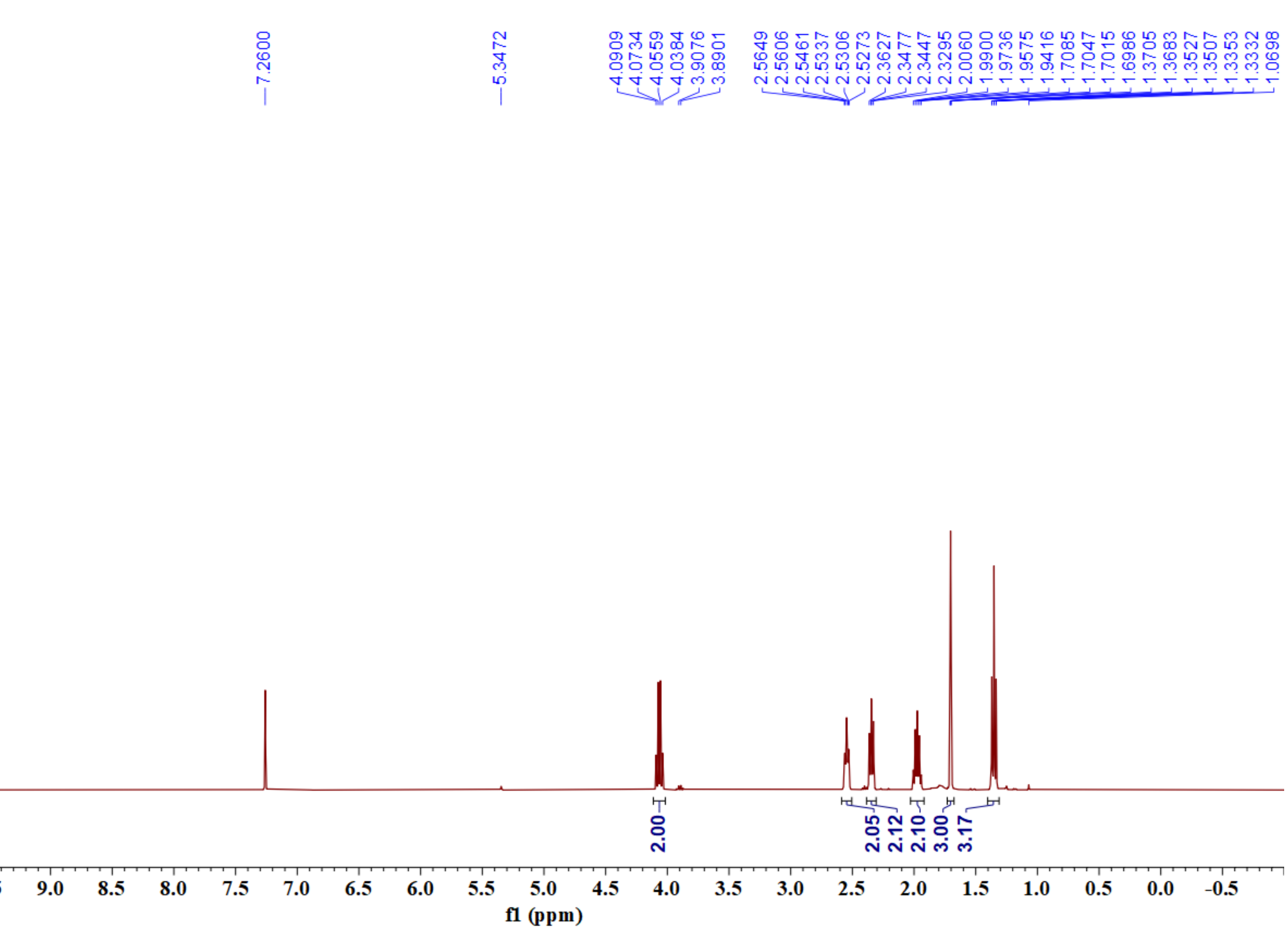

^{1}H NMR Spectrum of Compound 1c

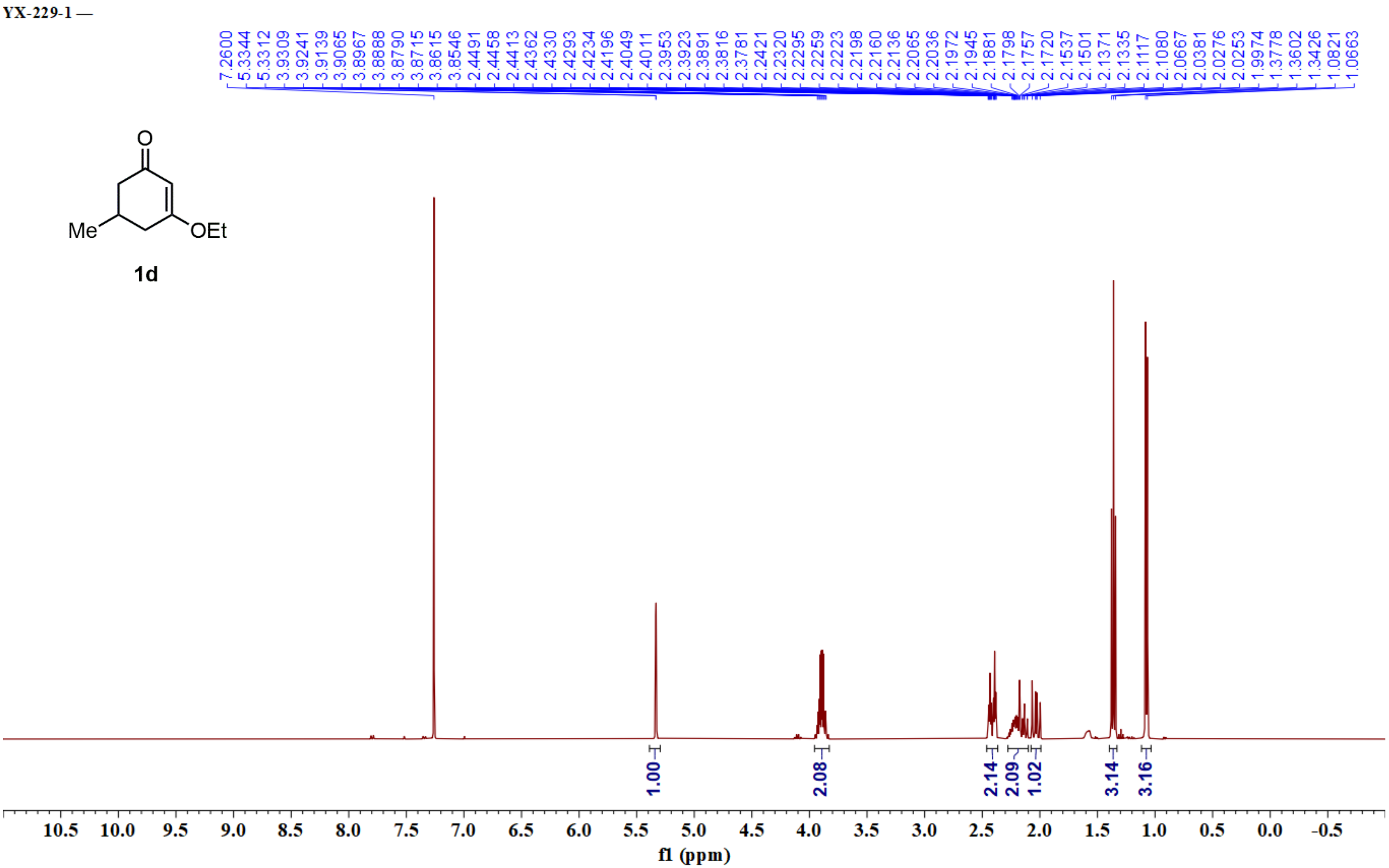

^{1}H NMR Spectrum of Compound 1d

YX-218-1 —

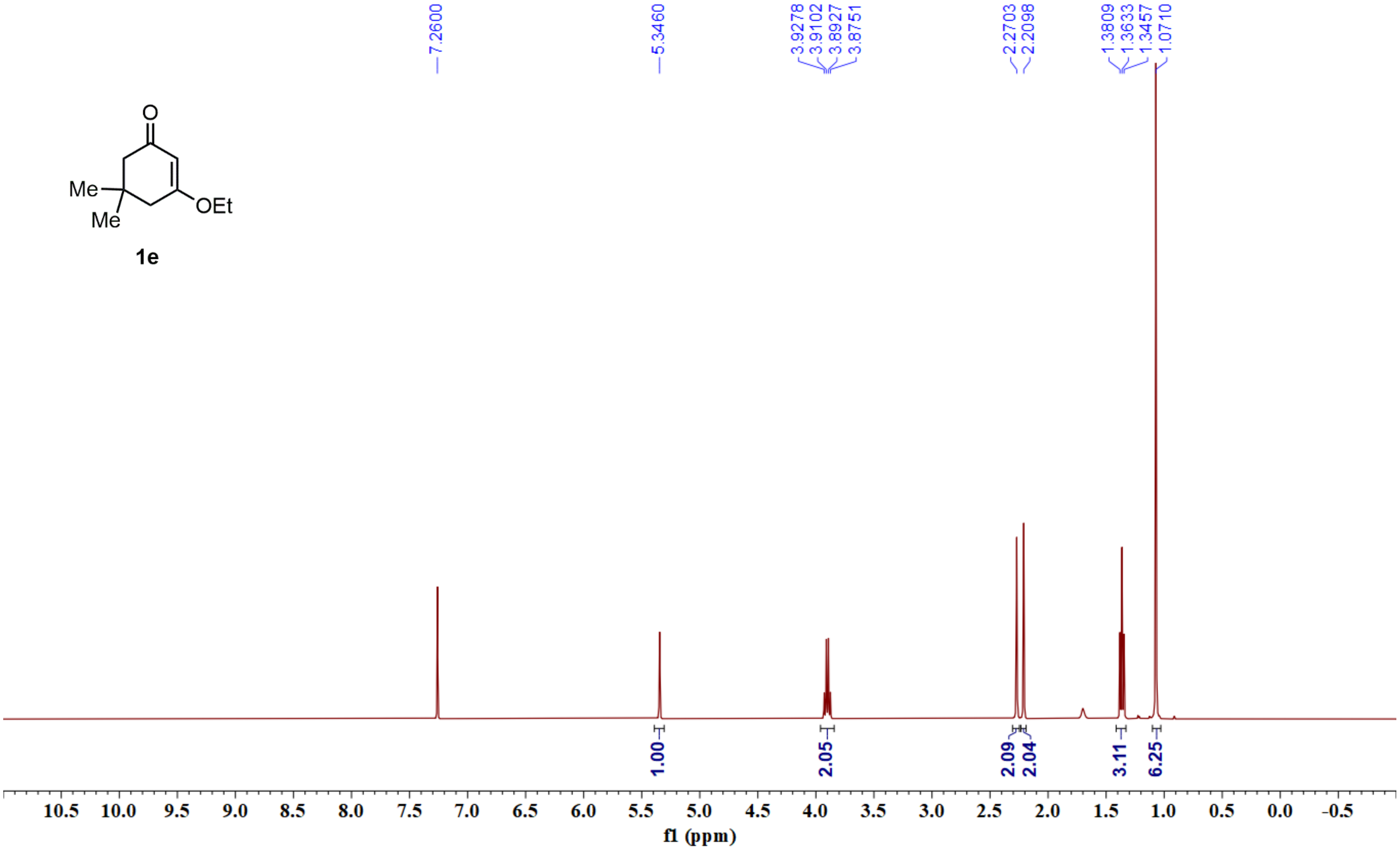

^{1}H NMR Spectrum of Compound 1e

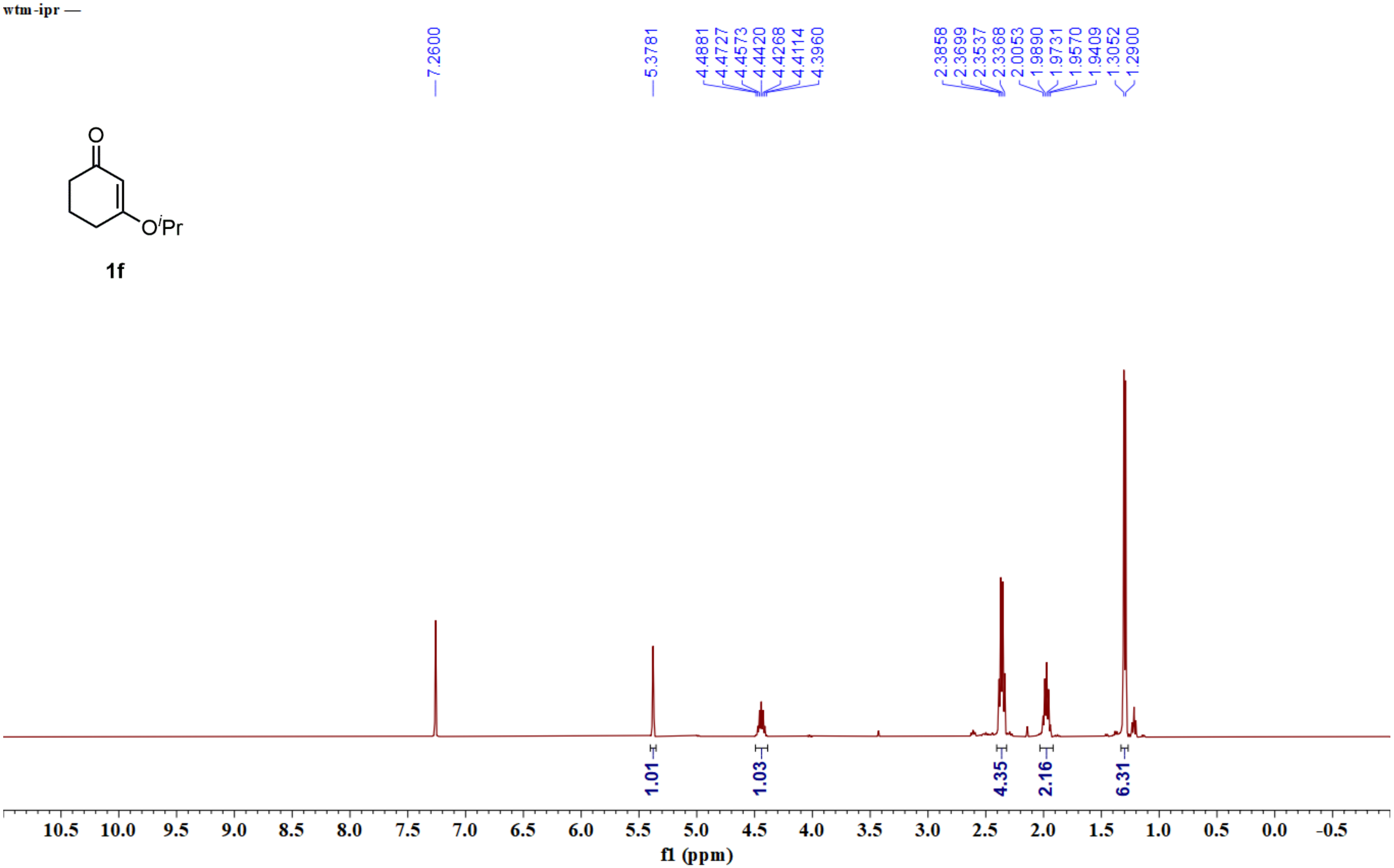

^{1}H NMR Spectrum of Compound 1f

YX-217-1 —

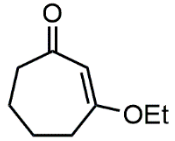

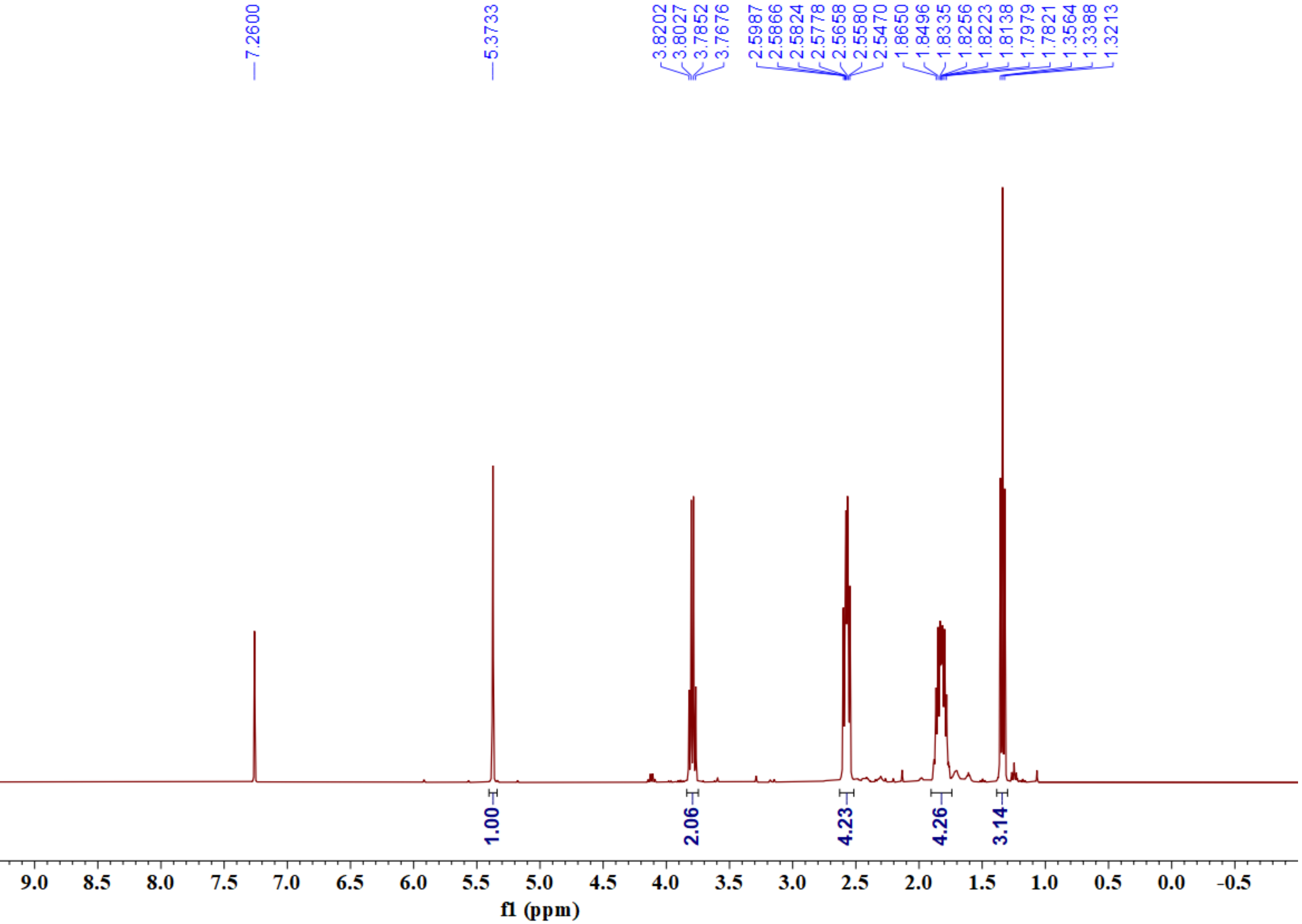

^{1}H NMR Spectrum of Compound 1g

cyc-dixc-xan-2.2g-c —

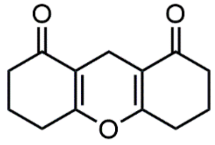

1h

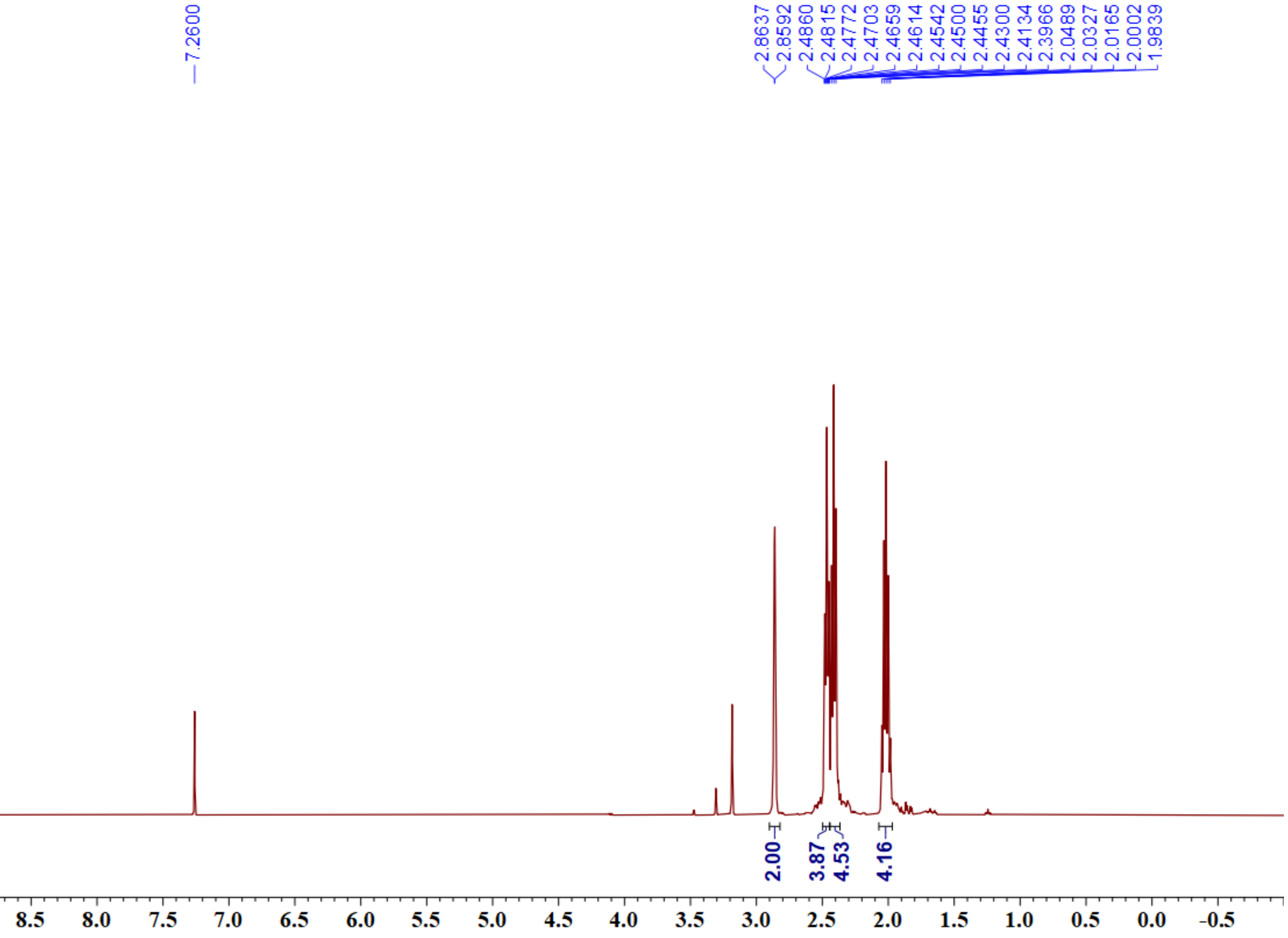

^{1}H NMR Spectrum of Compound 1h

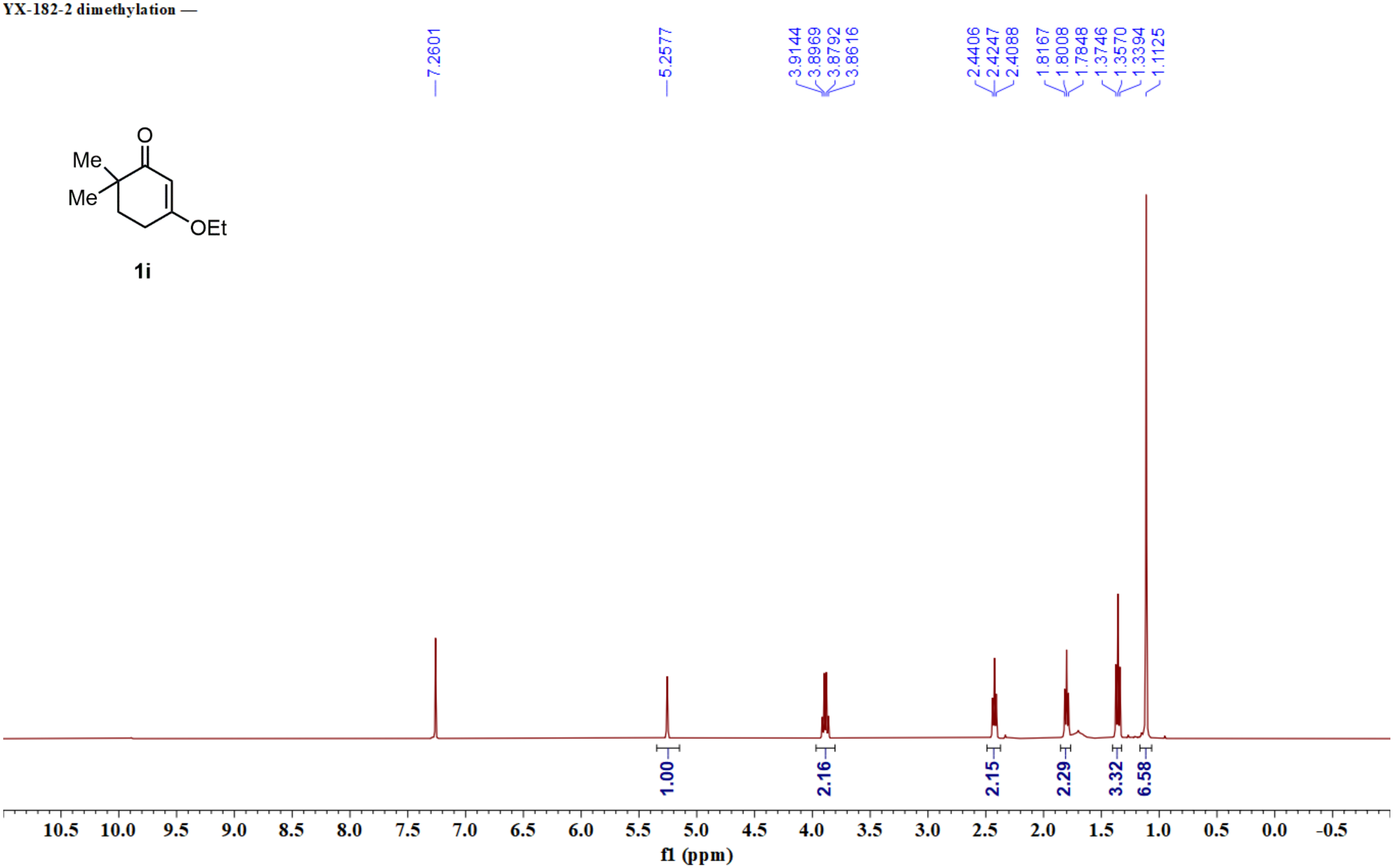

^{1}H NMR Spectrum of Compound 1i

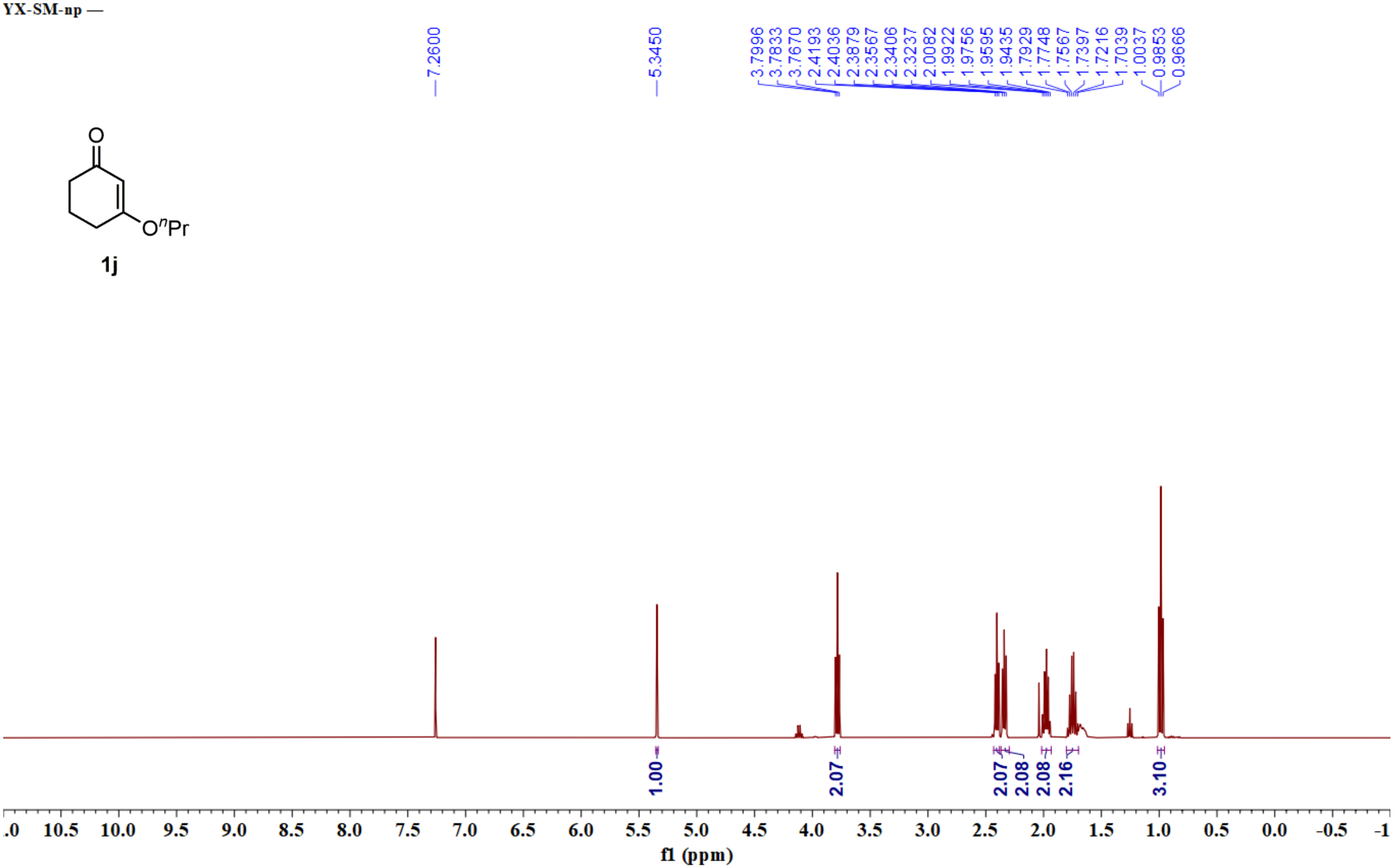

^{1}H NMR Spectrum of Compound 1j

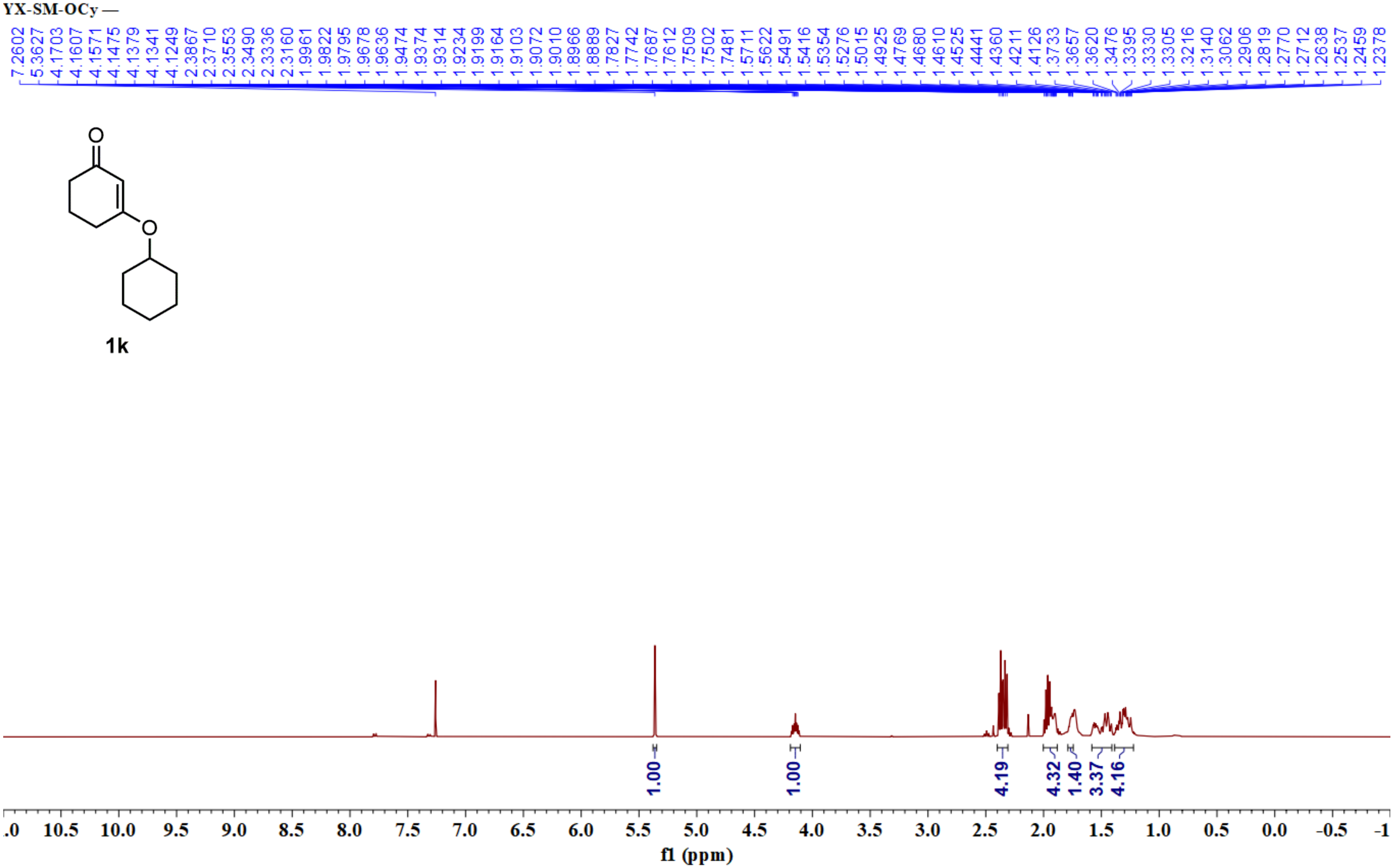

^{1}H NMR Spectrum of Compound 1k

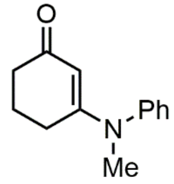

1l

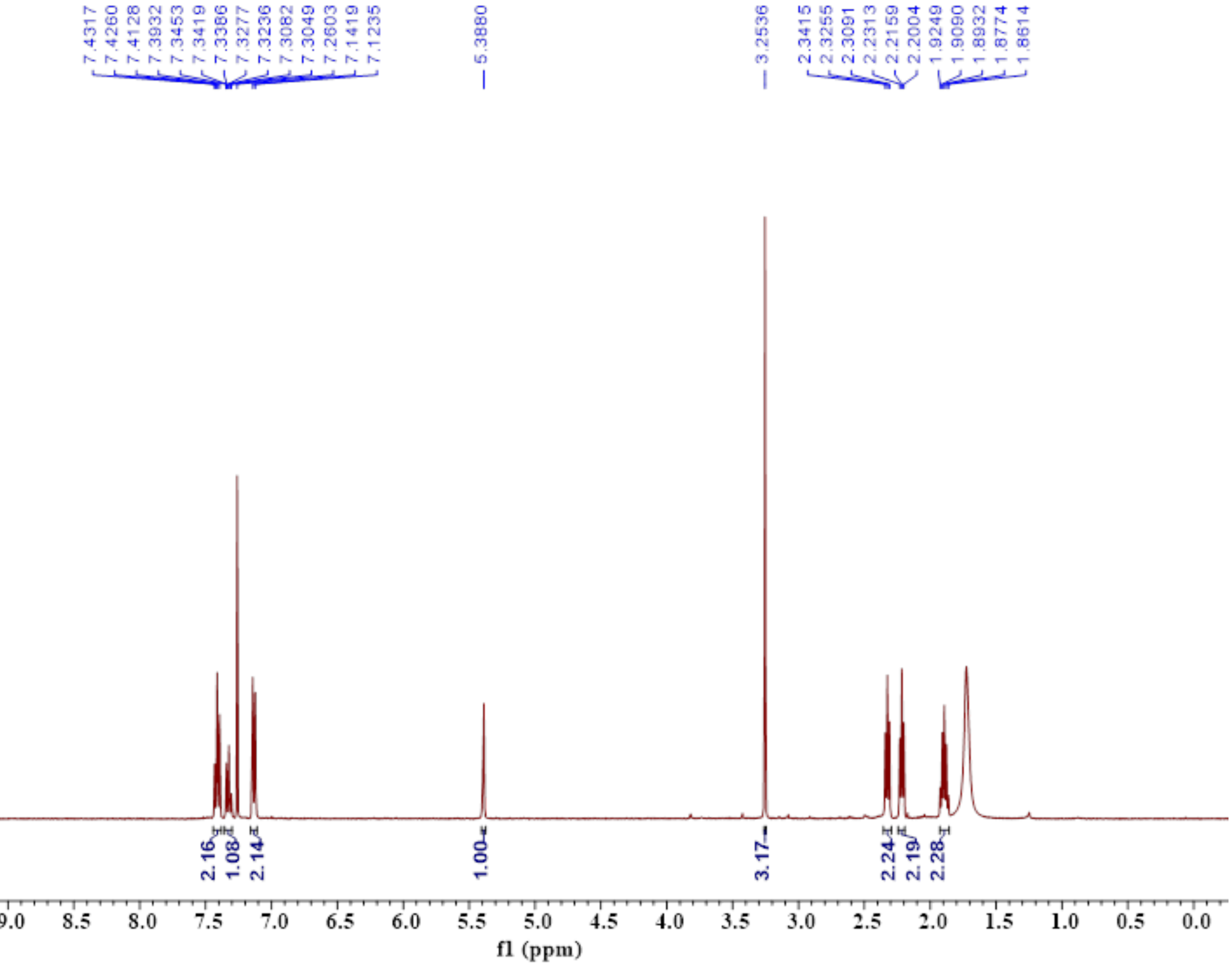

^{1}H NMR Spectrum of Compound 1l

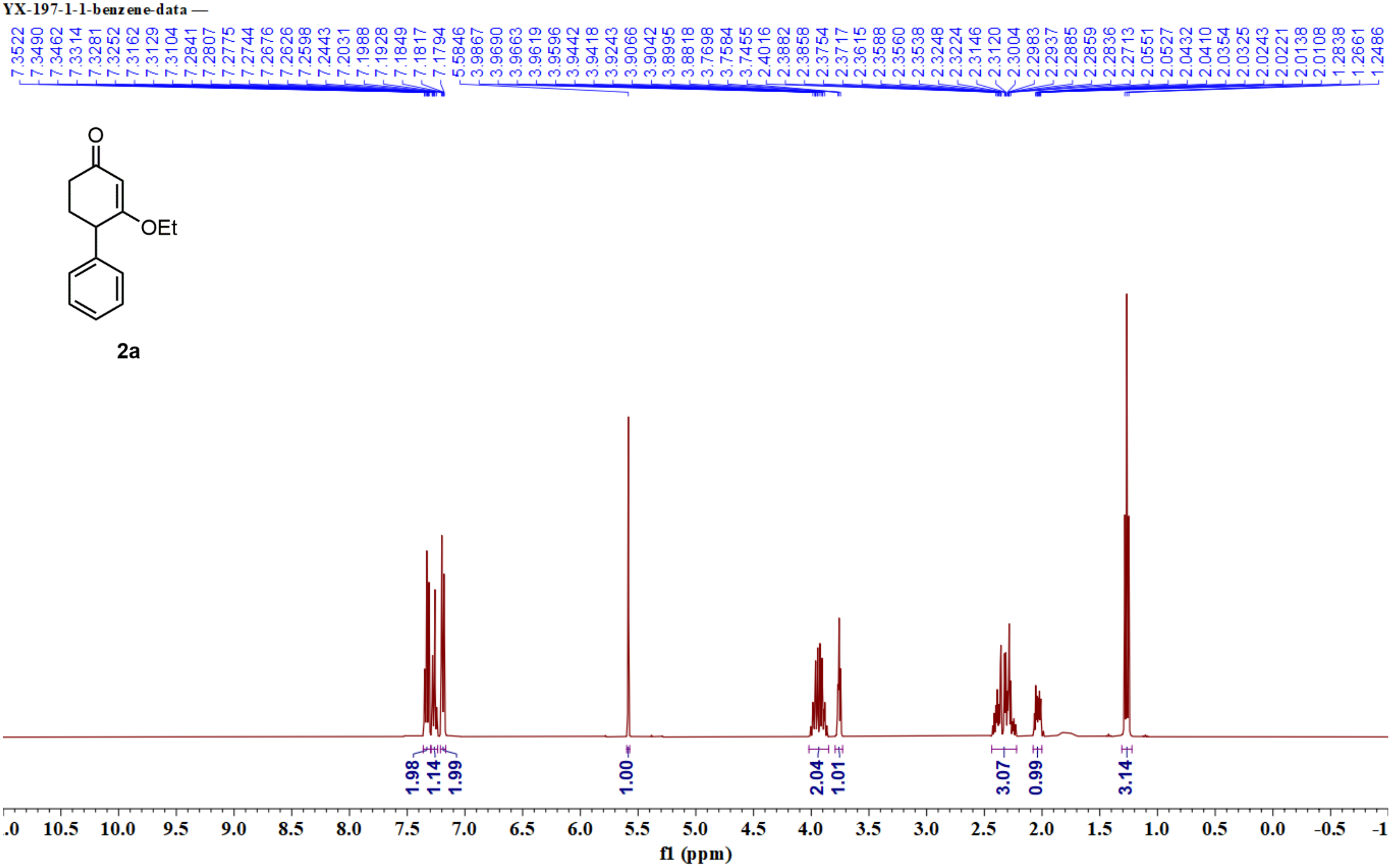

^{1}H NMR Spectrum of Compound 2a

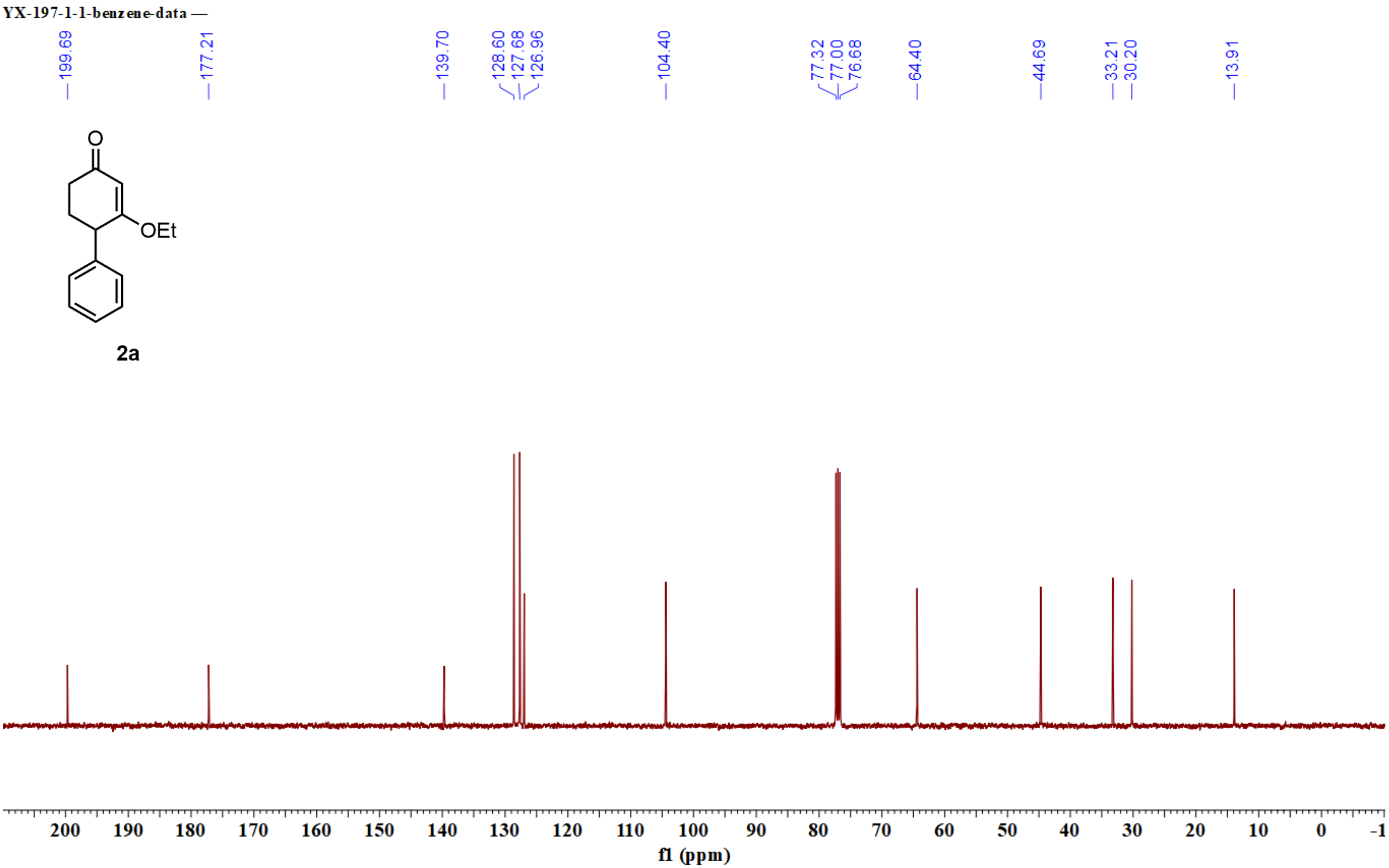

^{13}C NMR spectrum of compound 2a

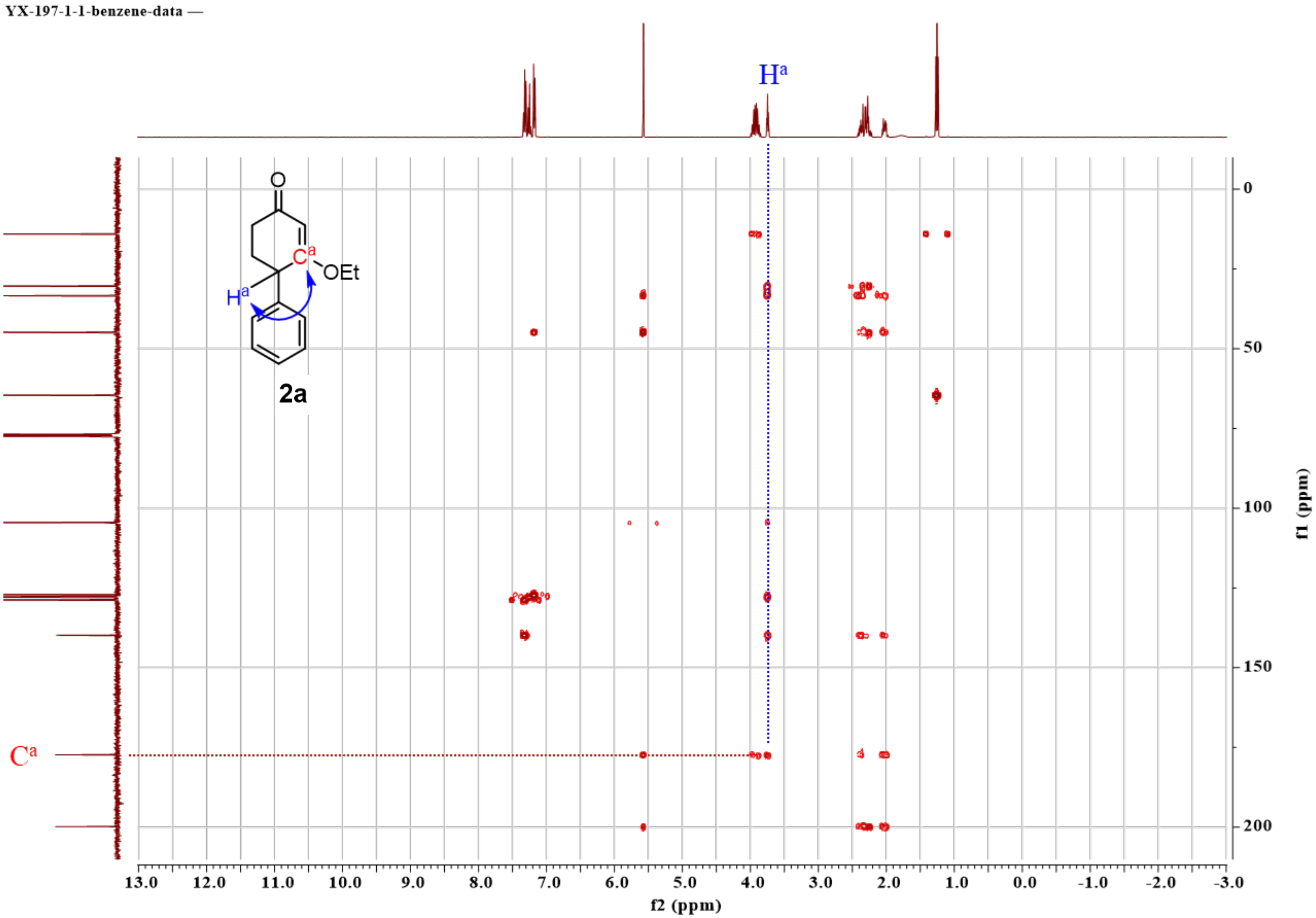

HMBC spectrum of compound 2a

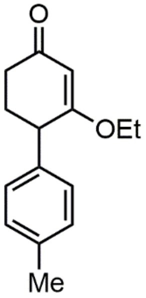

2b

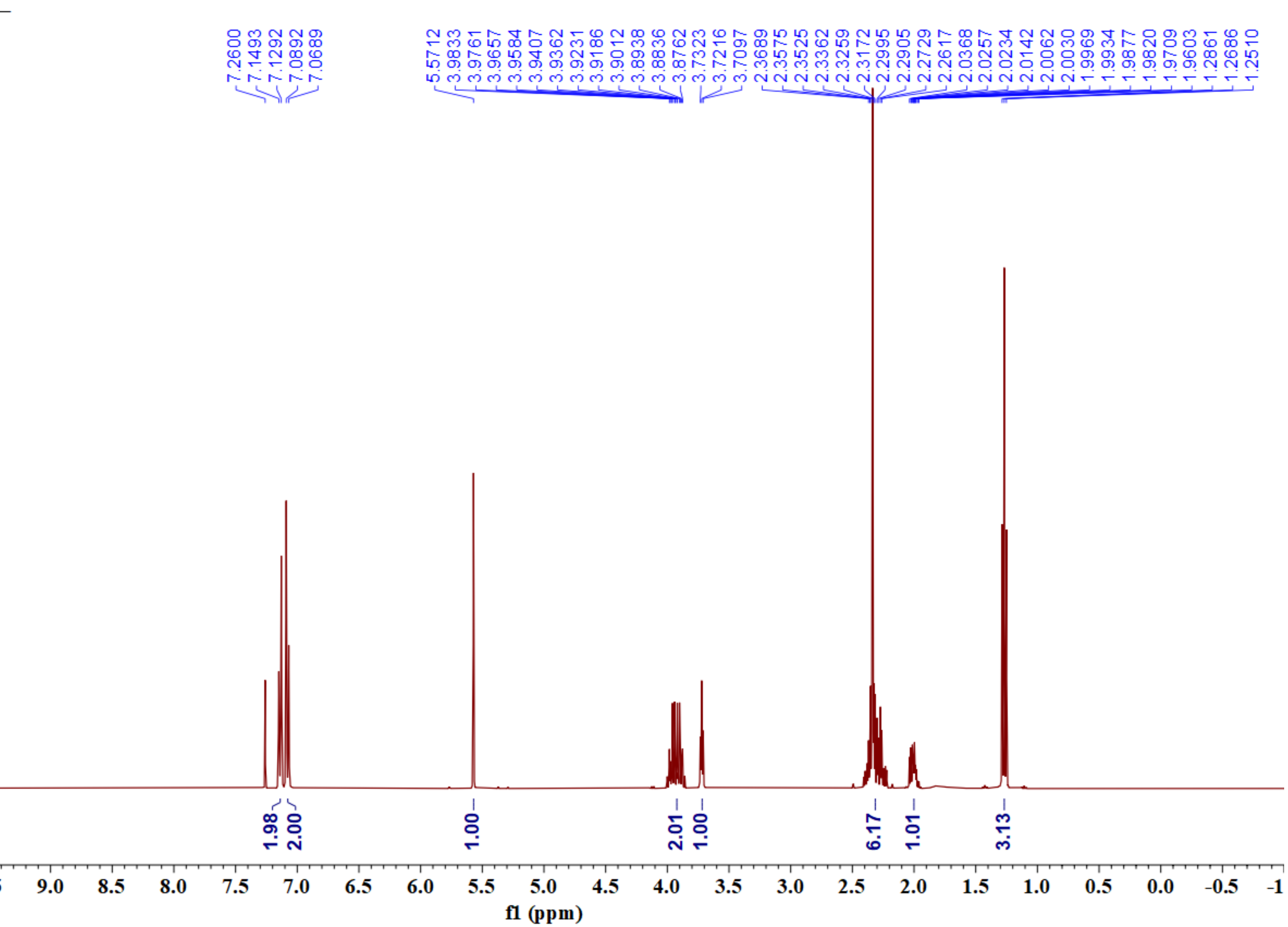

^{1}H NMR spectrum of compound 2b

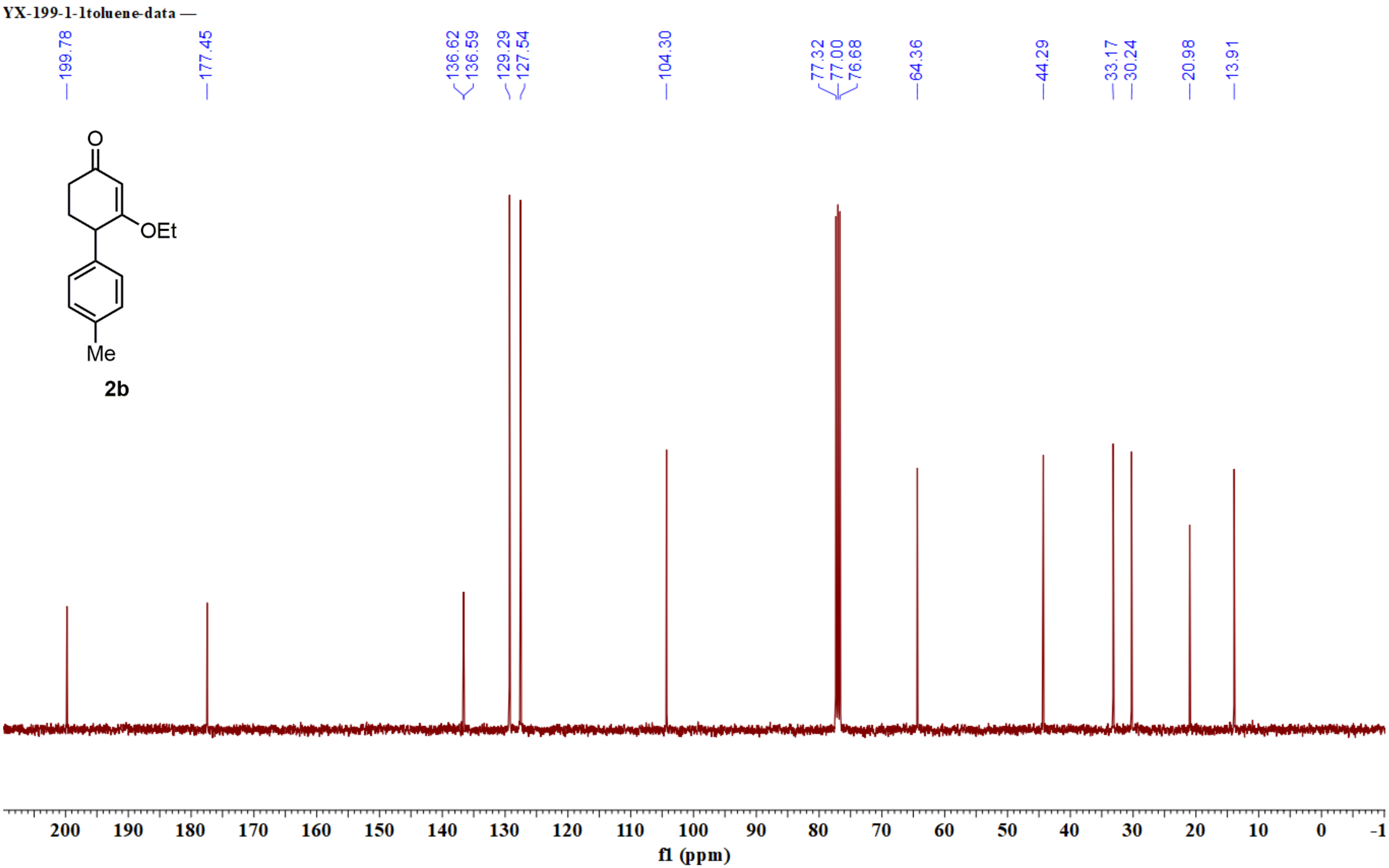

^{13}C NMR spectrum of compound 2b

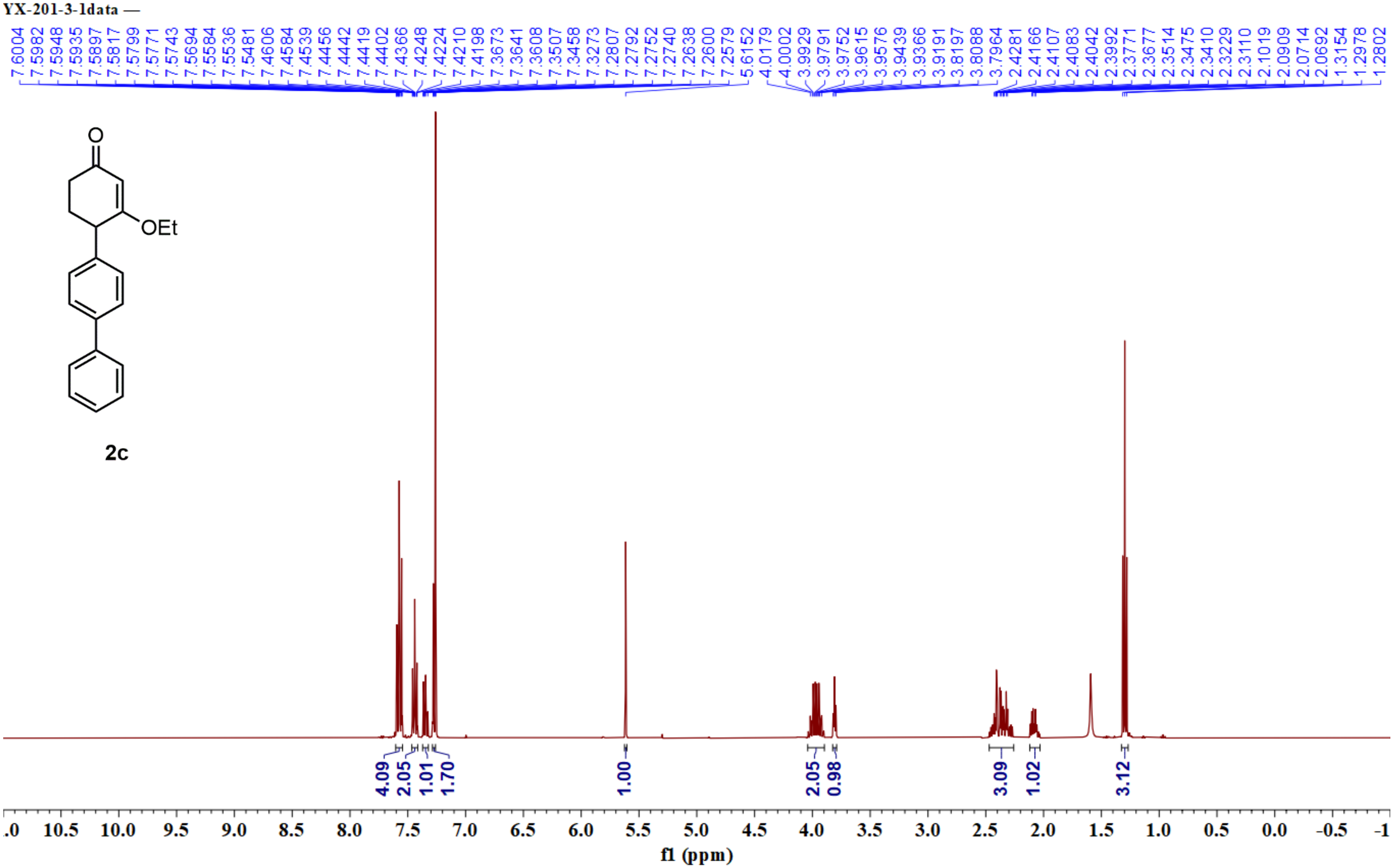

[1] H NMR spectrum of compound 177

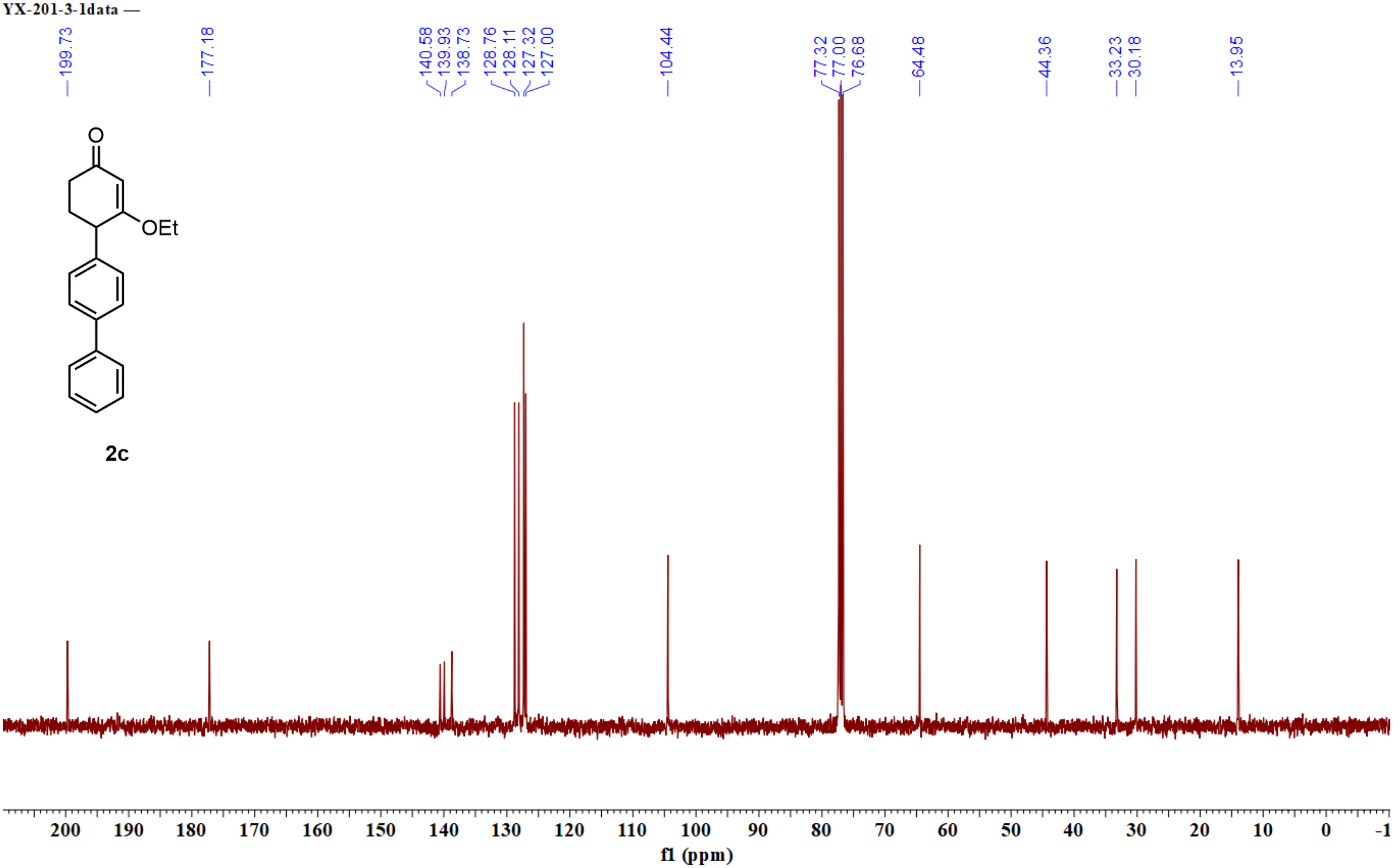

^{13}C NMR spectrum of compound 177

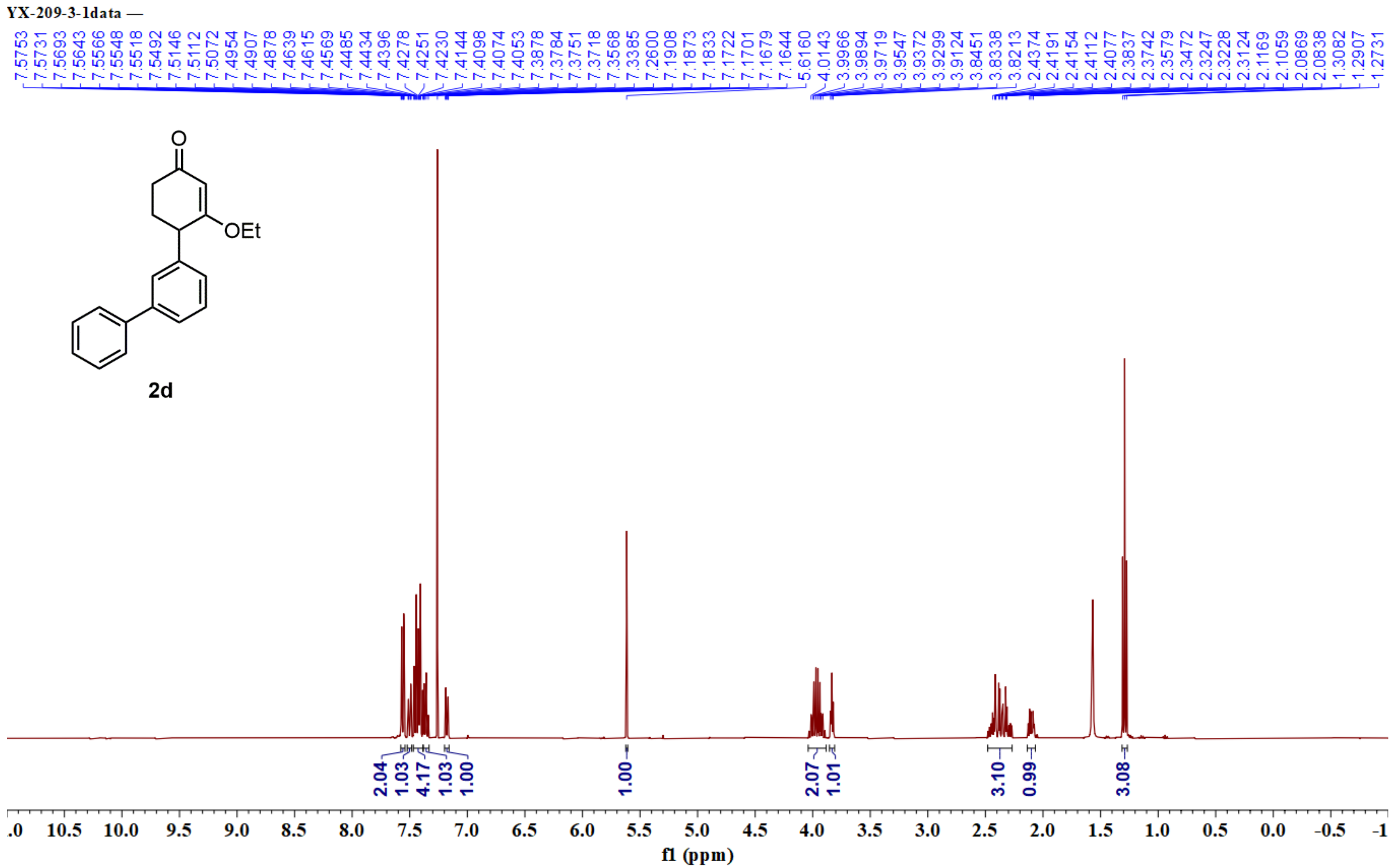

^{1}H NMR spectrum of compound 2d

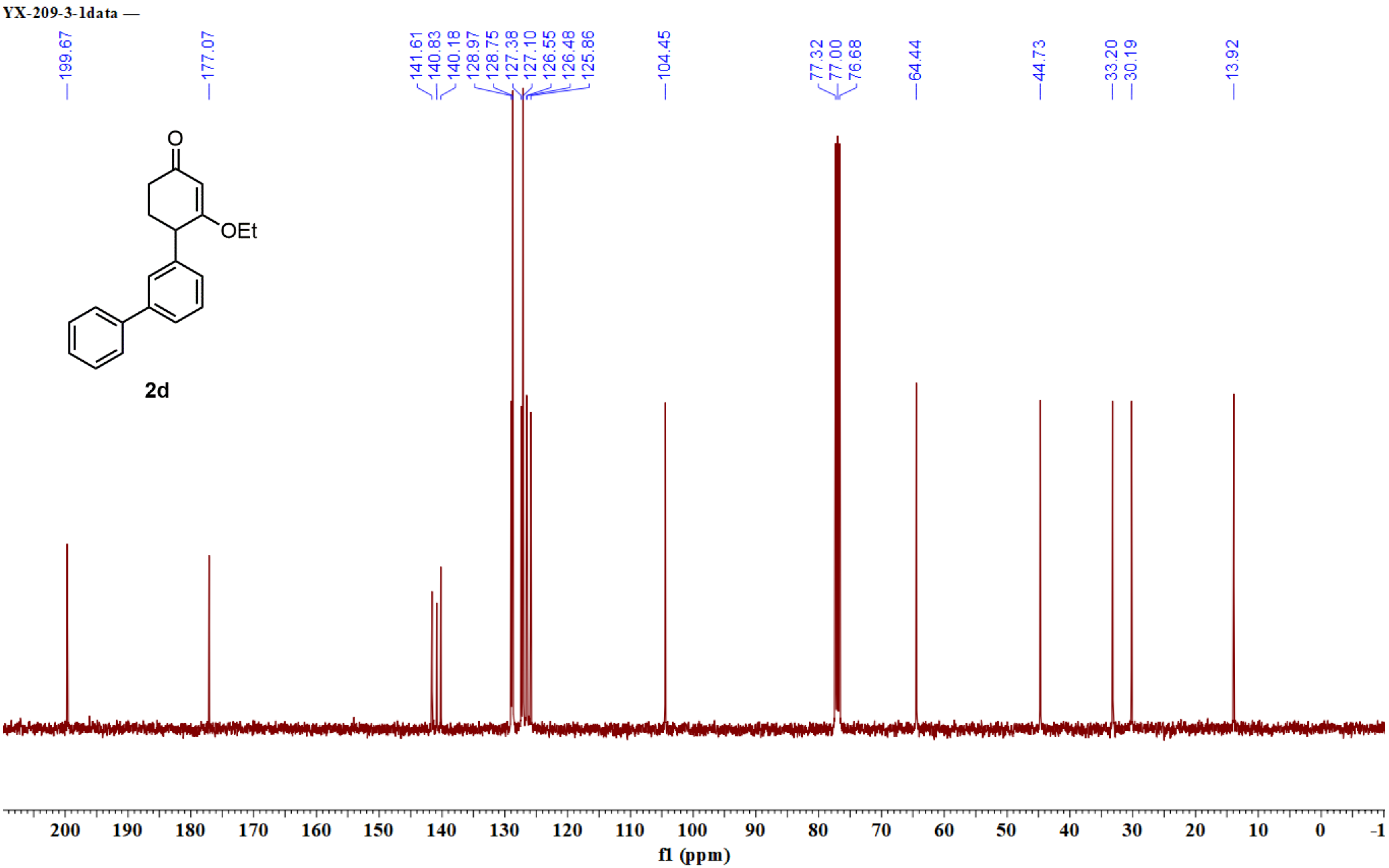

^{13}C NMR spectrum of compound 2d

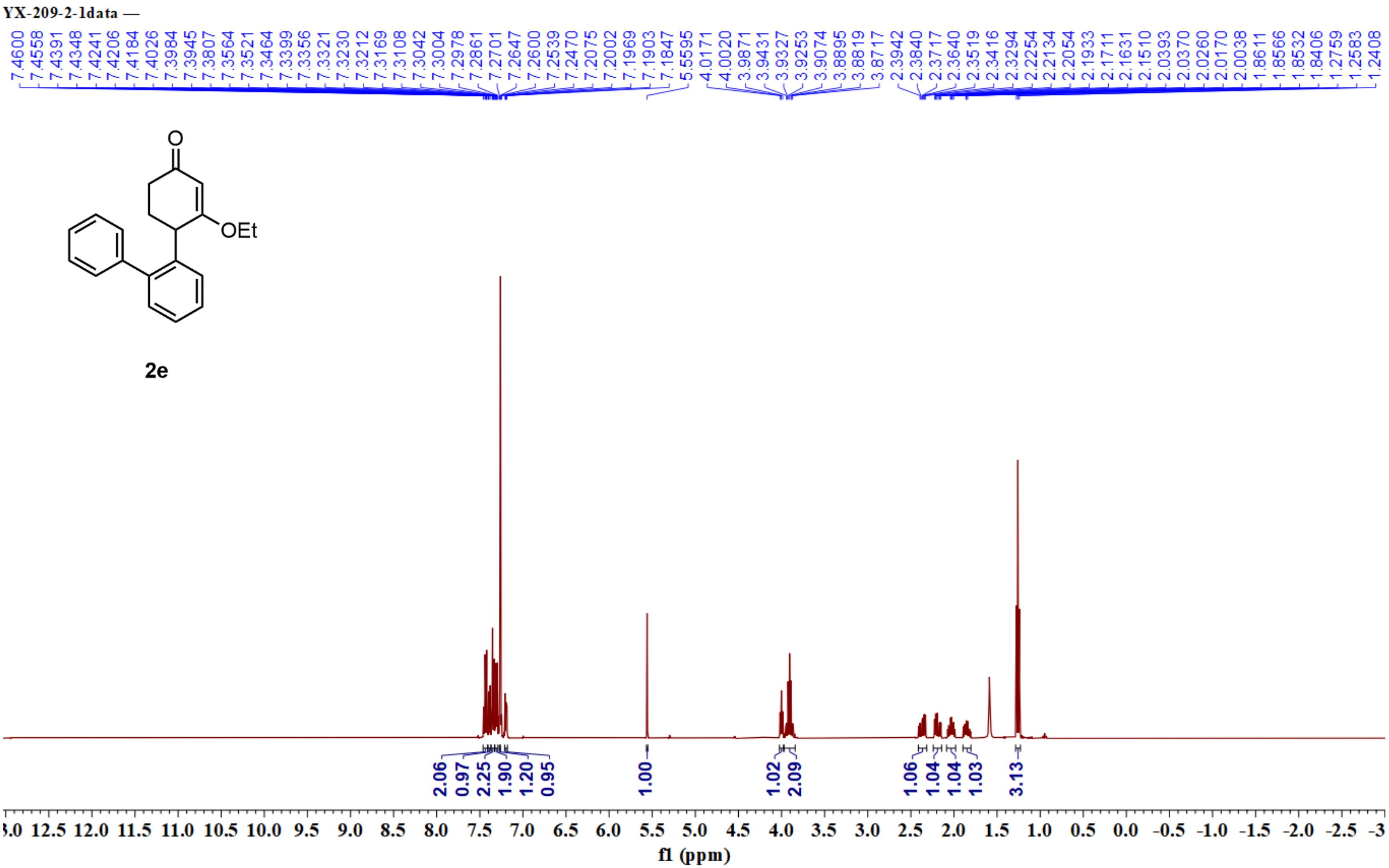

^{1}H NMR spectrum of compound 2e

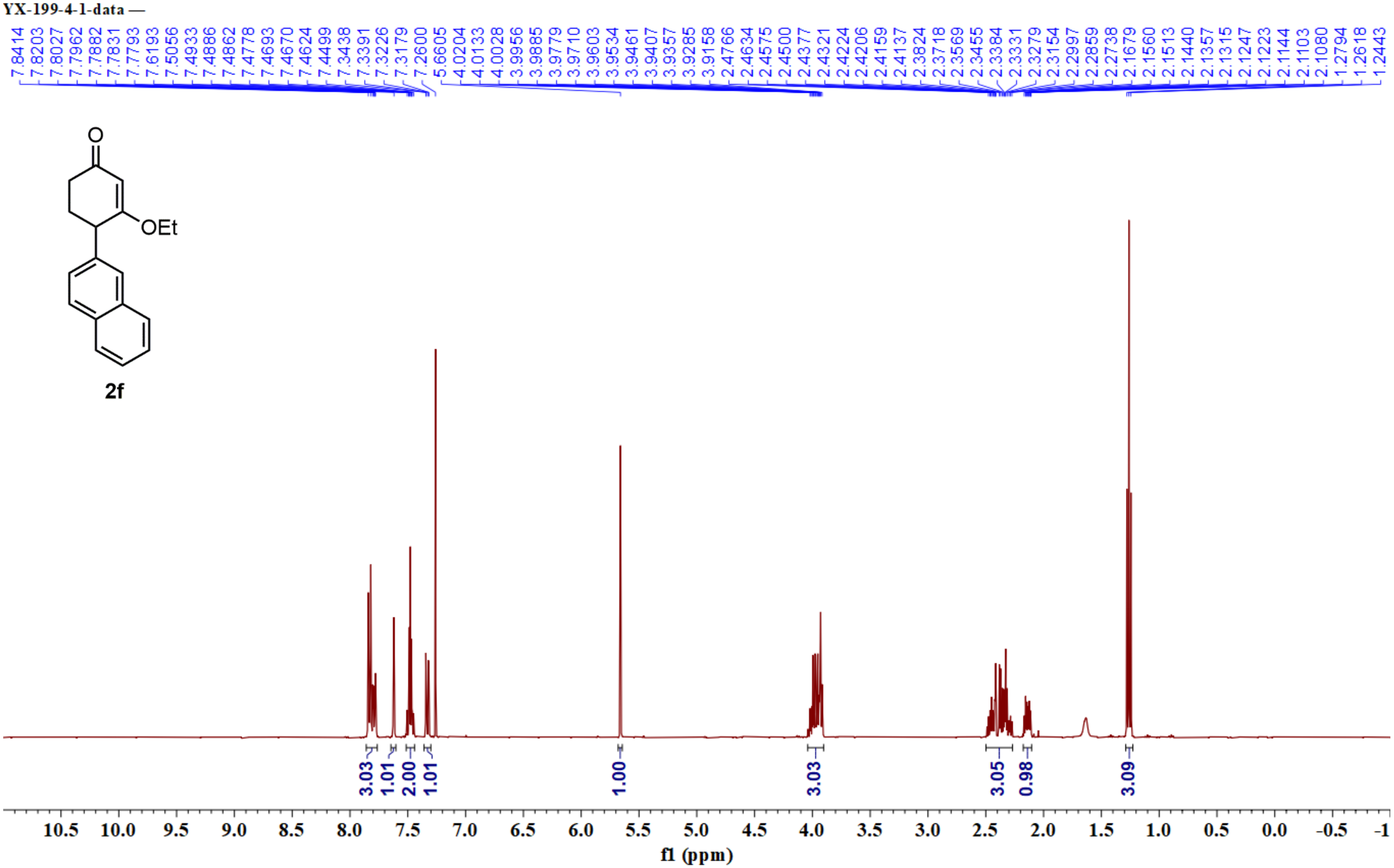

^{1}H NMR spectrum of compound 2f

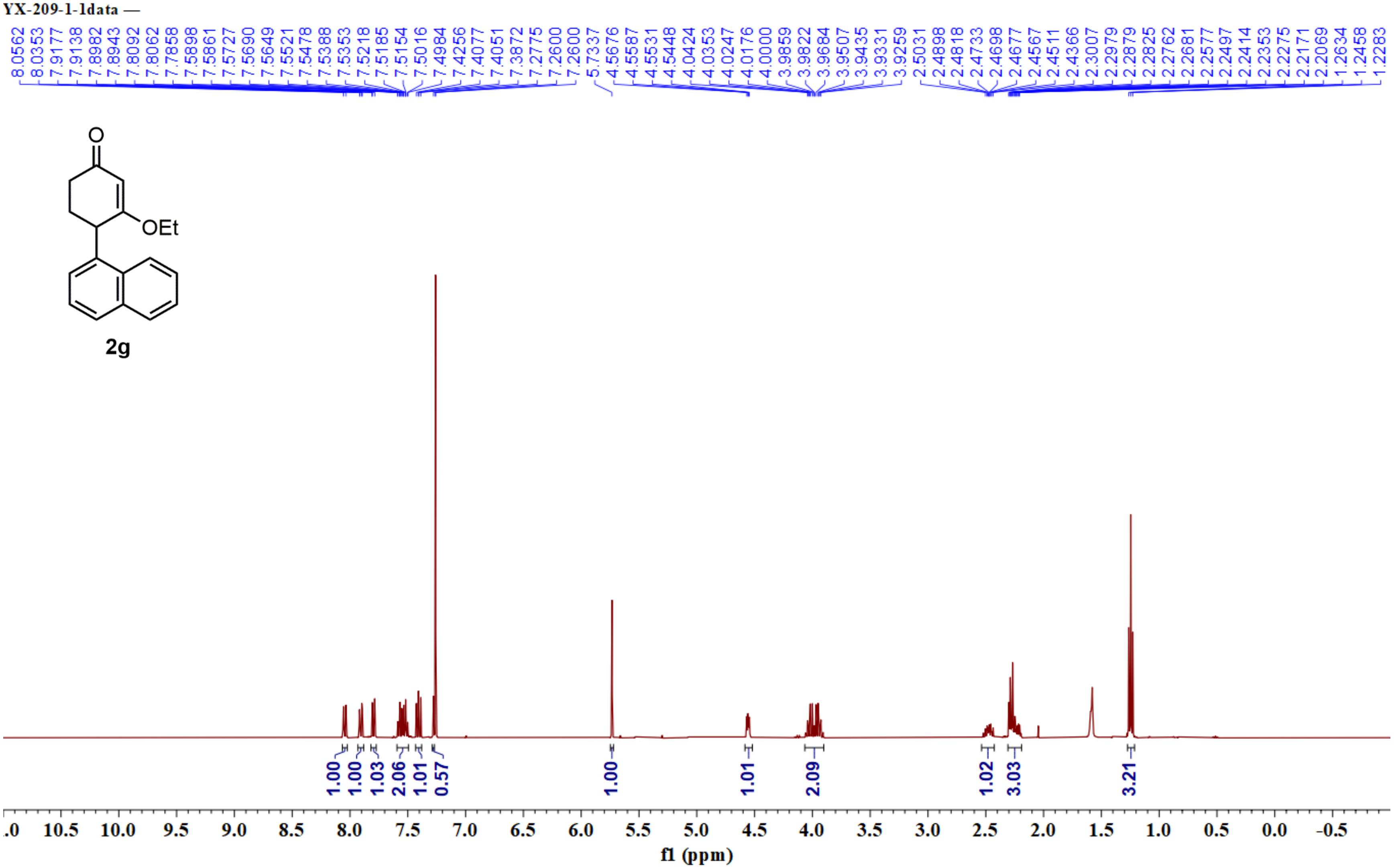

^{1}H NMR spectrum of compound 2g

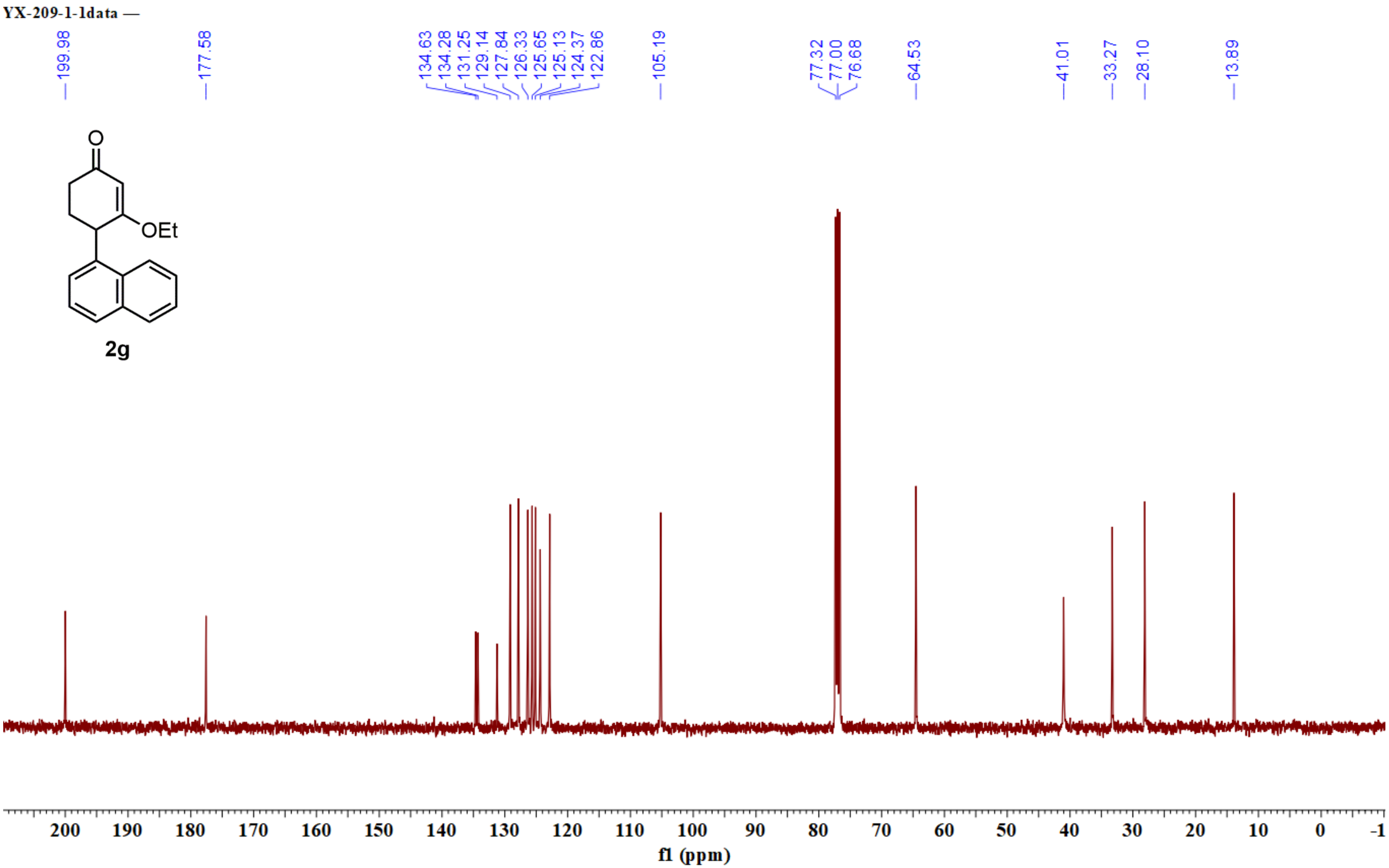

^{13}C NMR spectrum of compound 2g

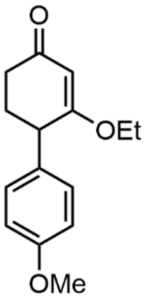

2h

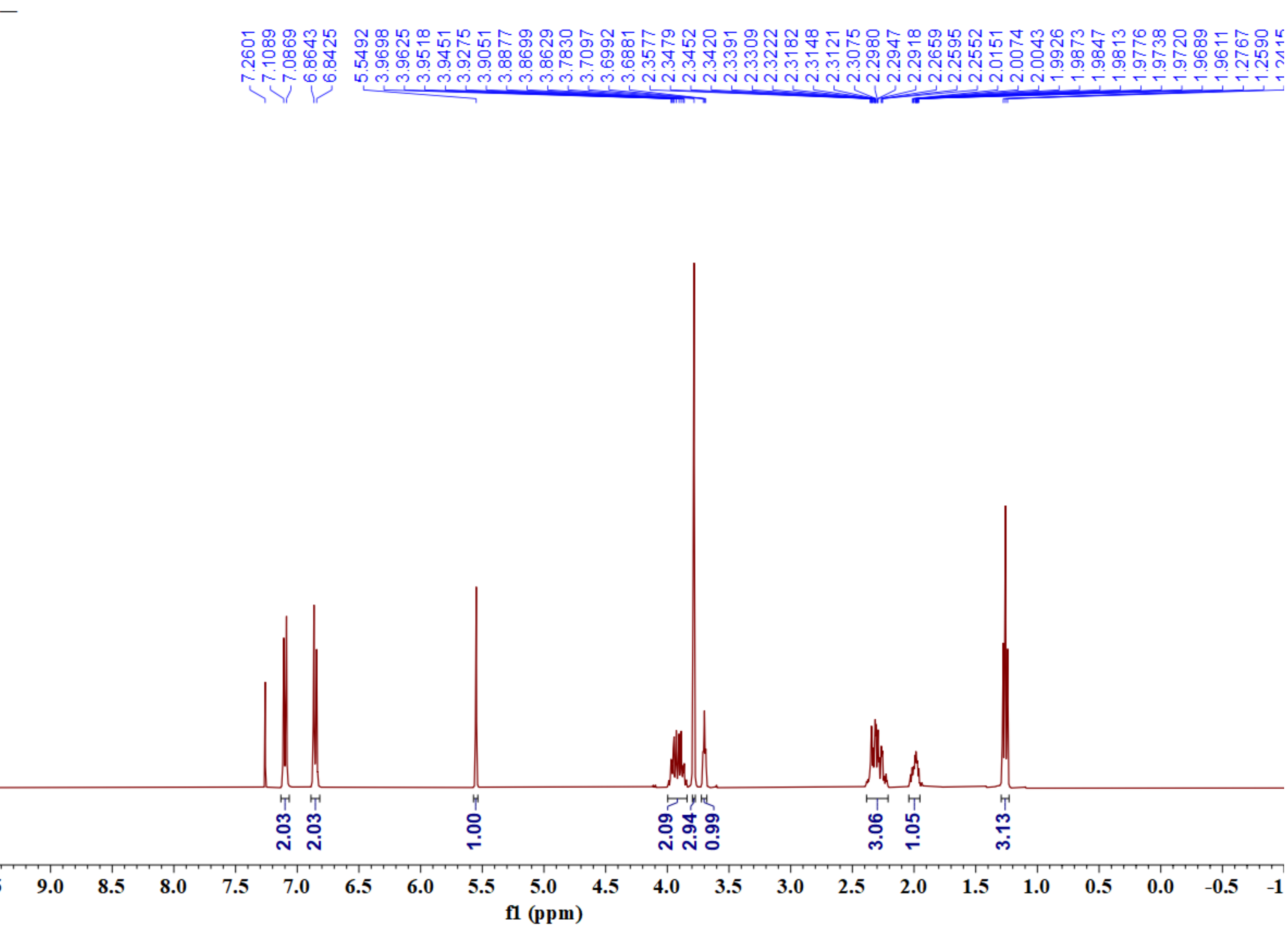

^{1}H NMR spectrum of compound 2h

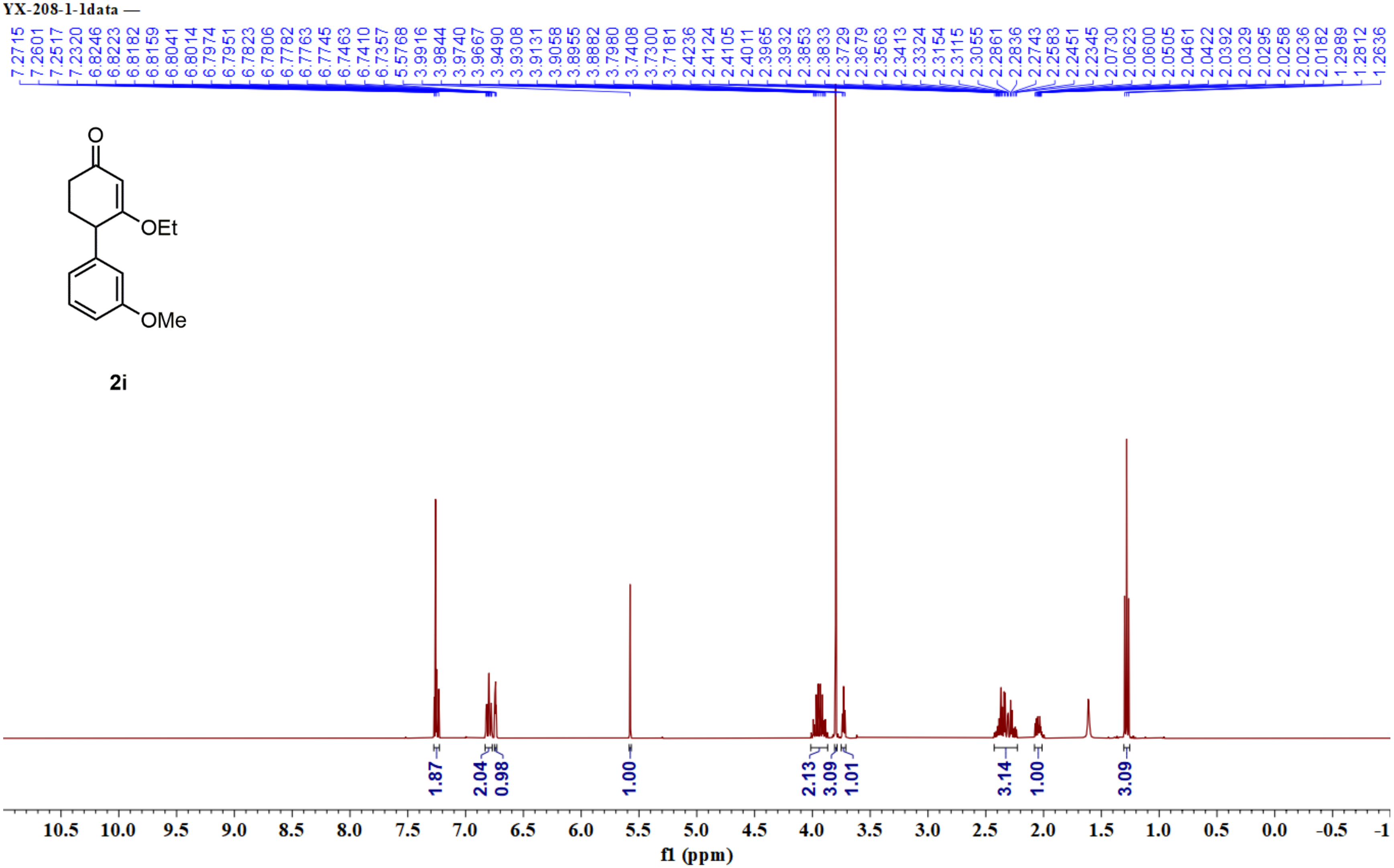

^{1}H NMR spectrum of compound 2i

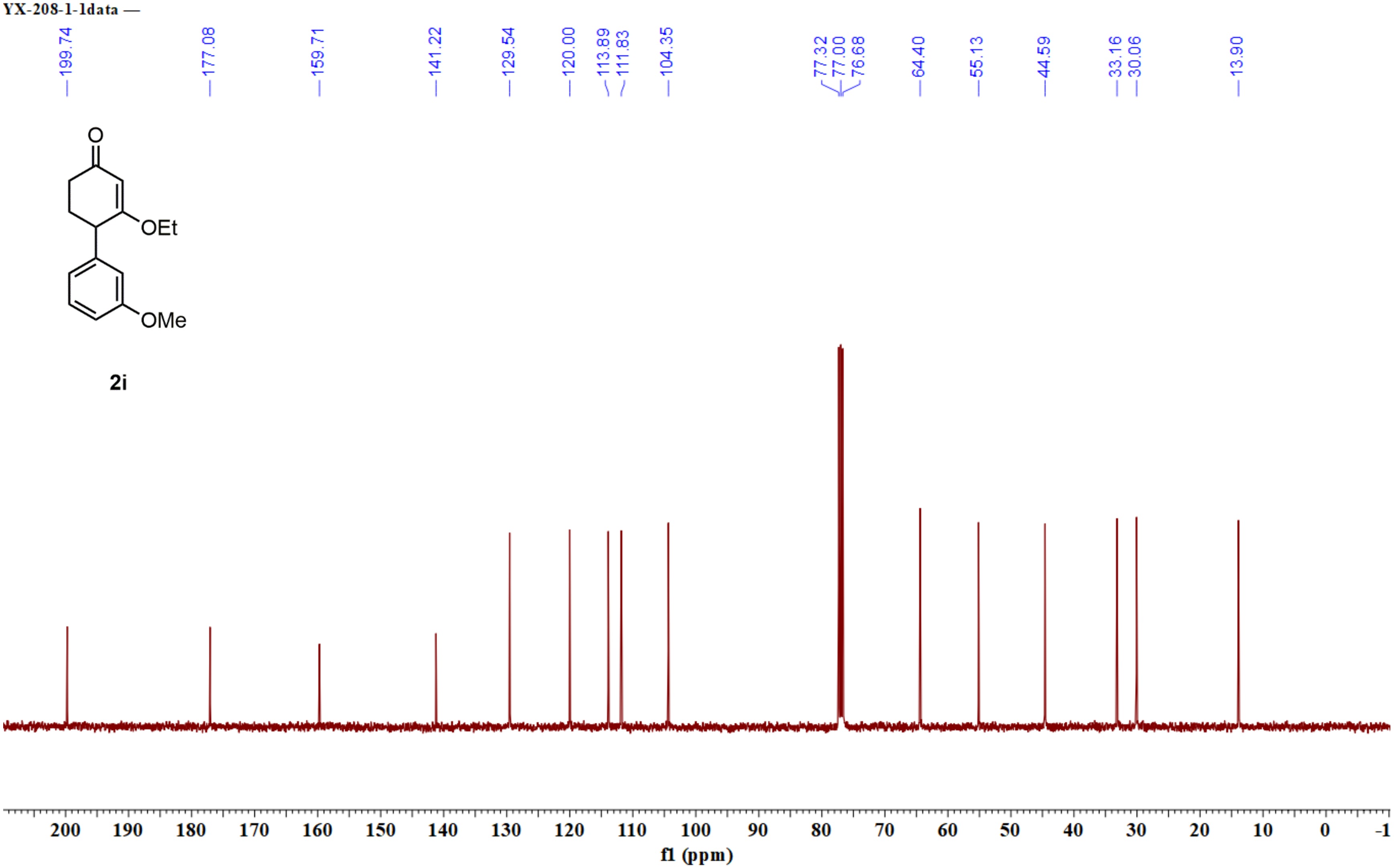

^{13}C NMR spectrum of compound 2i

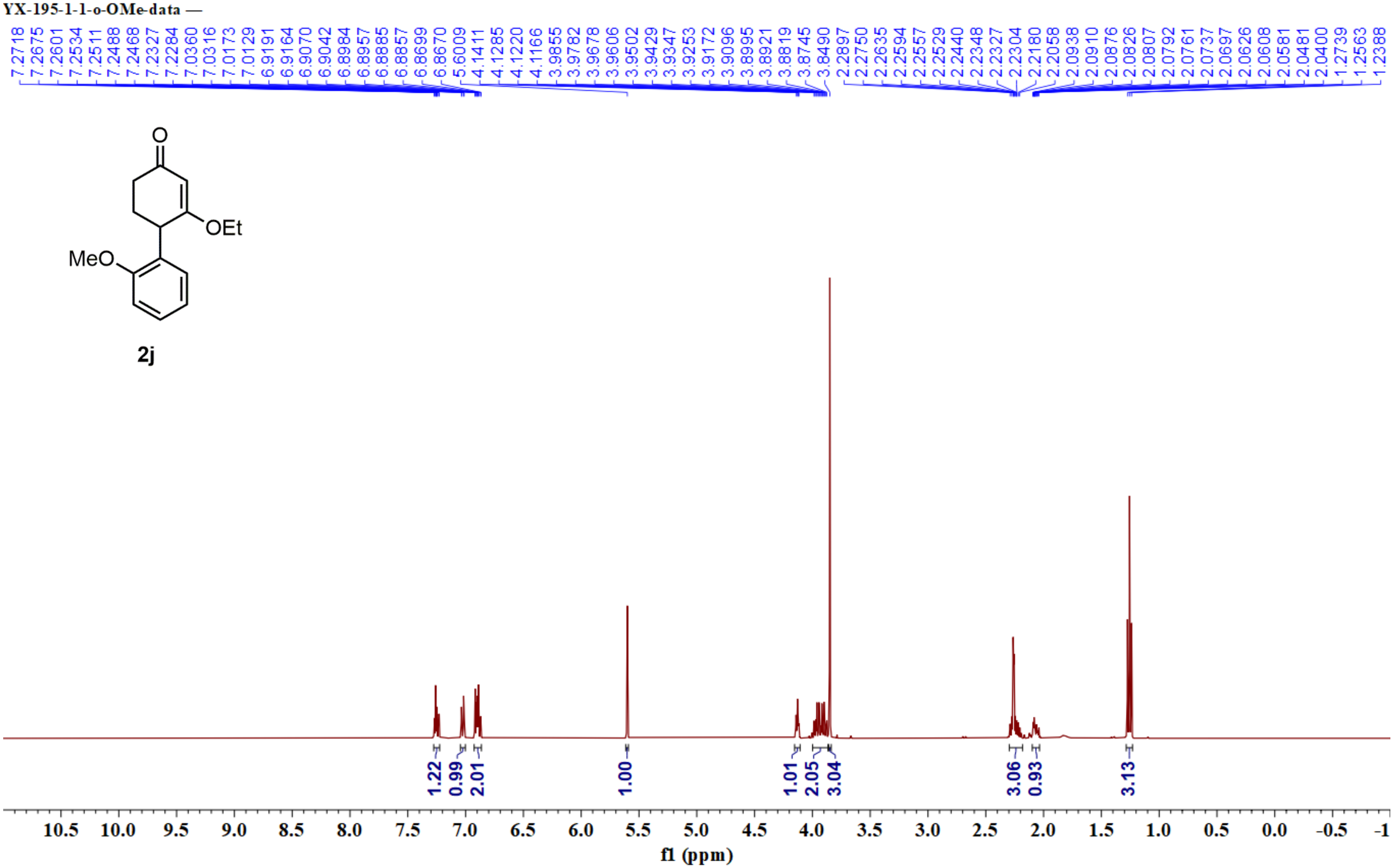

^{1}H NMR spectrum of compound 2j

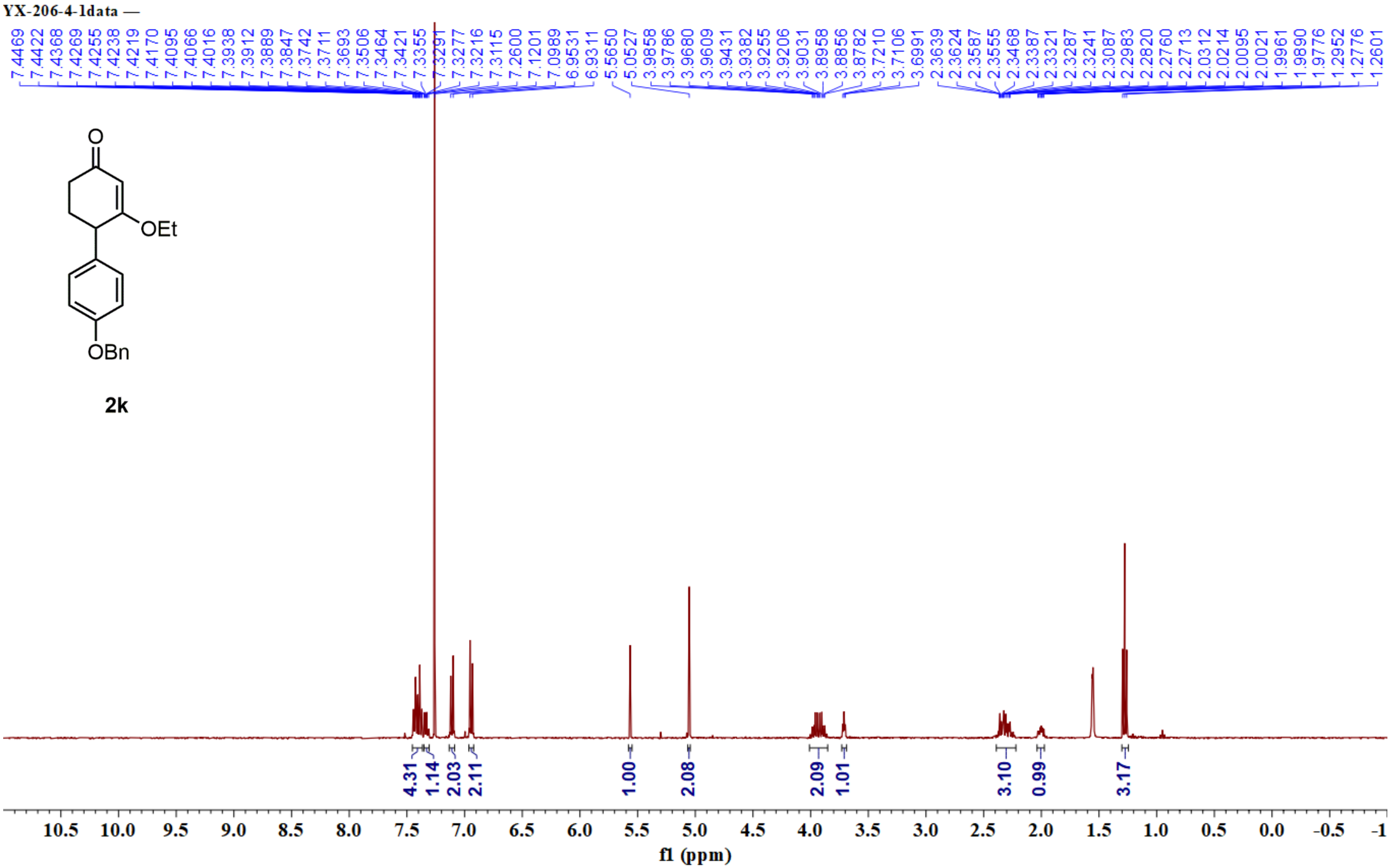

^{1}H NMR spectrum of compound 2k

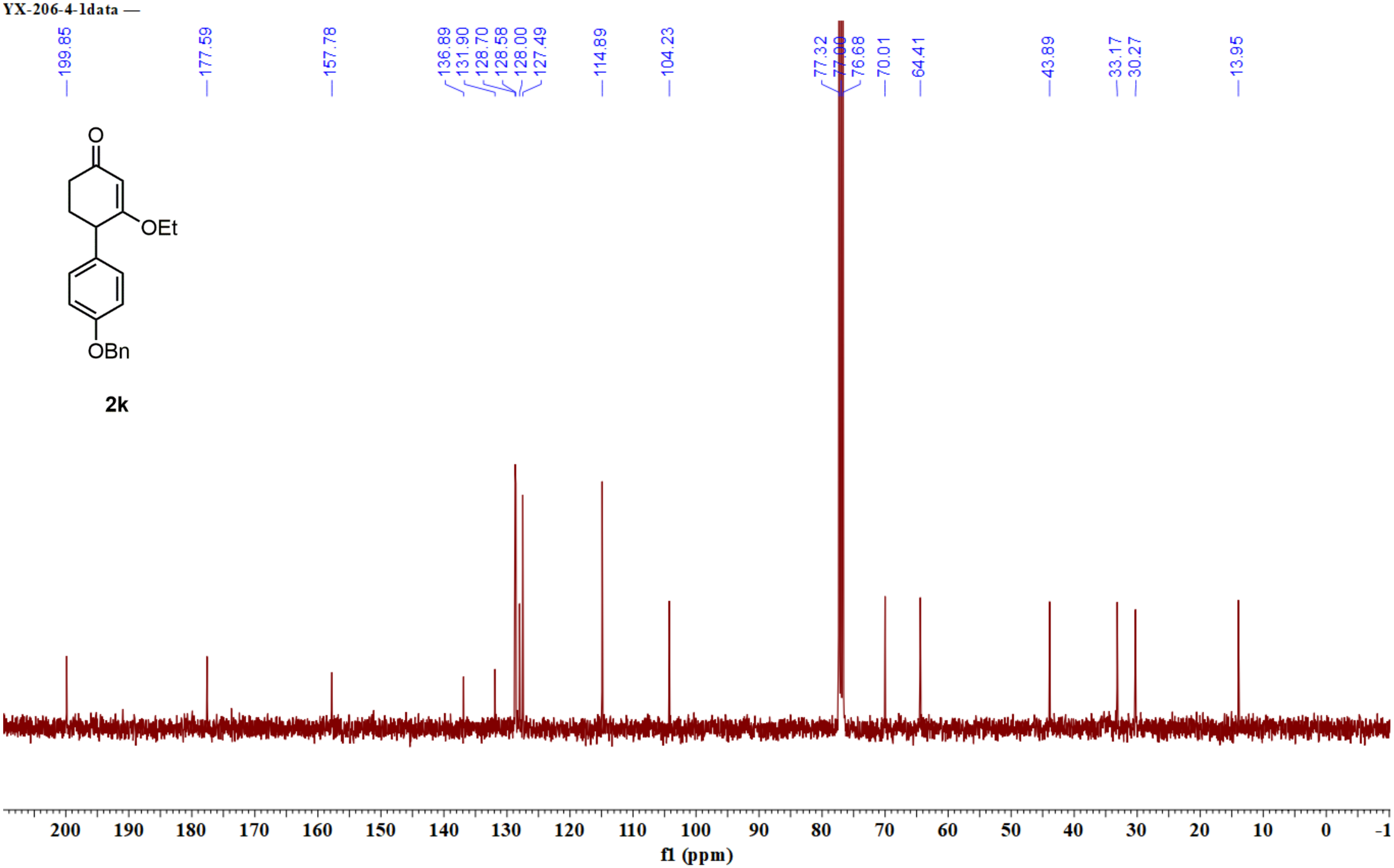

^{13}C NMR spectrum of compound 2k

YX-206-3-1data —

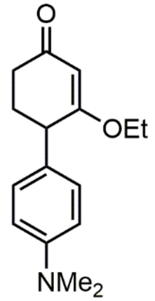

2l

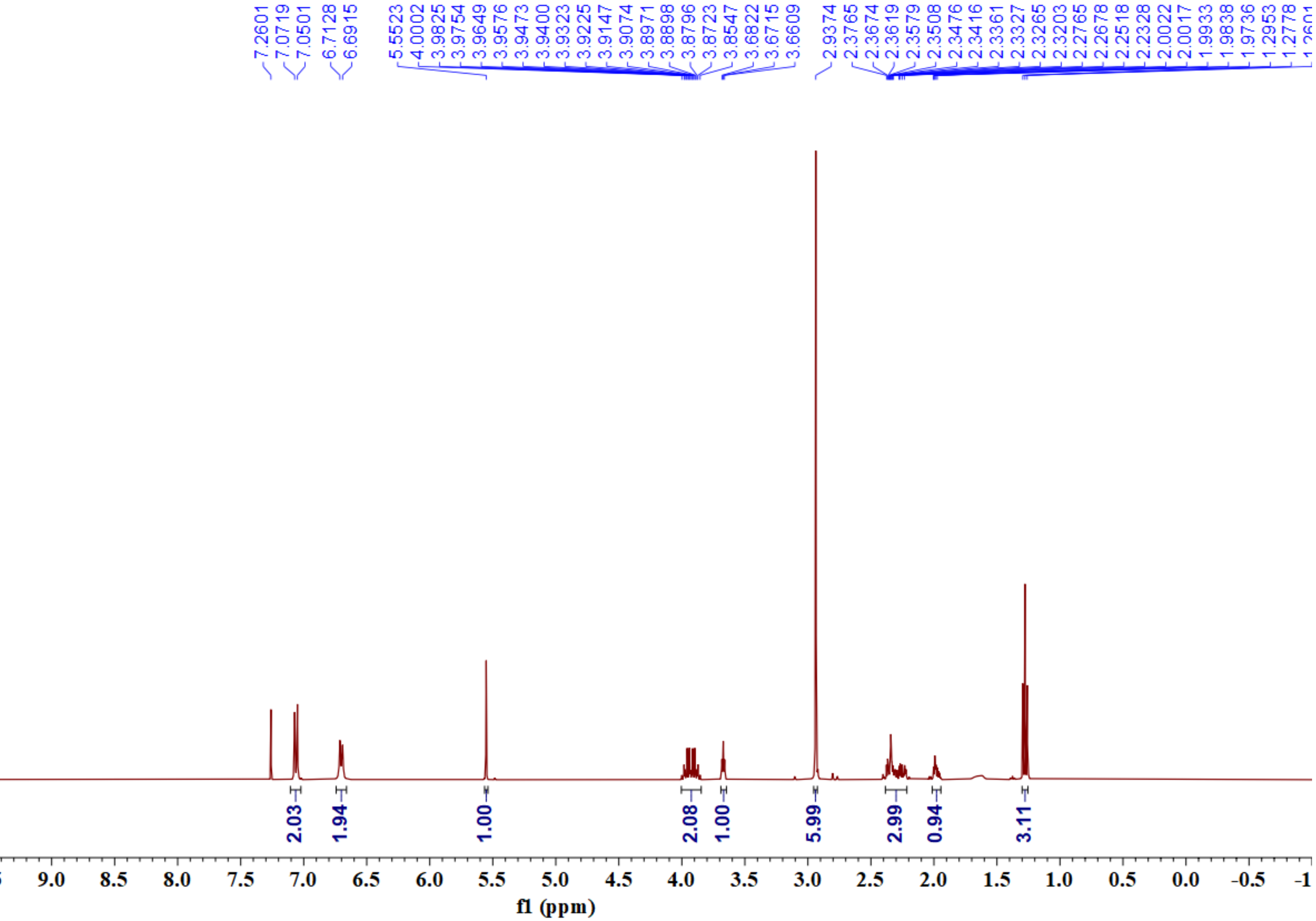

^{1}H NMR spectrum of compound 2l

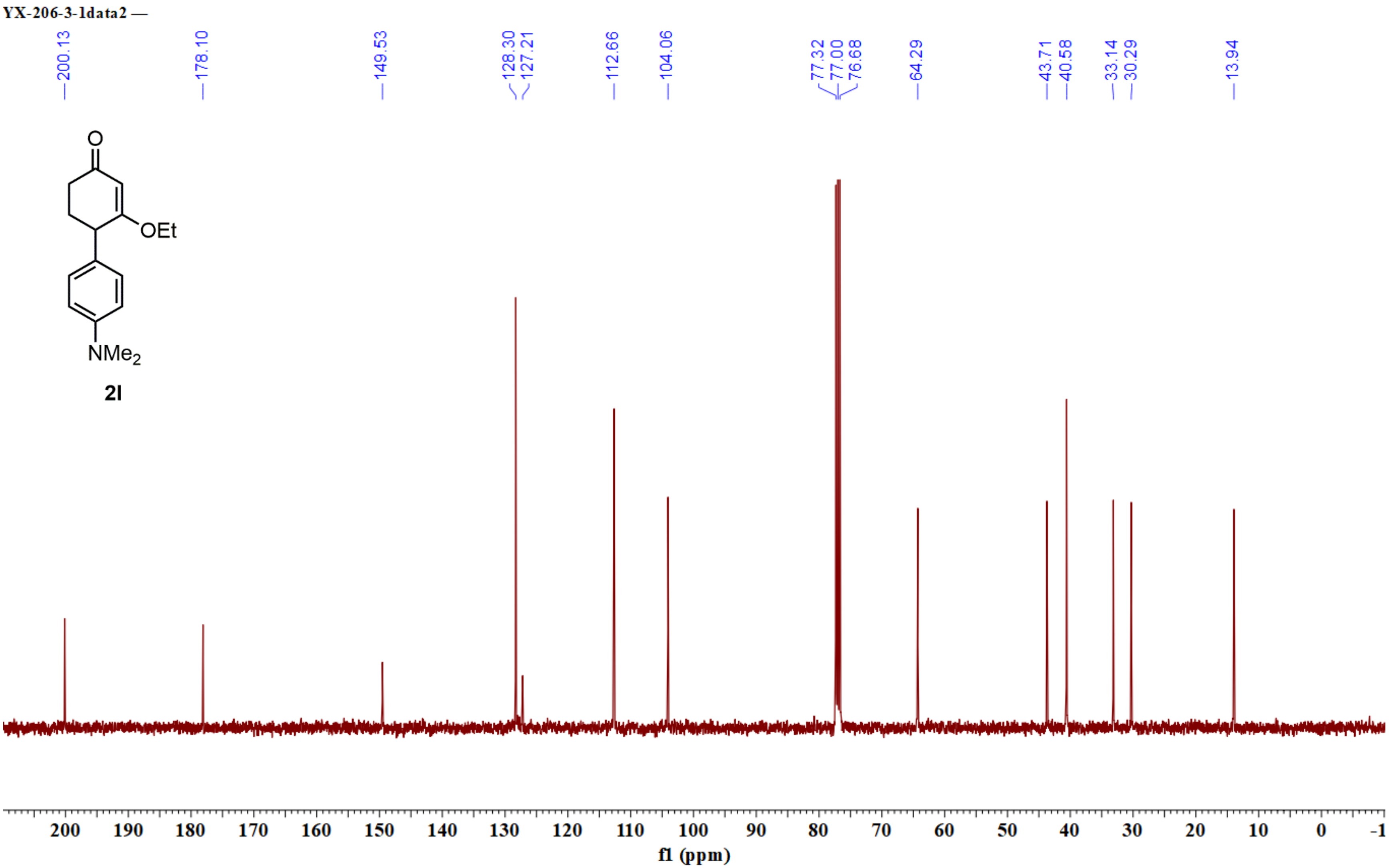

^{13}C NMR spectrum of compound 2l

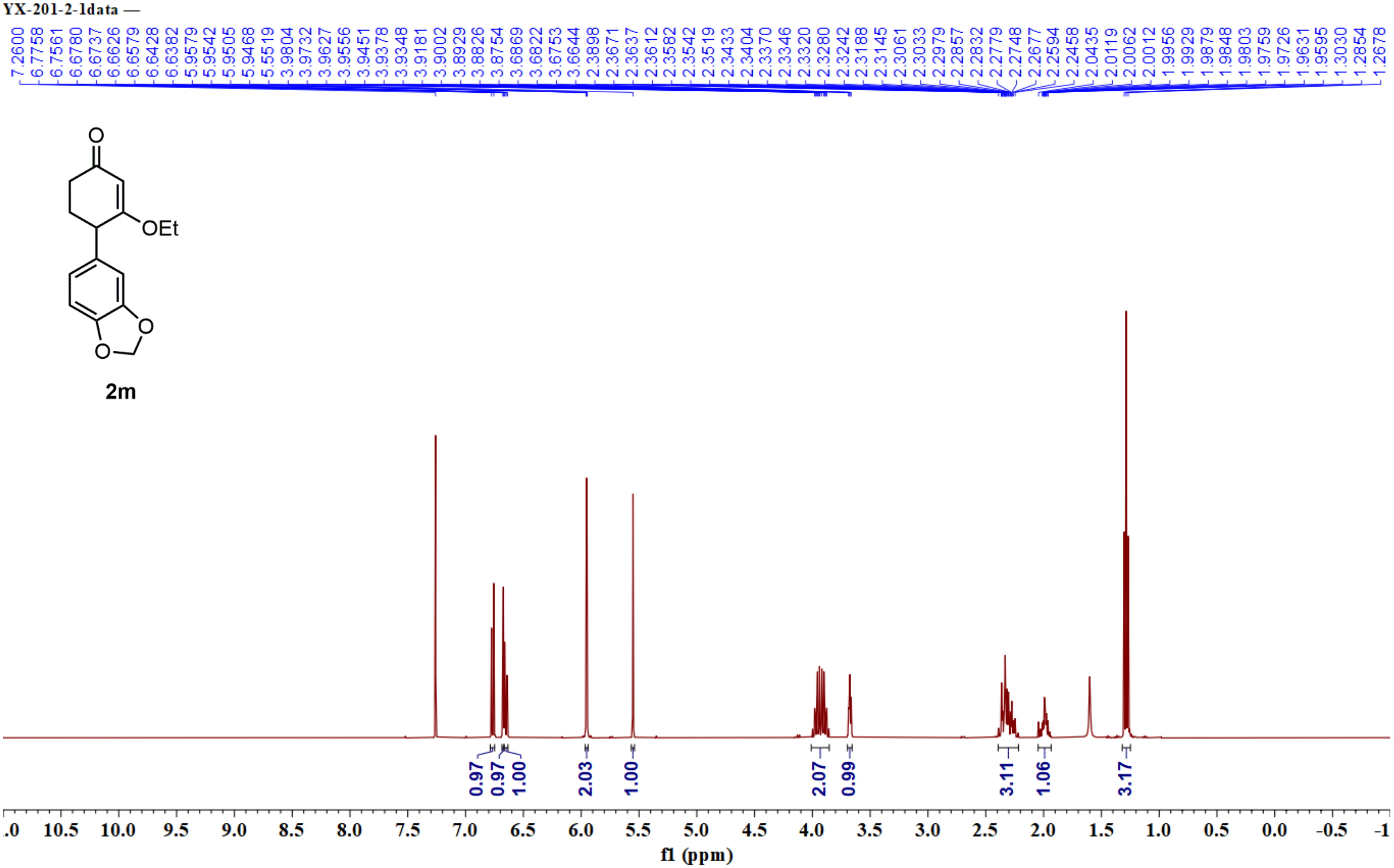

^{1}H NMR spectrum of compound 2m

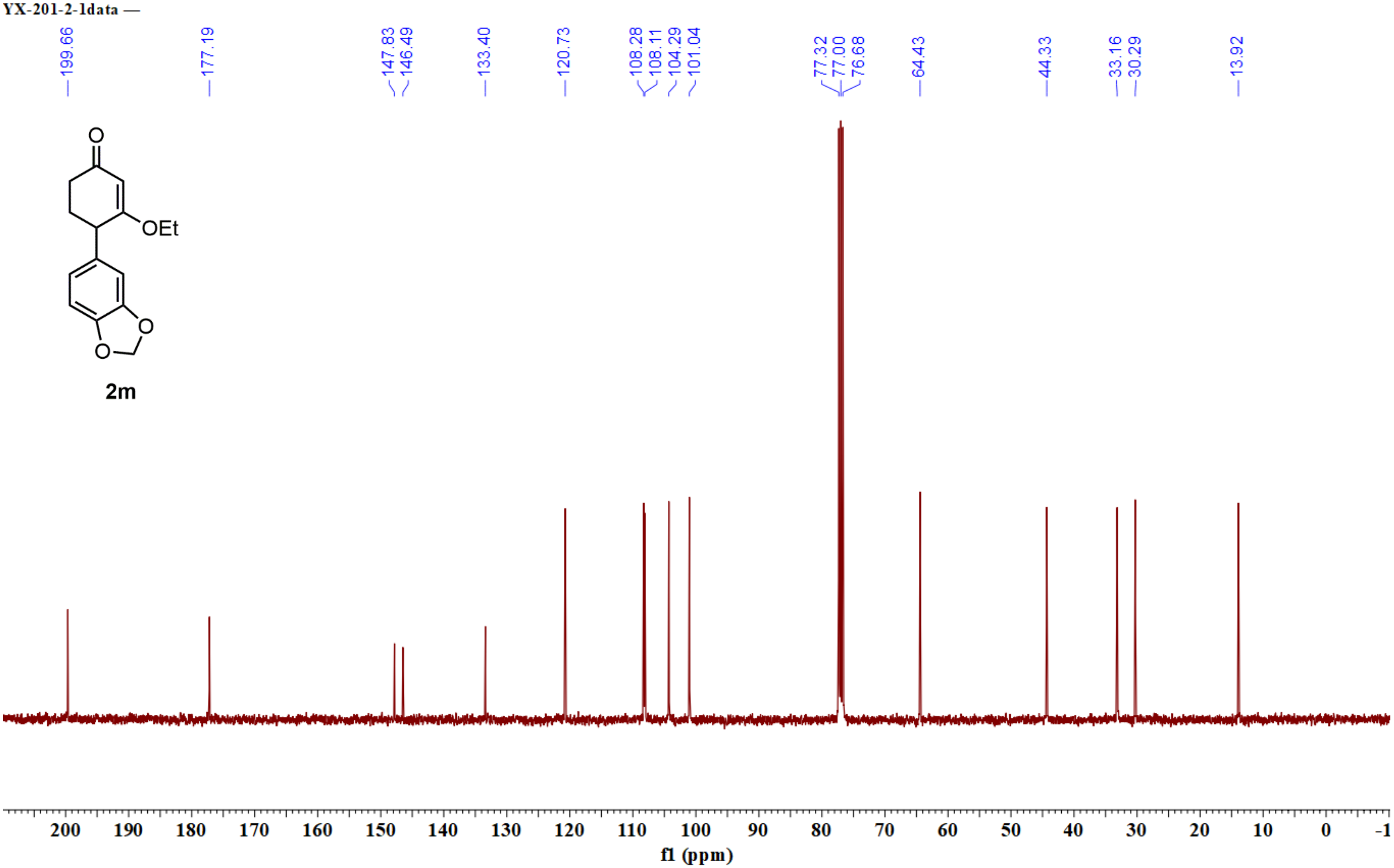

^{13}C NMR spectrum of compound 2m

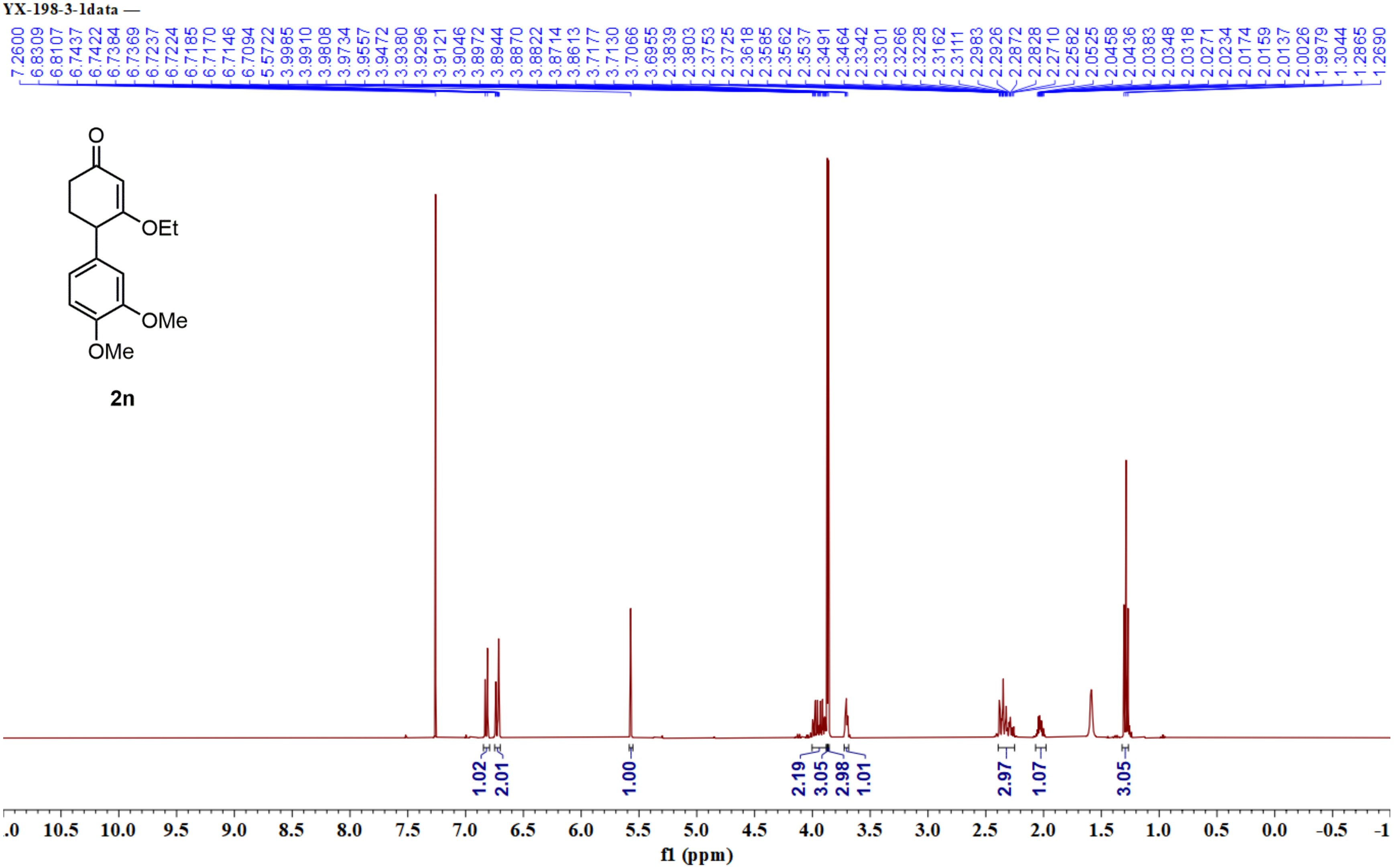

^{1}H NMR spectrum of compound 2n

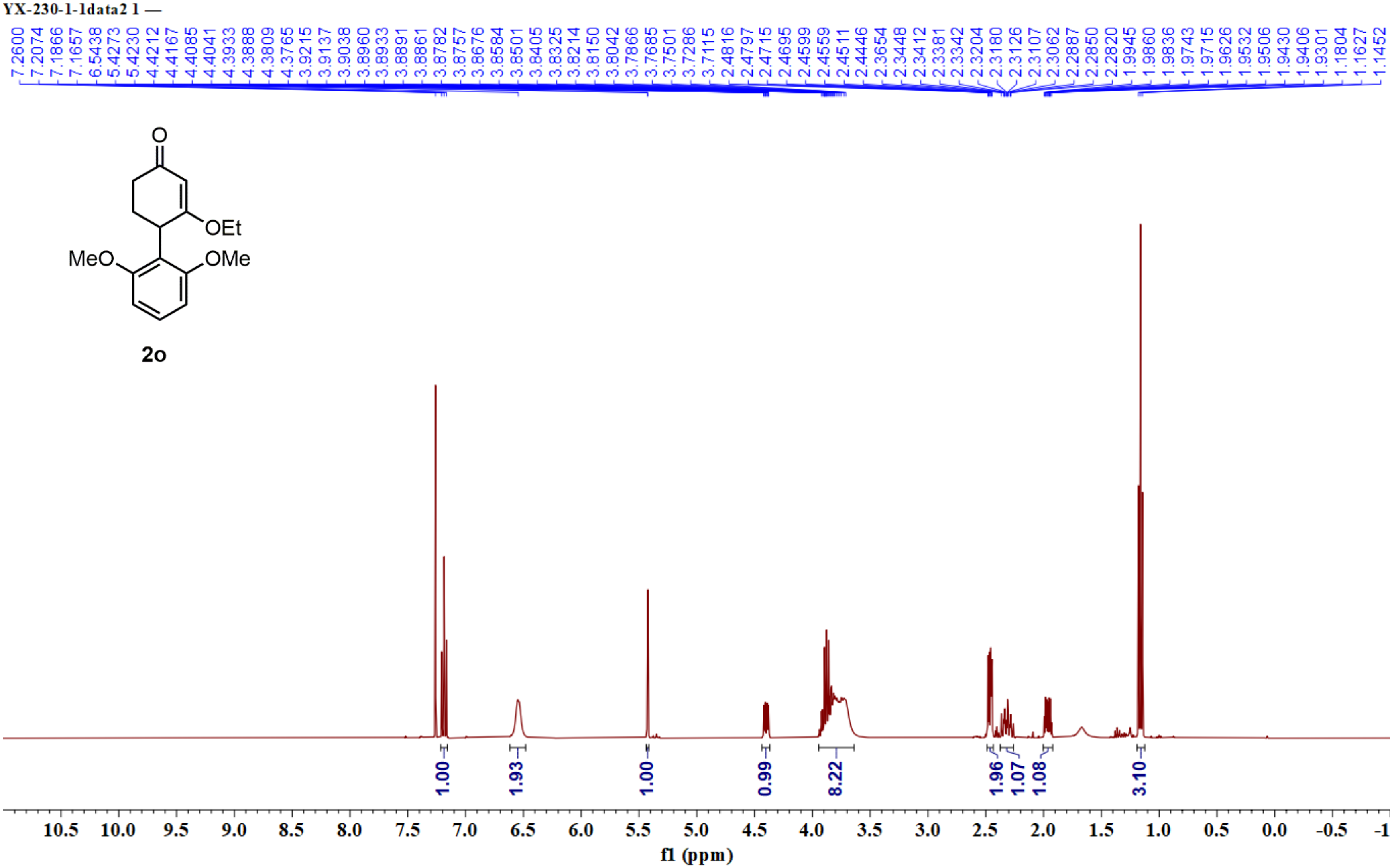

^{1}H NMR spectrum of compound 2o

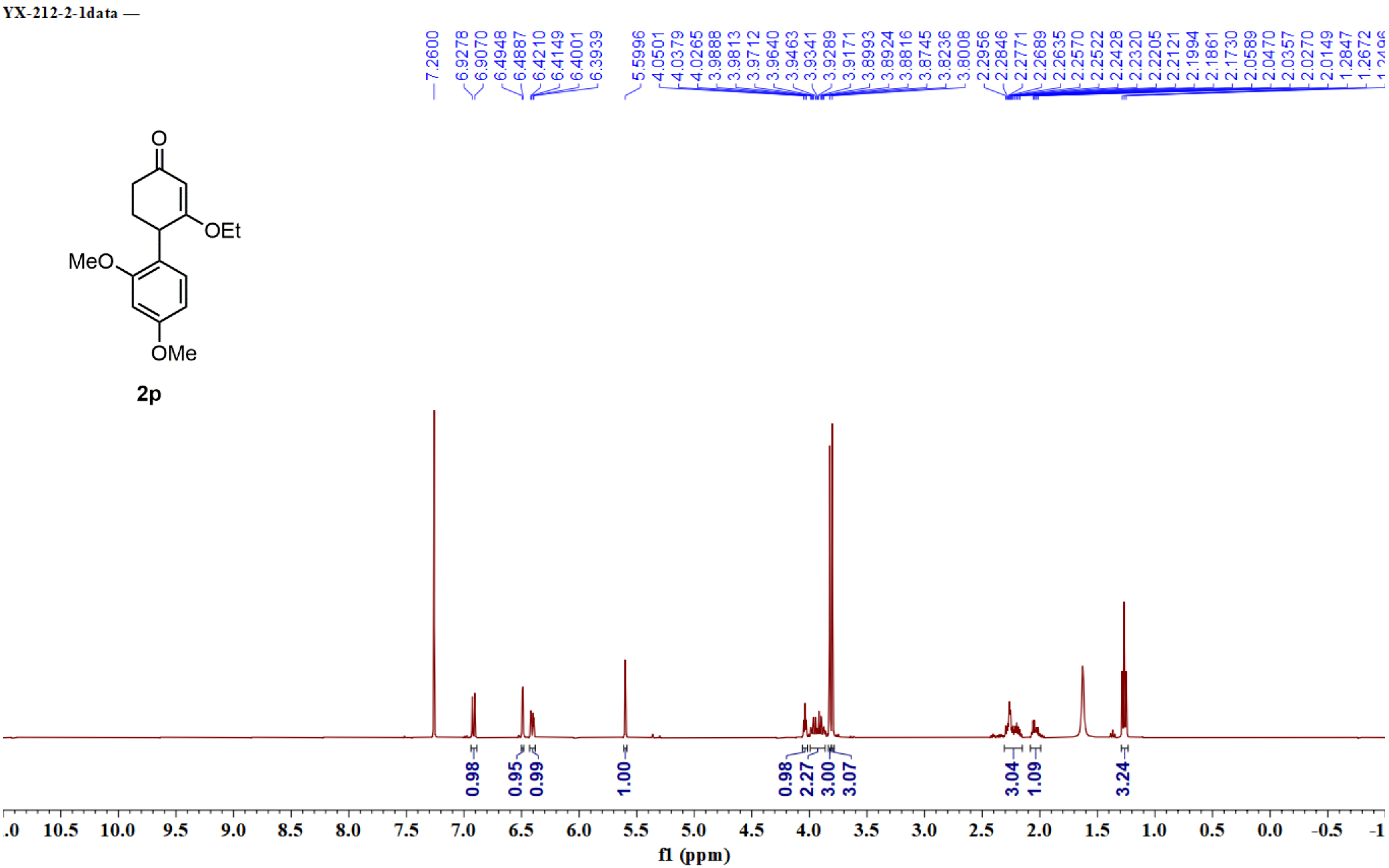

^{1}H NMR spectrum of compound 2p

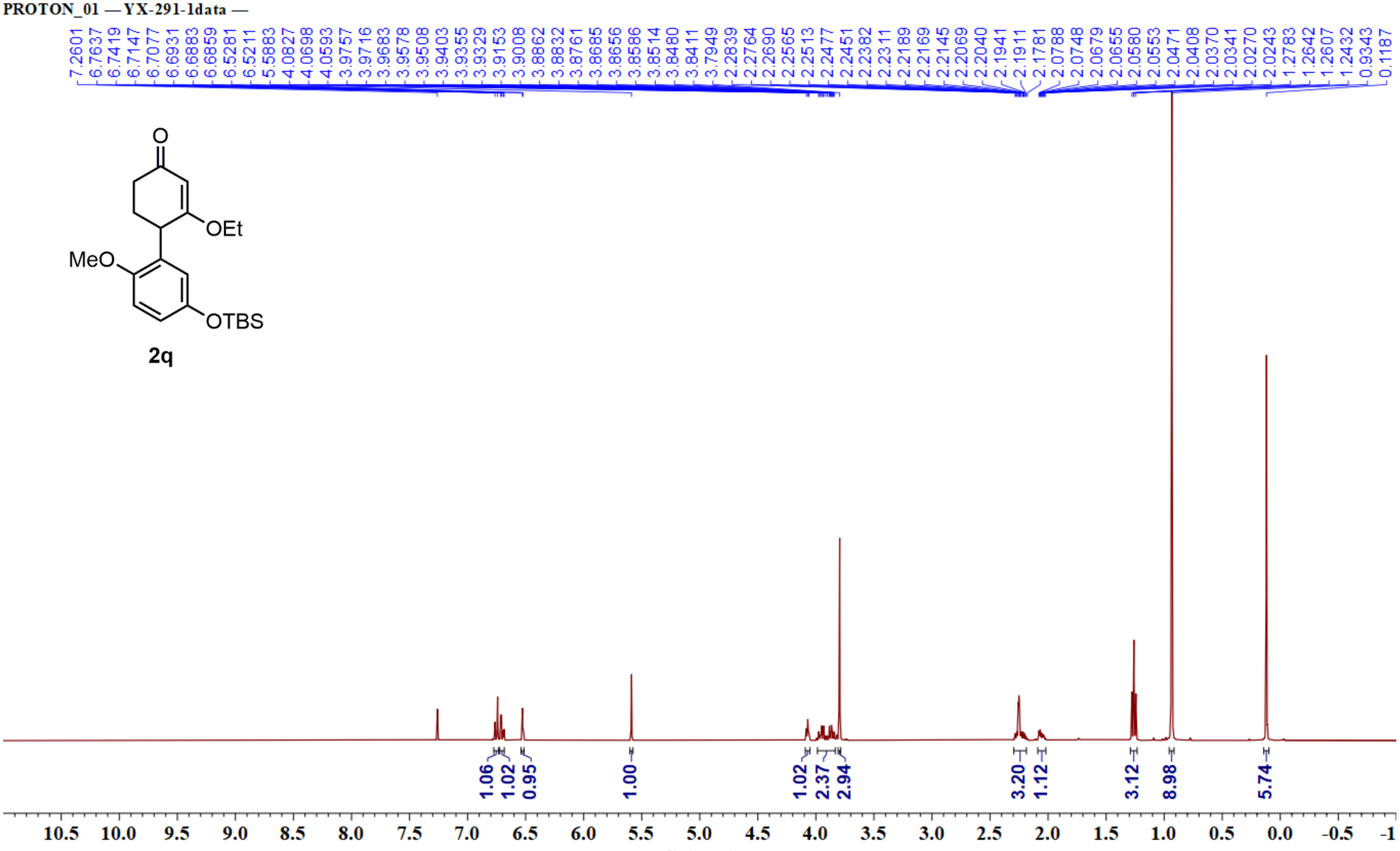

^{1}H NMR spectrum of compound 2q

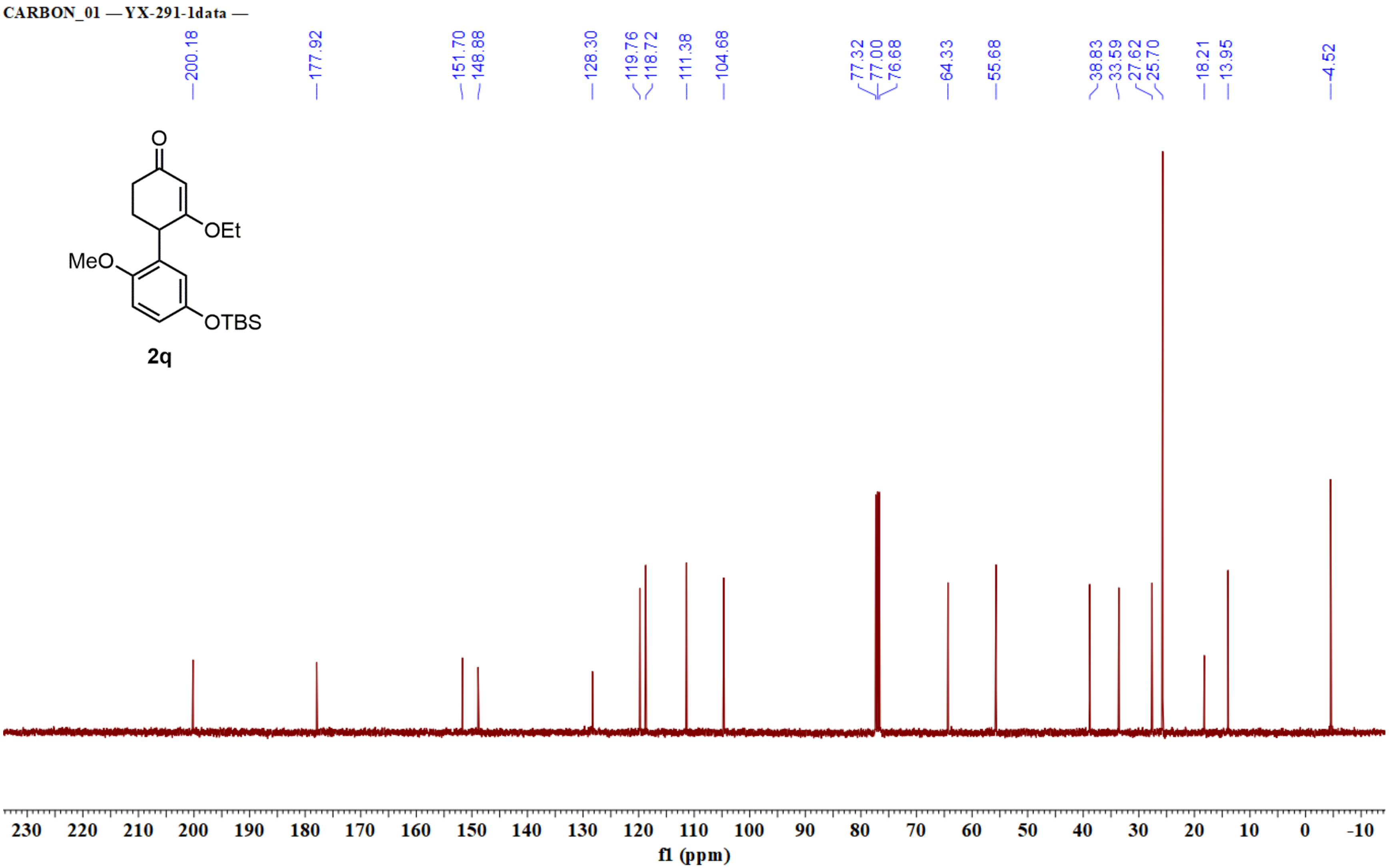

^{13}C NMR spectrum of compound 2q

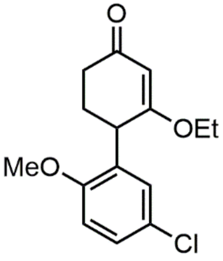

2r

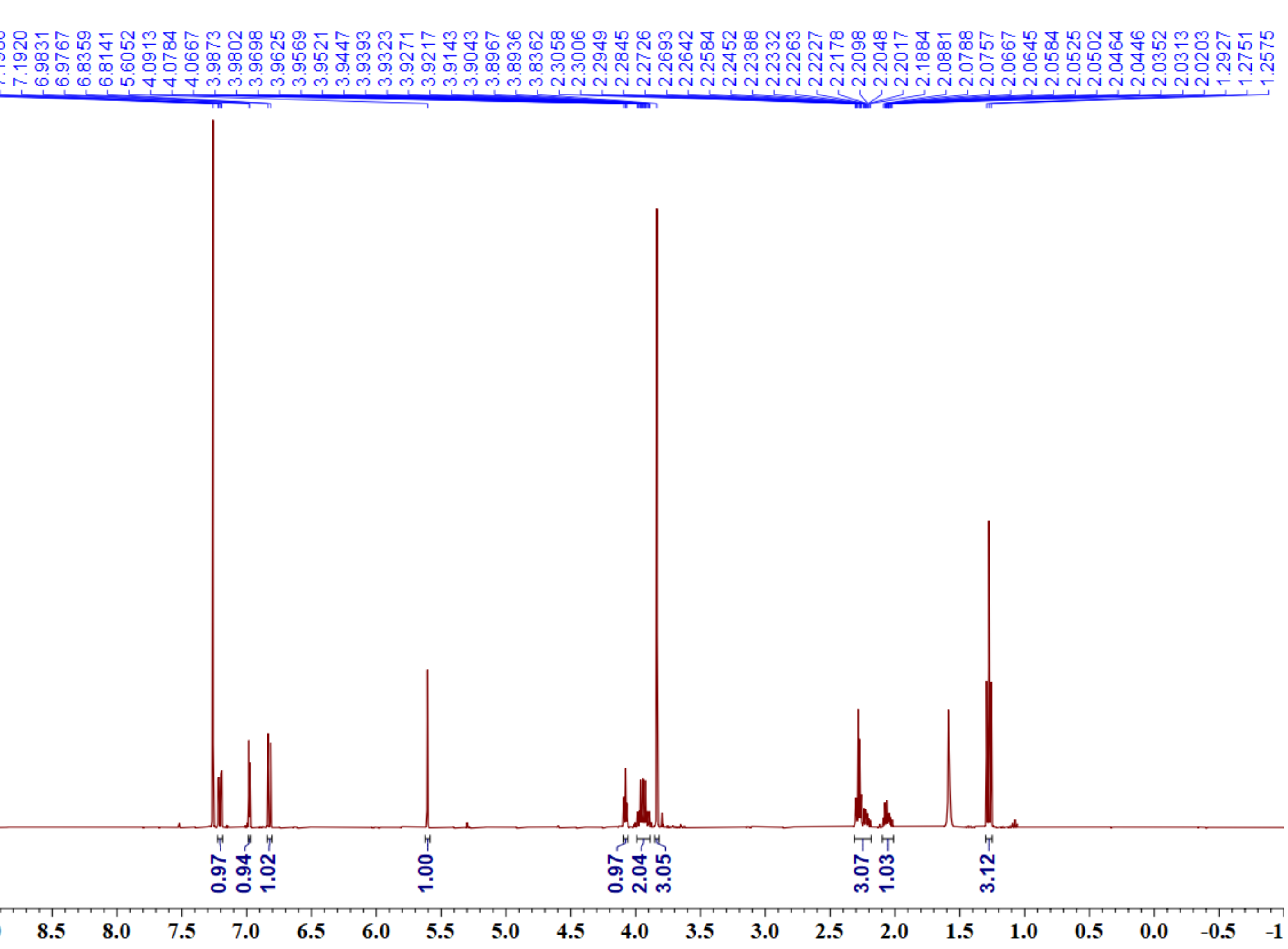

^{1}H NMR spectrum of compound 2r

YX-207-4-1data 1 —

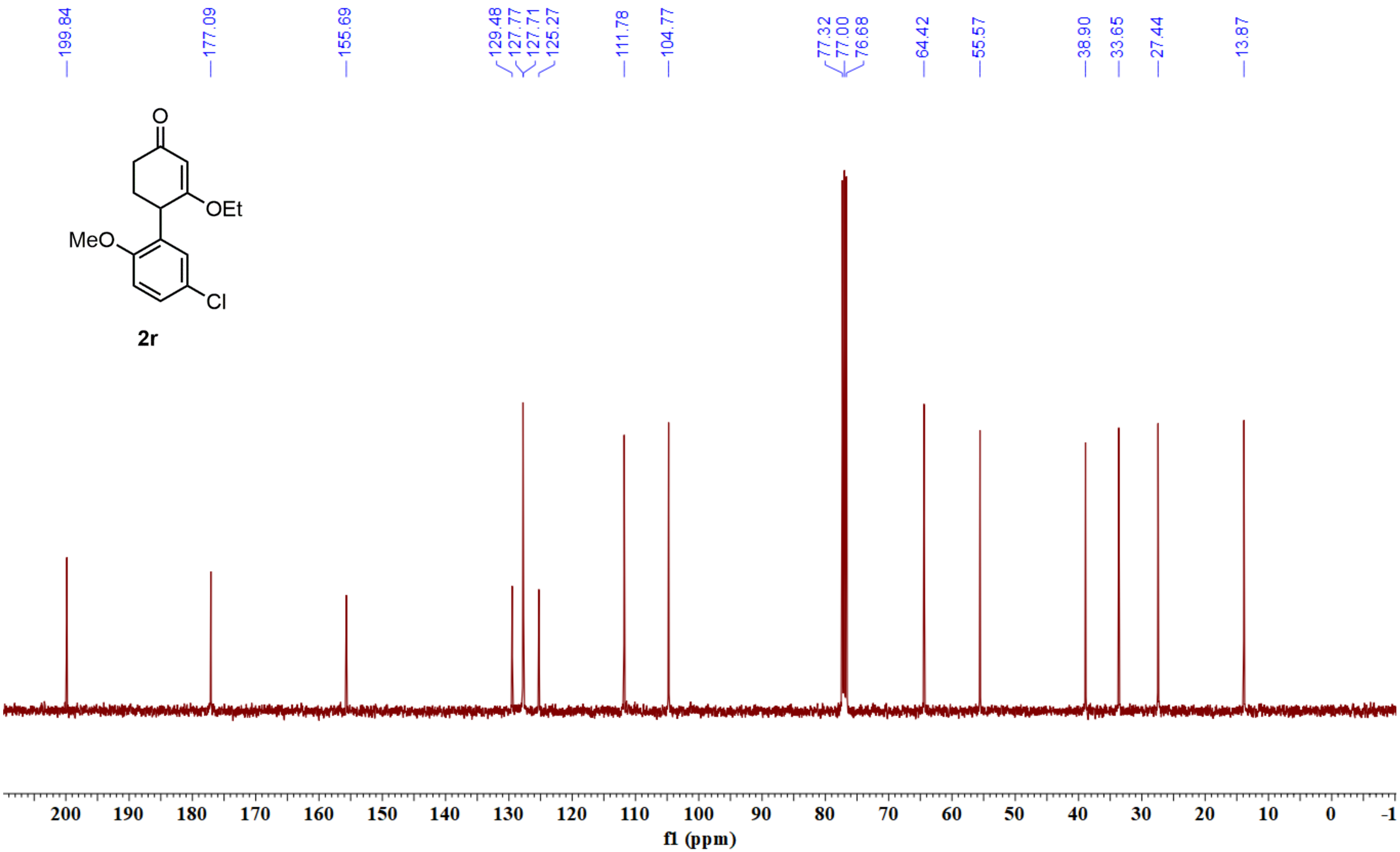

^{13}C NMR spectrum of compound 2r

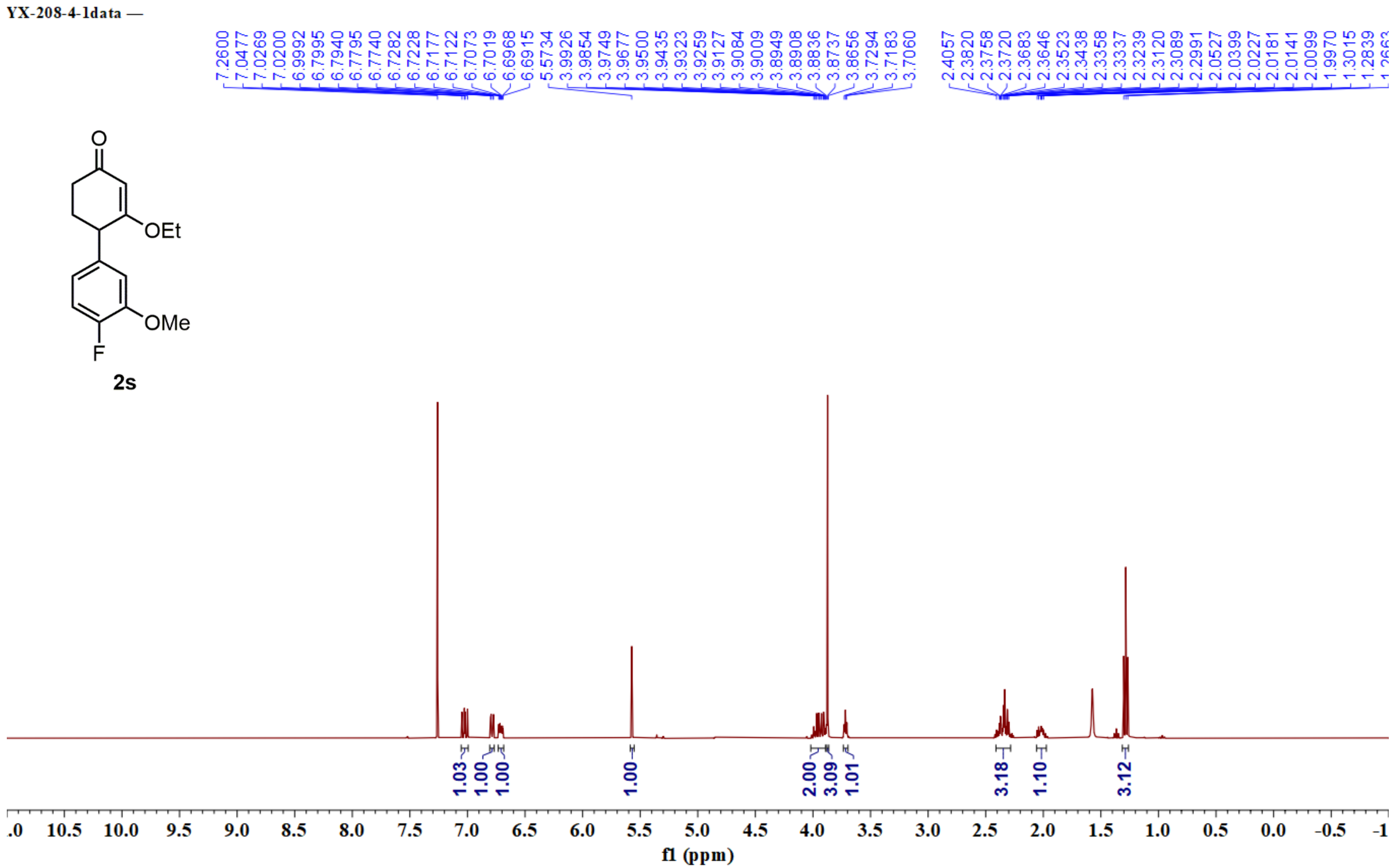

^{1}H NMR spectrum of compound 2s

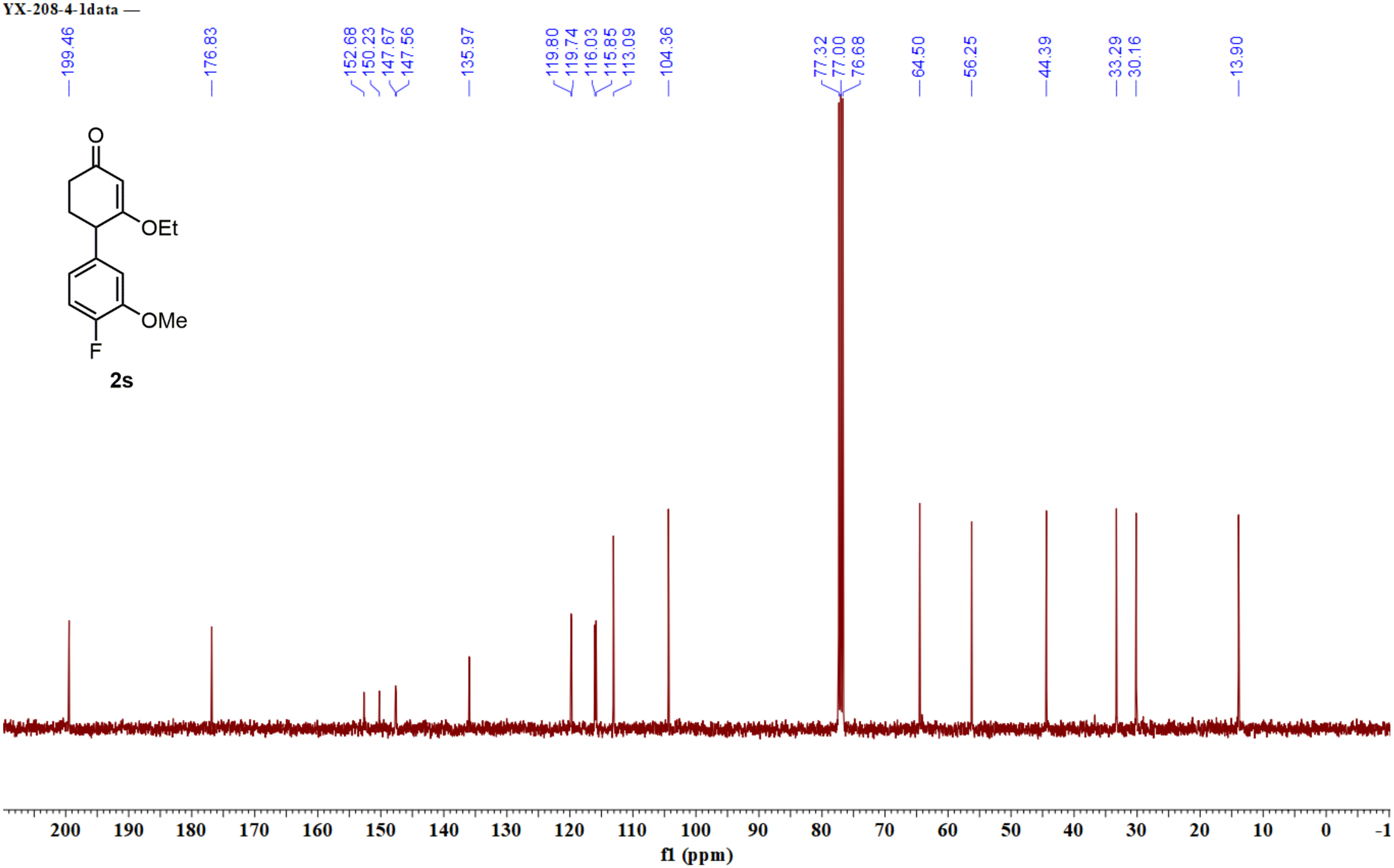

^{13}C NMR spectrum of compound 2s

YX-208-4-1data —

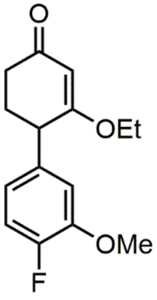

2s

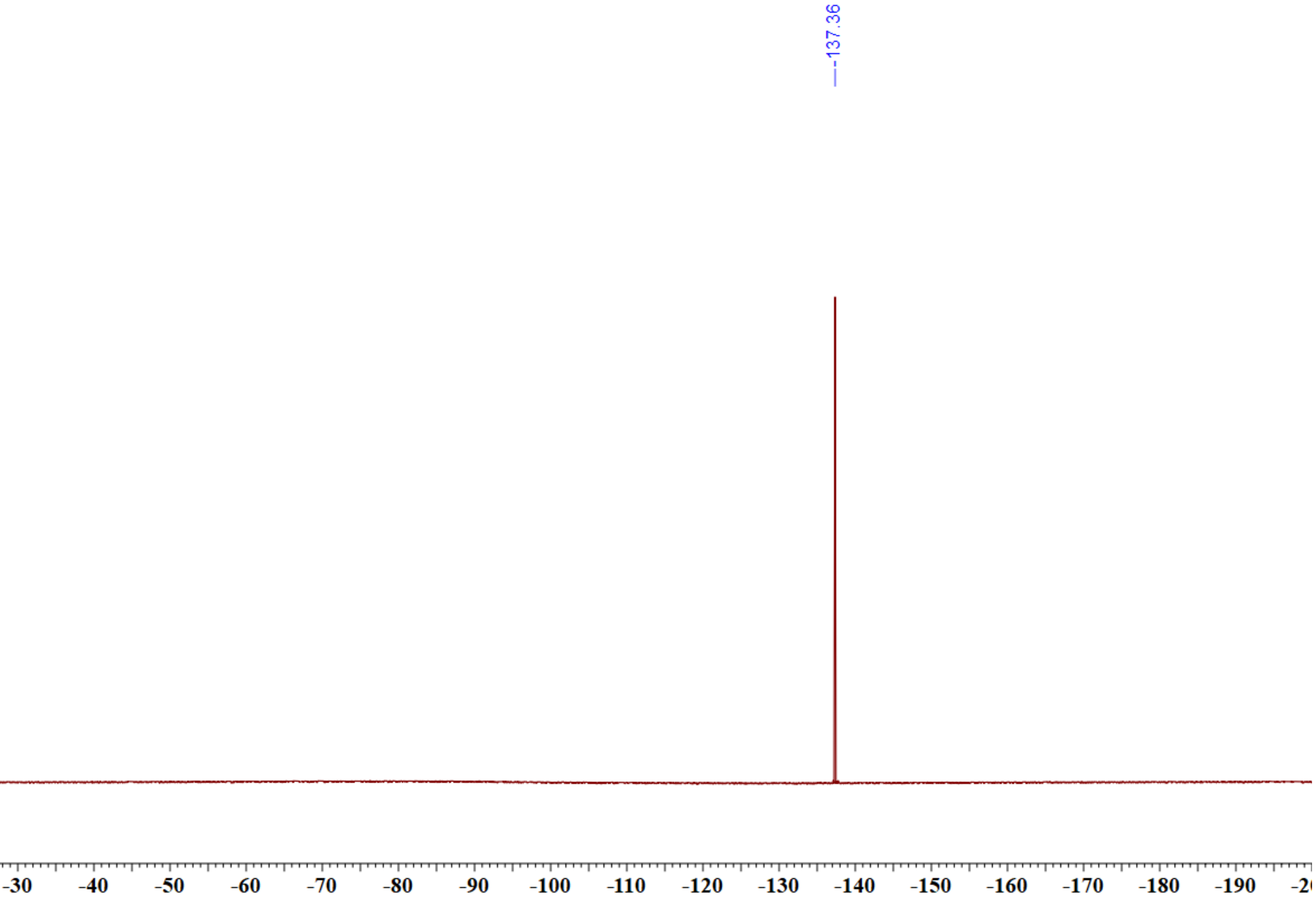

^{19}F NMR spectrum of compound 2s

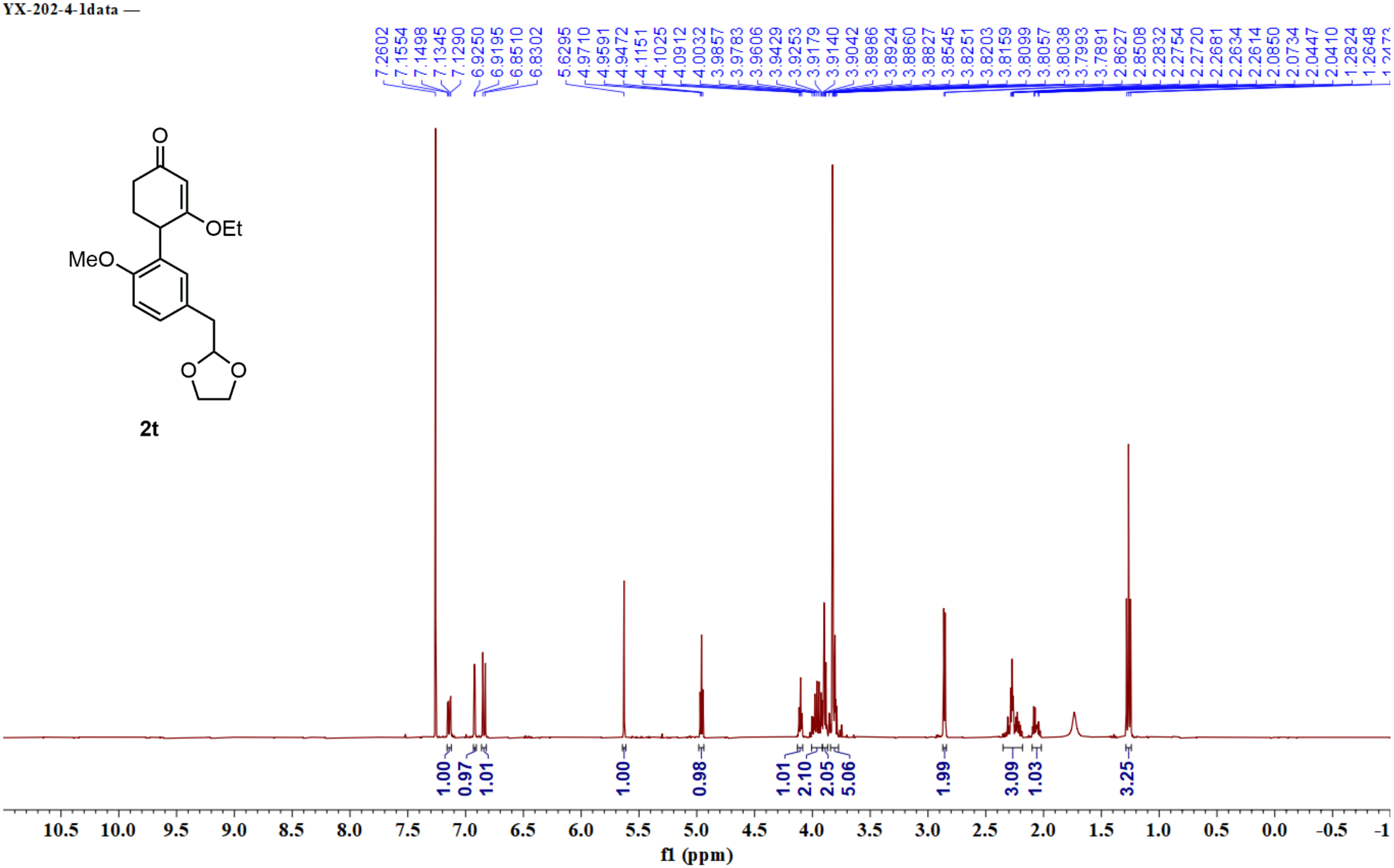

^{1}H NMR spectrum of compound 2t

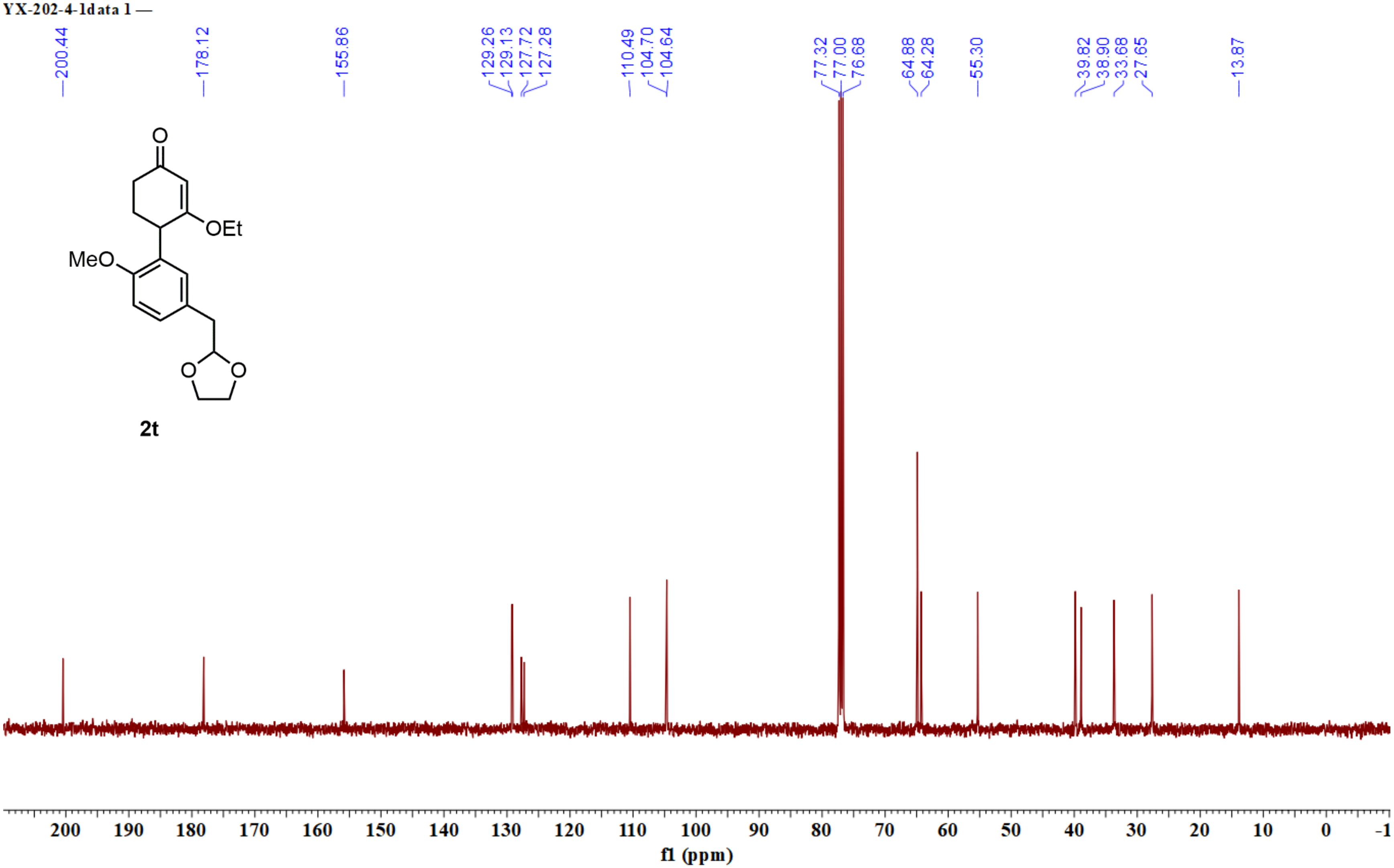

^{13}C NMR spectrum of compound 2t

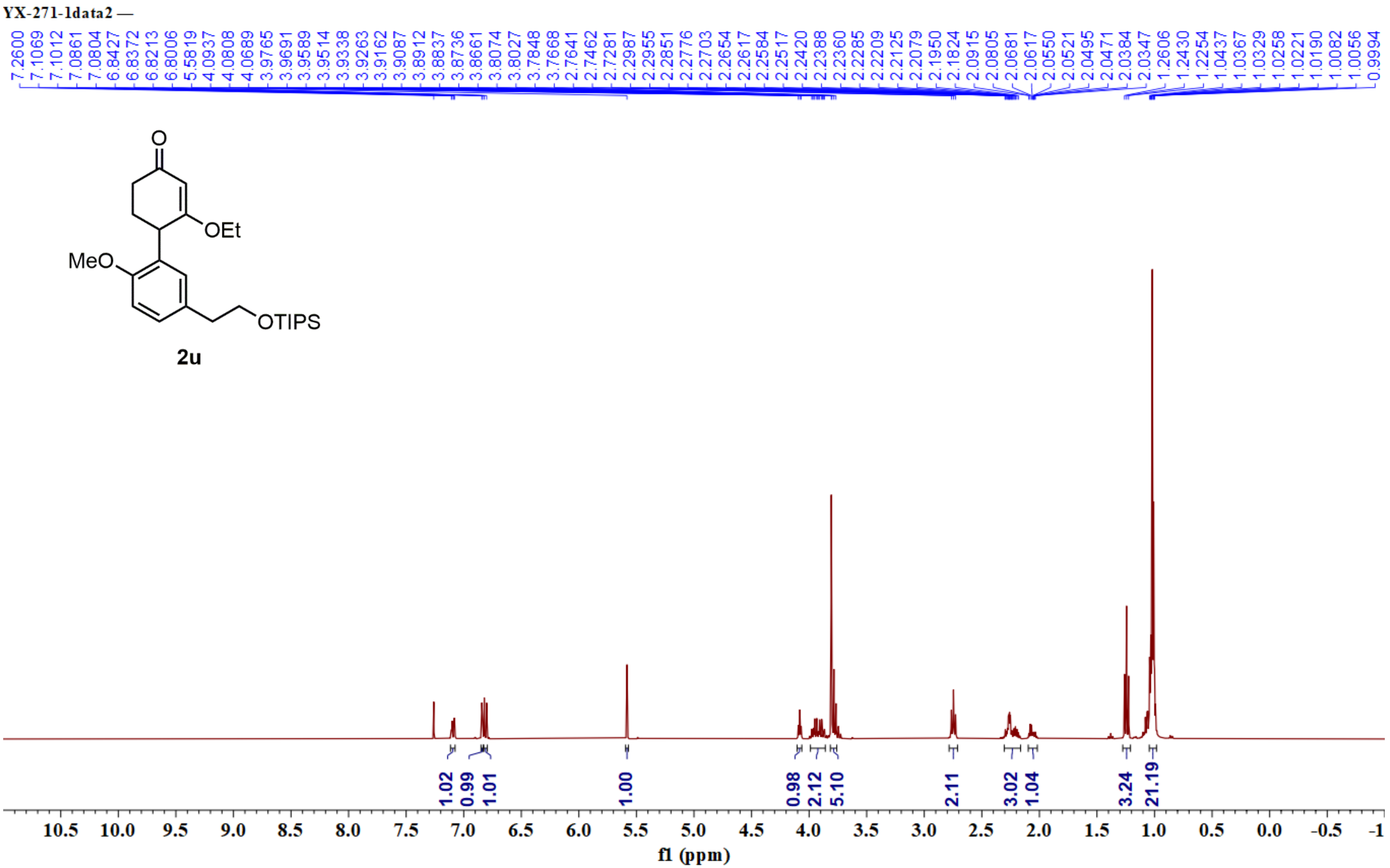

^{1}H NMR spectrum of compound 2u

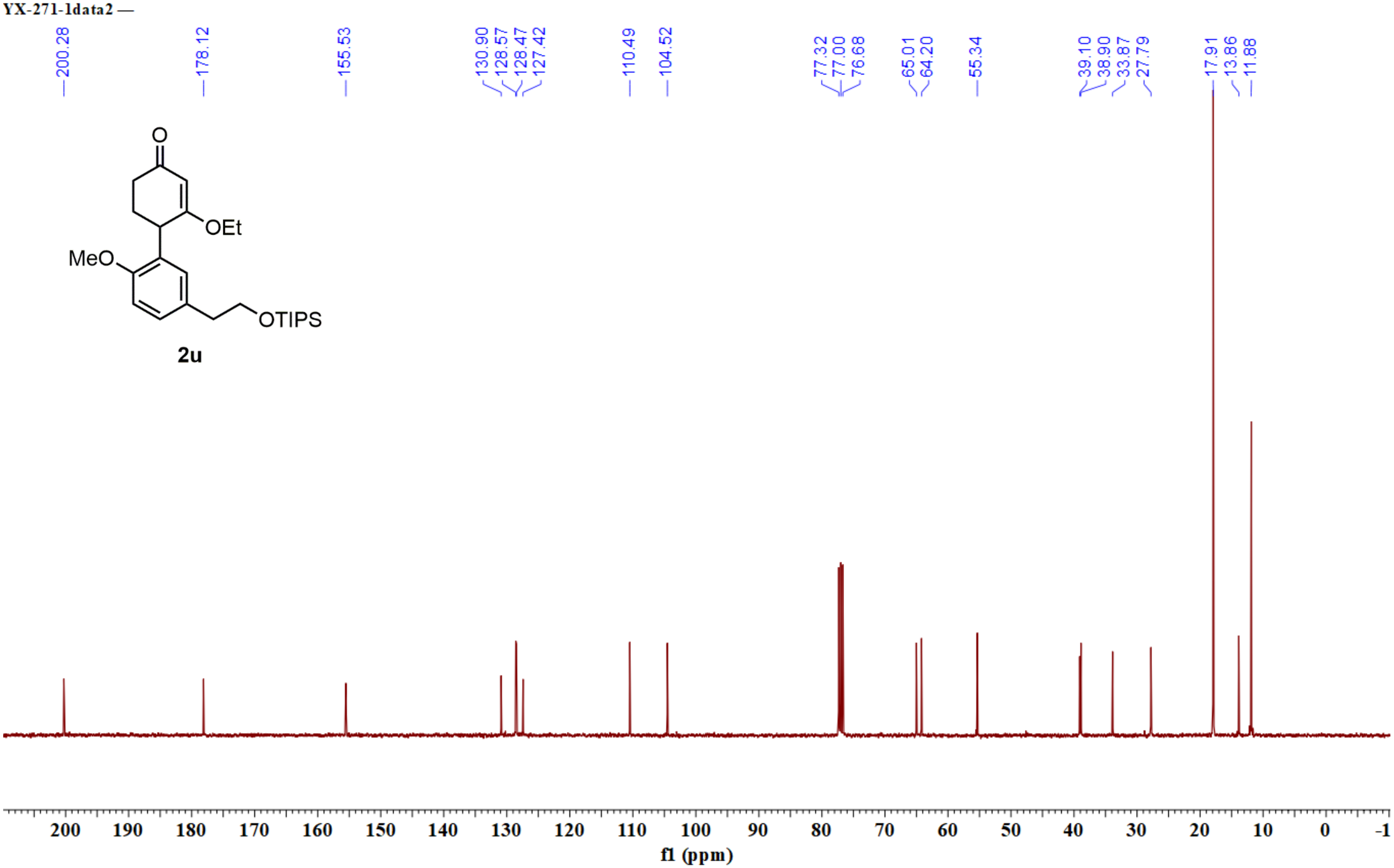

^{13}C NMR spectrum of compound 2u

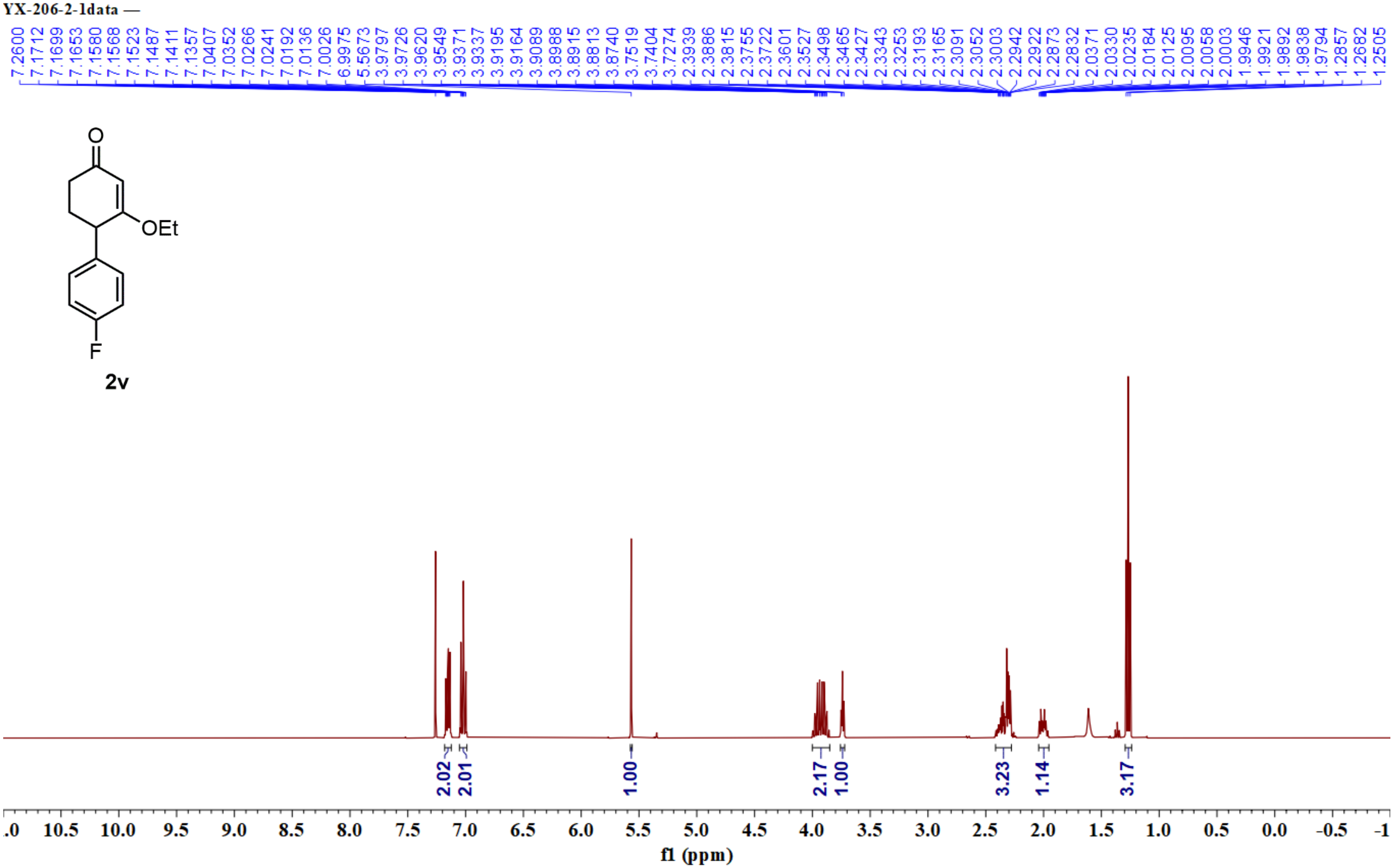

^{1}H NMR spectrum of compound 2v

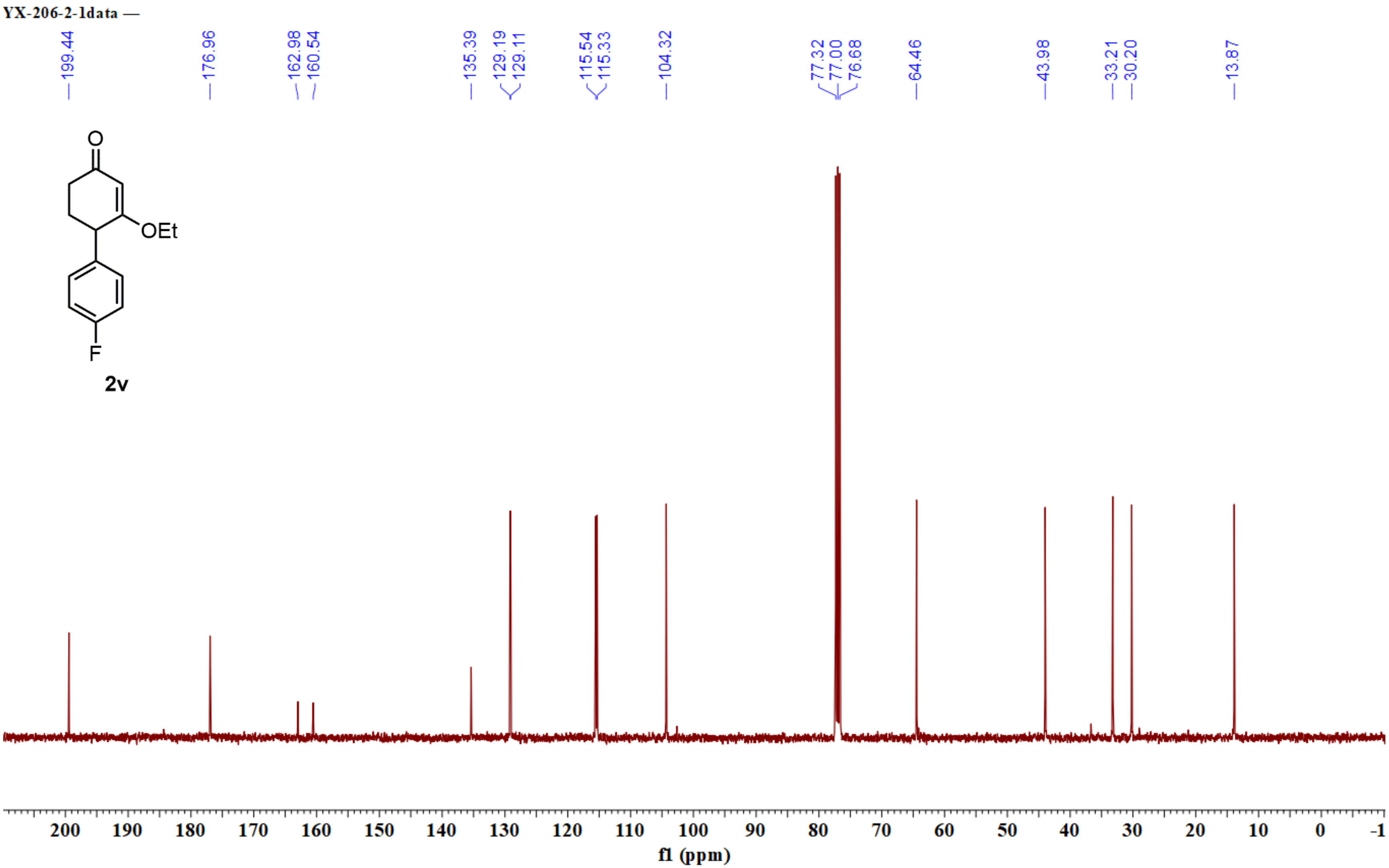

^{13}C NMR spectrum of compound 2v

YX-206-2-1data —

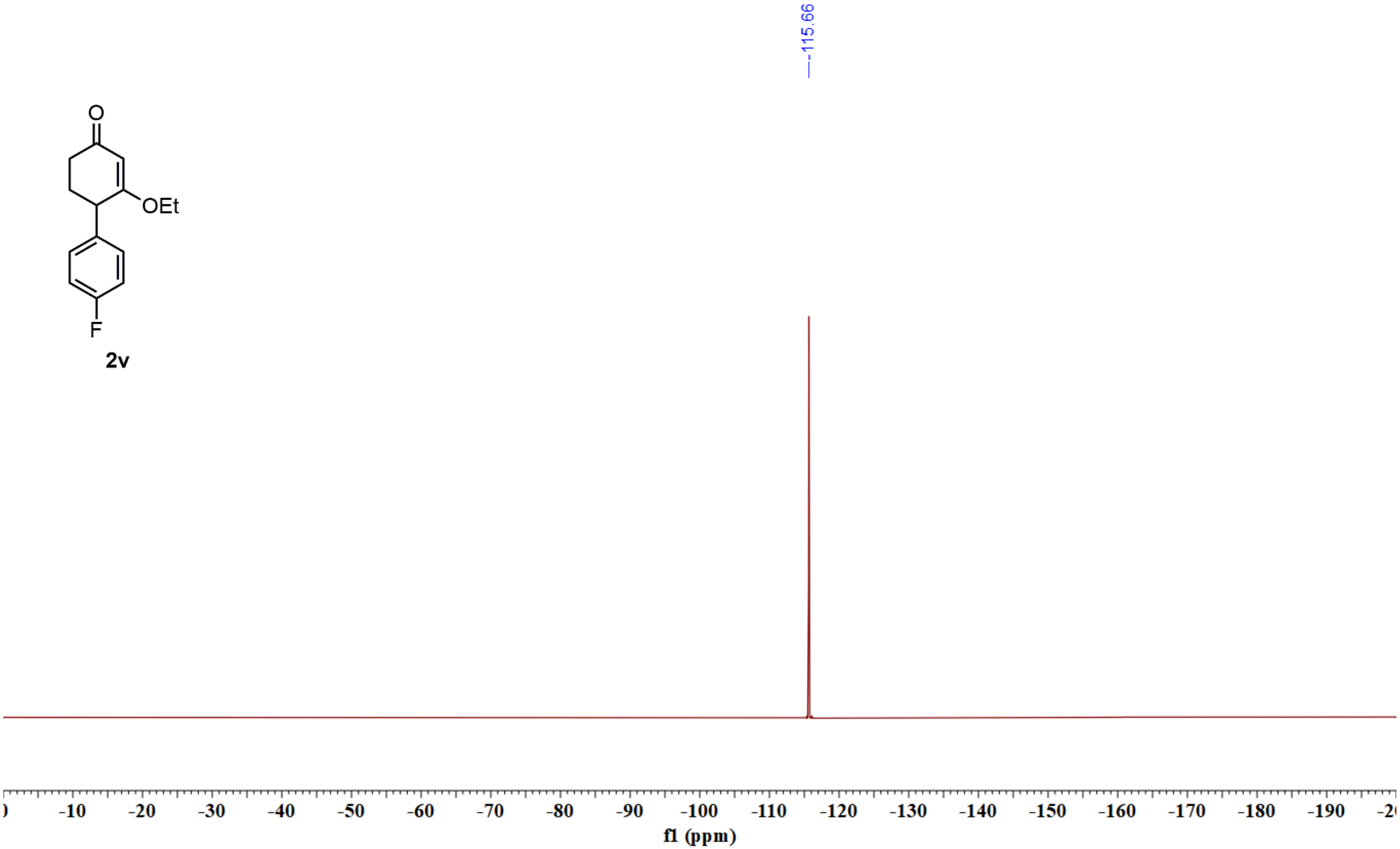

^{19}F NMR spectrum of compound 2v

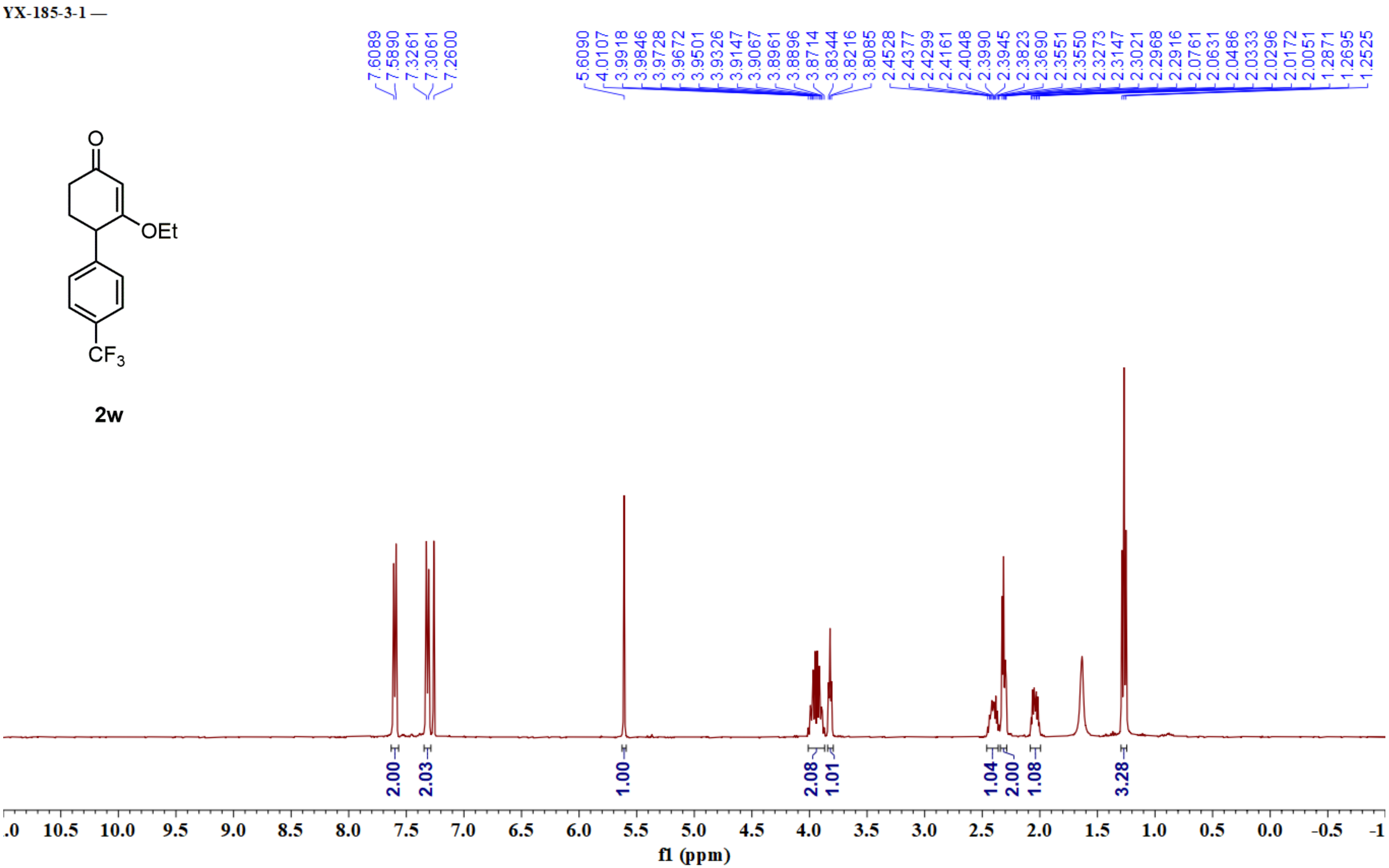

^{1}H NMR spectrum of compound 2w

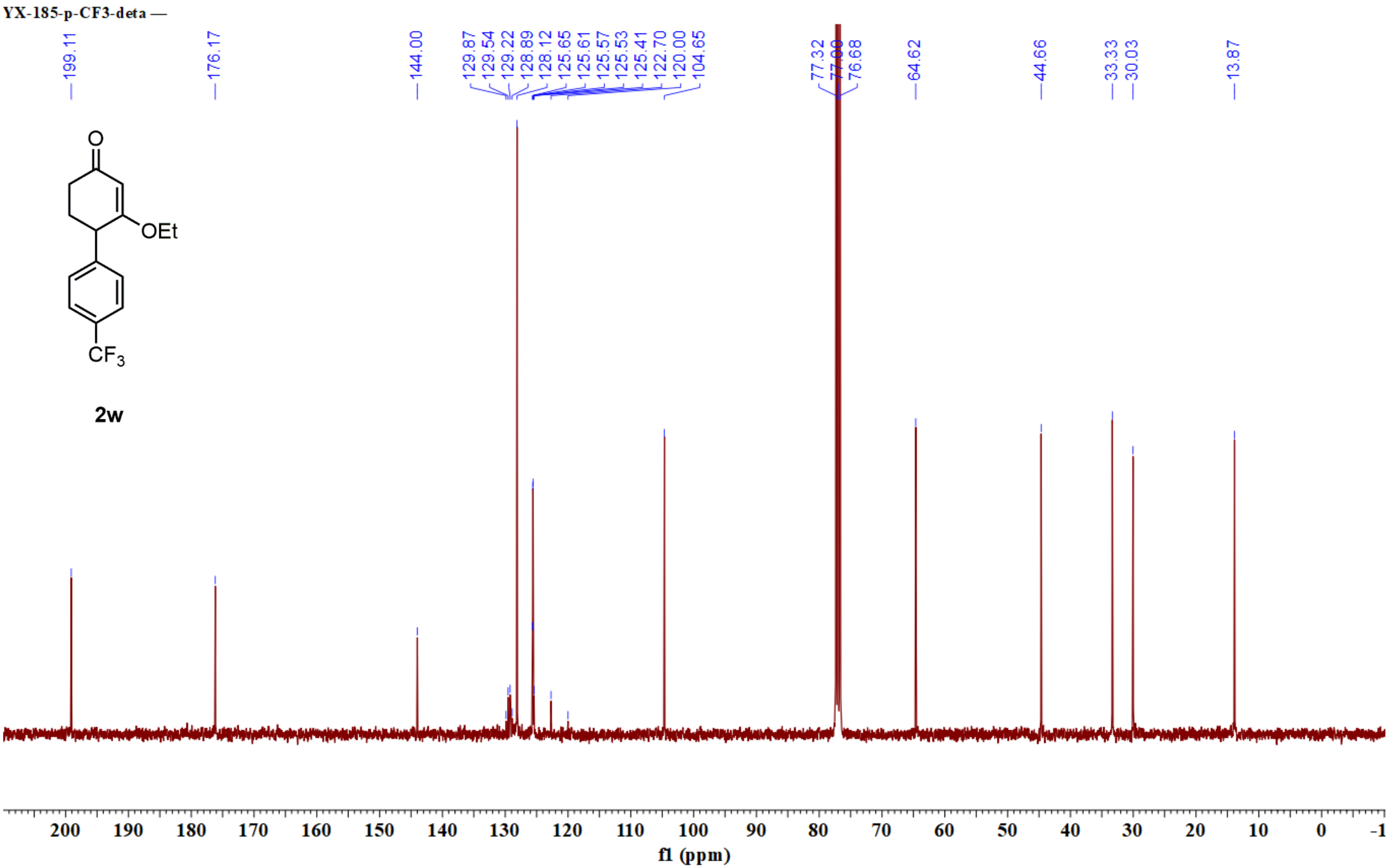

^{13}C NMR spectrum of compound 2w

YX-185-p-CF3-deta

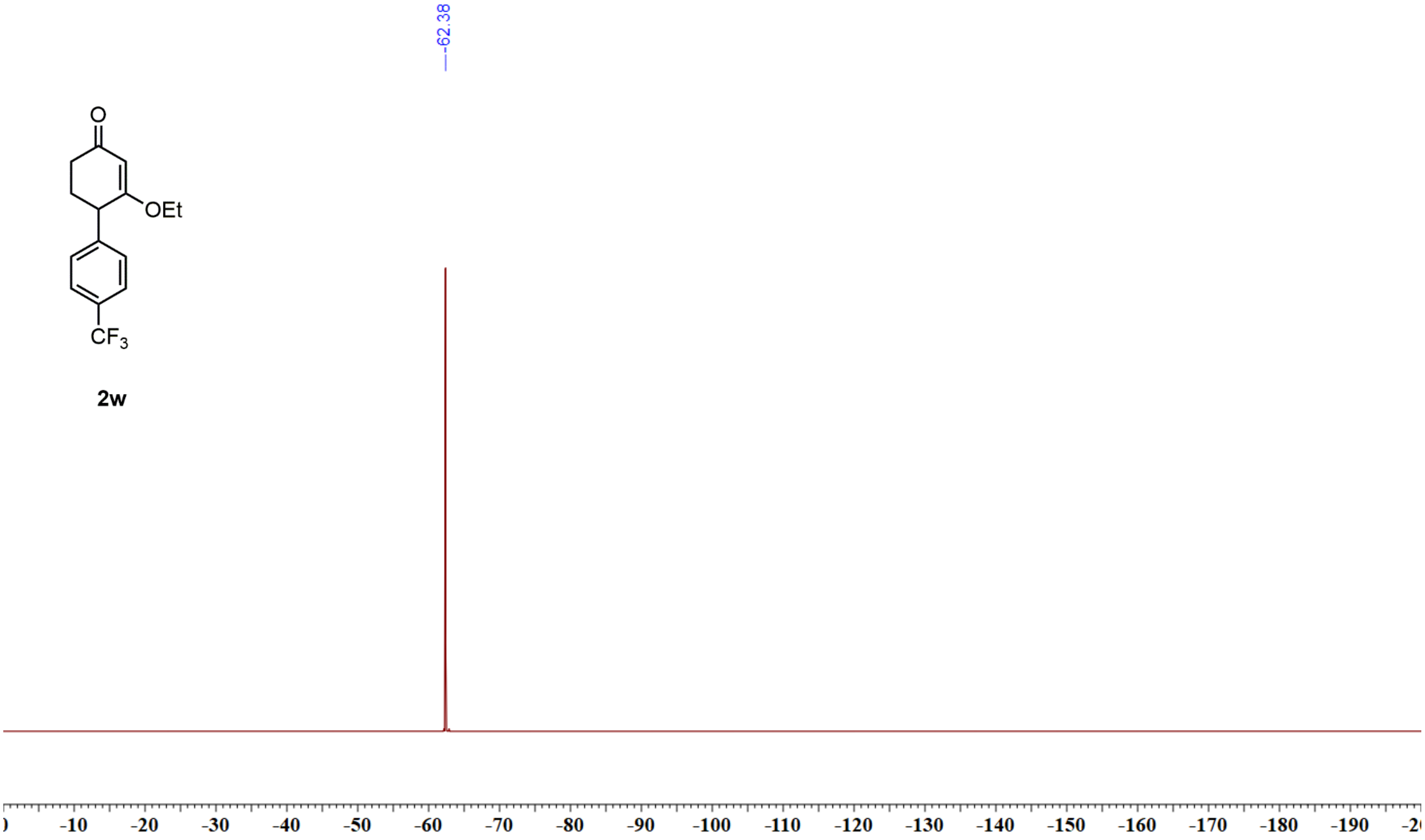

^{19}F NMR spectrum of compound 2w

PROTON_01 — YX-295-2data —

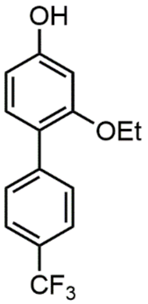

S1

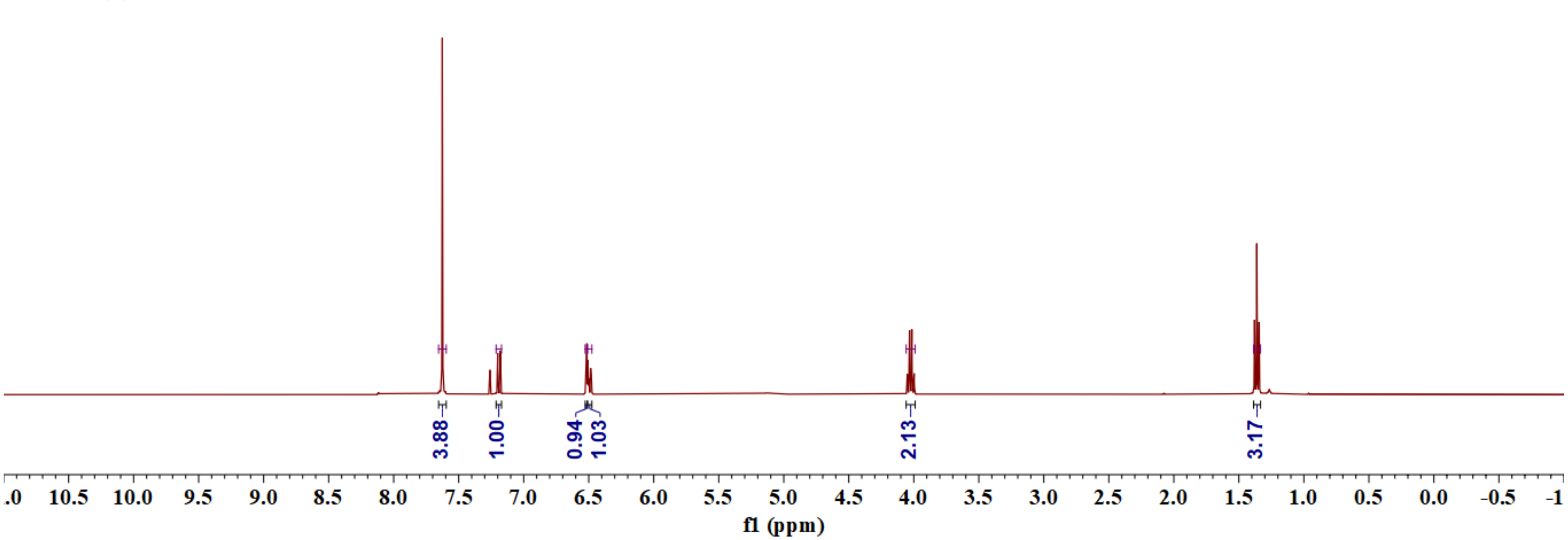

^{1}H NMR spectrum of compound S1

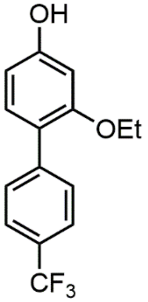

S1

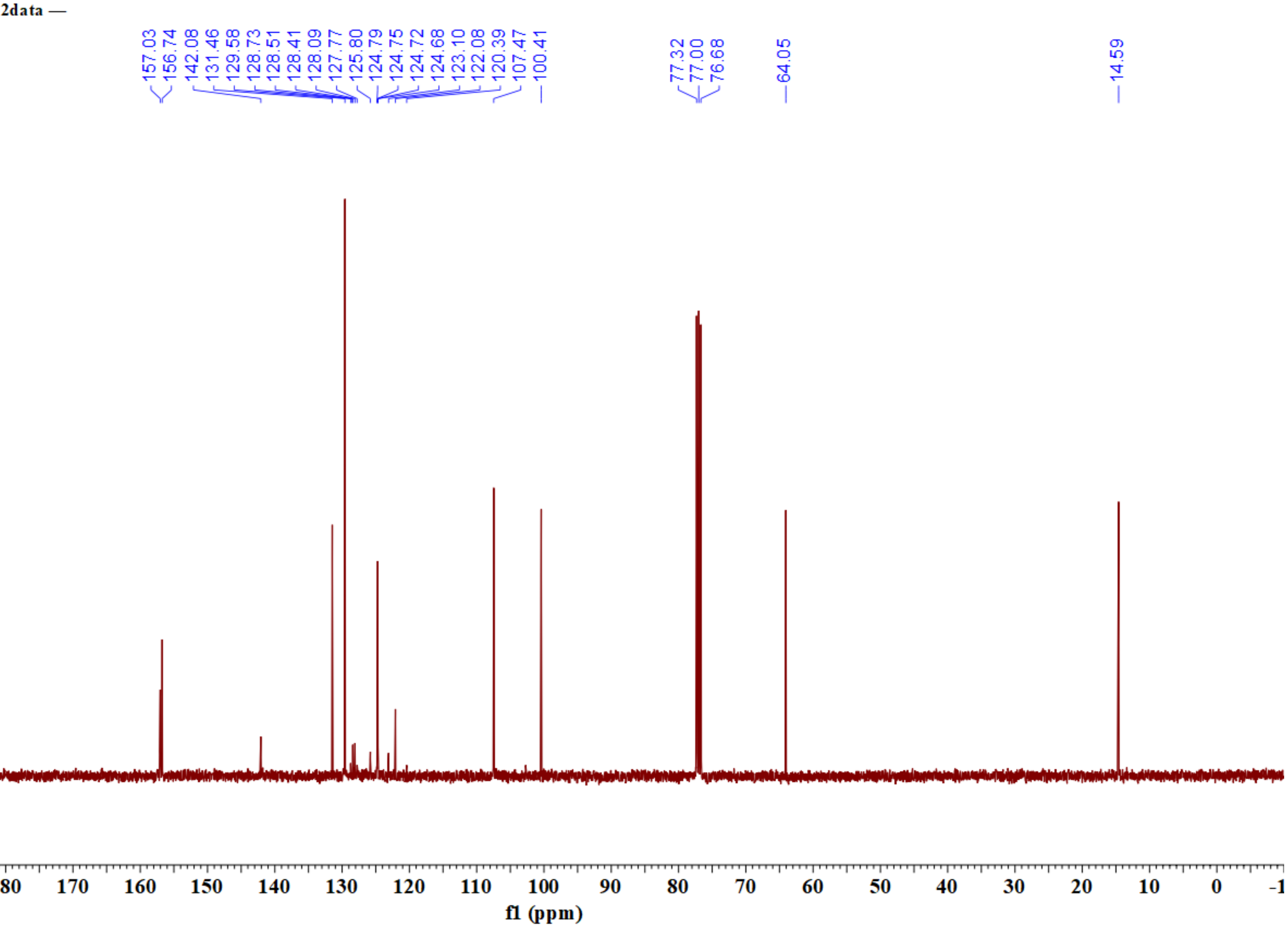

^{13}C NMR spectrum of compound S1

FLUORINE_01 — YX-295-2data —

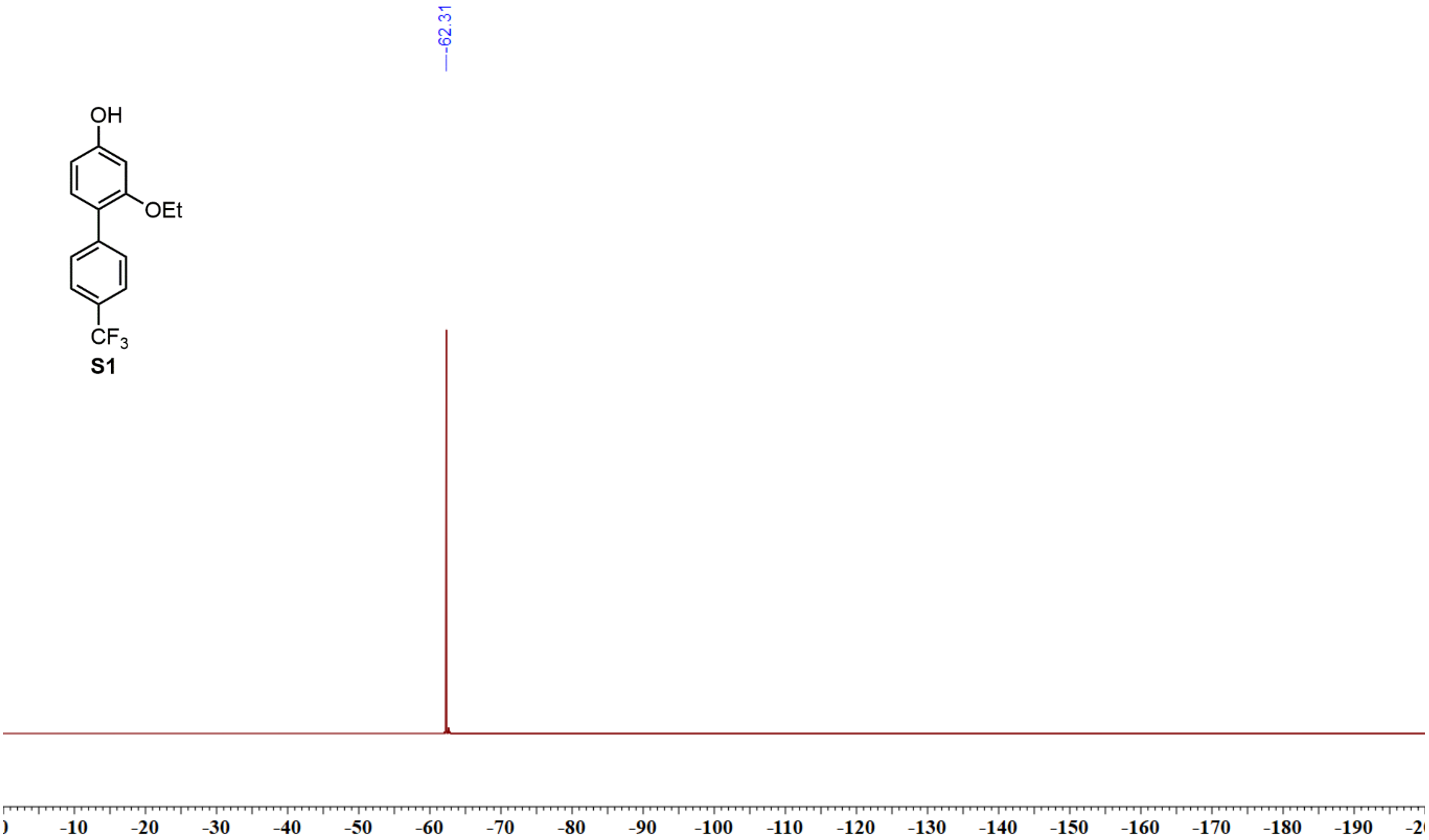

^{19}F NMR spectrum of compound S1

YX-SM-aromatization test —

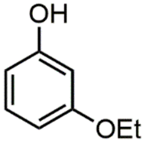

S2

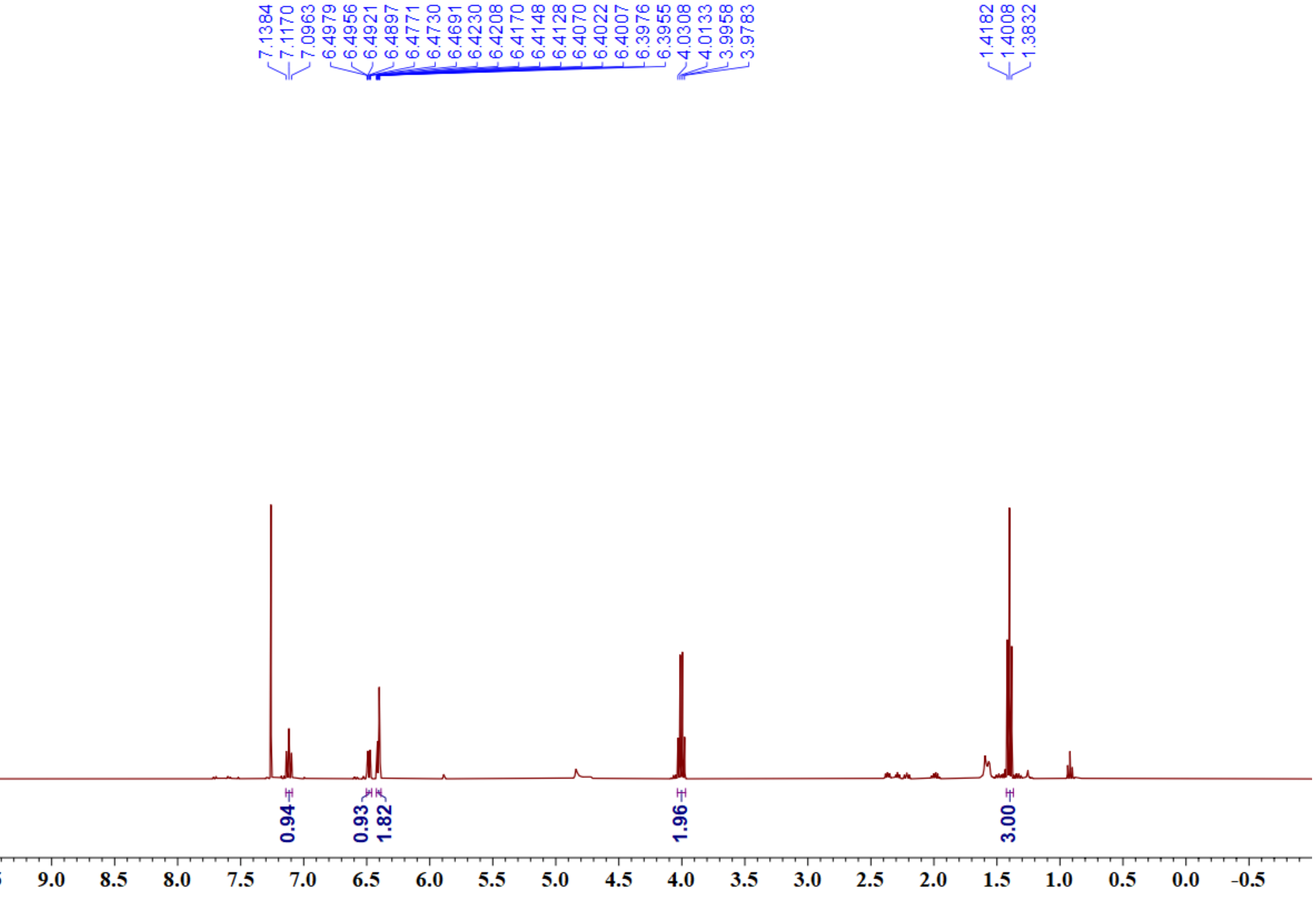

^{1}H NMR spectrum of compound S2

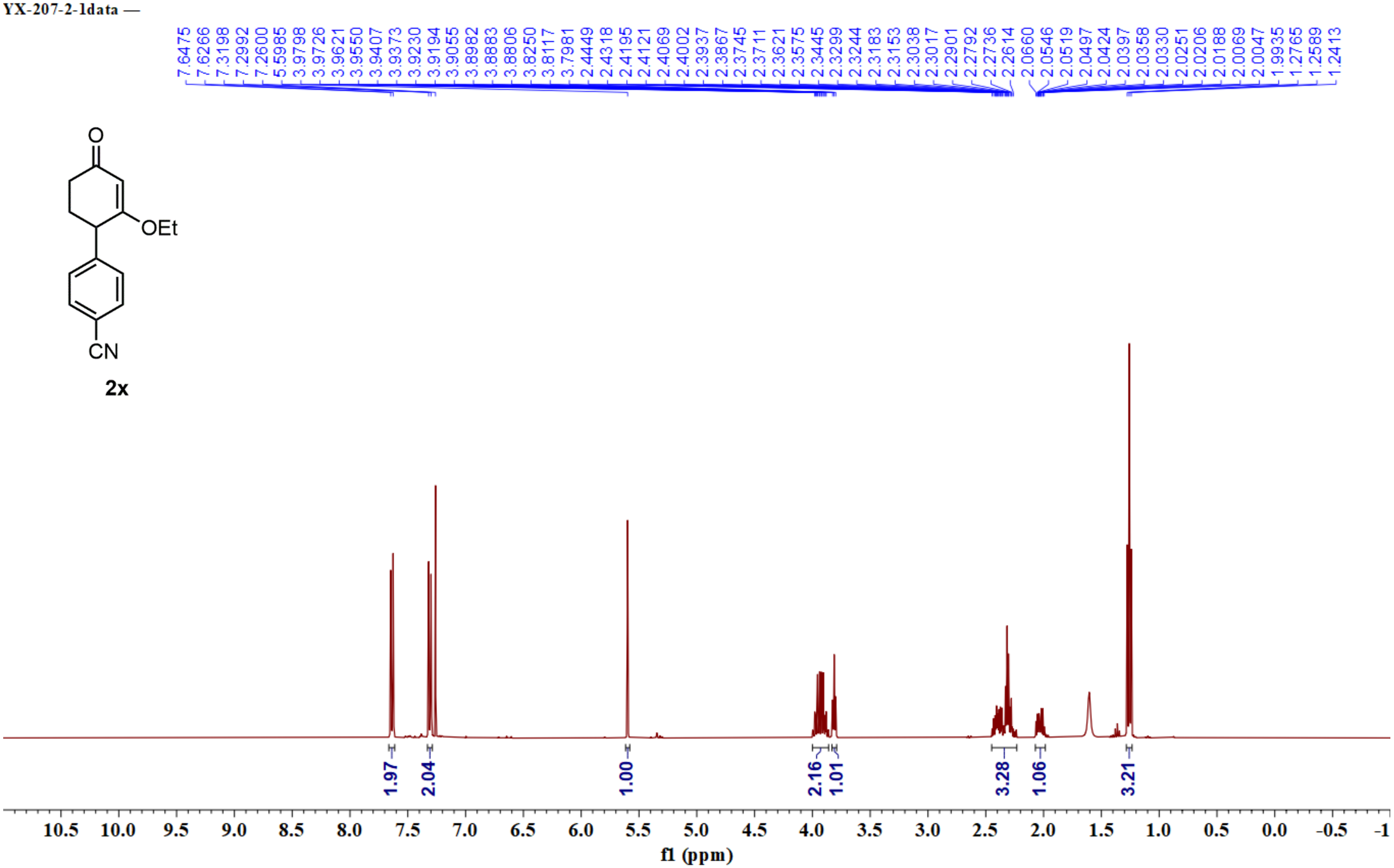

^{1}H NMR spectrum of compound 2x

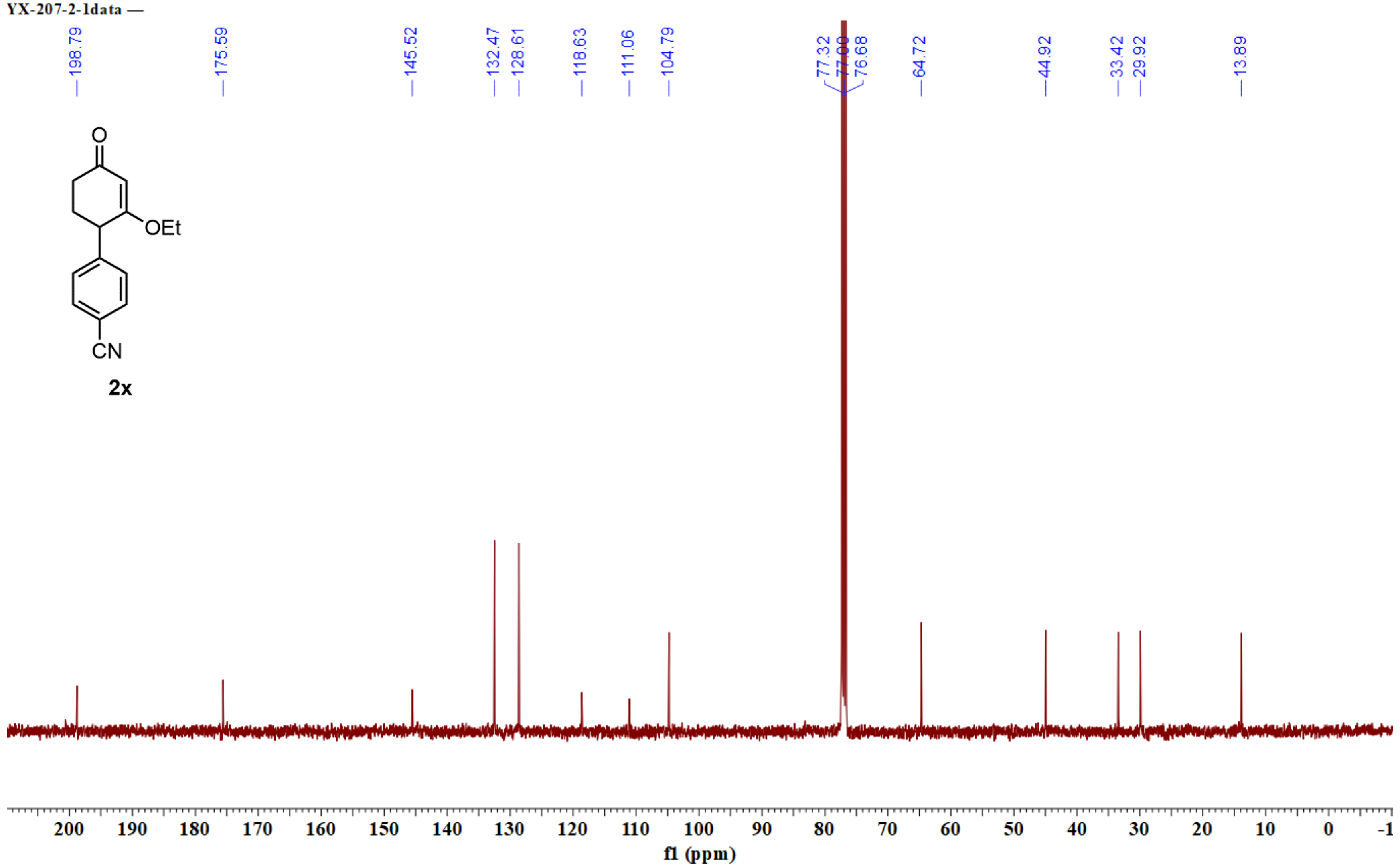

^{13}C NMR spectrum of compound 2x

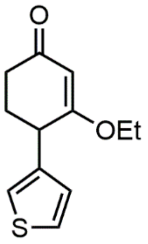

2y

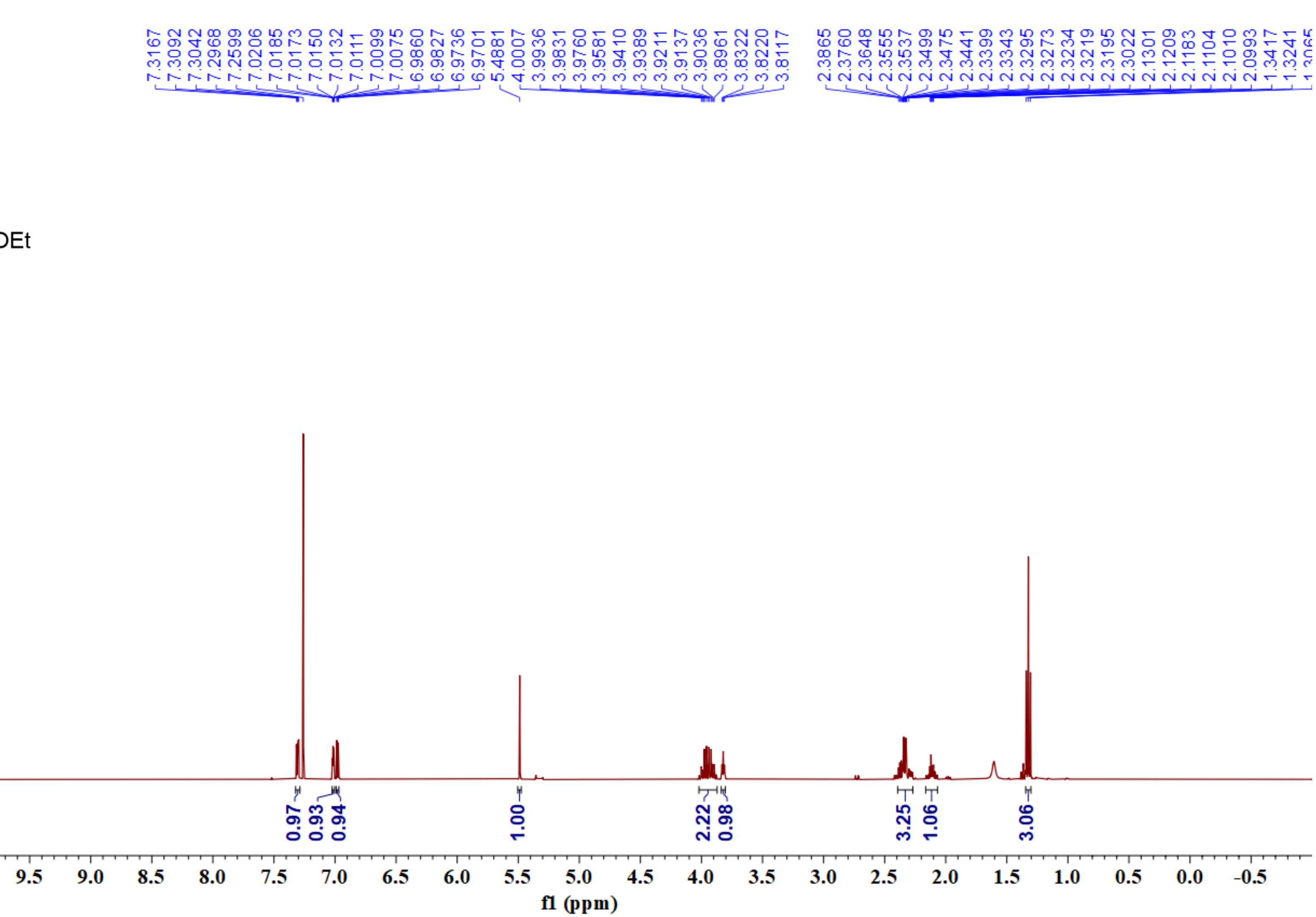

^{1}H NMR spectrum of compound 2y

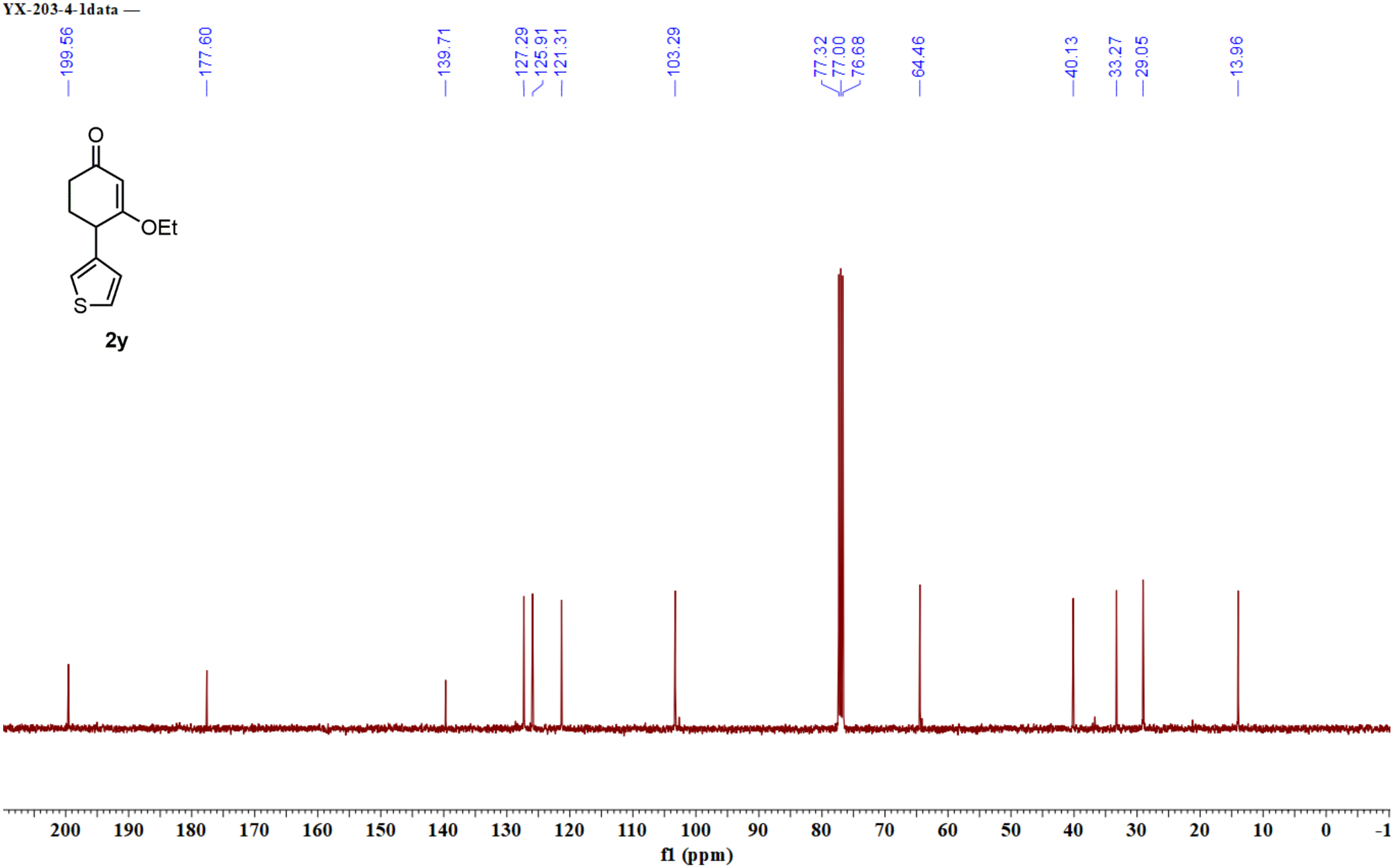

^{13}C NMR spectrum of compound 2y

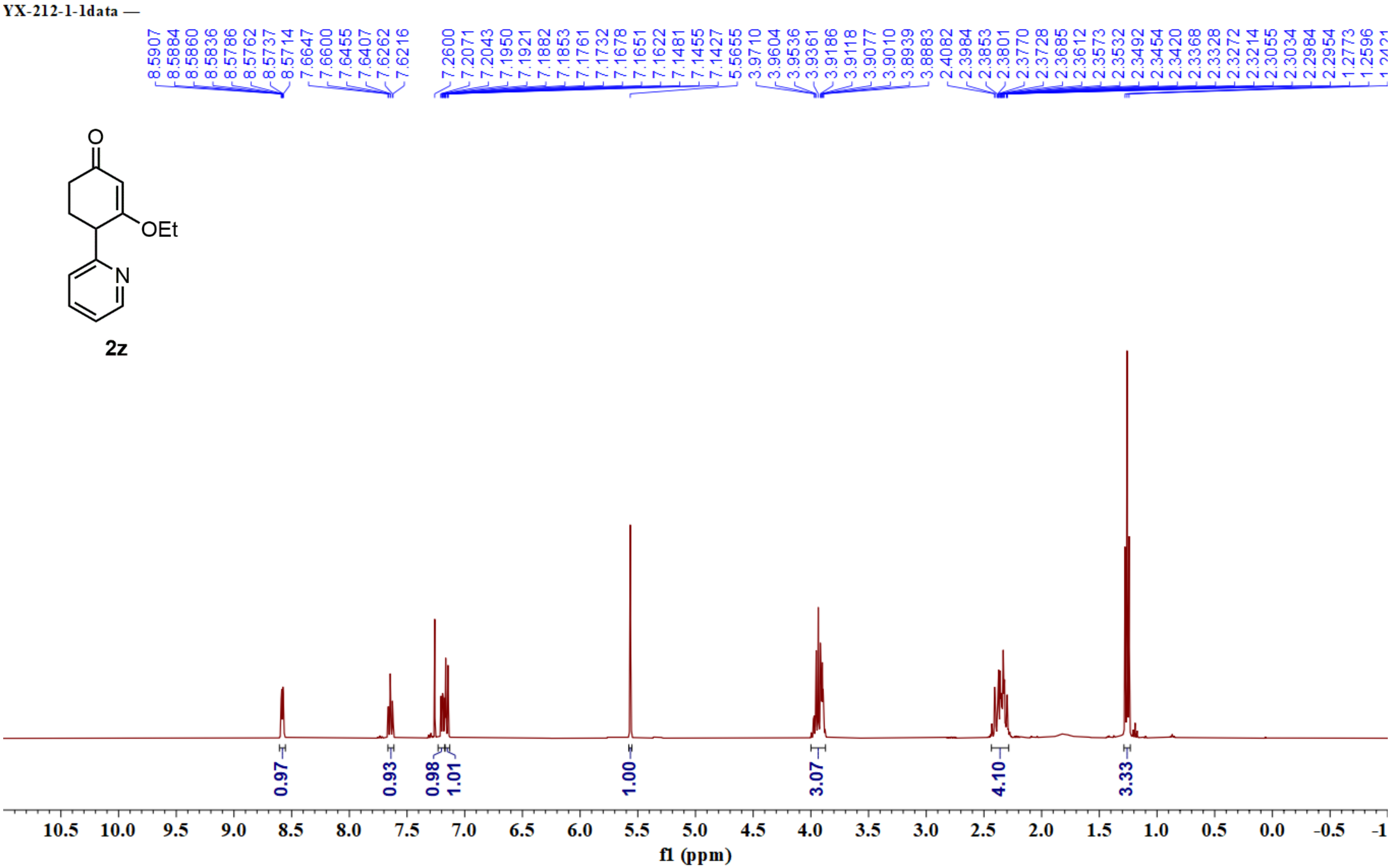

^{1}H NMR spectrum of compound 2z

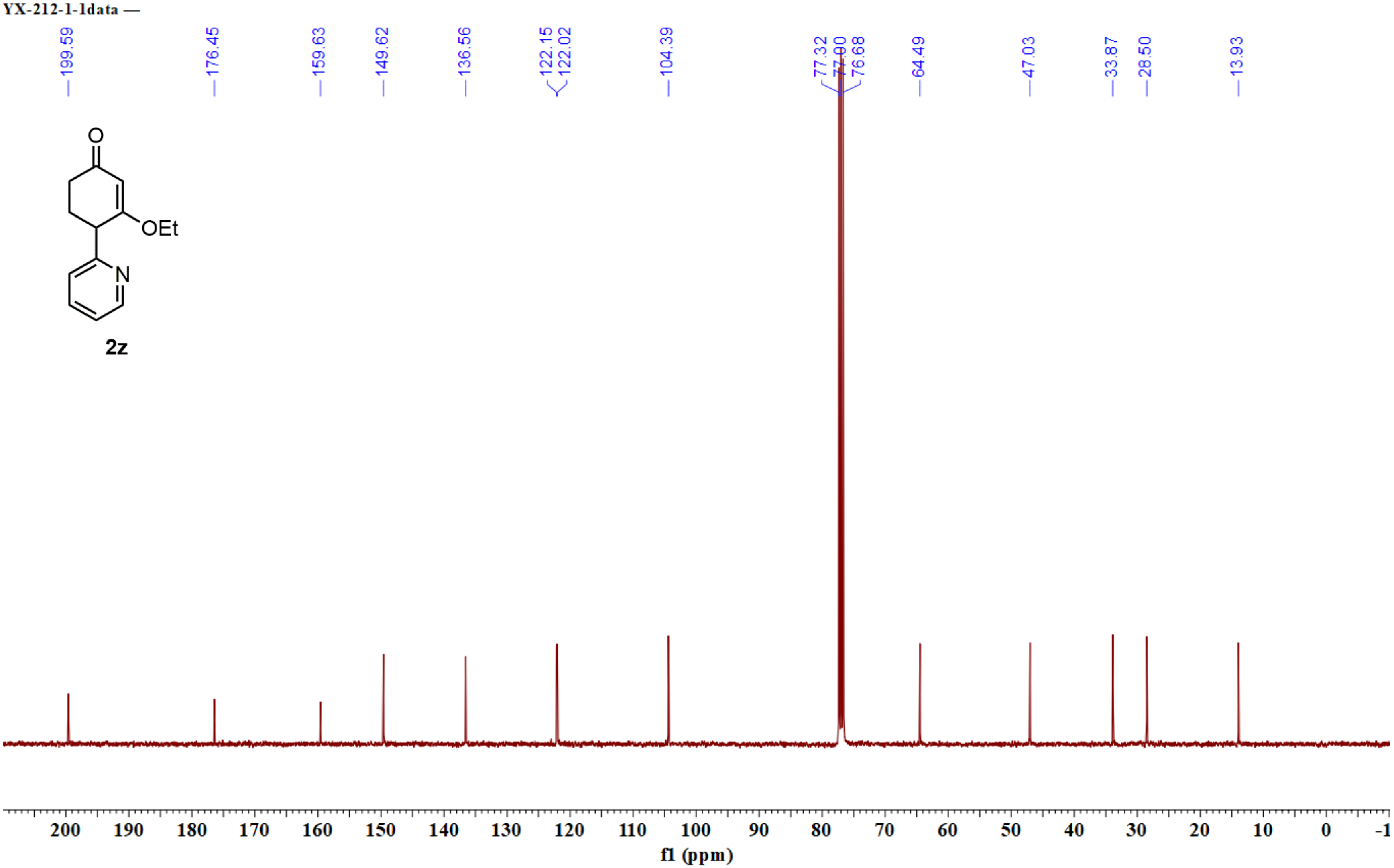

^{13}C NMR spectrum of compound 2z

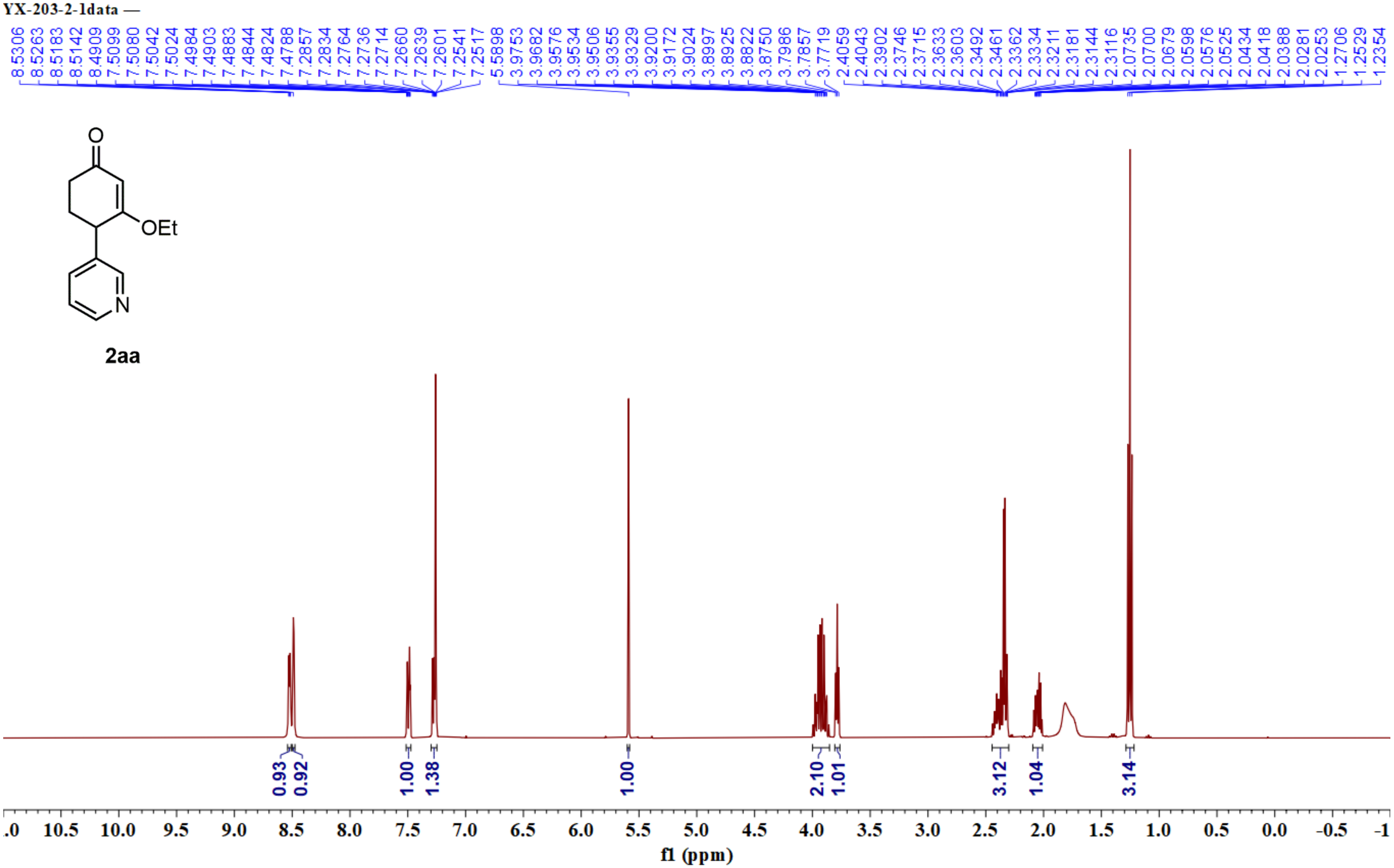

^{1}H NMR spectrum of compound 2aa

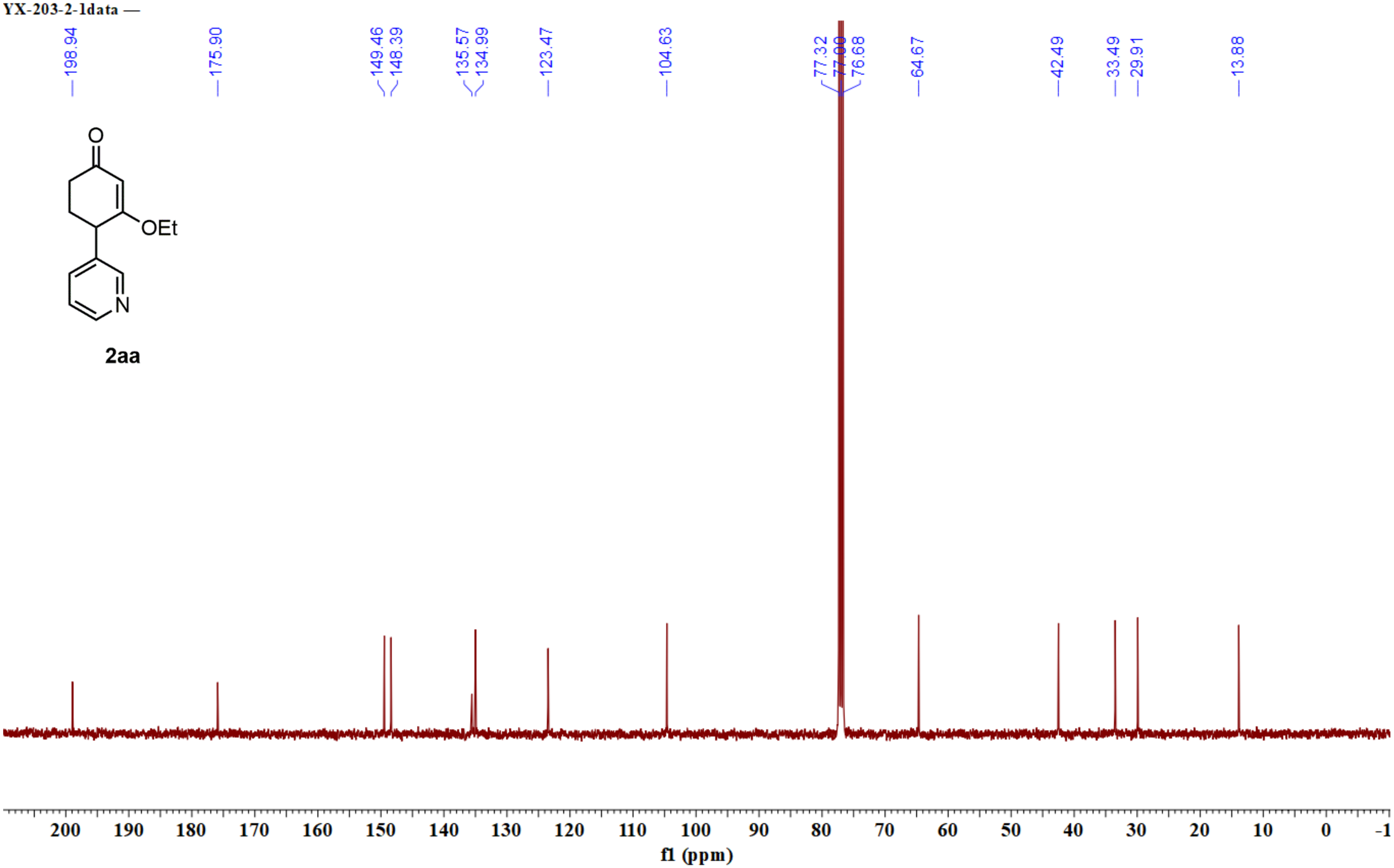

^{13}C NMR spectrum of compound 2aa

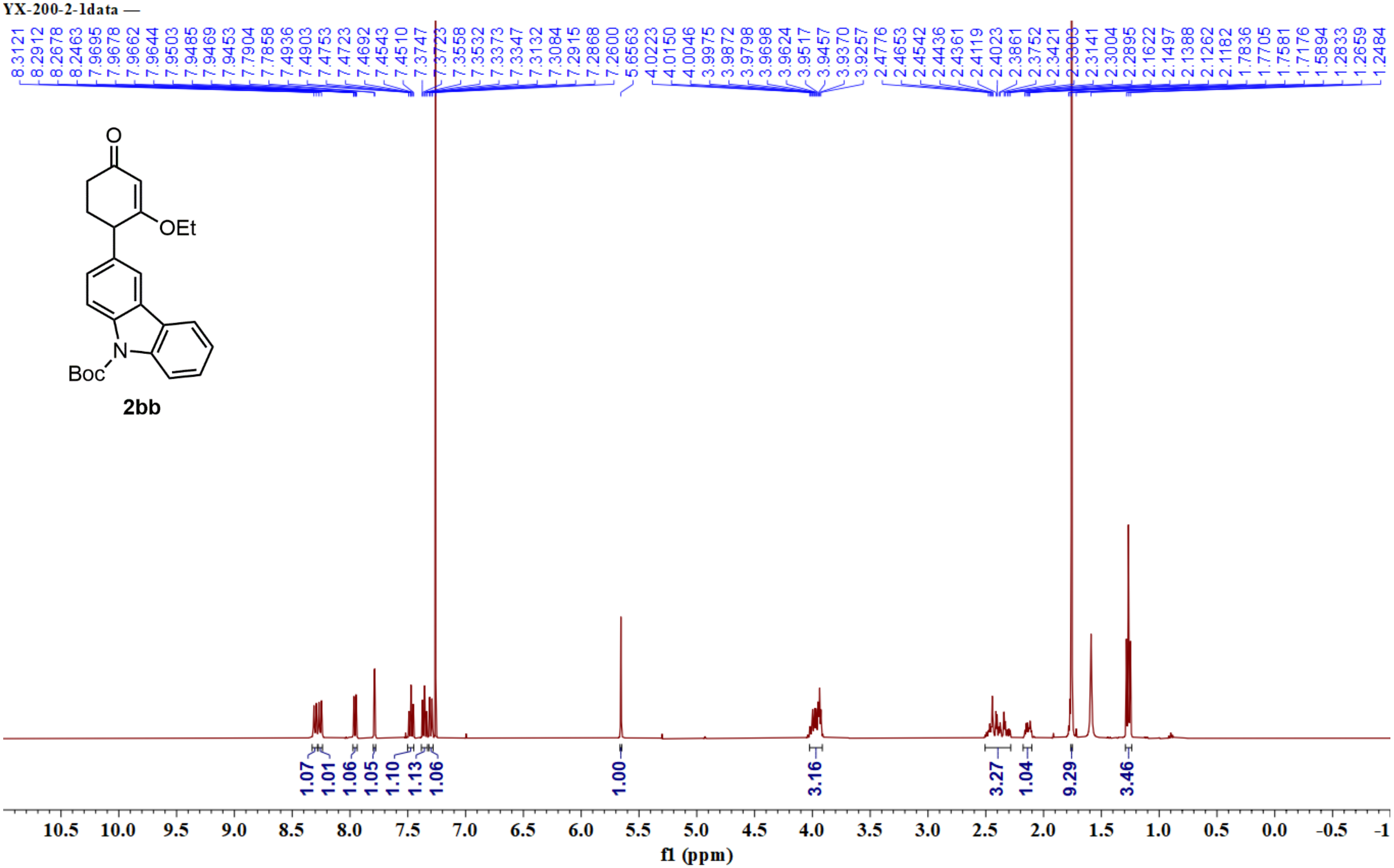

^{1}H NMR spectrum of compound 2bb

YX-200-2-1data 1 —

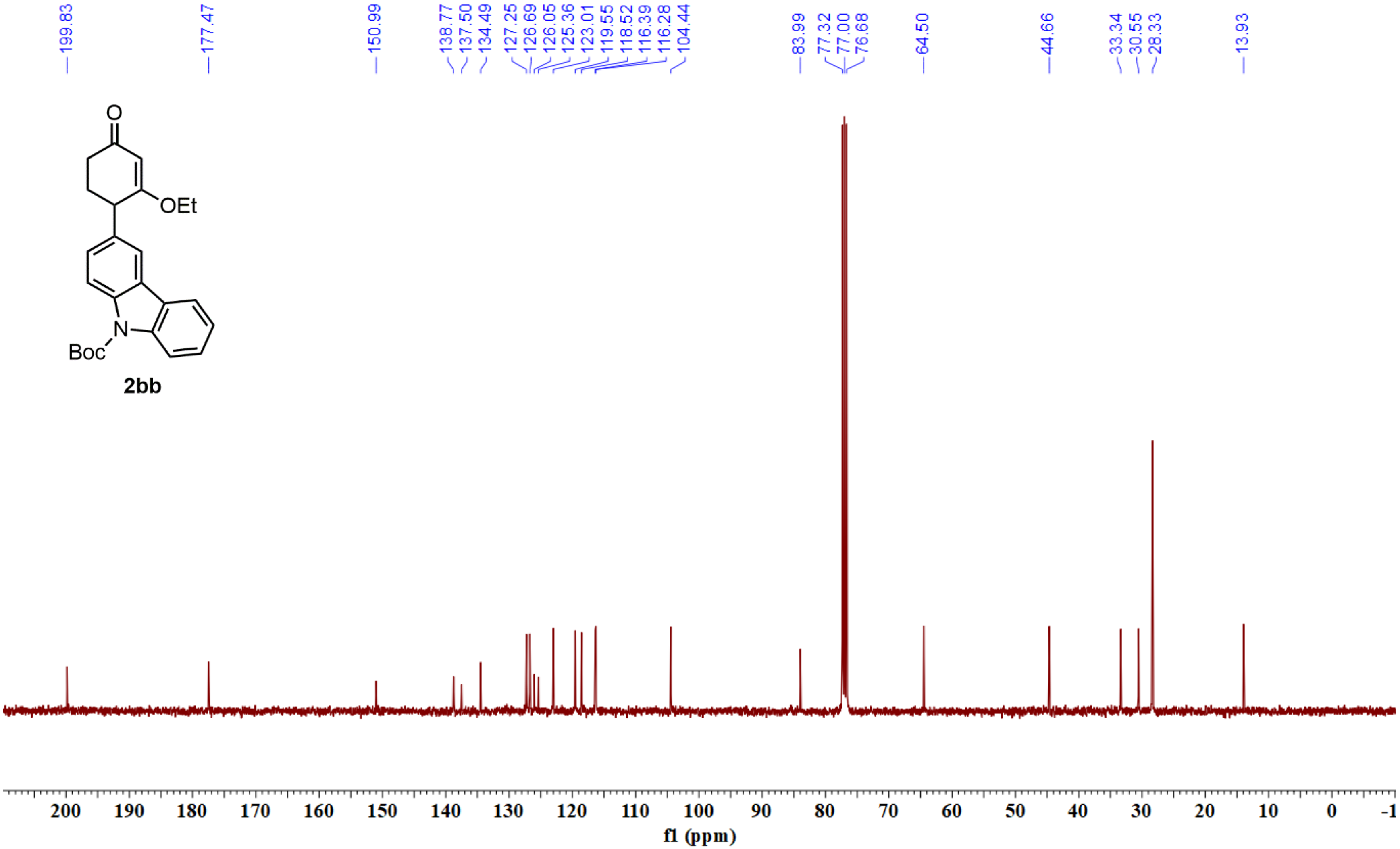

^{13}C NMR spectrum of compound 2bb

YX-233-1-1data —

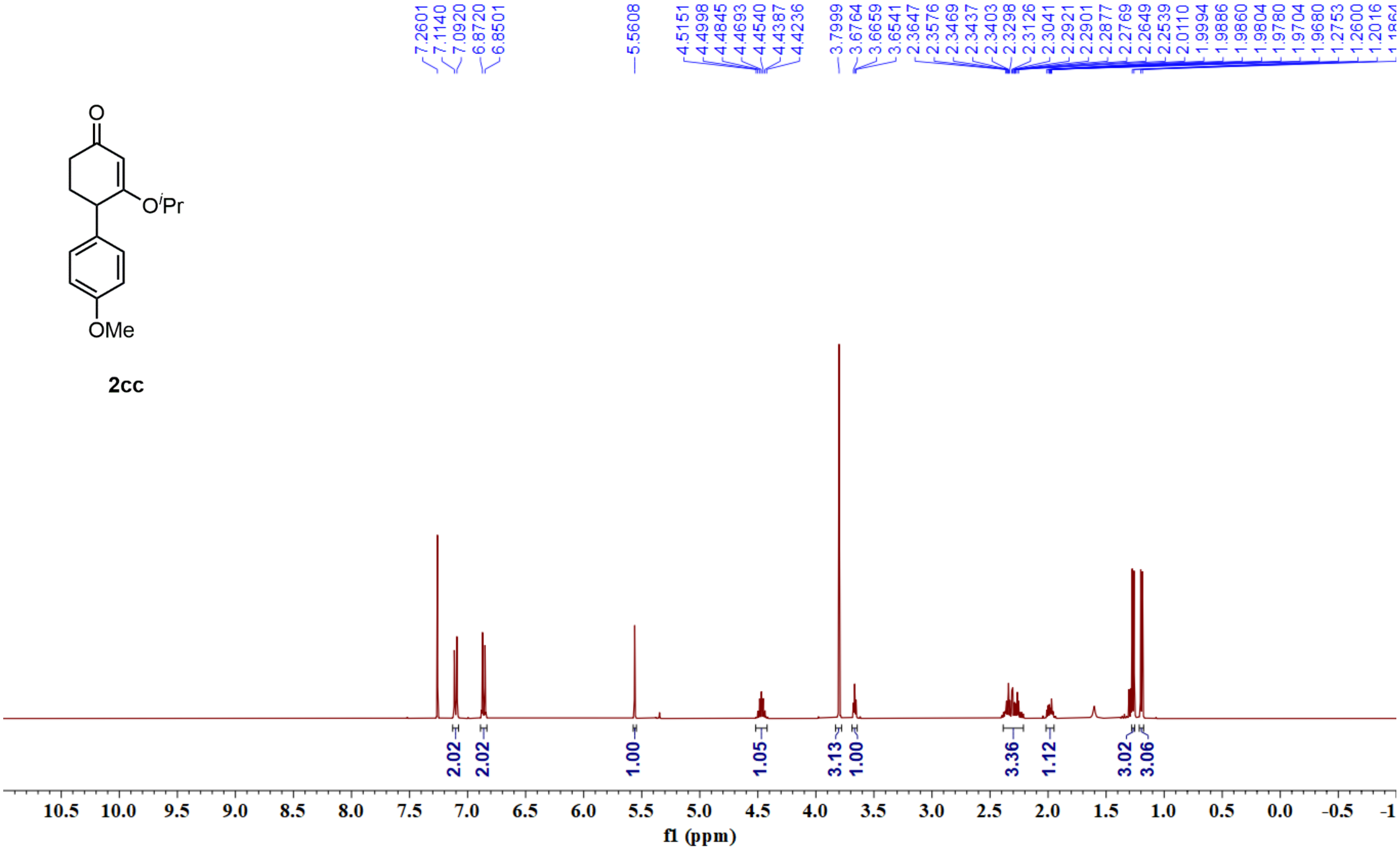

^{1}H NMR spectrum of compound 2cc

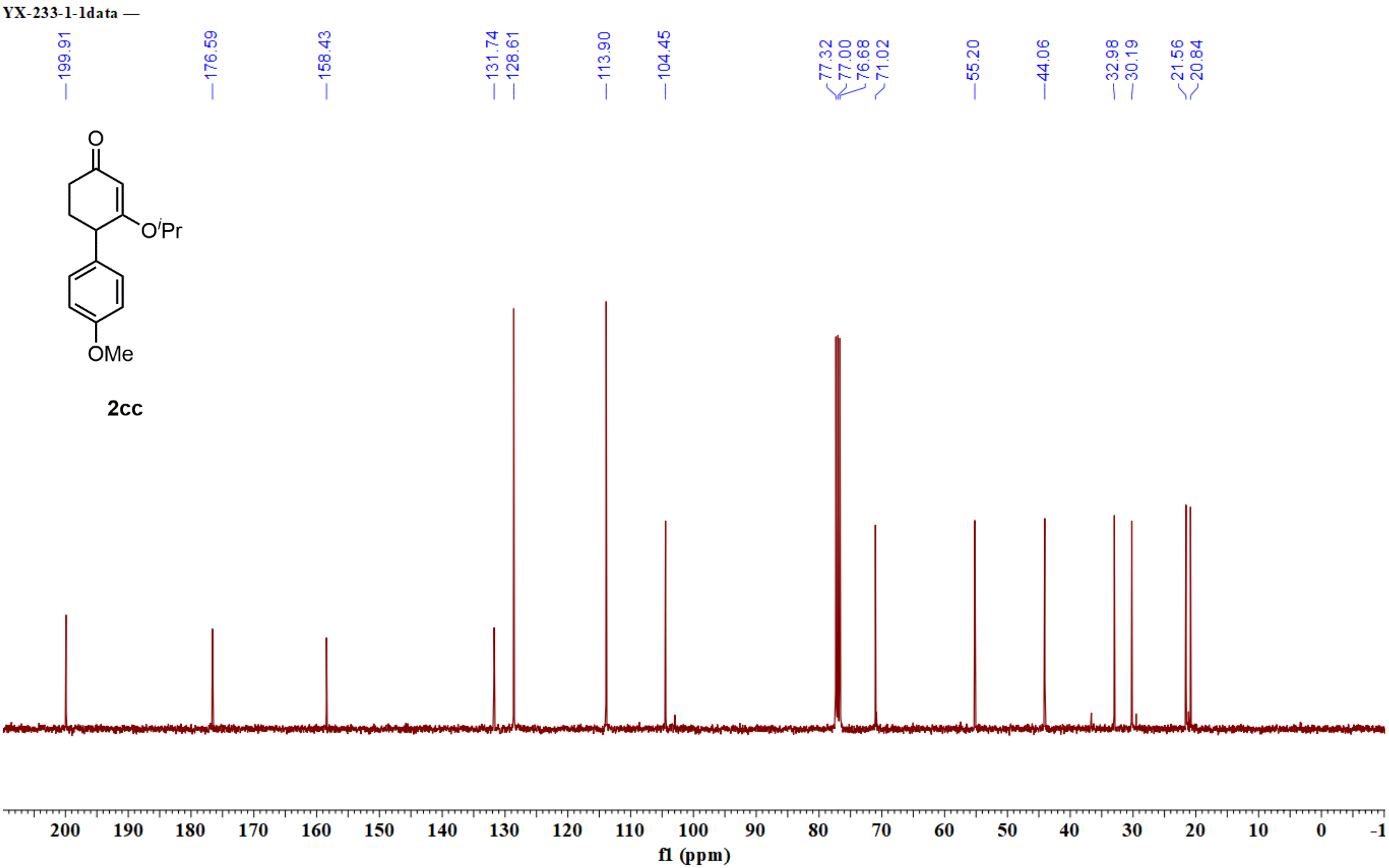

^{13}C NMR spectrum of compound 2cc

YX-233-2-1data —

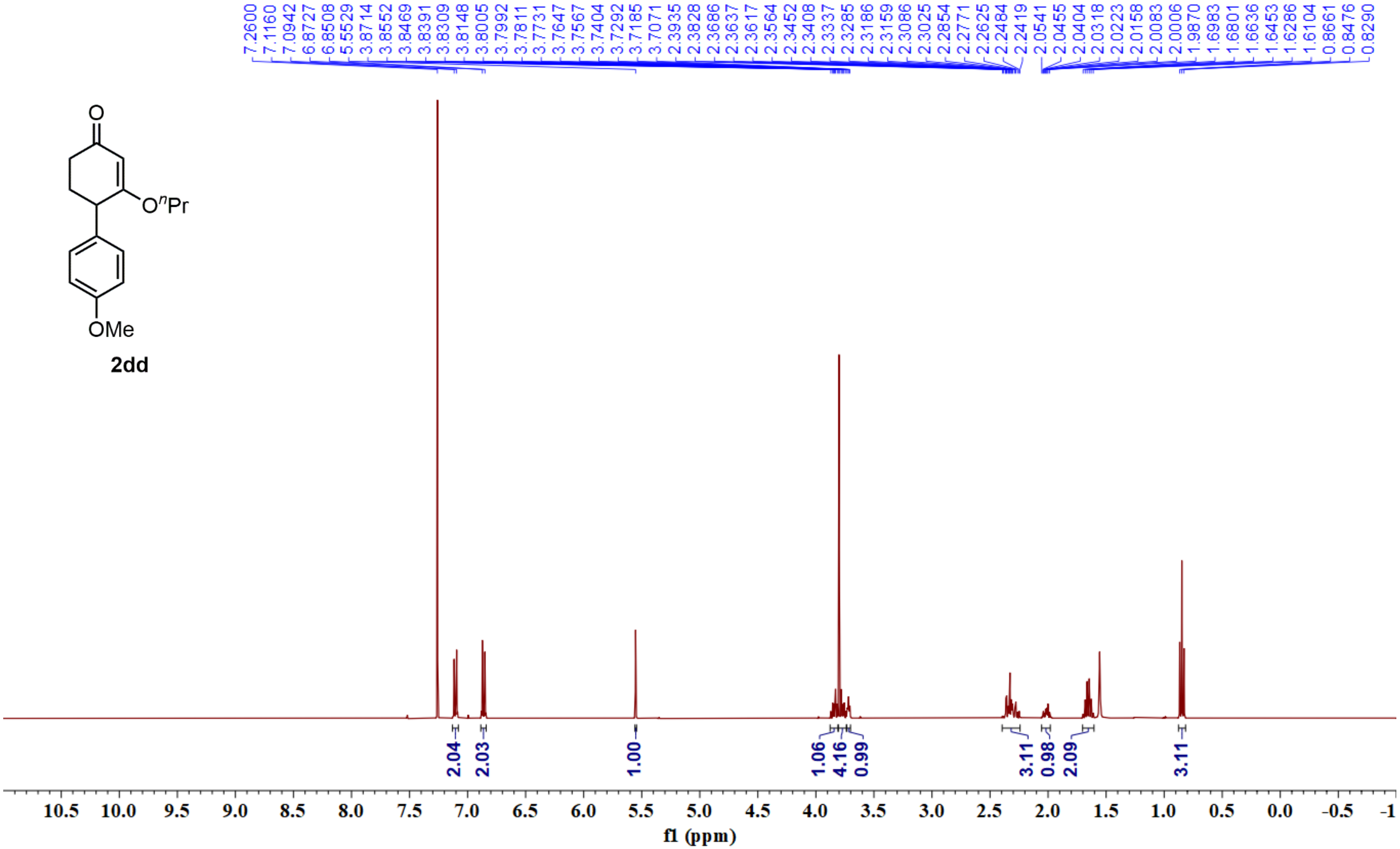

^{1}H NMR spectrum of compound 2dd

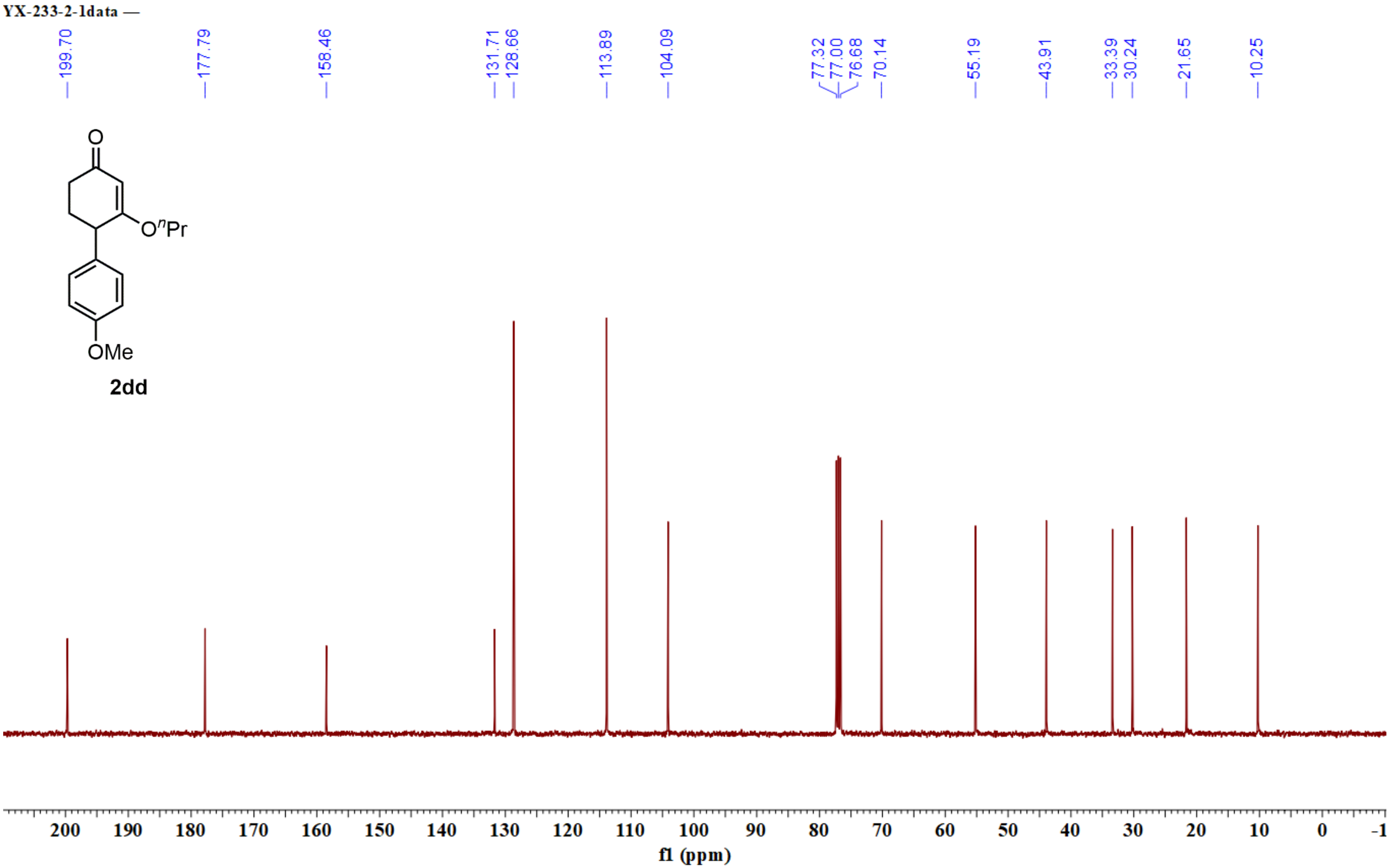

^{13}C NMR spectrum of compound 2dd

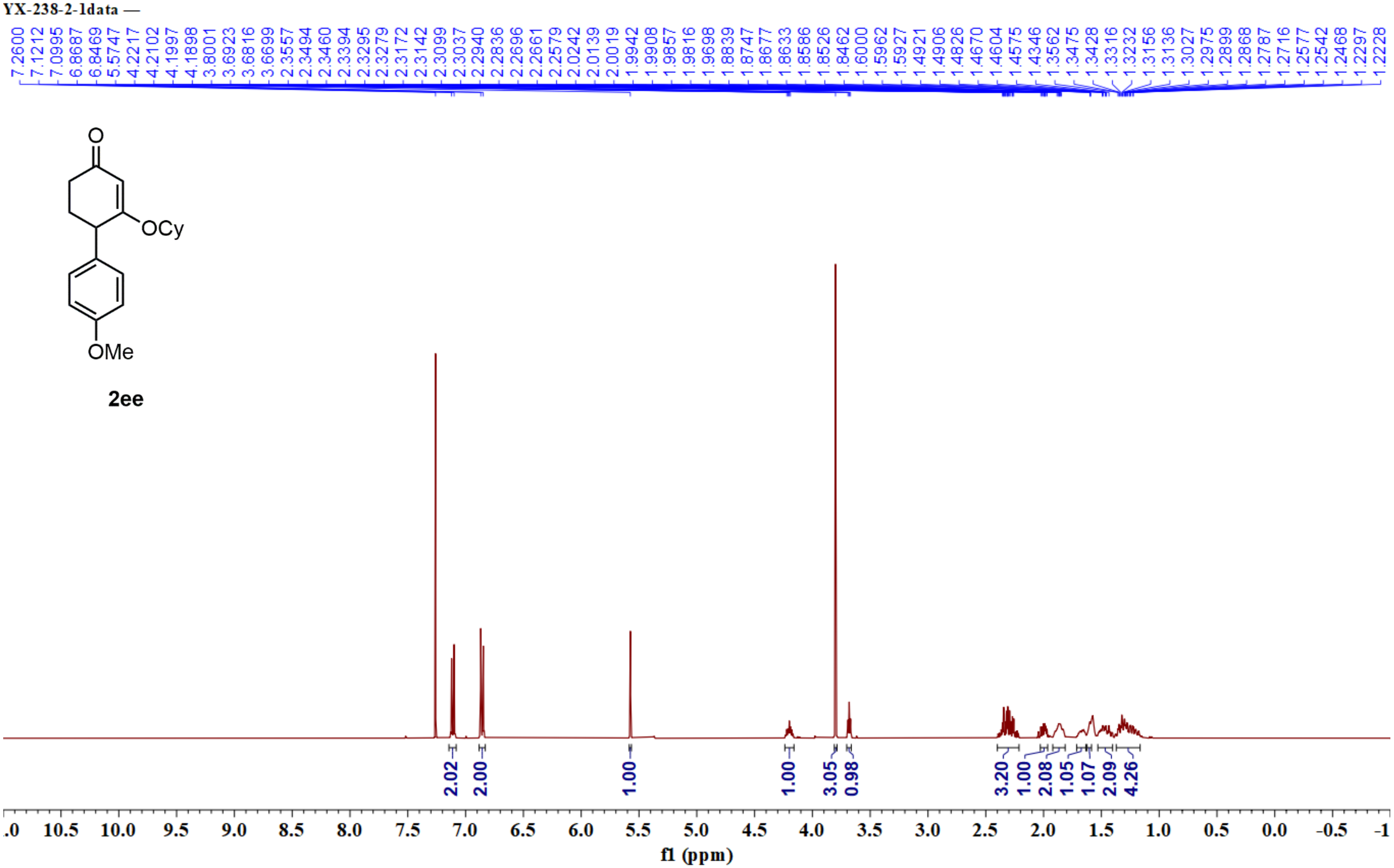

^{1}H NMR spectrum of compound 2cc

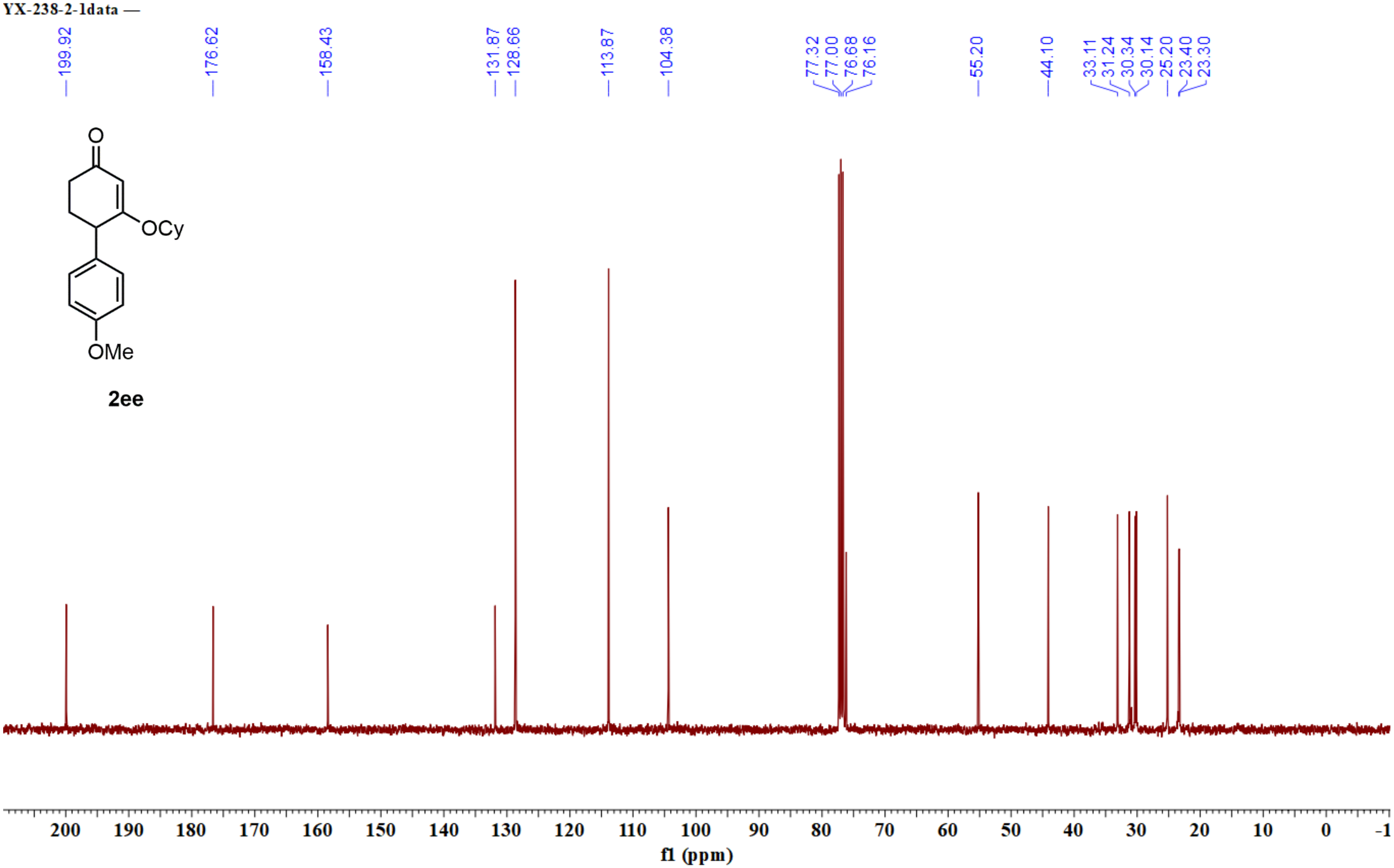

^{13}C NMR spectrum of compound 2cc

PROTON_01 — —YX-238-3-1NPhMe—

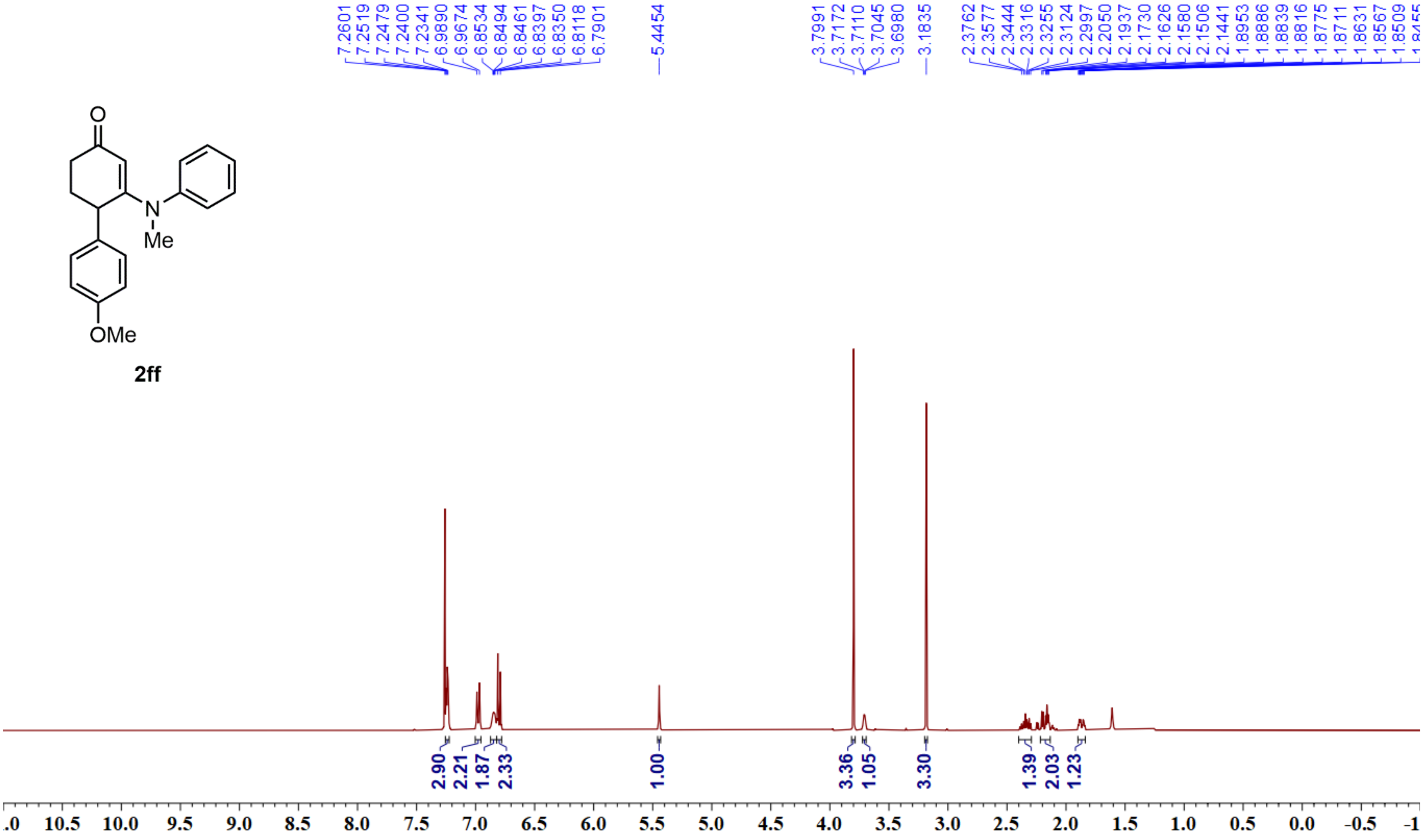

^{1}H NMR spectrum of compound 2ff

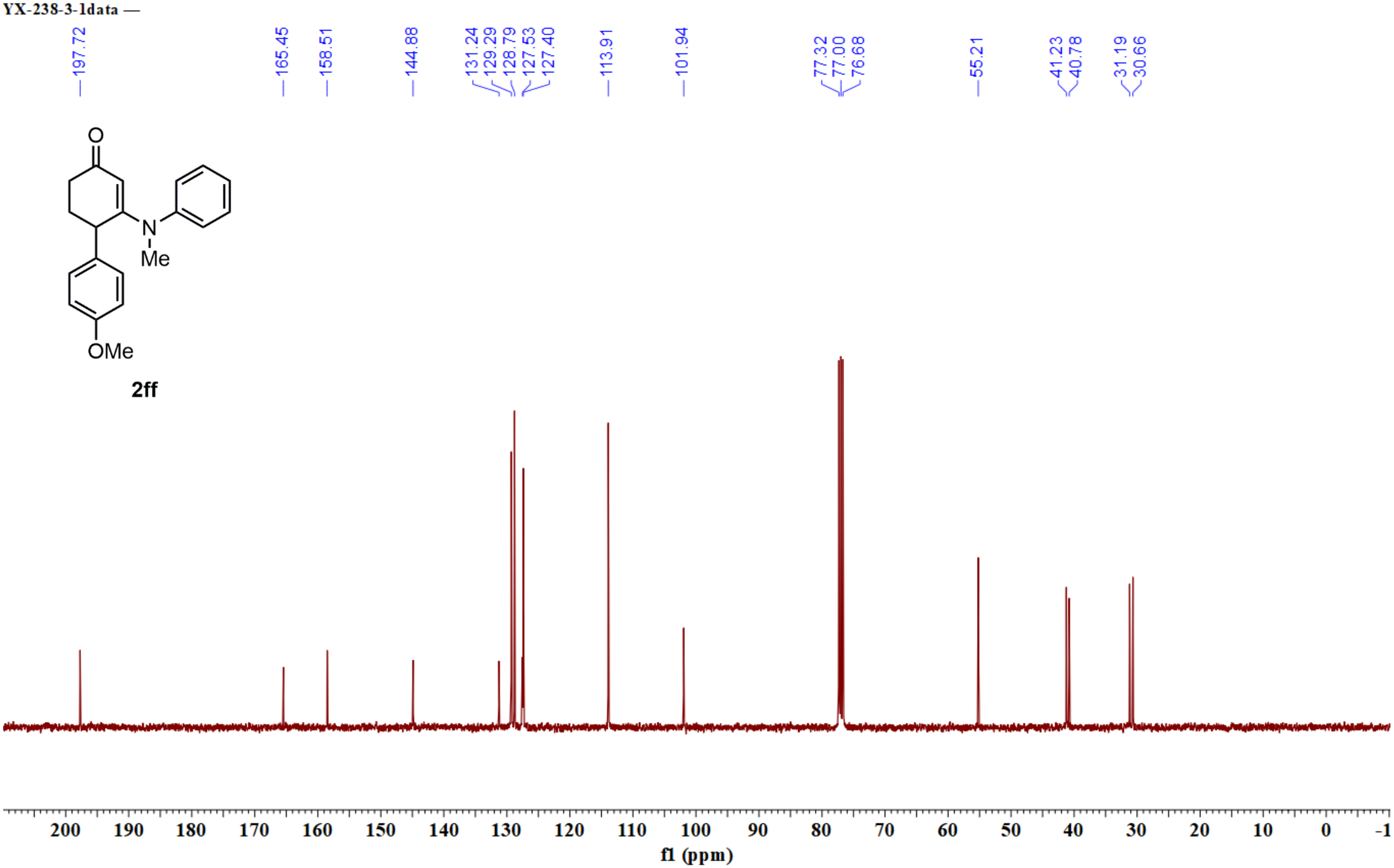

^{13}C NMR spectrum of compound 2ff

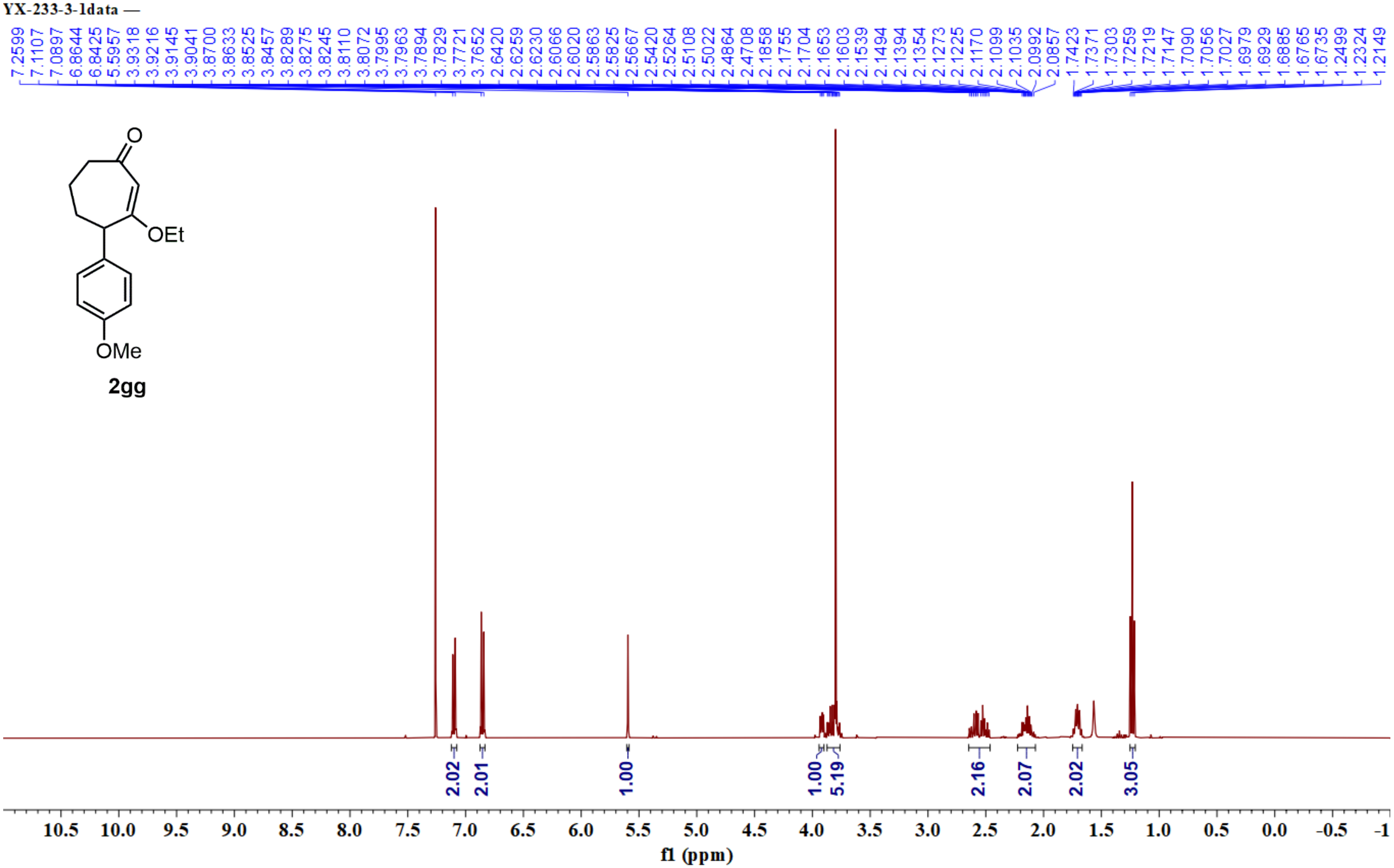

^{1}H NMR spectrum of compound 2gg

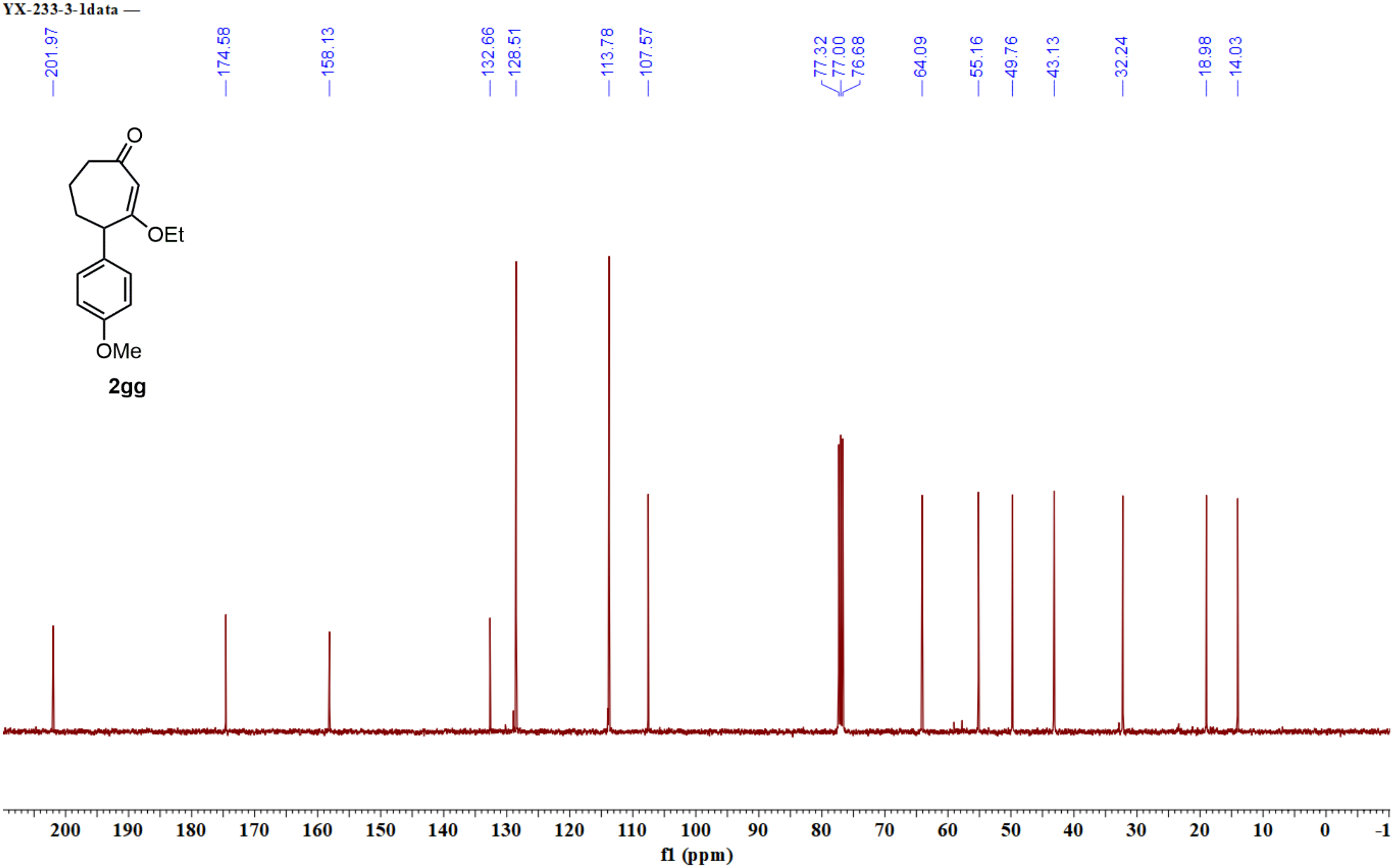

^{13}C NMR spectrum of compound 2gg

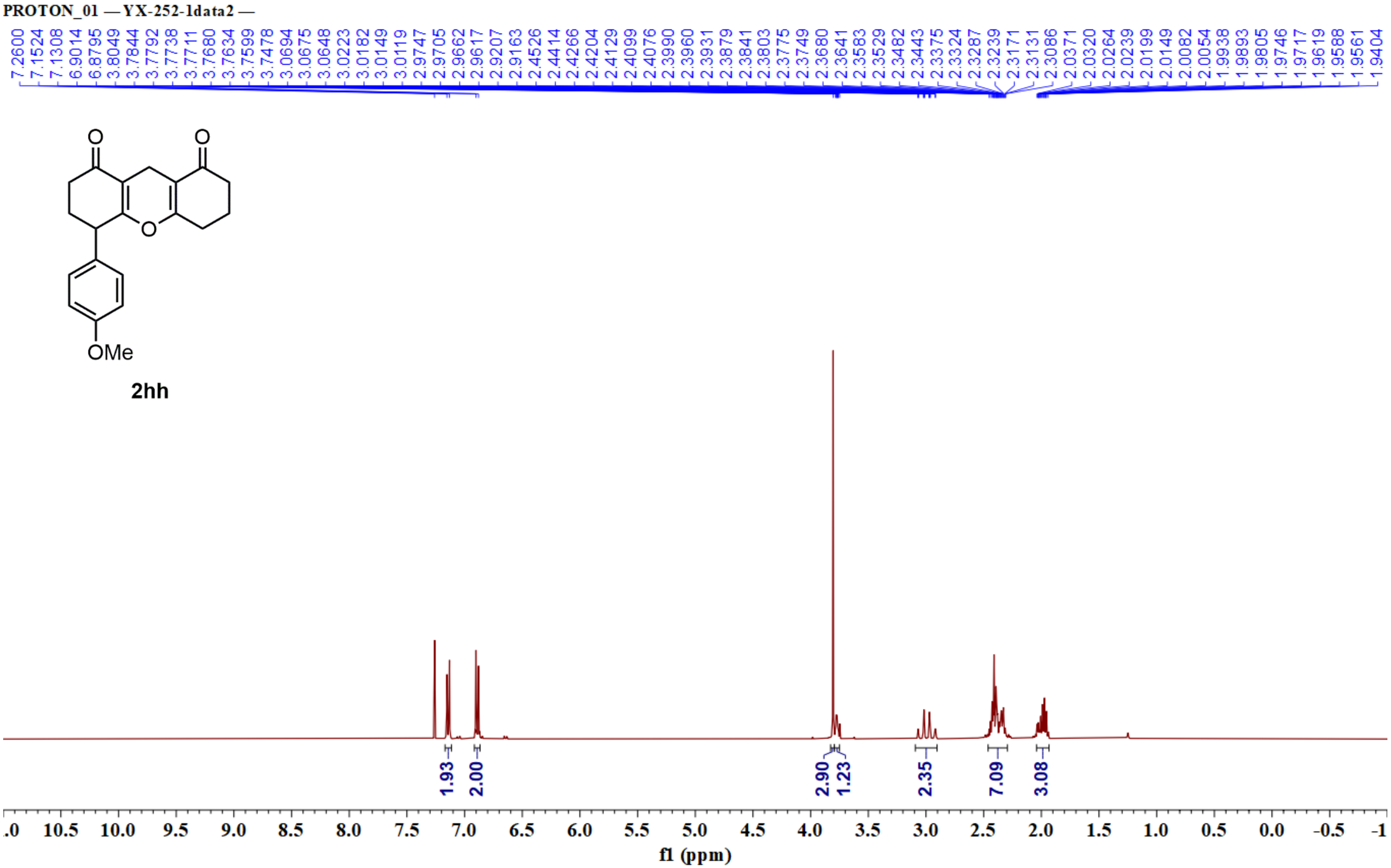

^{1}H NMR spectrum of compound 2hh

CARBON_01 —YX-252-1data2 —

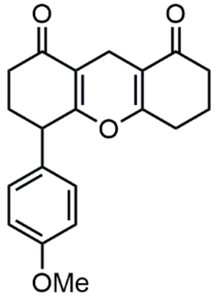

2hh

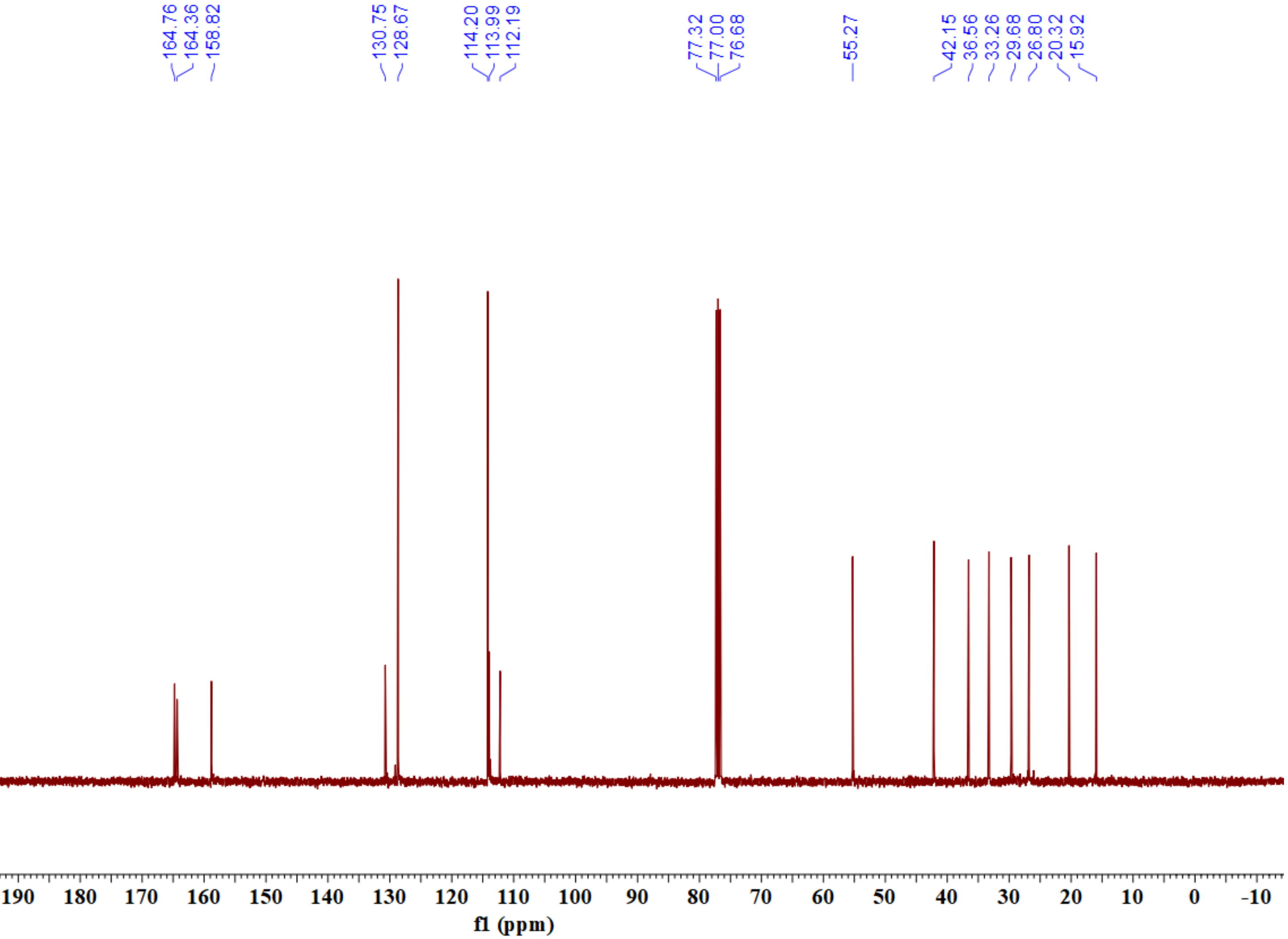

^{13}C NMR spectrum of compound 2hh

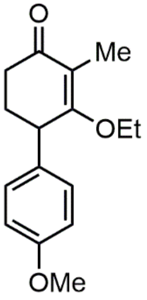

2ii

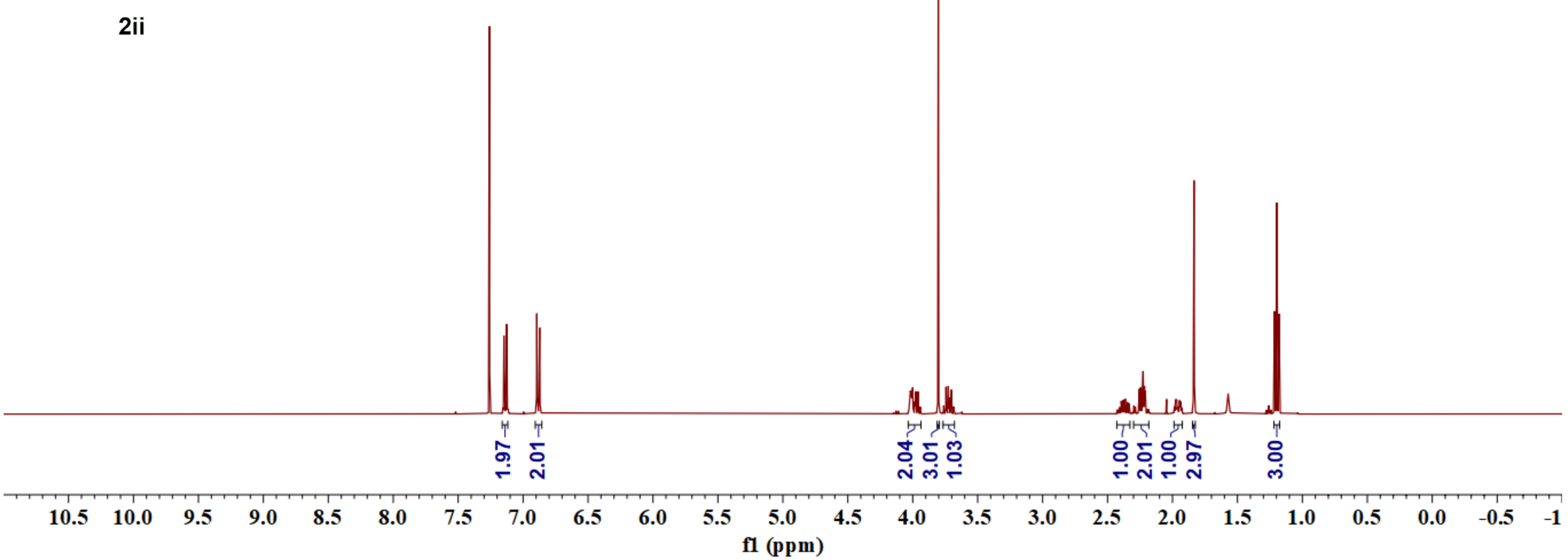

^{1}H NMR spectrum of compound 2ii

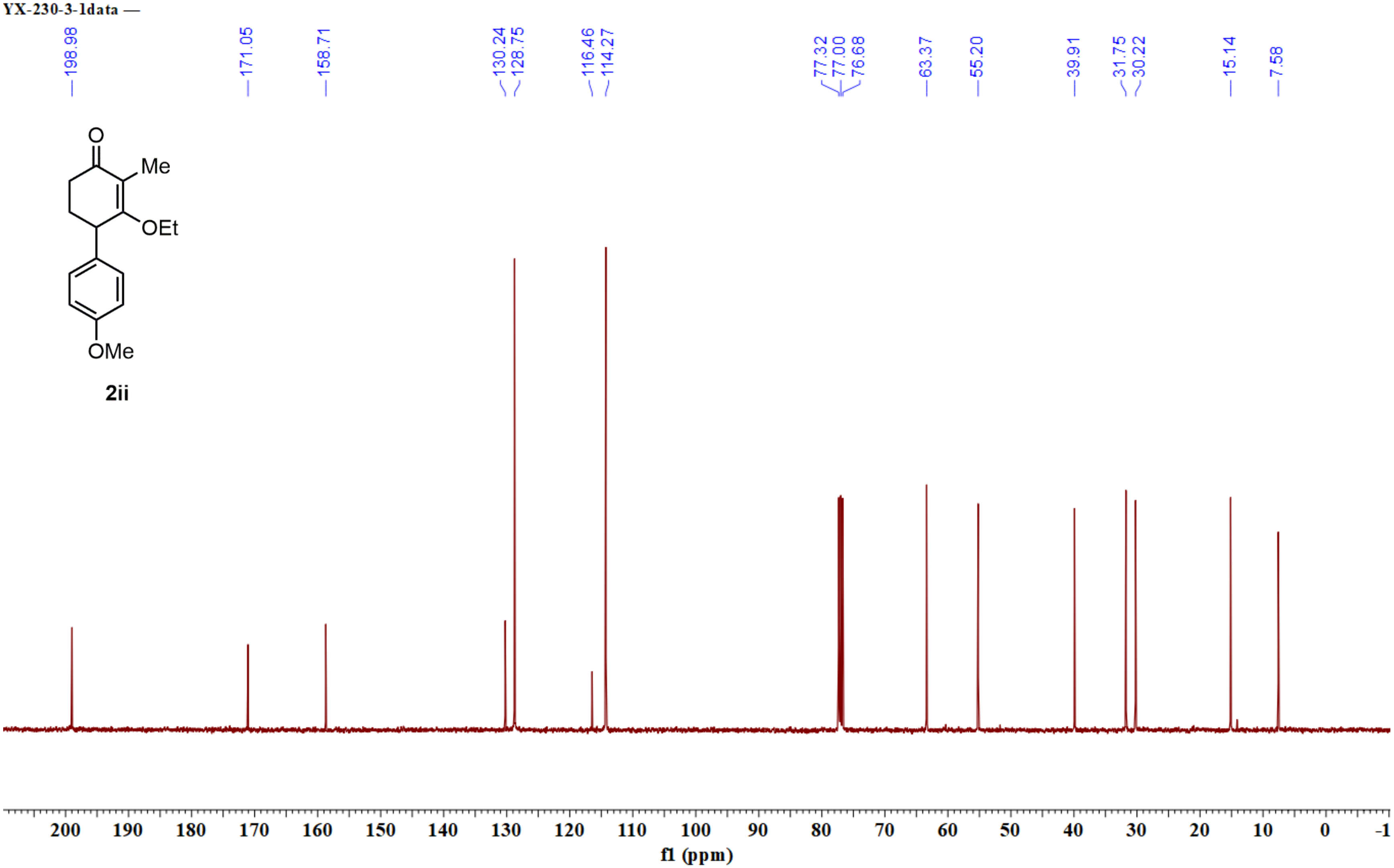

^{13}C NMR spectrum of compound 2ii

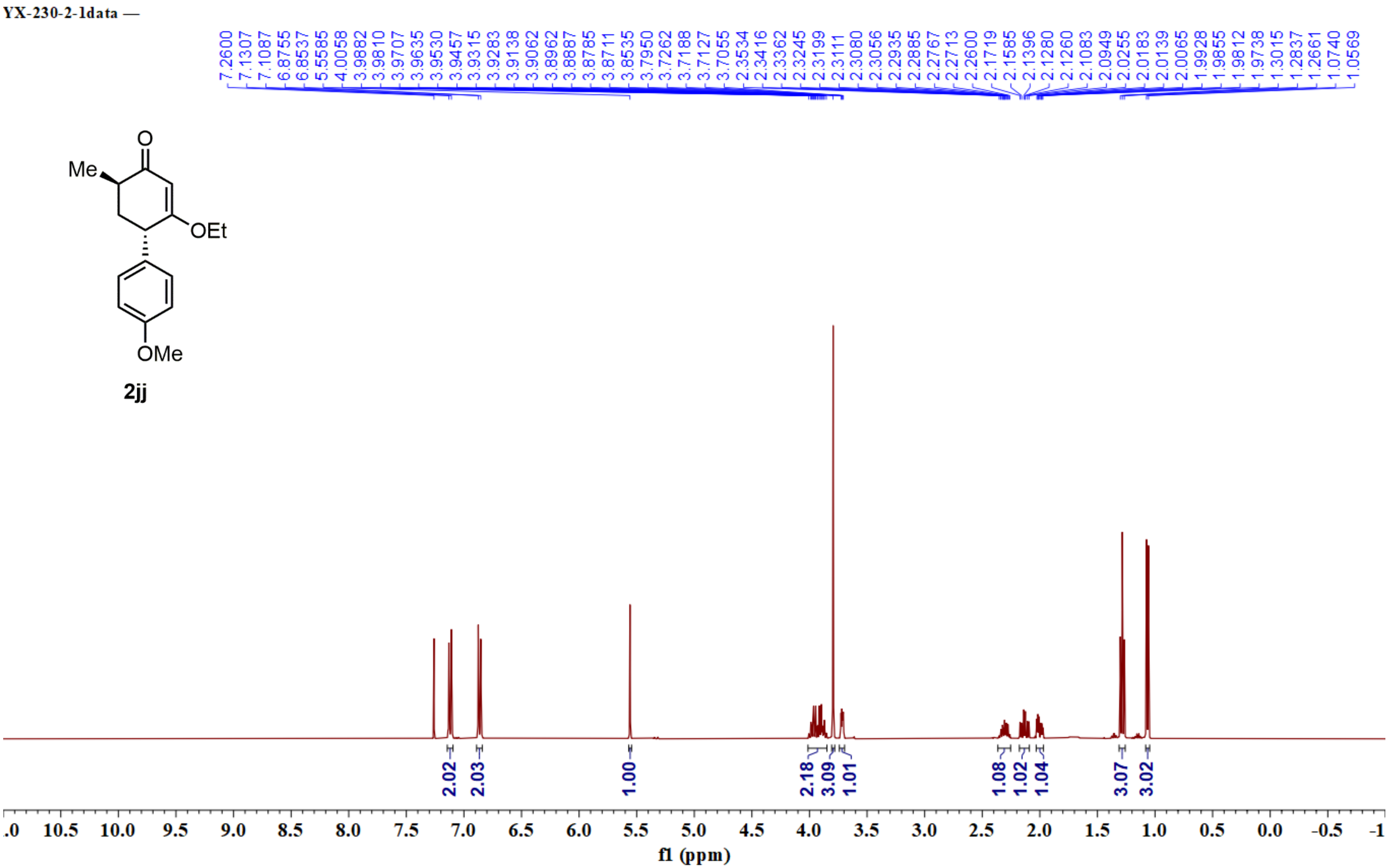

^{1}H NMR spectrum of compound 2jj

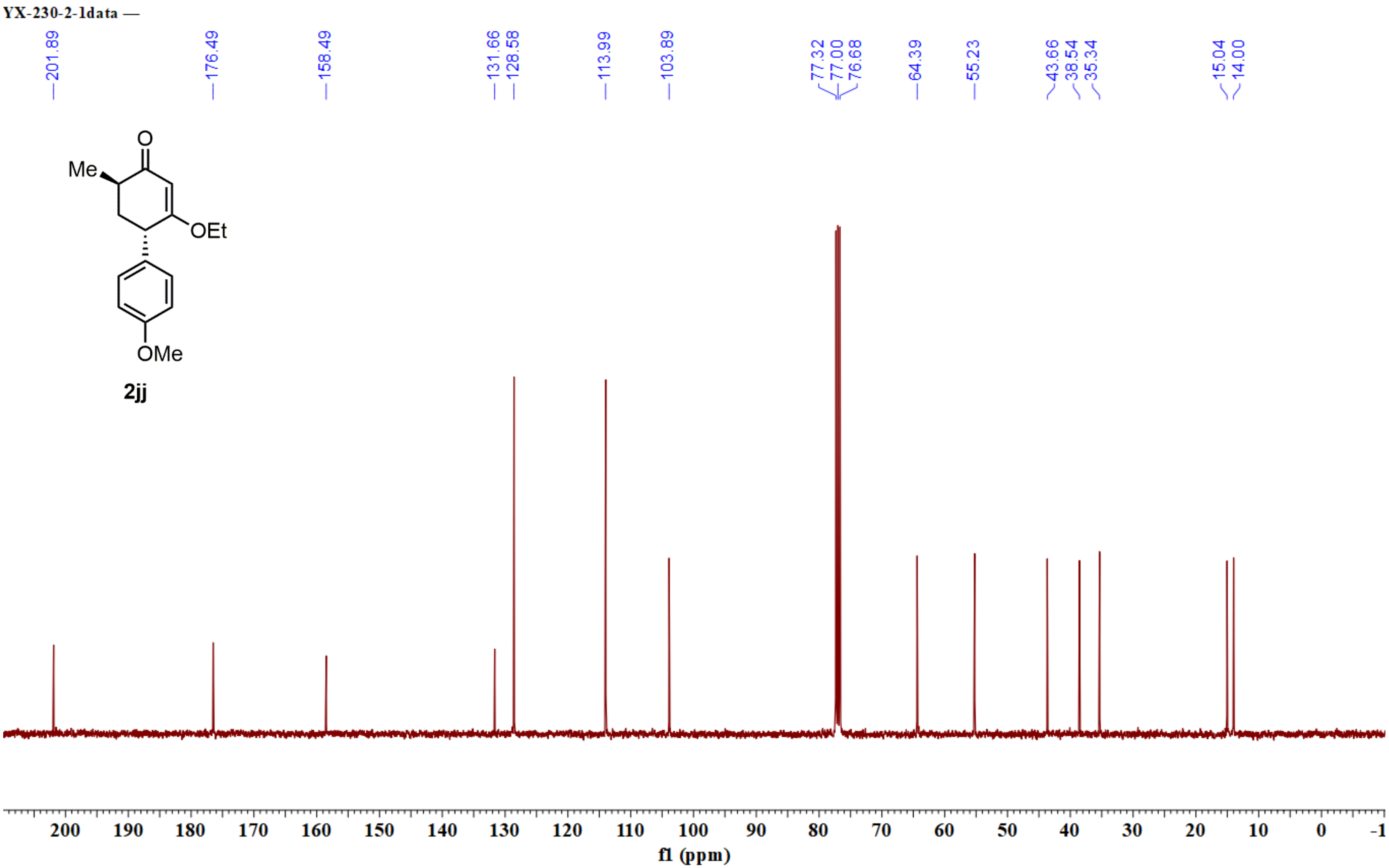

^{13}C NMR spectrum of compound 2jj

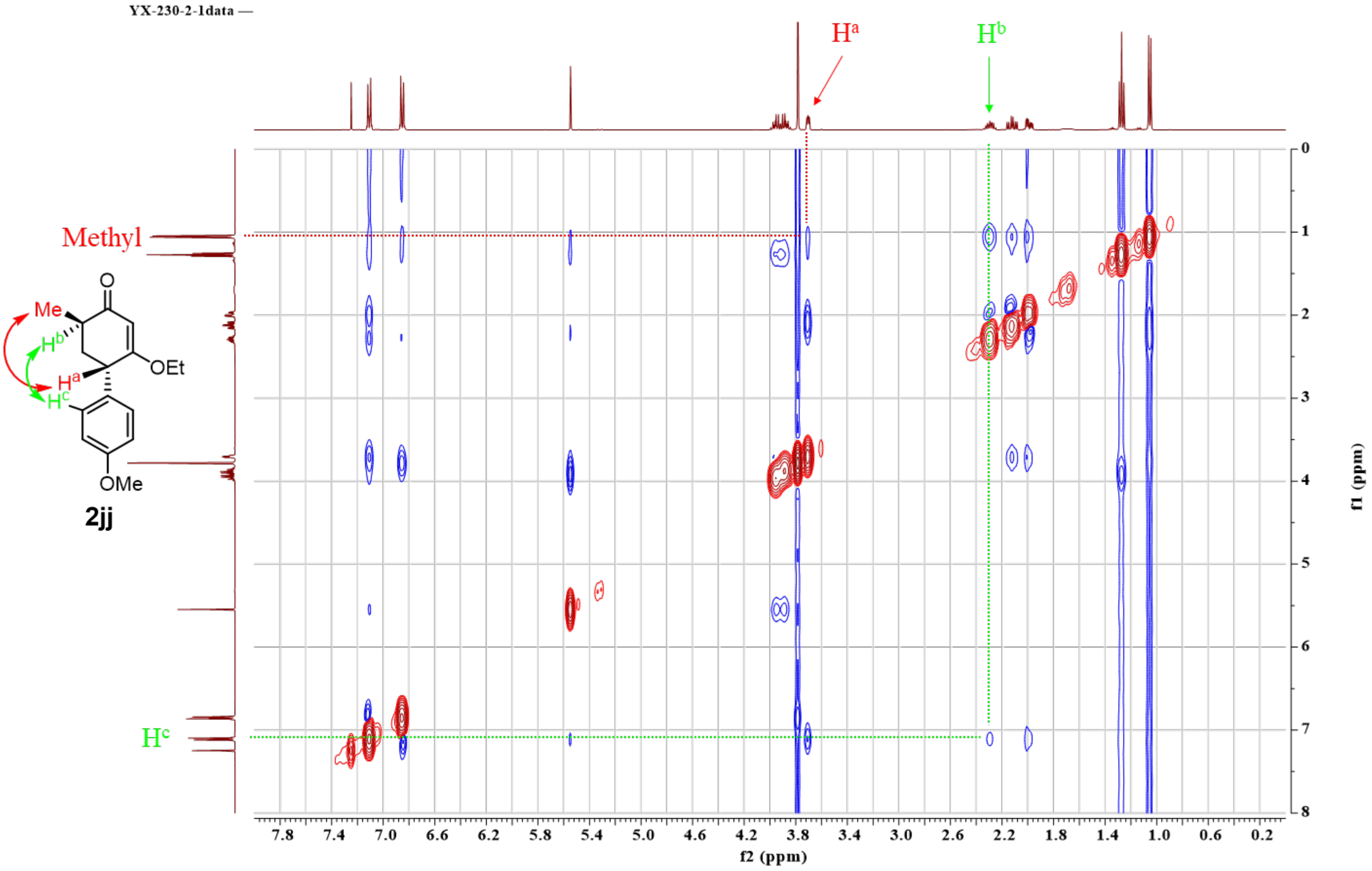

NOESY spectrum of compound 2jj

YX-230-2-2dr 1 —

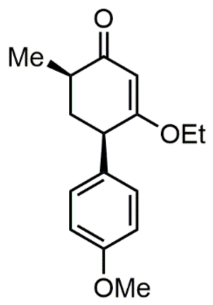

2jj'

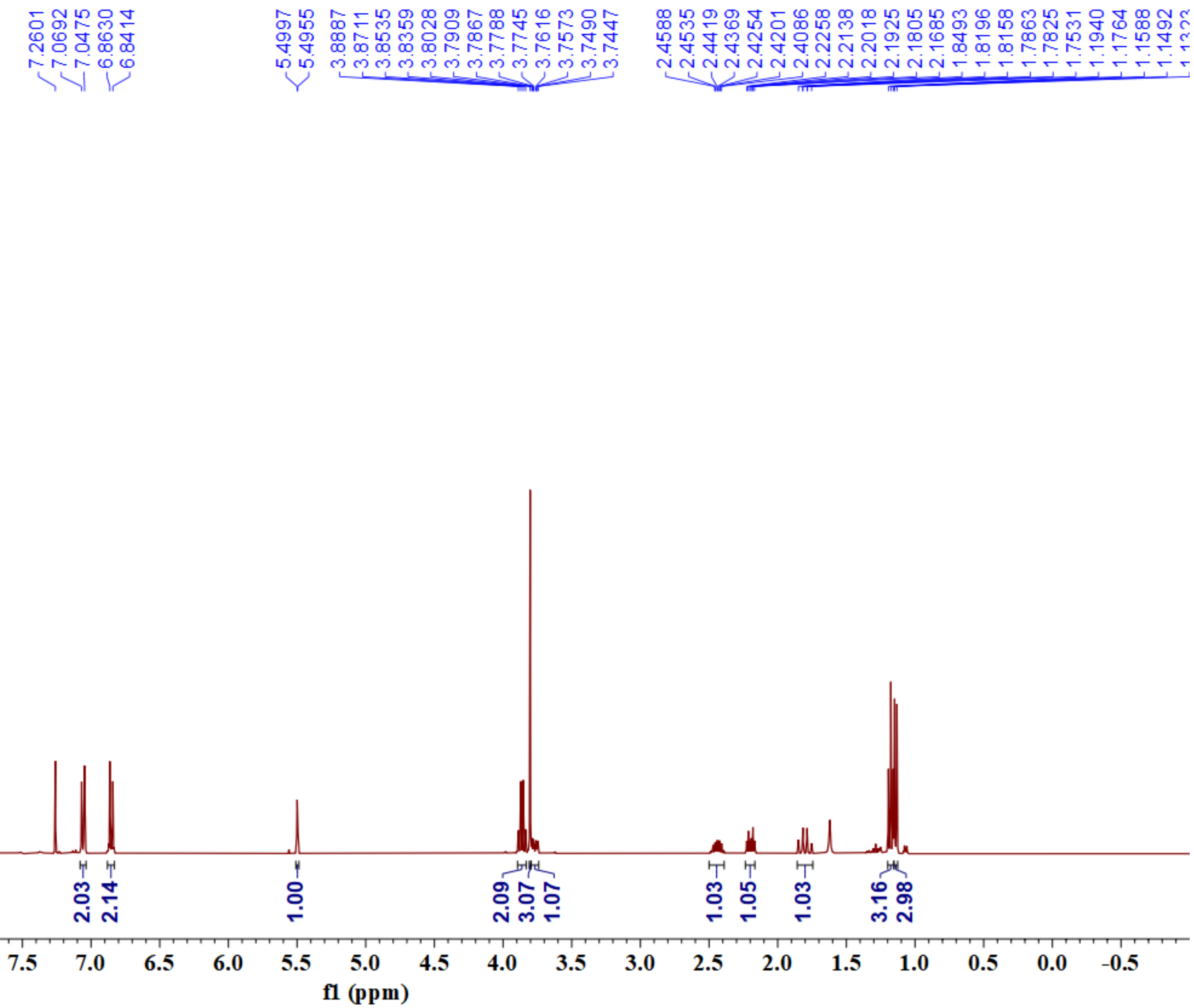

^{1}H NMR spectrum of compound 2jj'

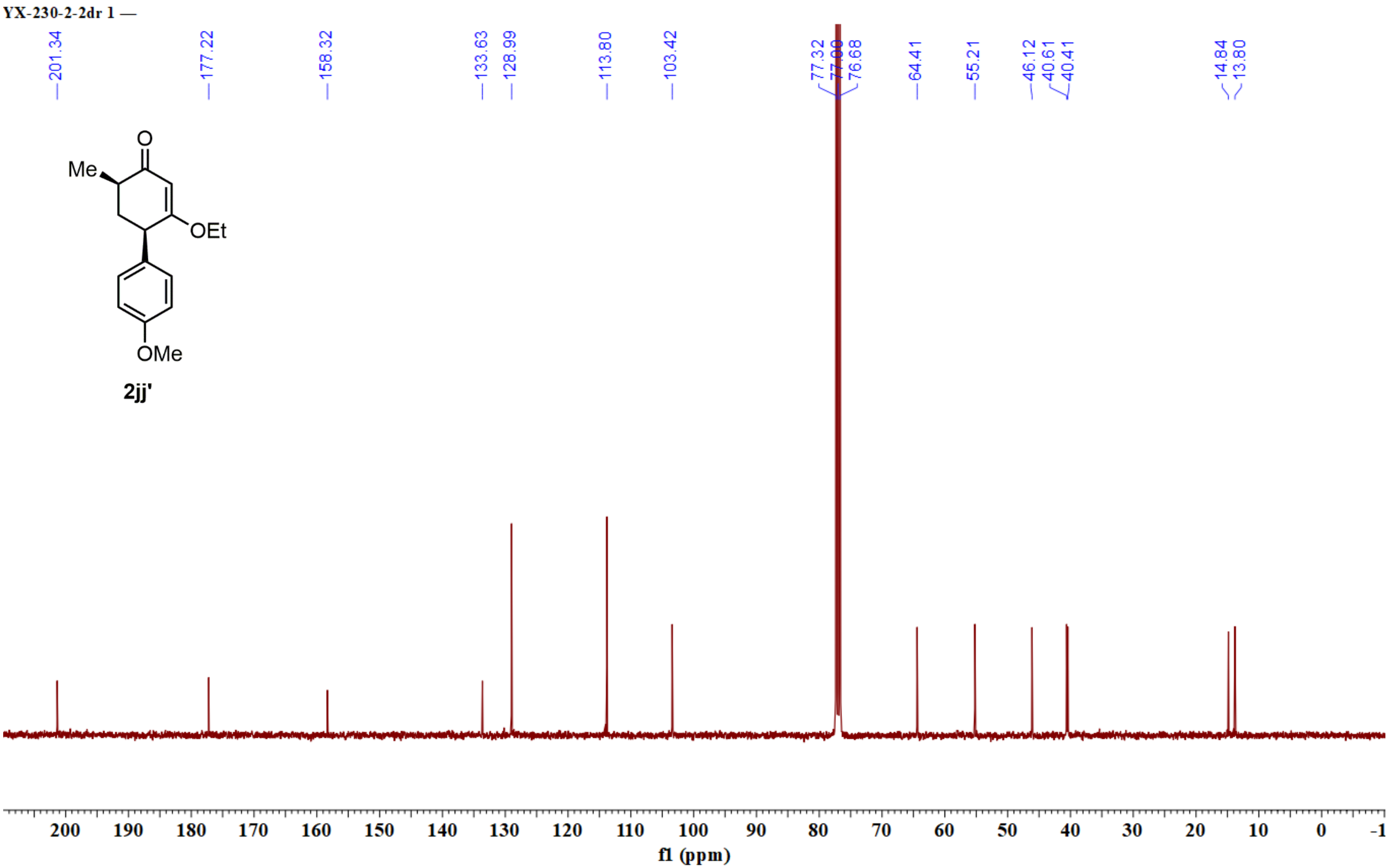

^{13}C NMR spectrum of compound 2jj'

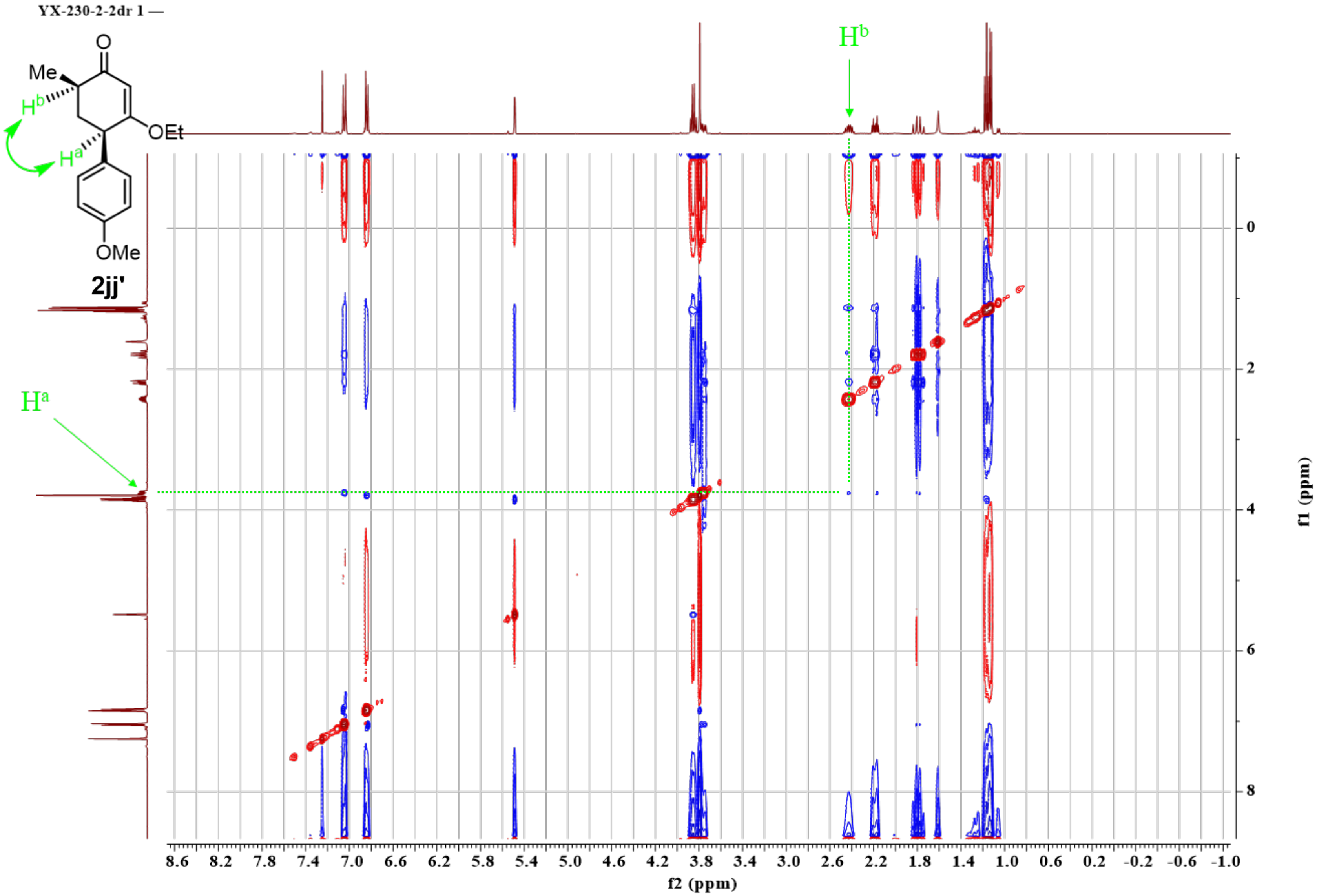

NOESY spectrum of compound 2jj'

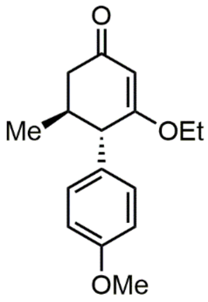

2kk

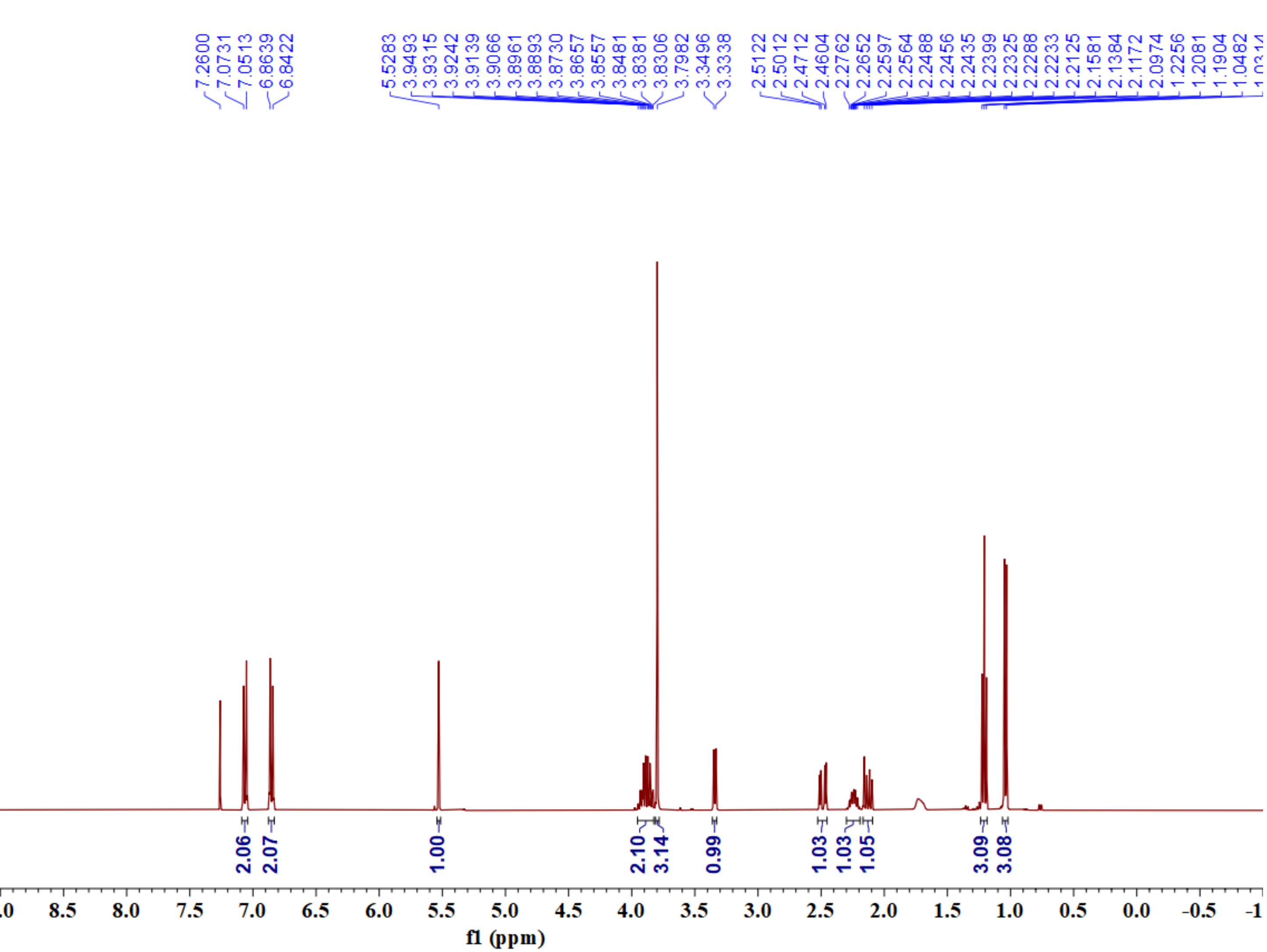

^{1}H NMR spectrum of compound 2kk

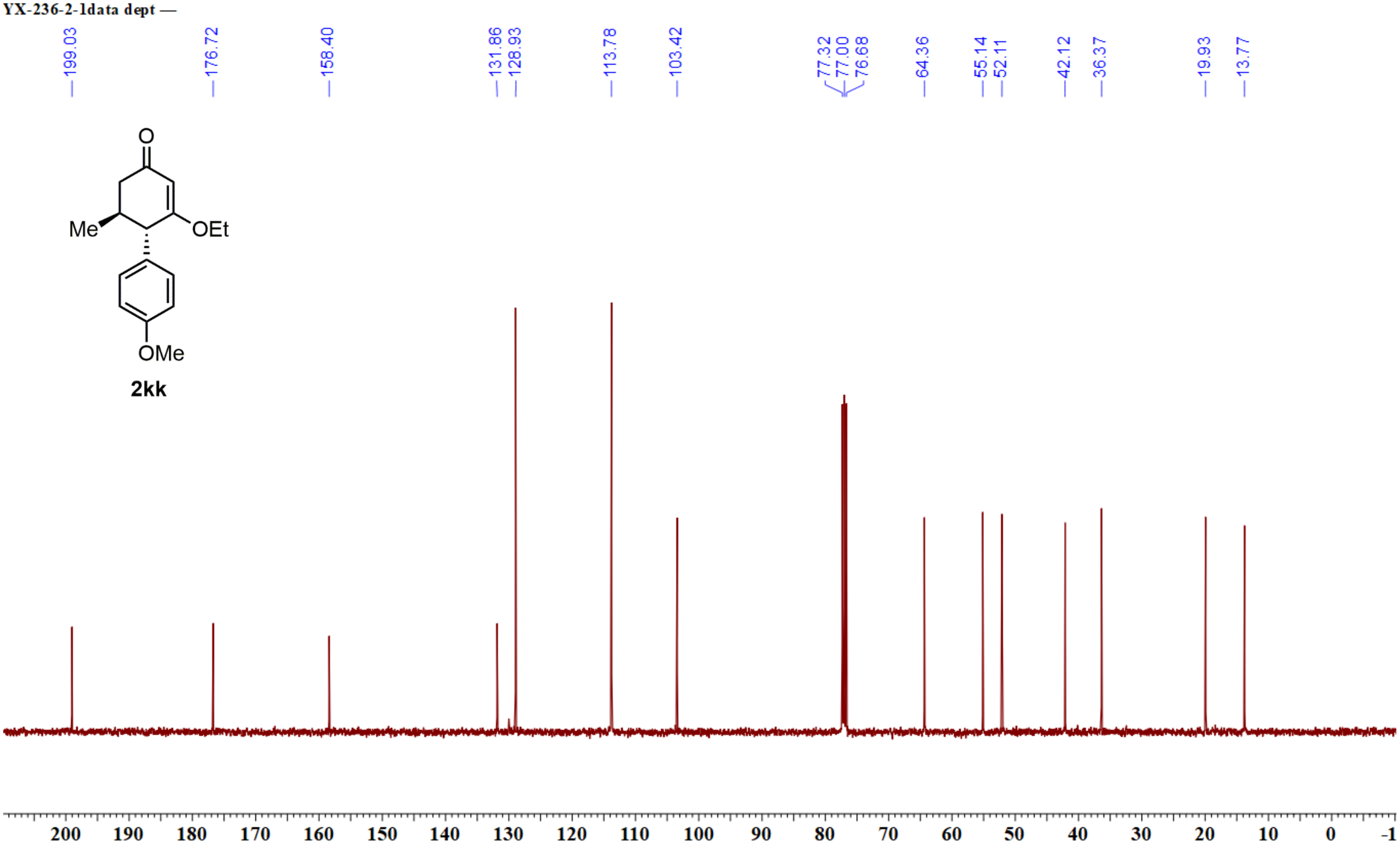

^{13}C NMR spectrum of compound 2kk

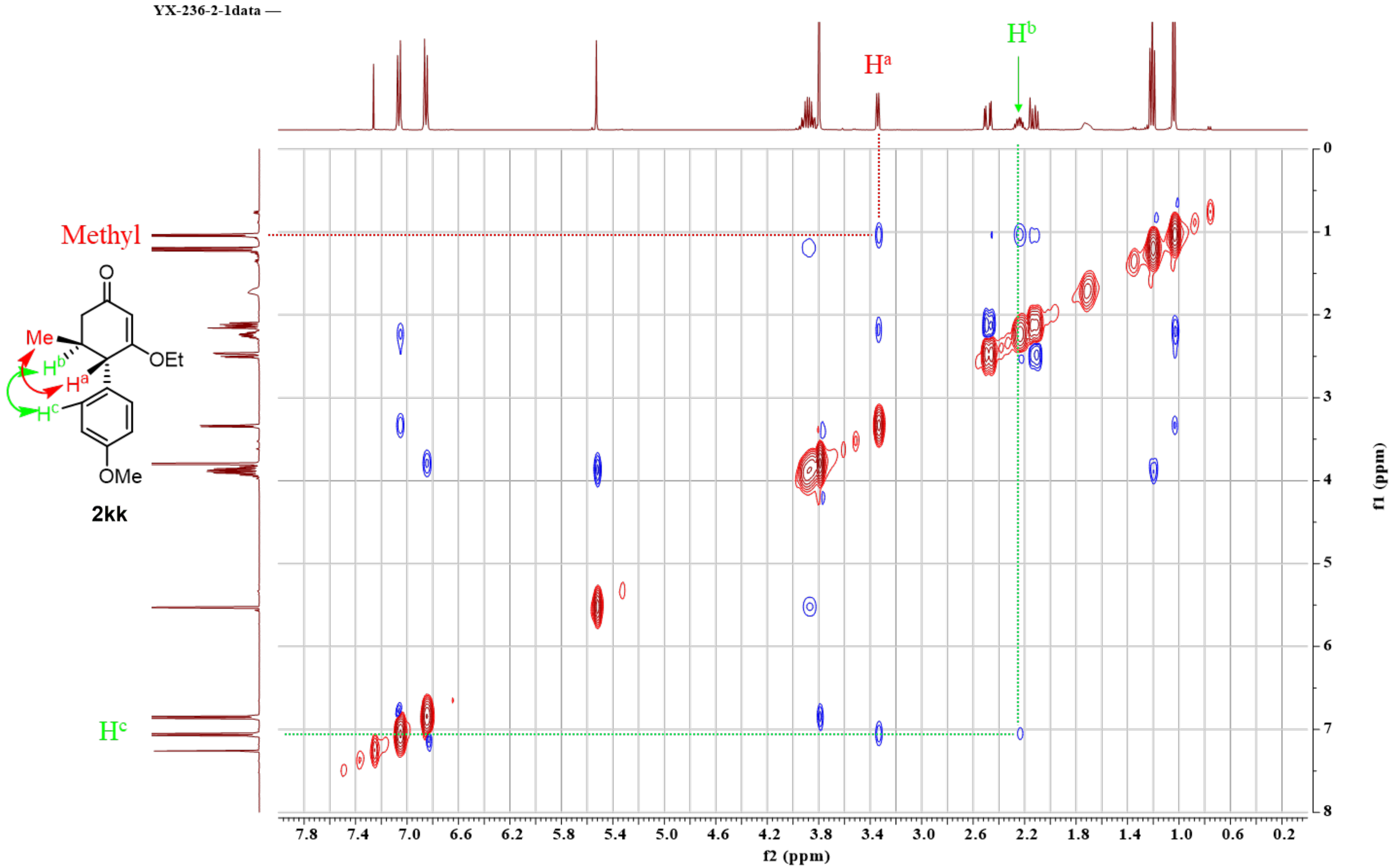

NOESY spectrum of compound 2kk

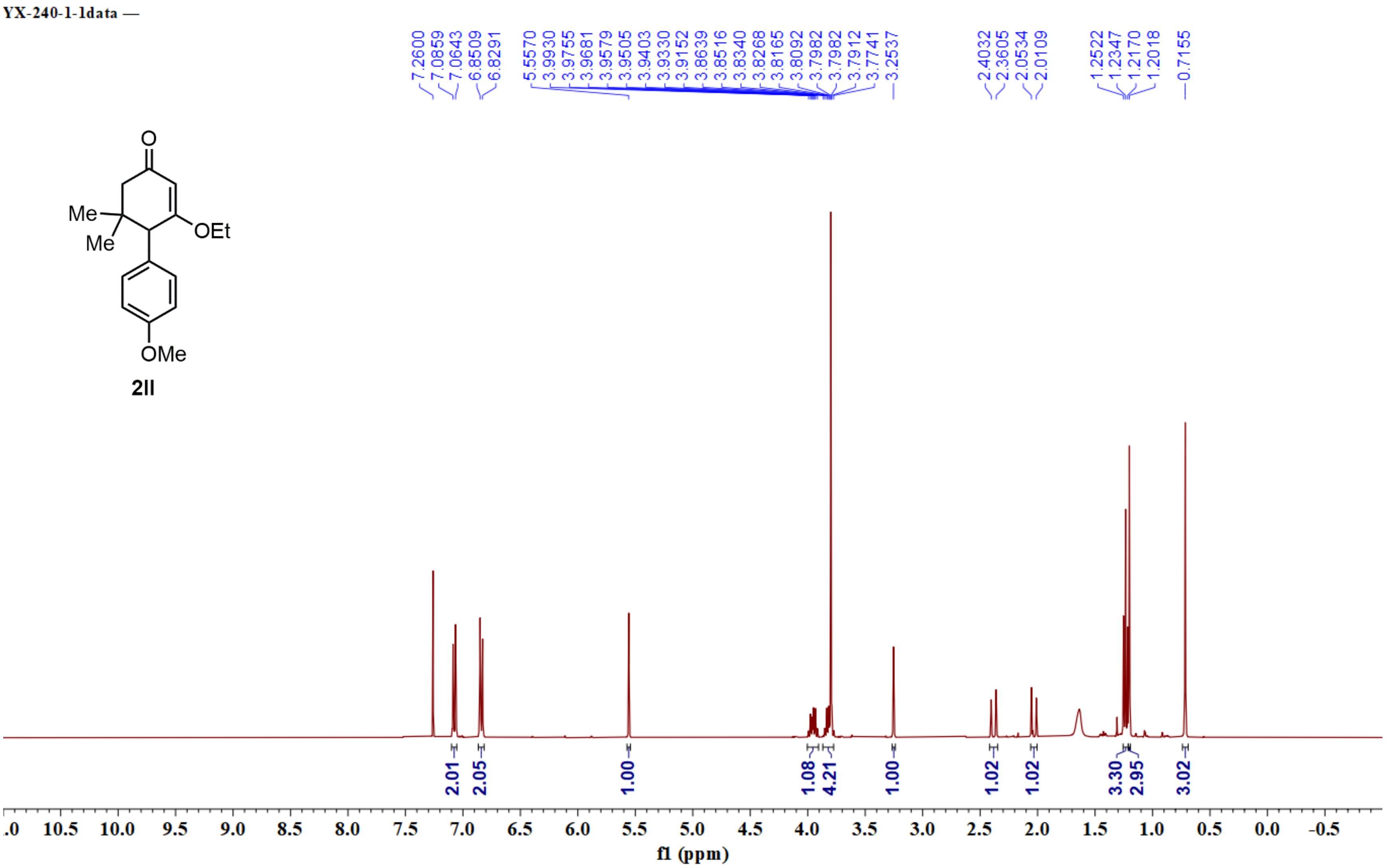

^{1}H NMR spectrum of compound 2ll

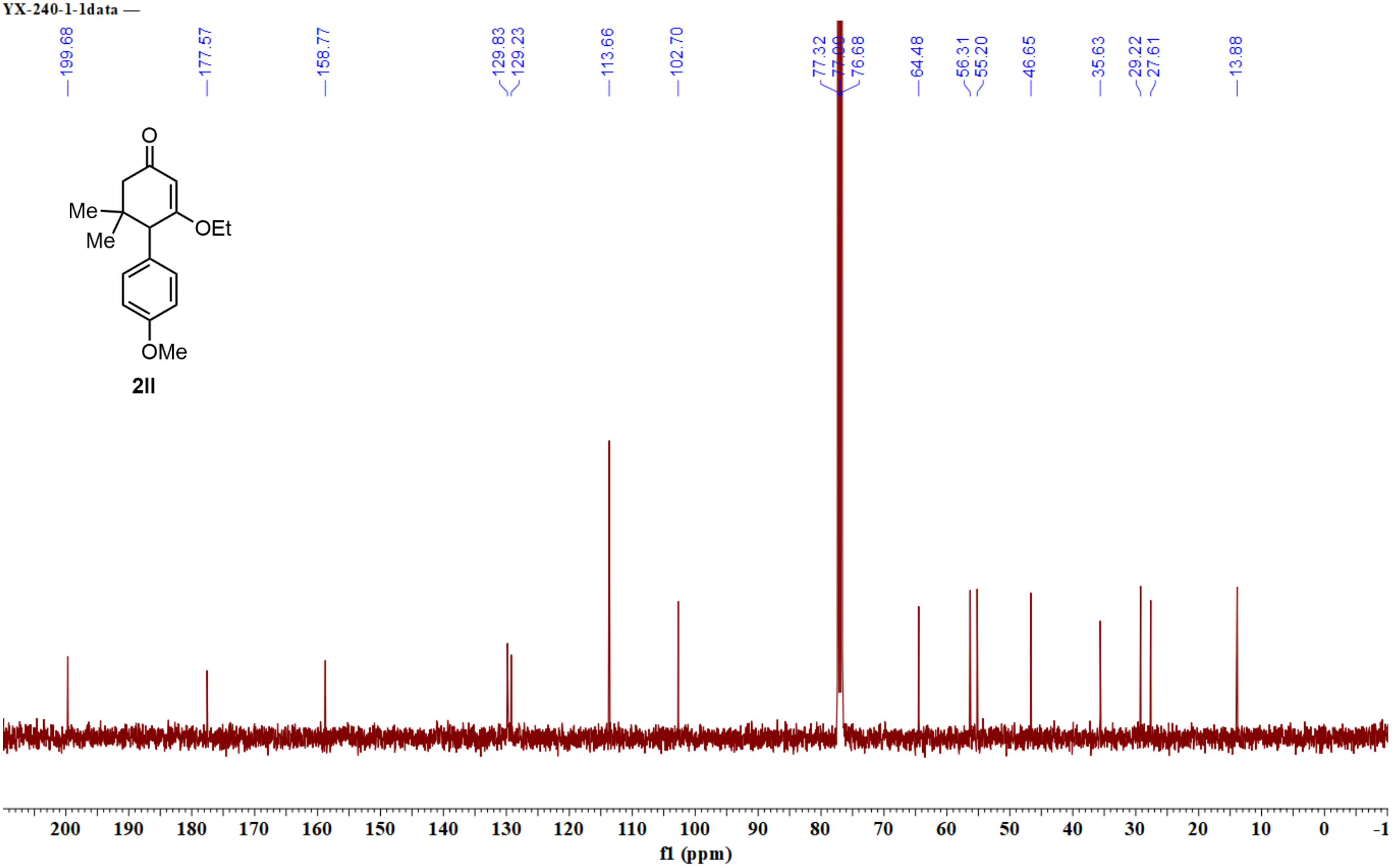

^{13}C NMR spectrum of compound 2ll

YX-224-2-1data2 —

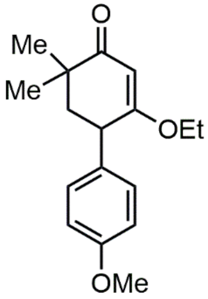

2mm

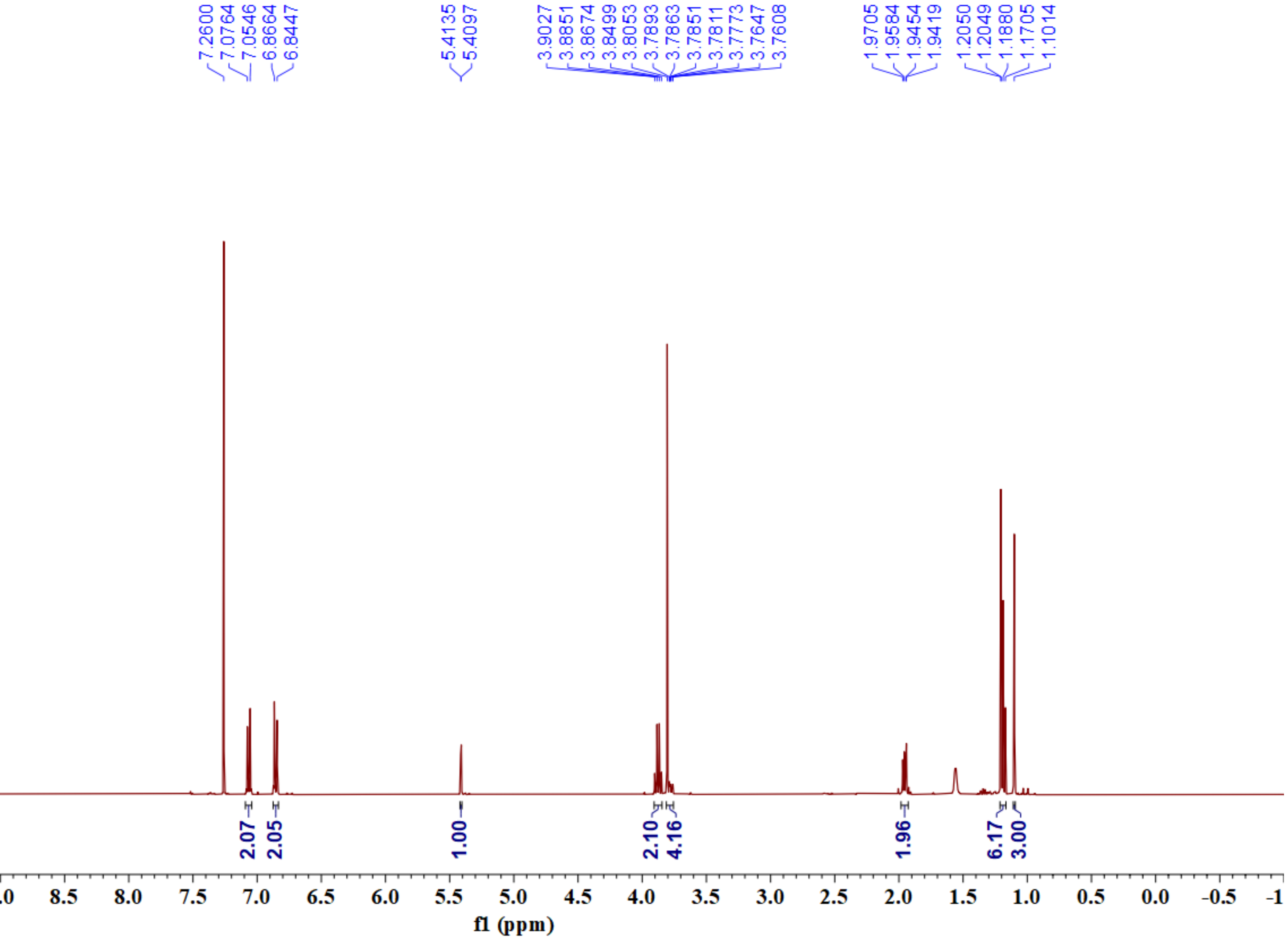

¹H NMR spectrum of compound 2mm

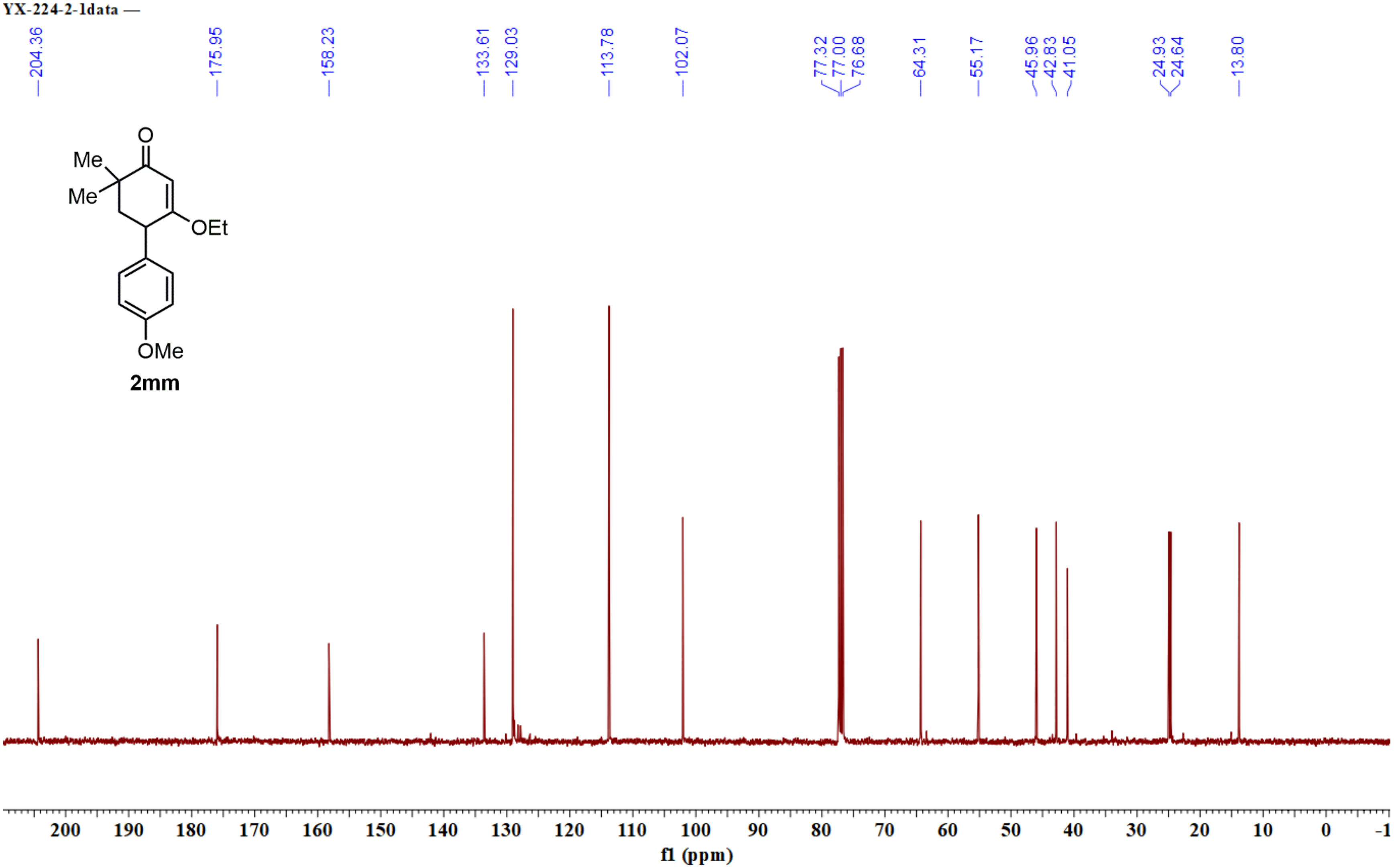

^{13}C NMR spectrum of compound 2mm

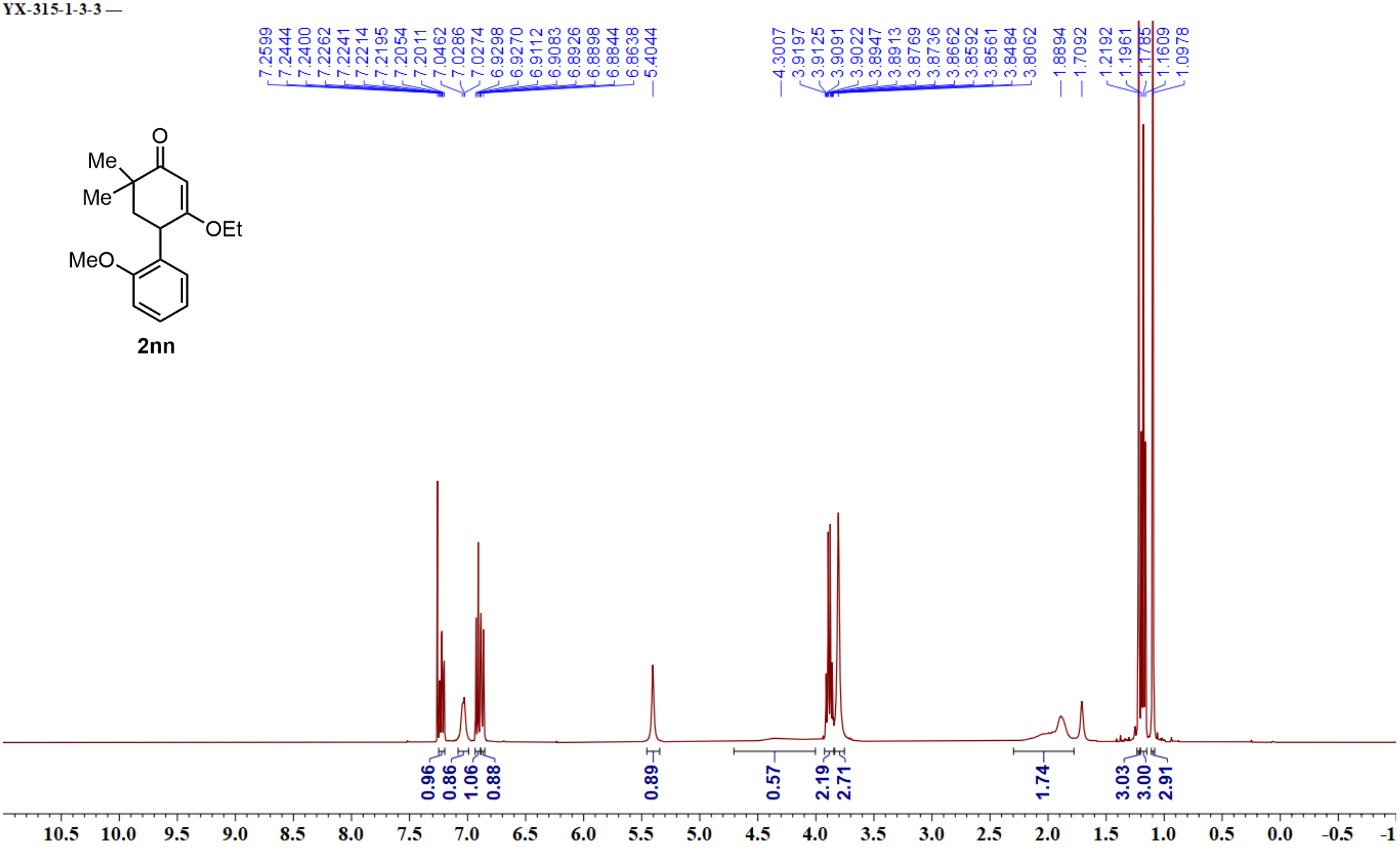

^{1}H NMR spectrum of compound 2nn

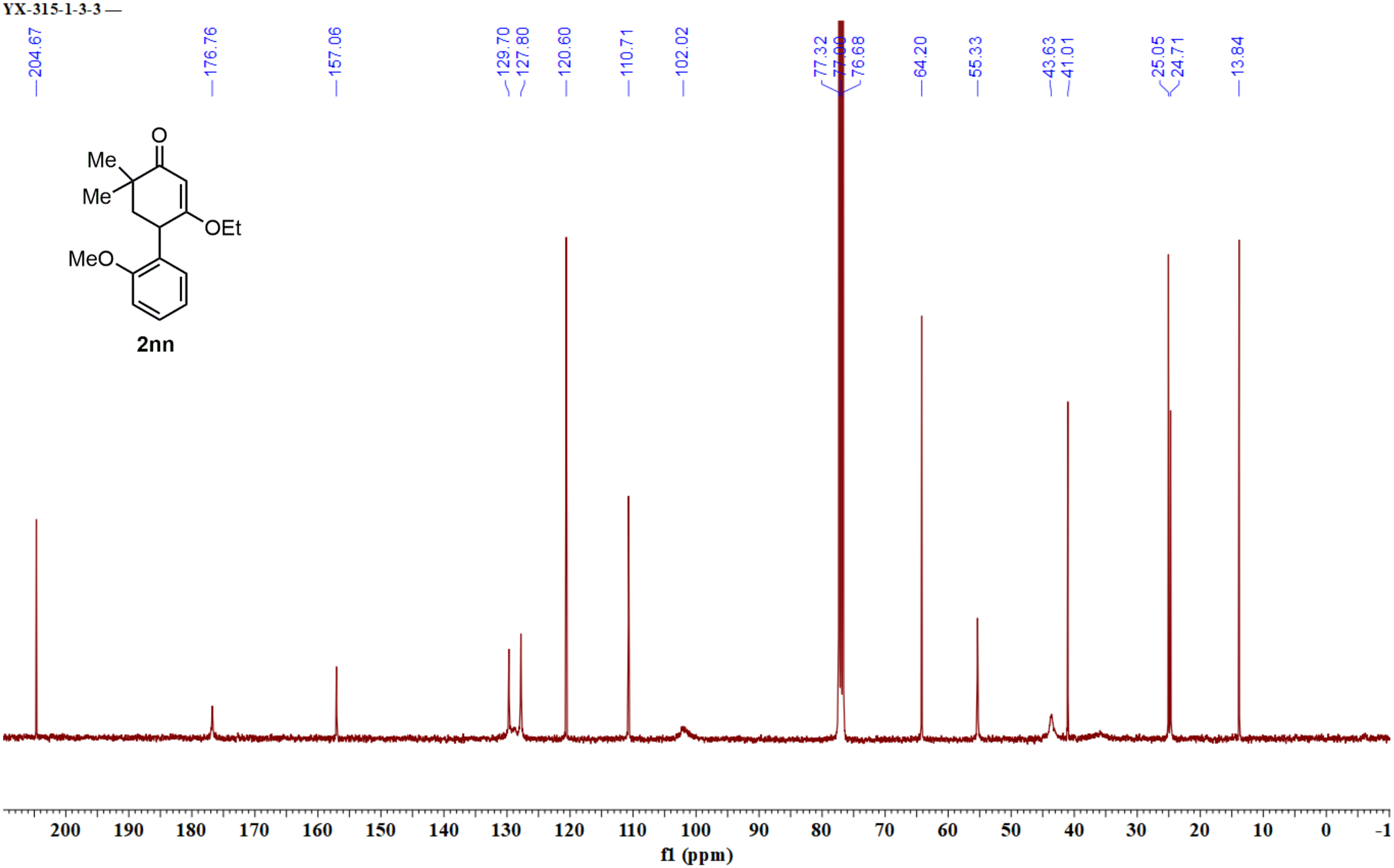

^{13}C NMR spectrum of compound 2nn

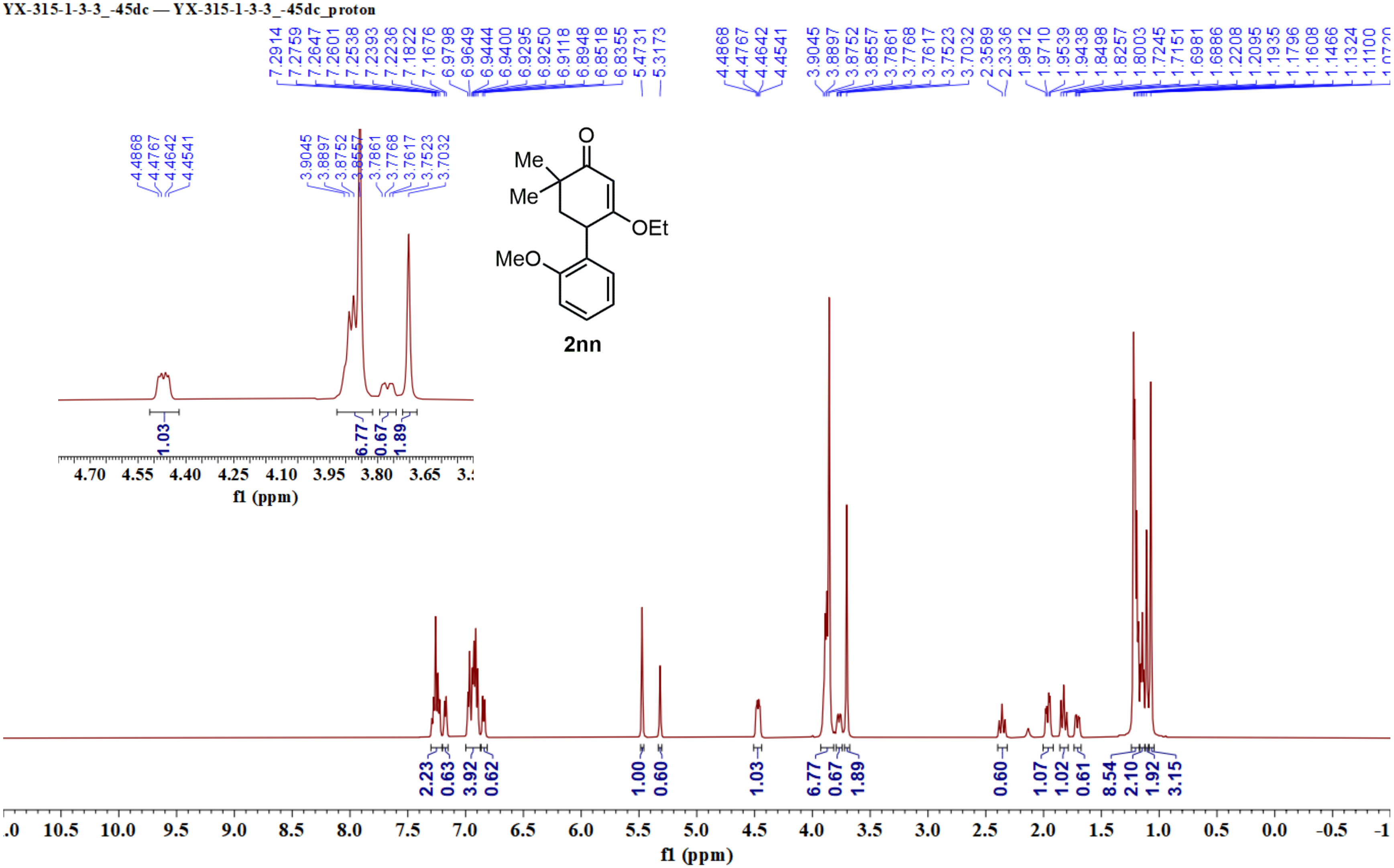

Temperature at −45 °C: ^{1}H NMR (500 MHz, $CDCl_3$) spectrum of compound 2nn

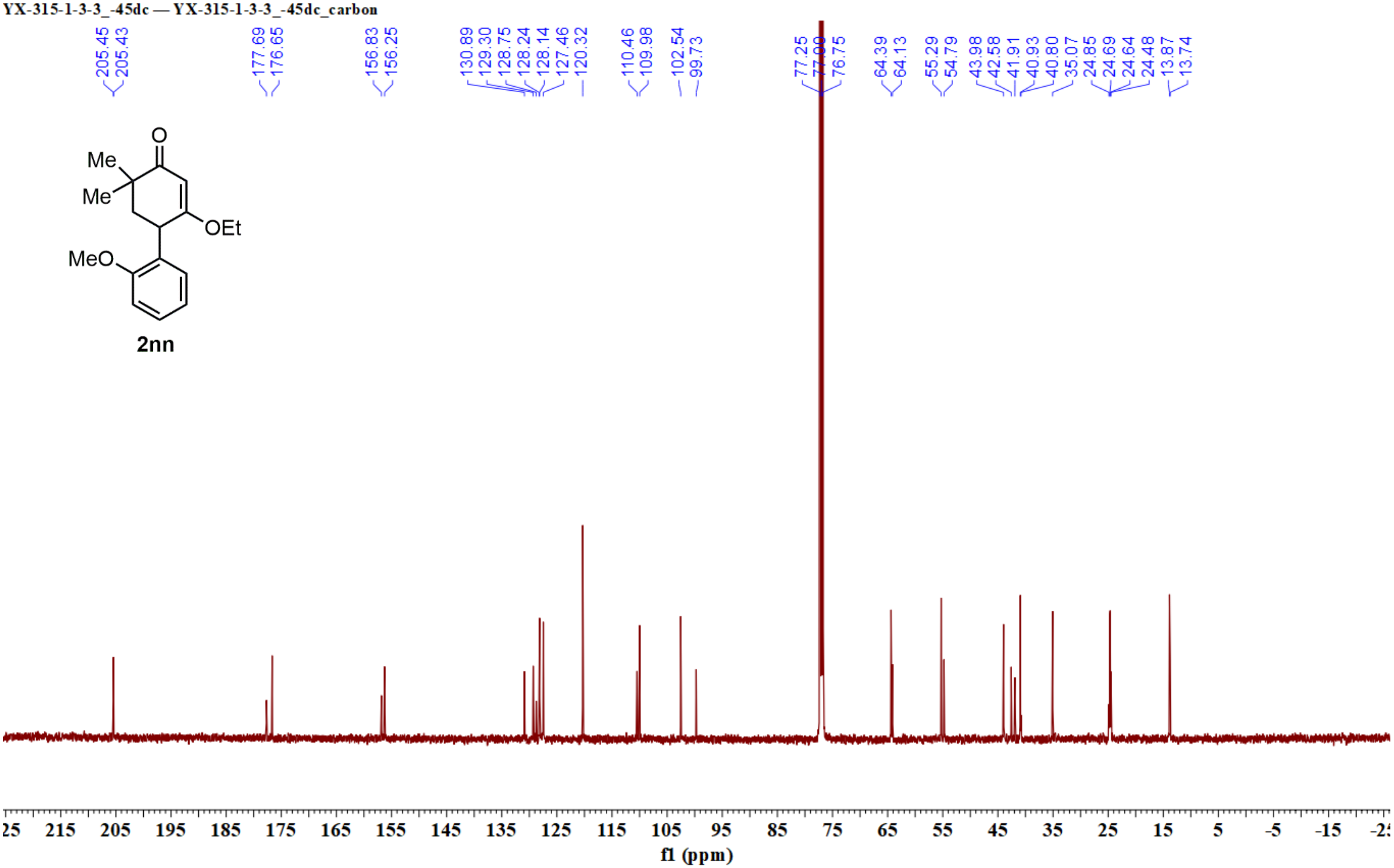

Temperature at −45 °C: ^{13}C NMR (125 MHz, $CDCl_3$) spectrum of compound 2nn

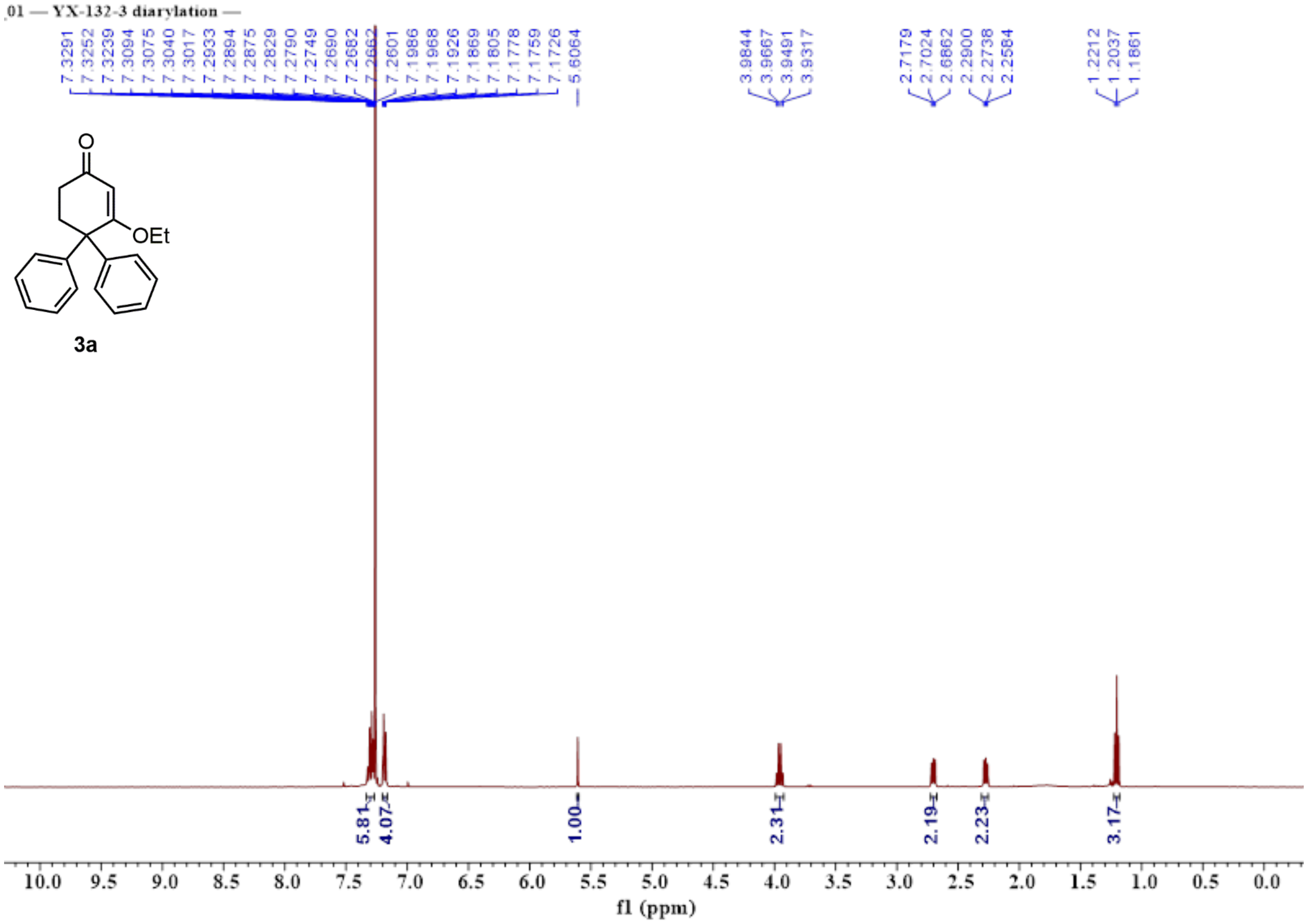

^{1}H NMR Spectrum of Compound 3a

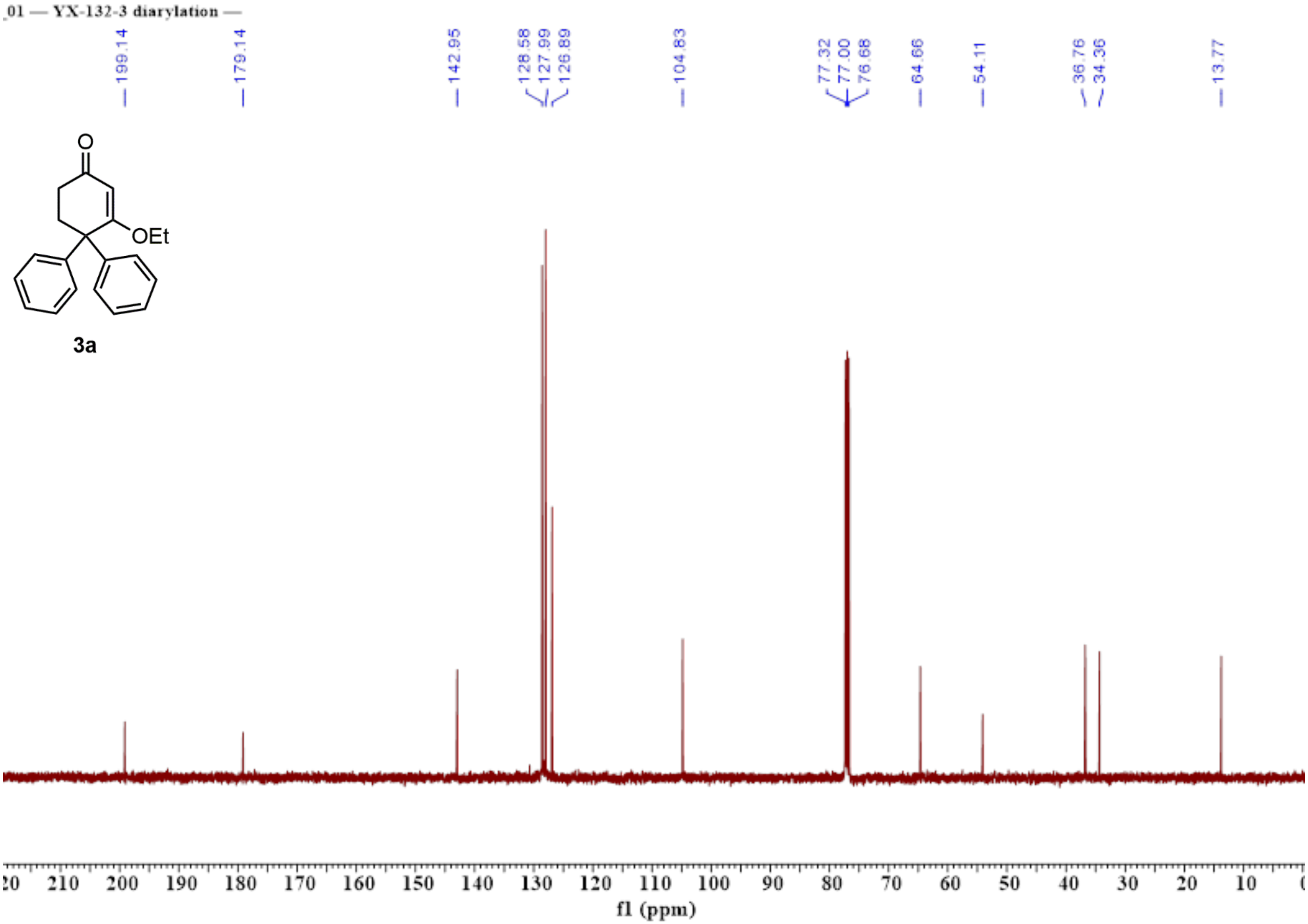

^{13}C NMR spectrum of compound 3a

gHMBCAD_01 — YX-132-3 diarylation —

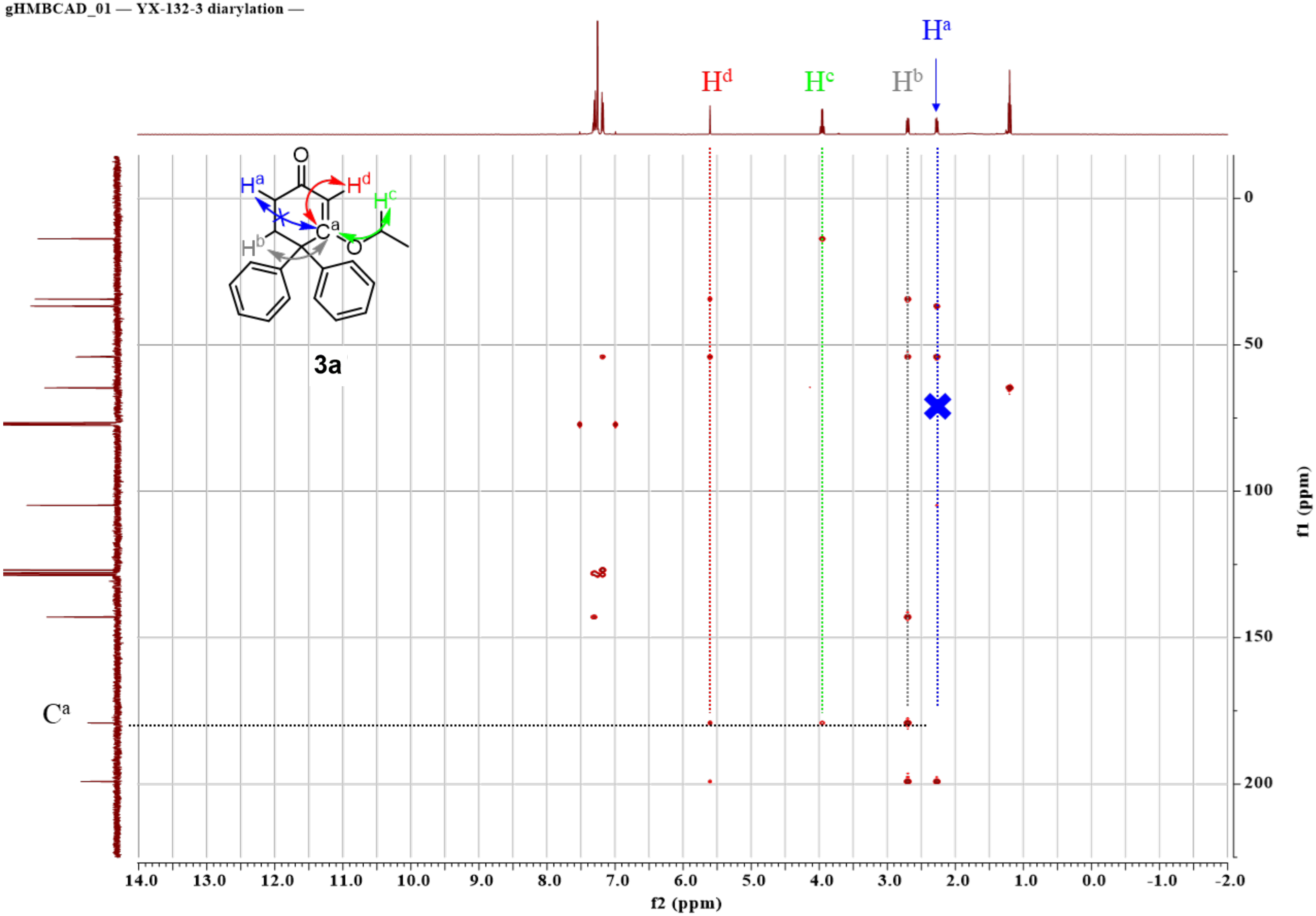

HMBC spectrum of compound 3a

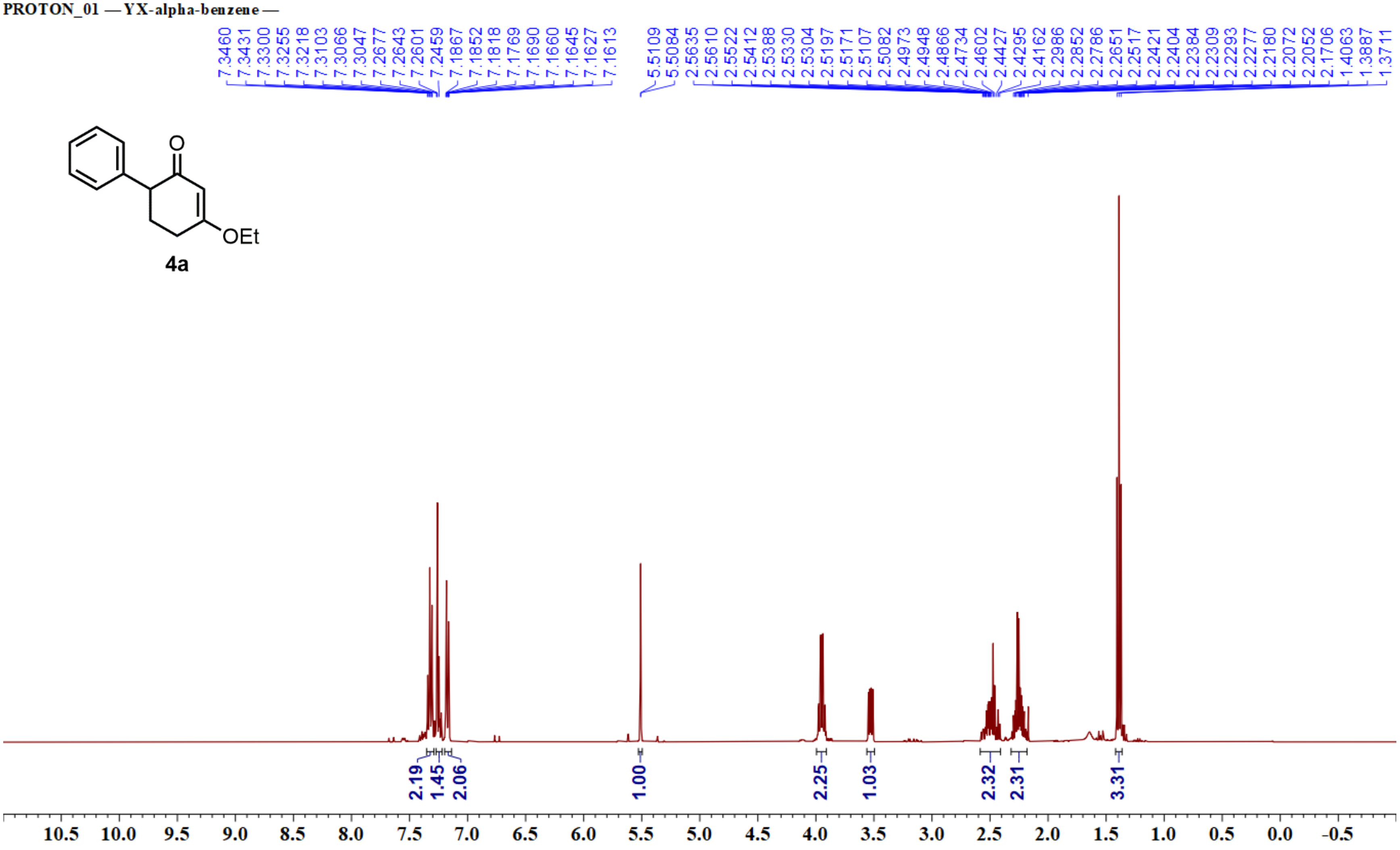

^{1}H NMR Spectrum of Compound 4a

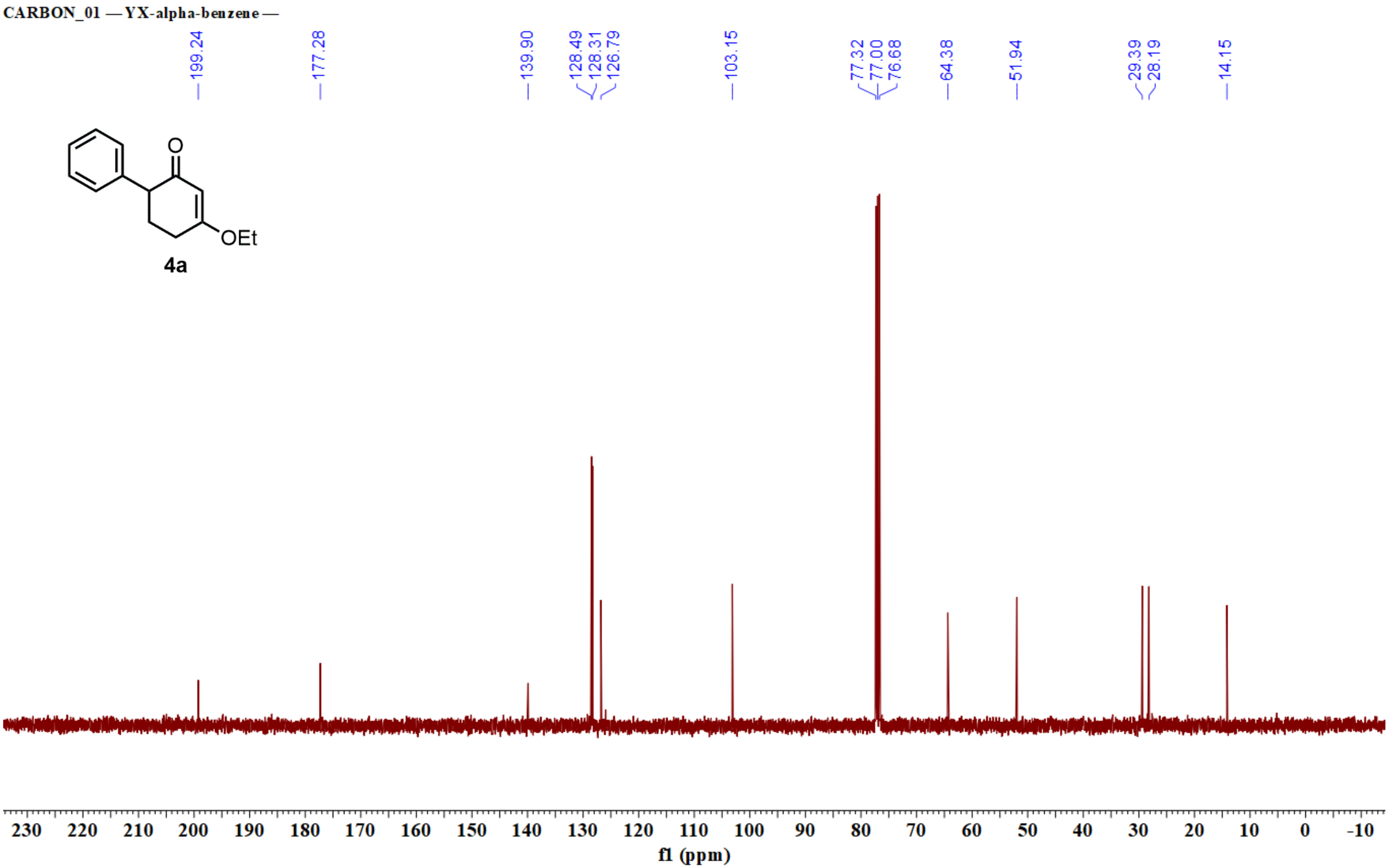

^{13}C NMR spectrum of compound 4a

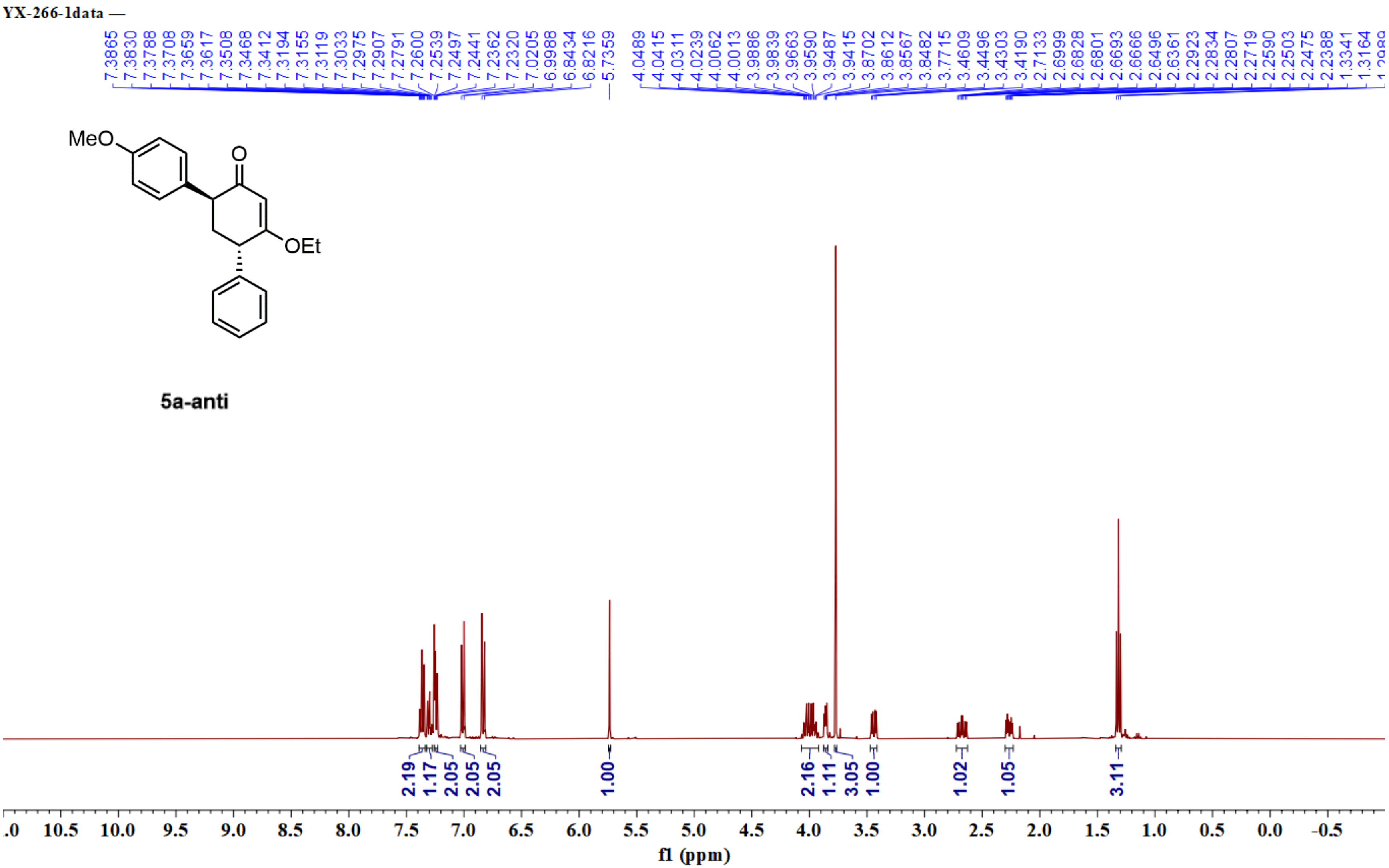

^1H NMR spectrum of compound 5a-anti

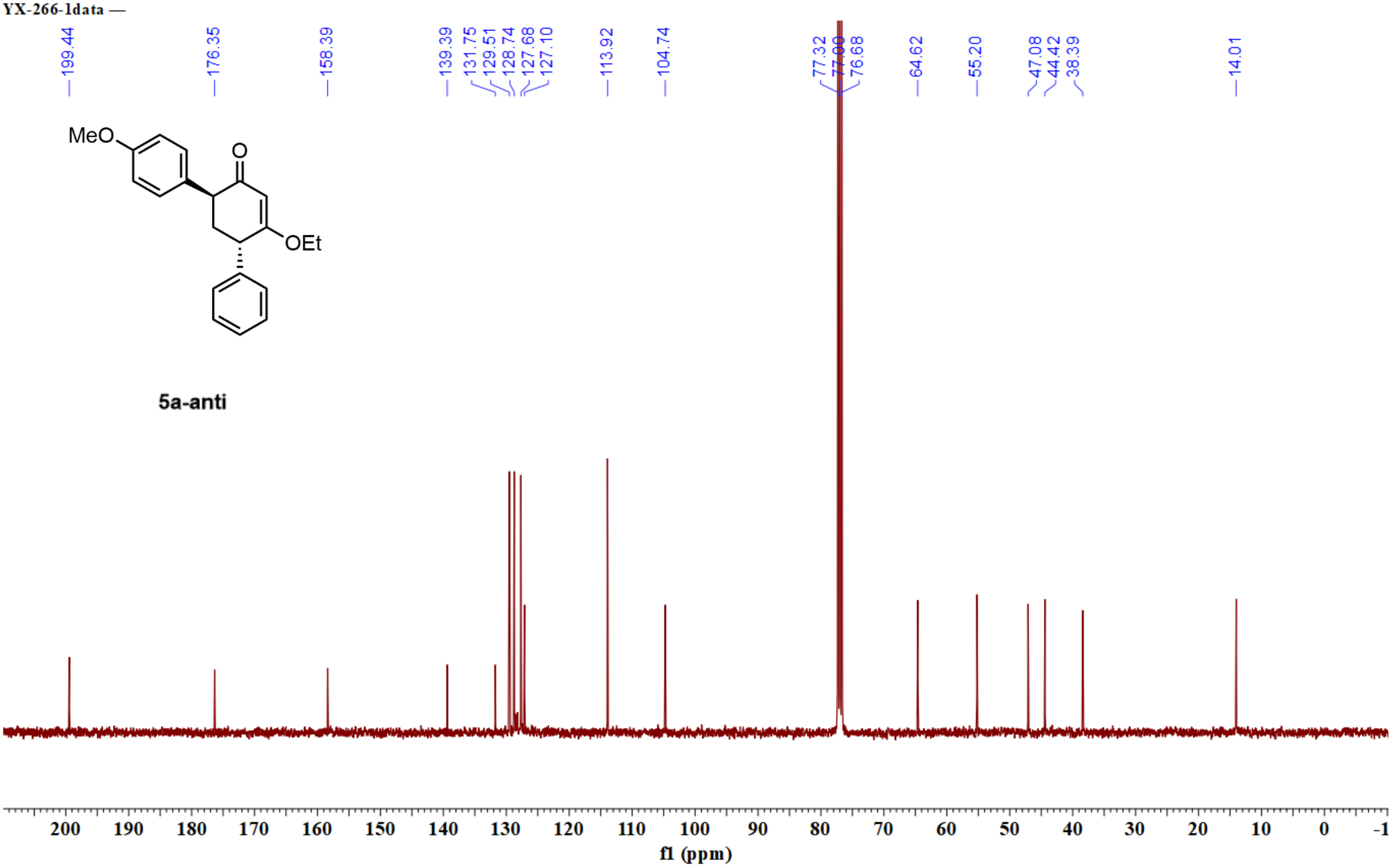

^{13}C NMR spectrum of compound 5a-anti

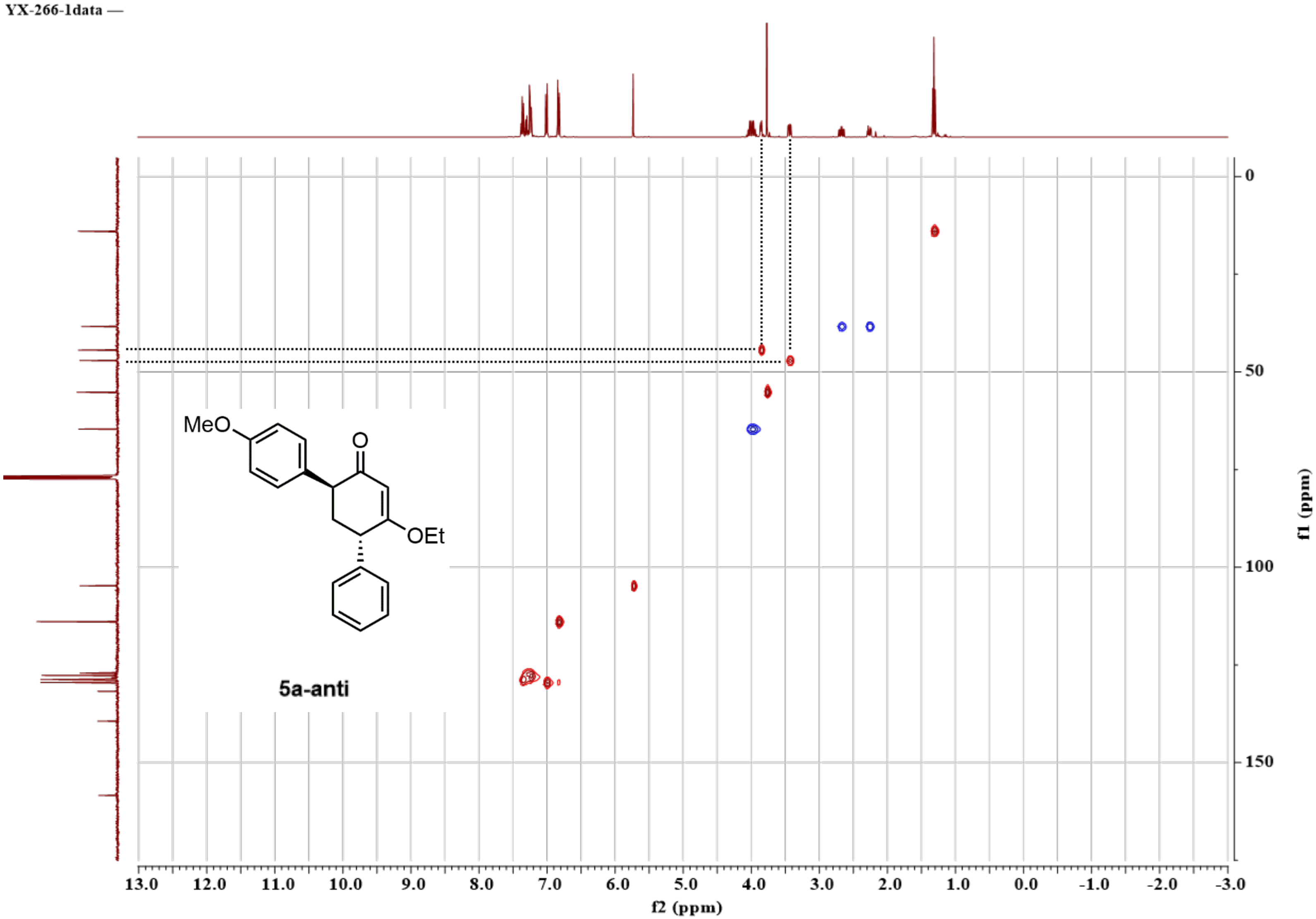

HSQC spectrum of compound 5a-anti

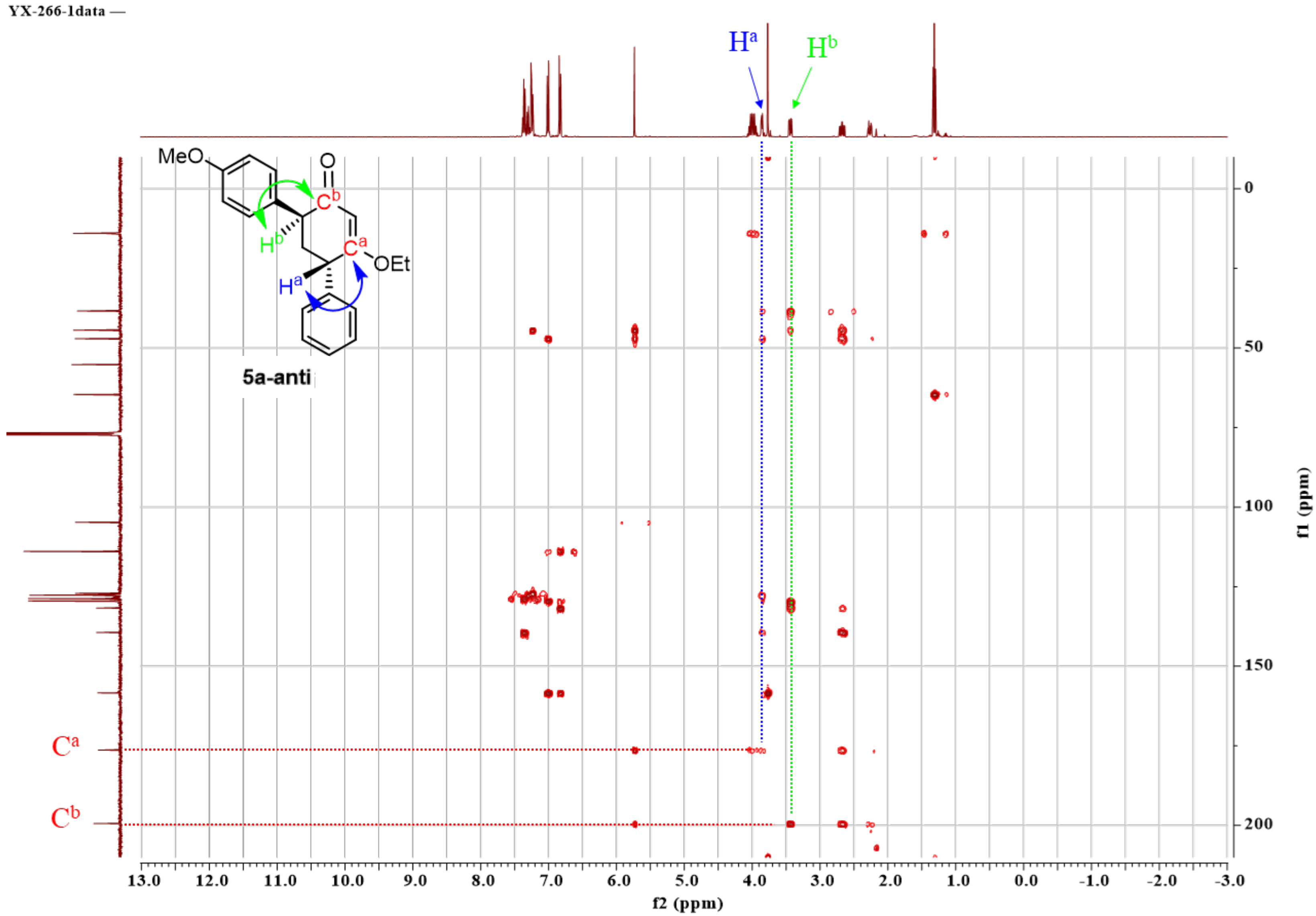

HMBC spectrum of compound 5a-anti

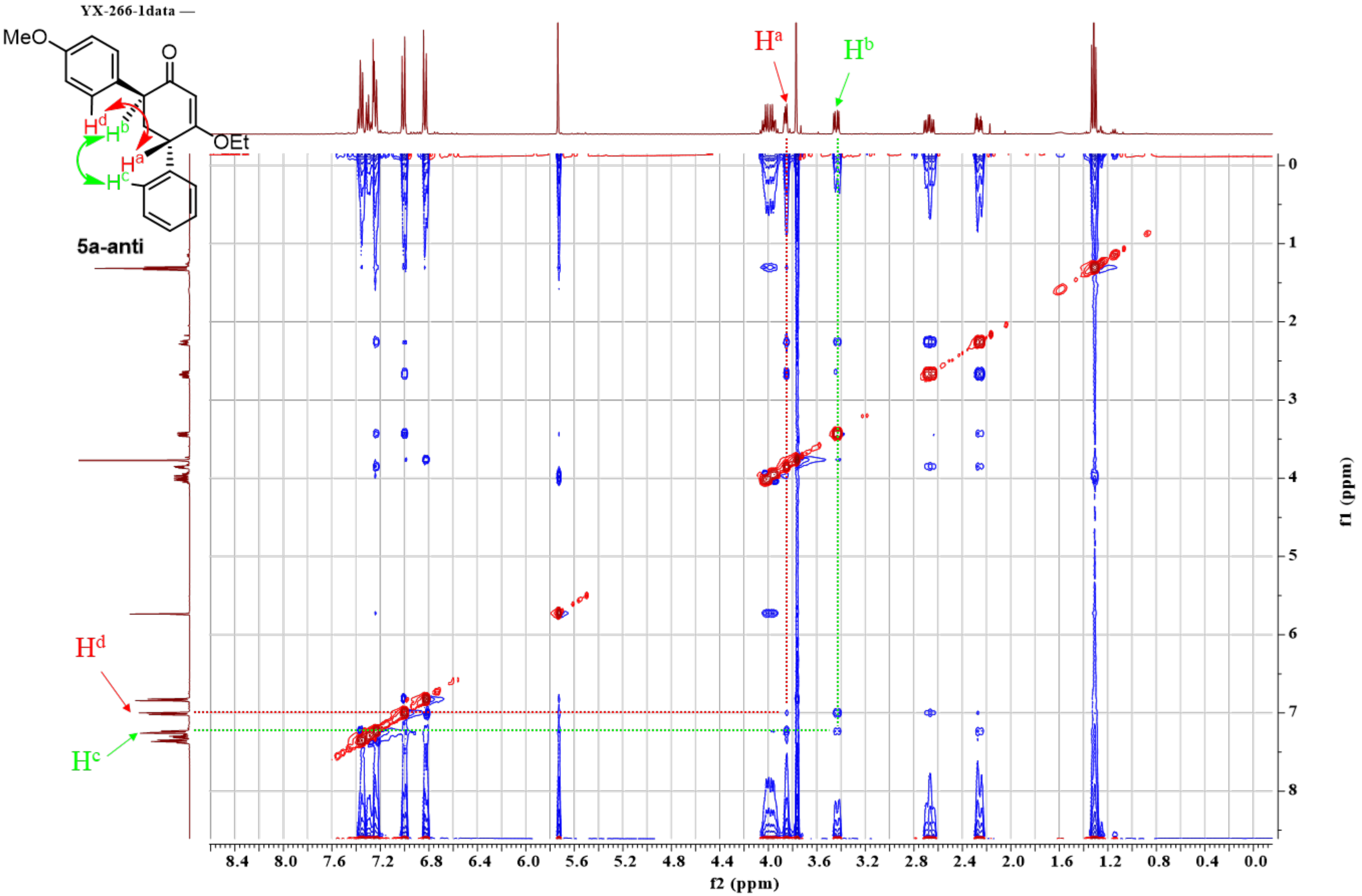

NOESY spectrum of compound 5a-anti

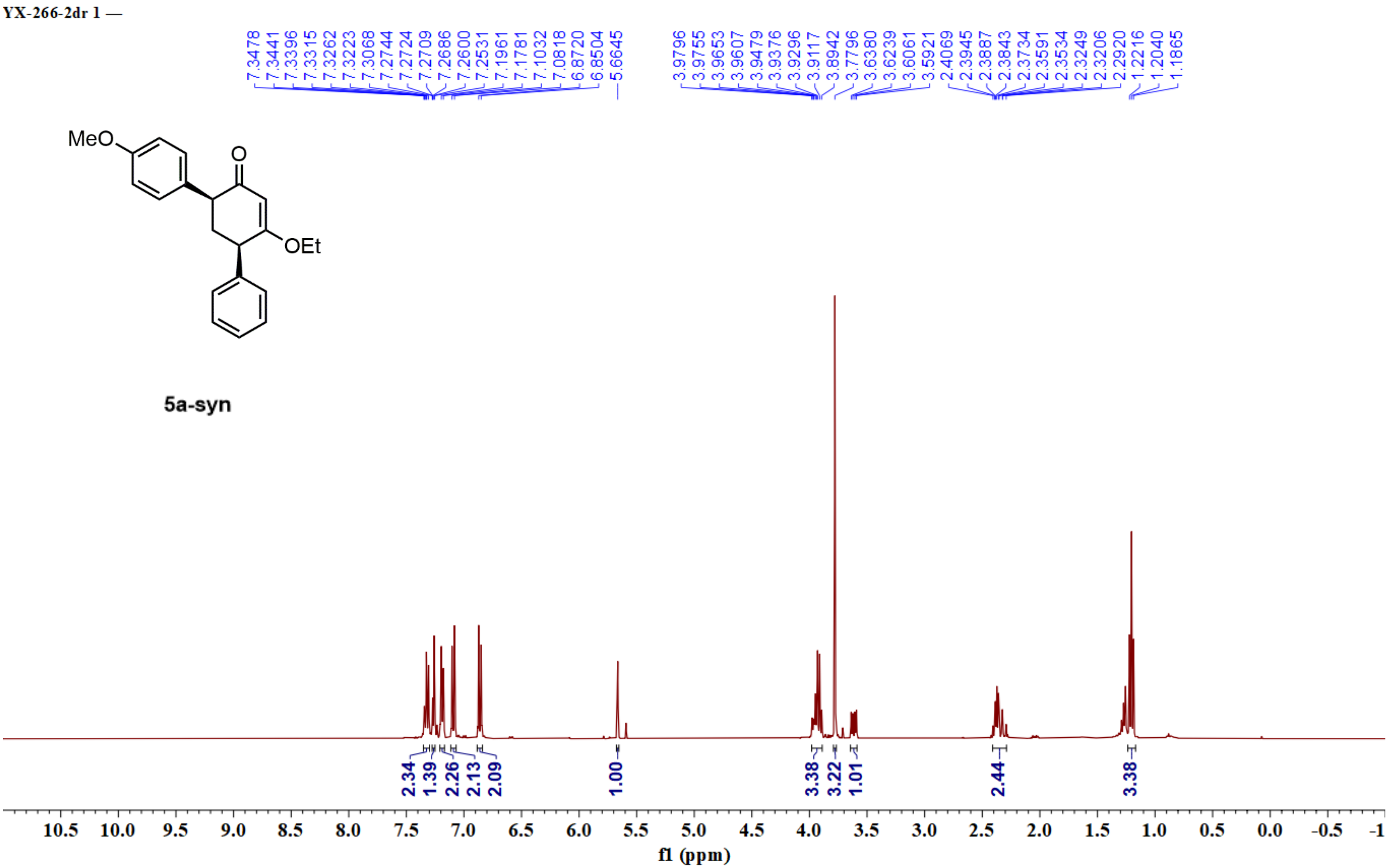

^{1}H NMR spectrum of compound 5a-syn

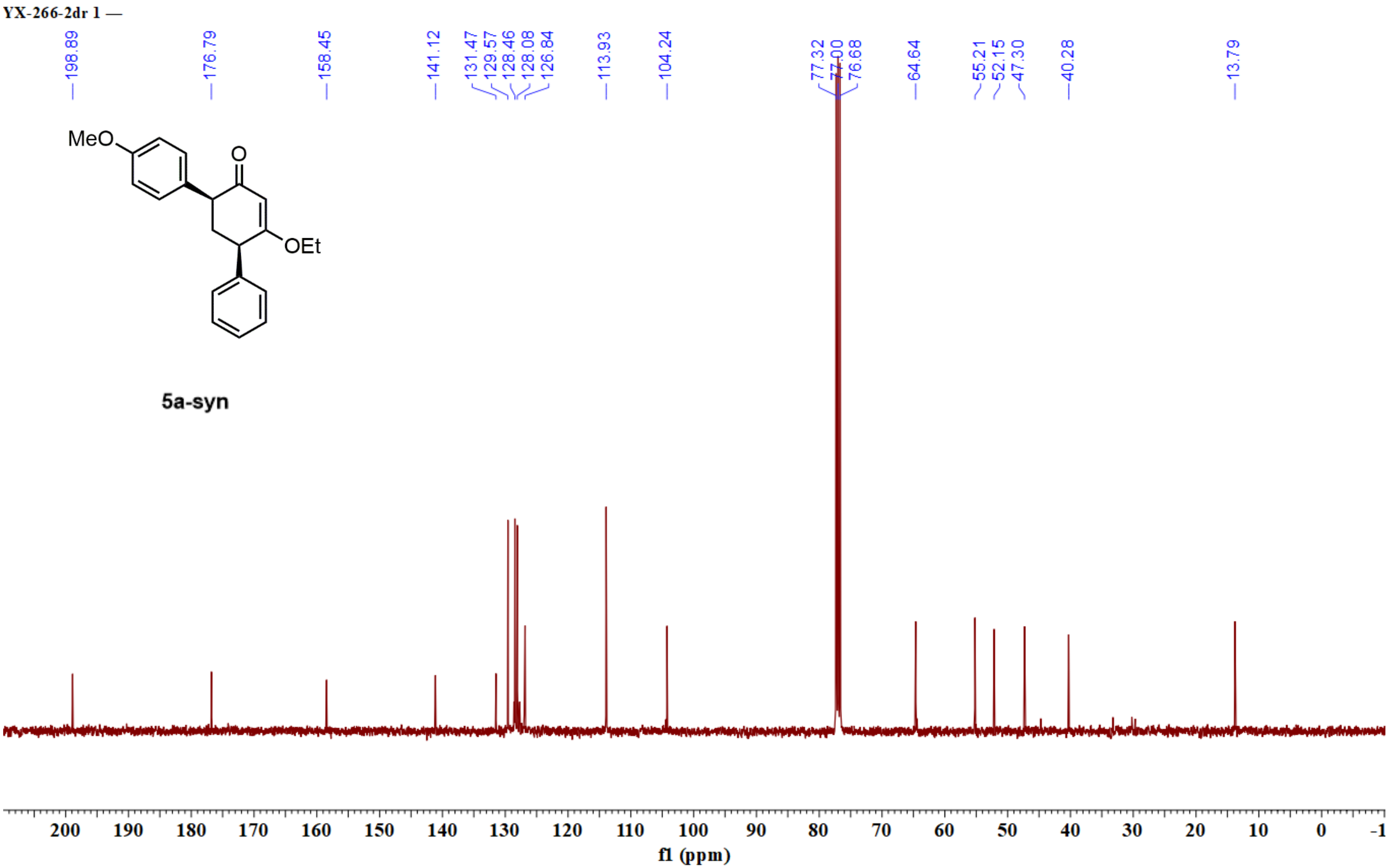

^{13}C NMR spectrum of compound 5a-syn

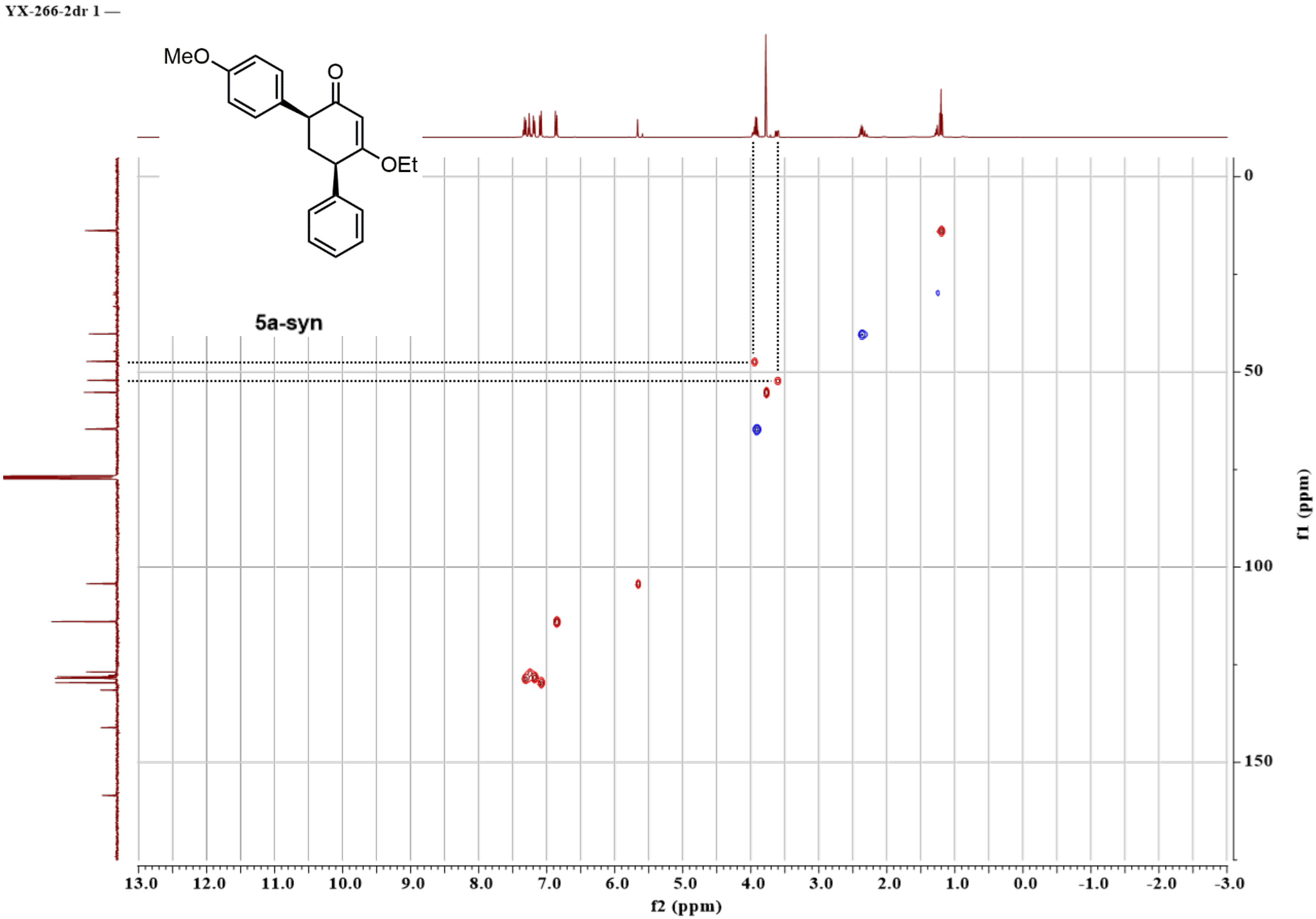

HSQC spectrum of compound 5a-syn

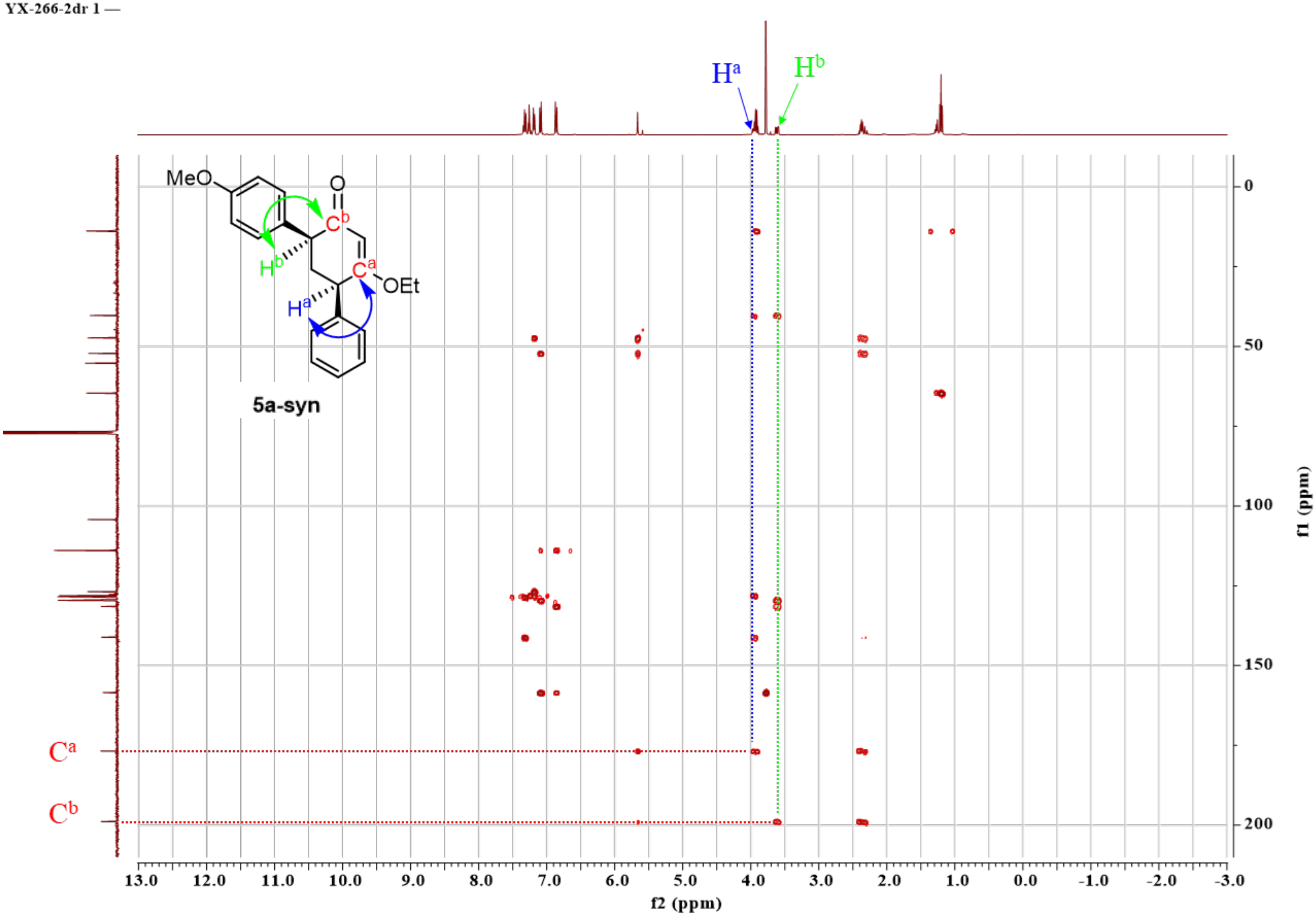

HMBC spectrum of compound 5a-syn

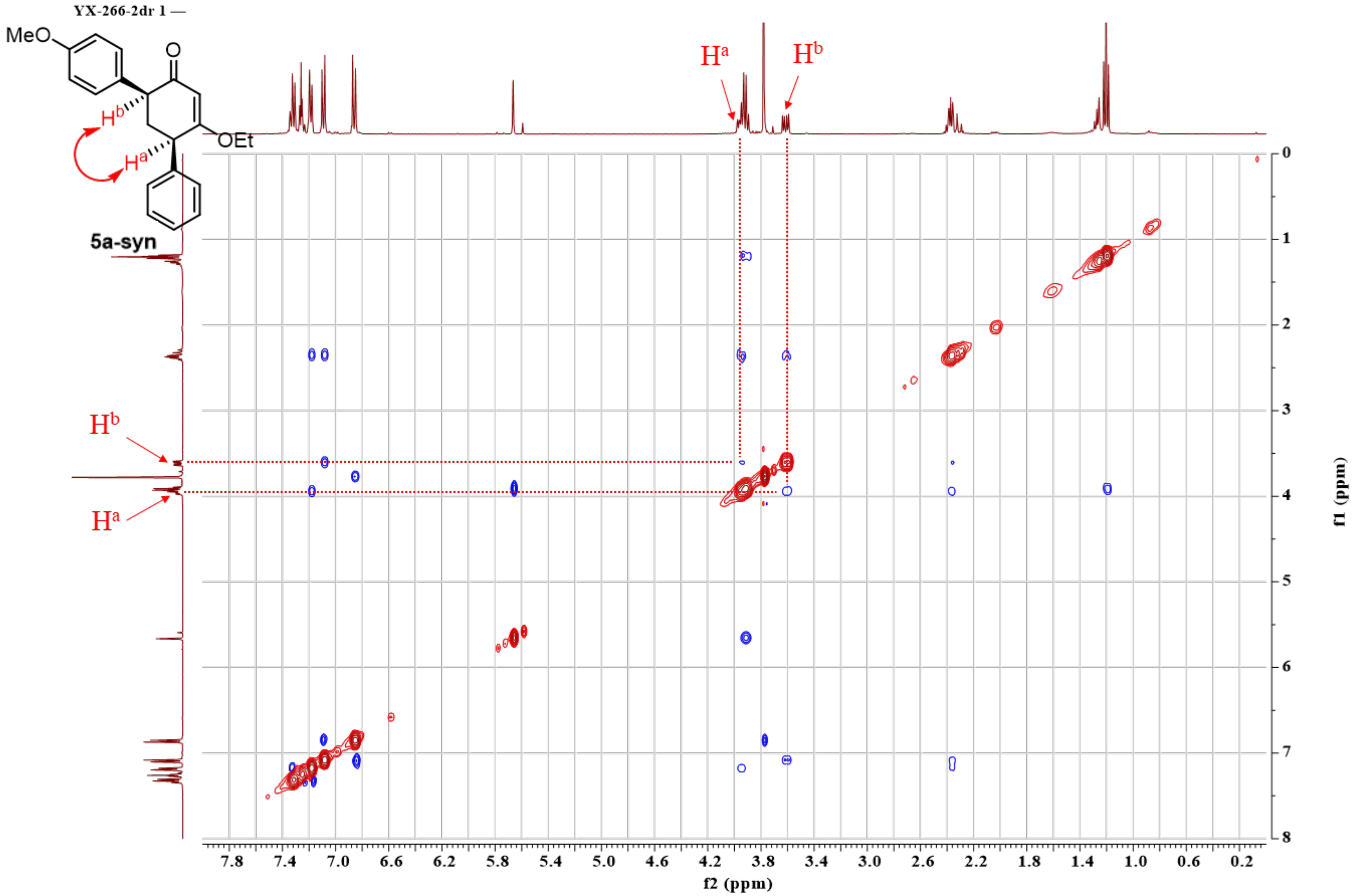

NOESY spectrum of compound 5a-syn

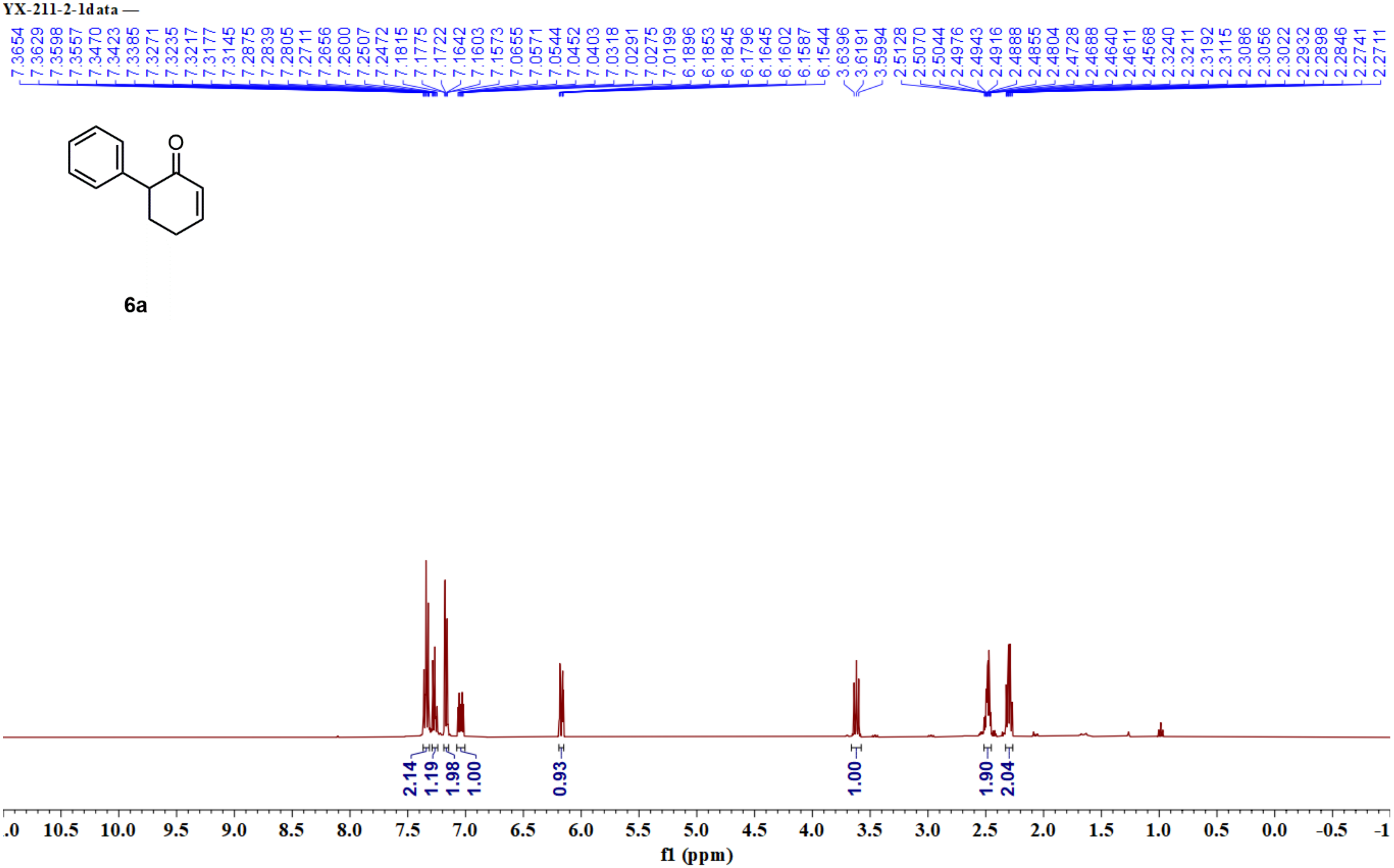

^{1}H NMR spectrum of compound 6a

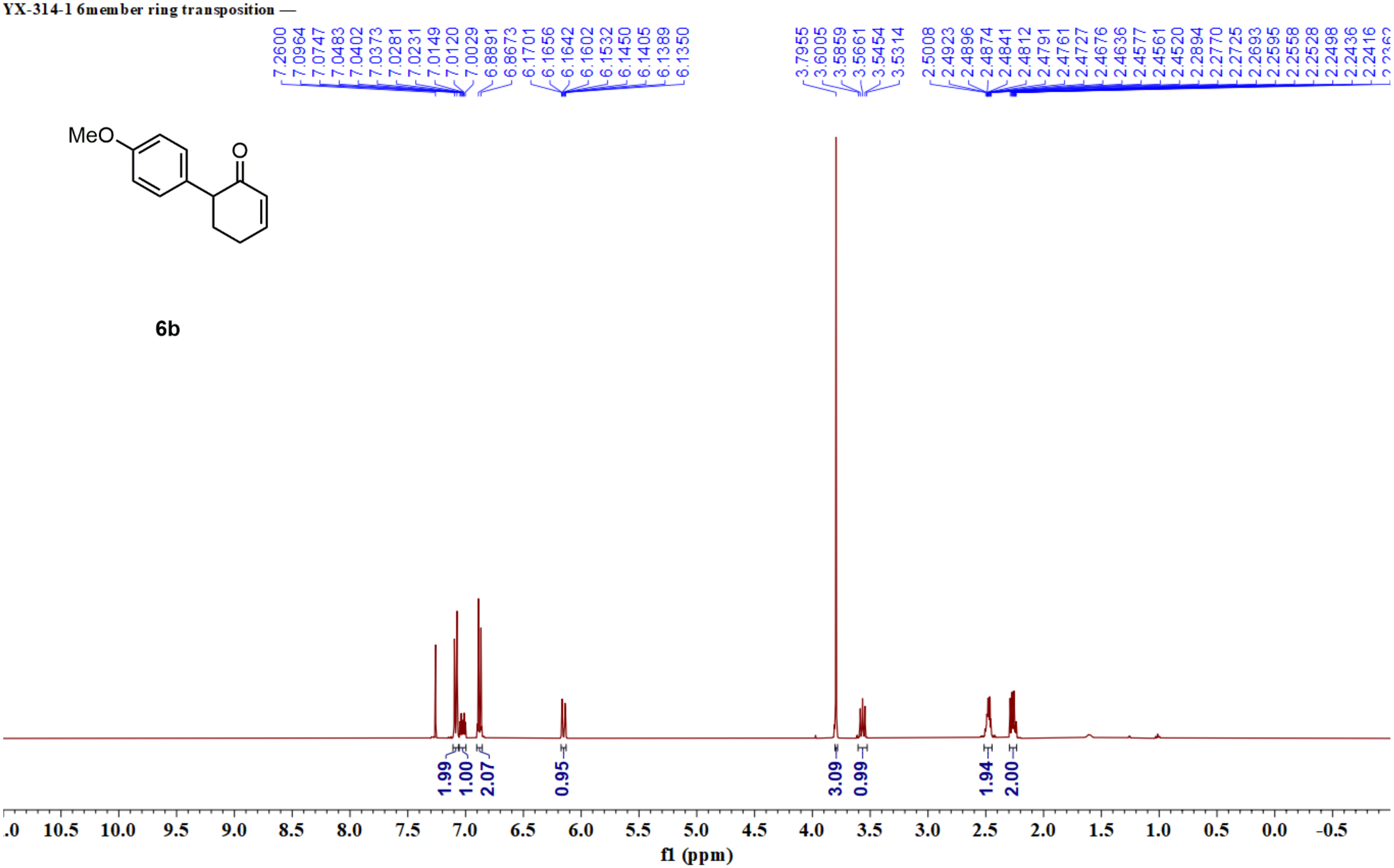

^{1}H NMR spectrum of compound 6b

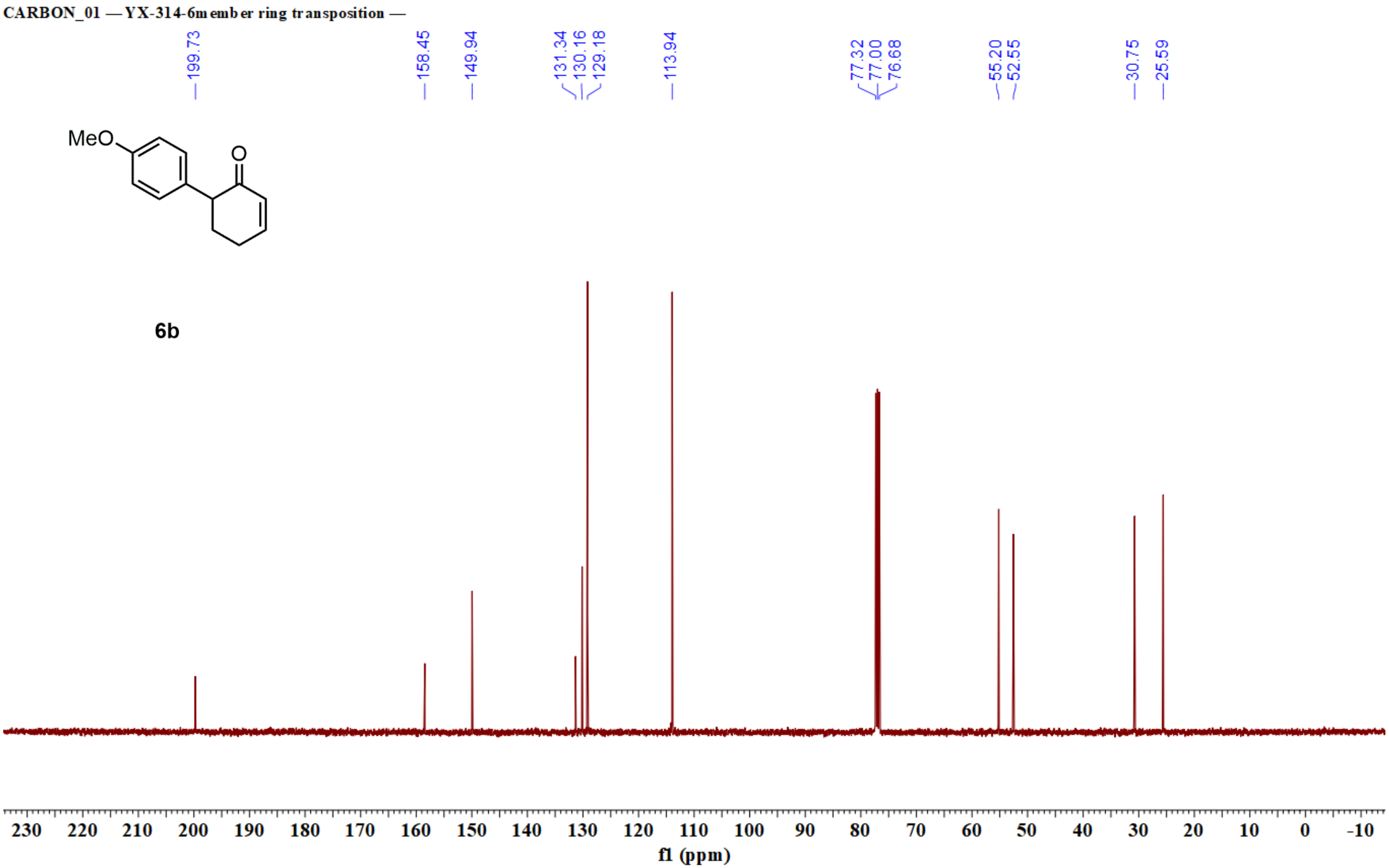

^{13}C NMR spectrum of compound 6b

PROTON_01 —YX-313-7member_ring_transposition—

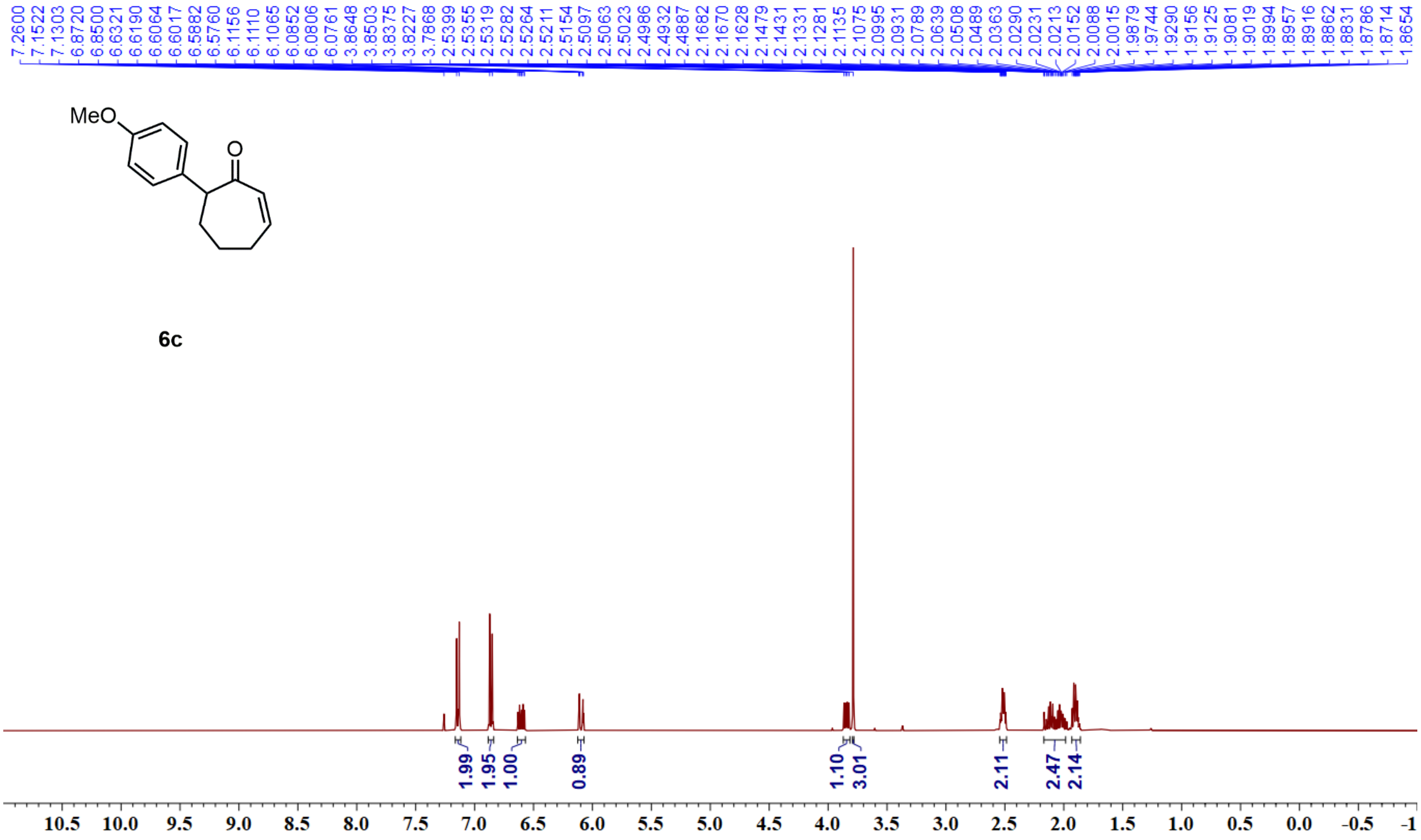

^{1}H NMR spectrum of compound 6c

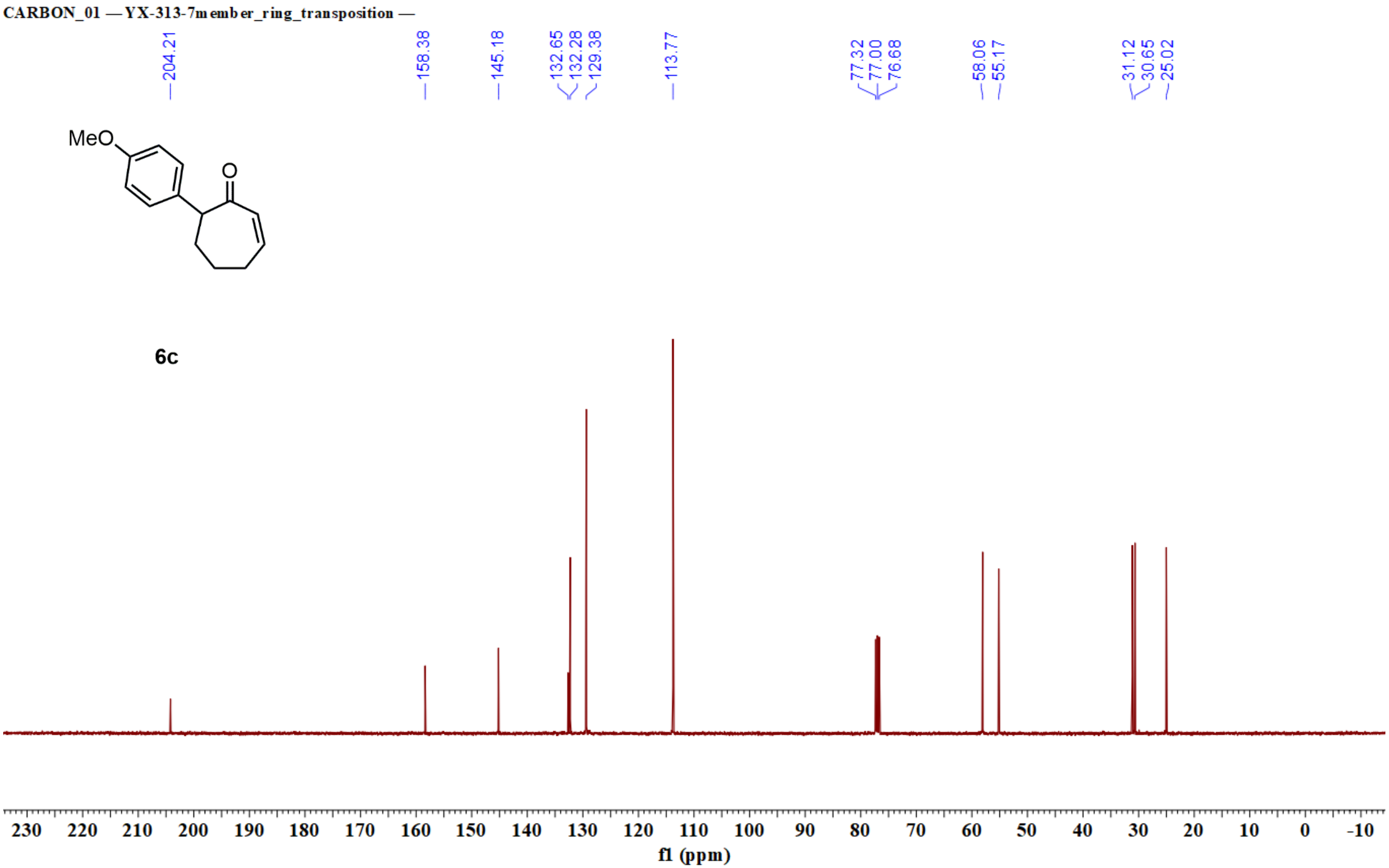

^{13}C NMR spectrum of compound 6c

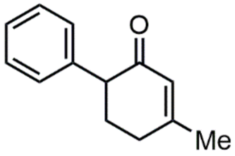

6d

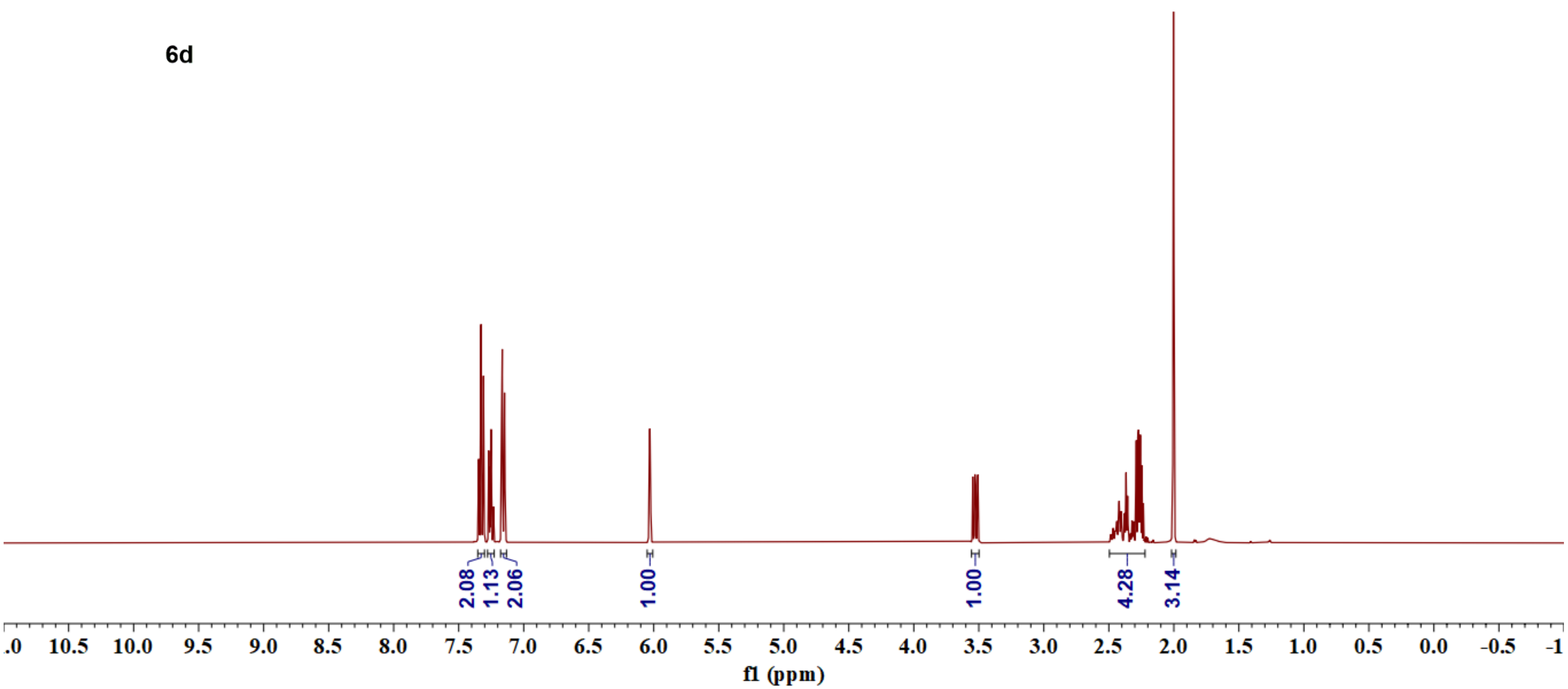

^{1}H NMR spectrum of compound 6d

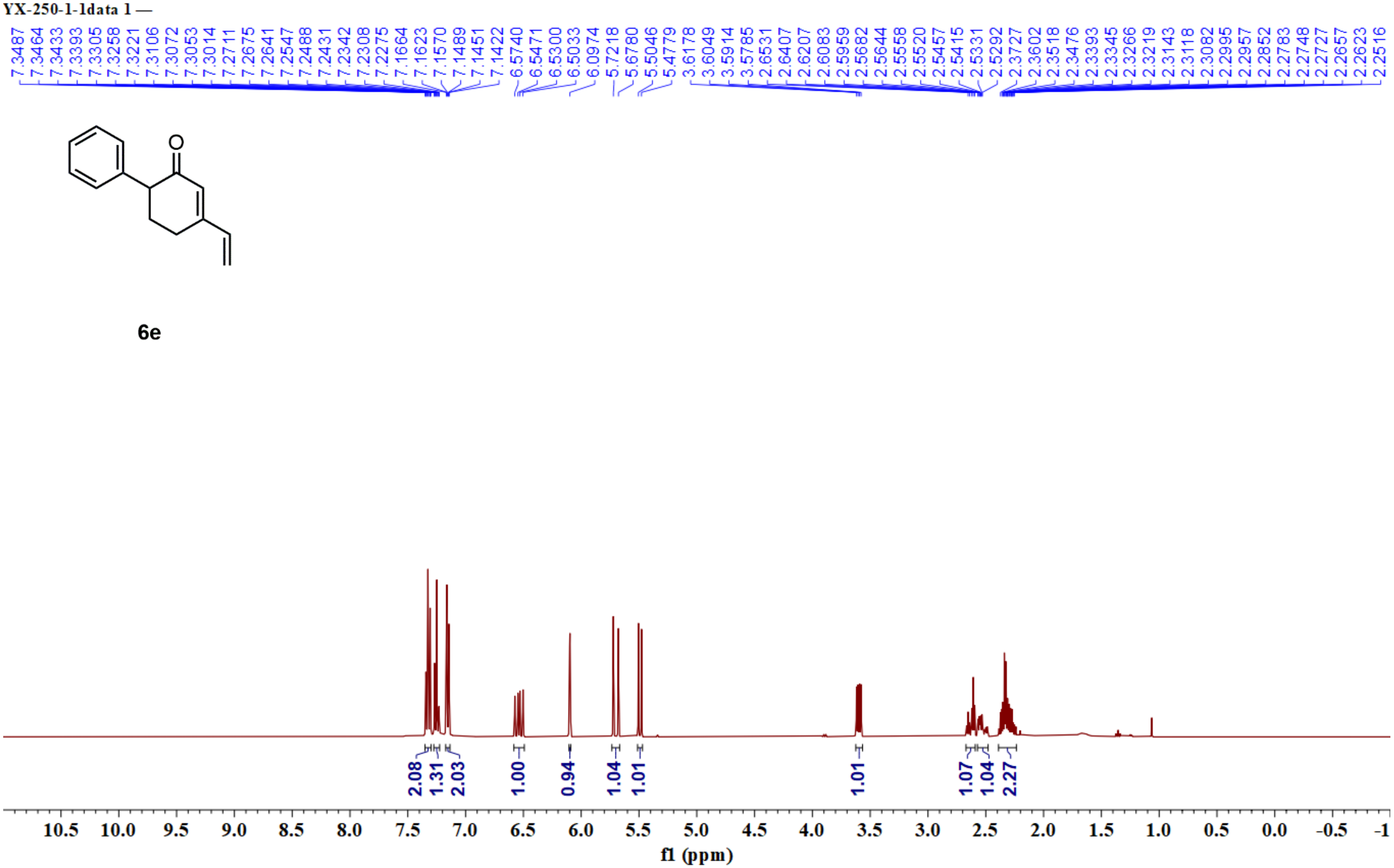

^{1}H NMR spectrum of compound 6e

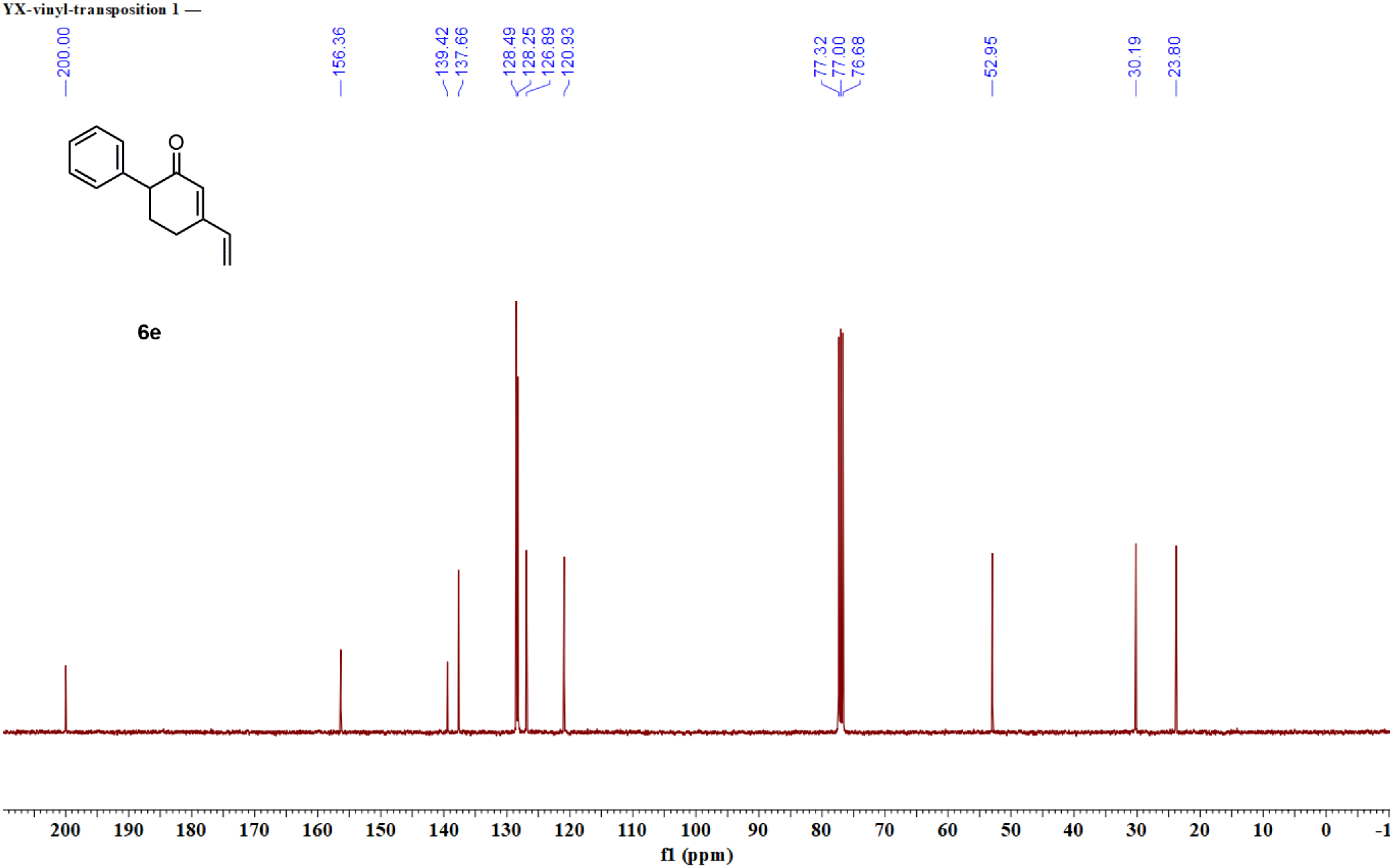

^{13}C NMR spectrum of compound 6e

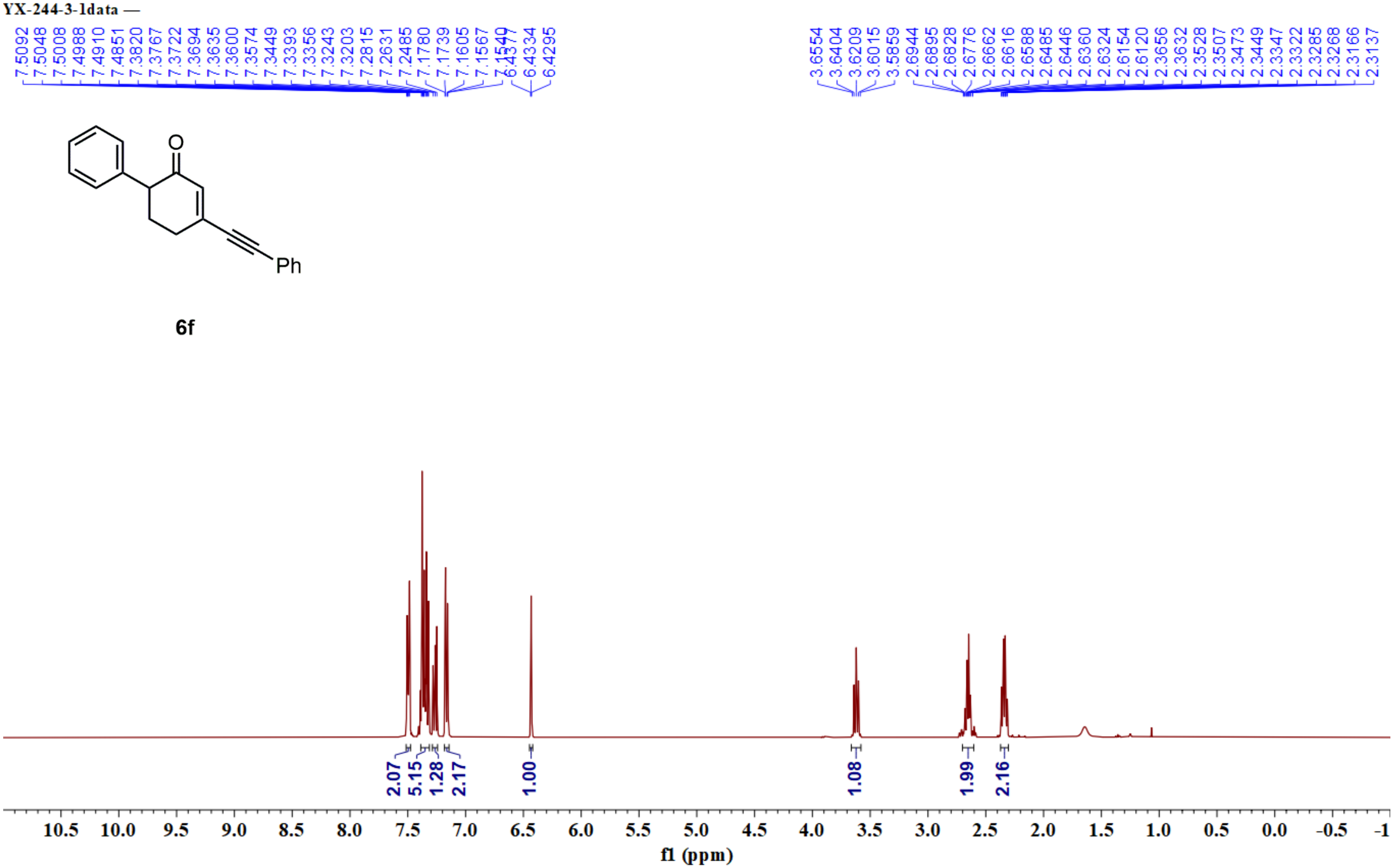

^{1}H NMR spectrum of compound 6f

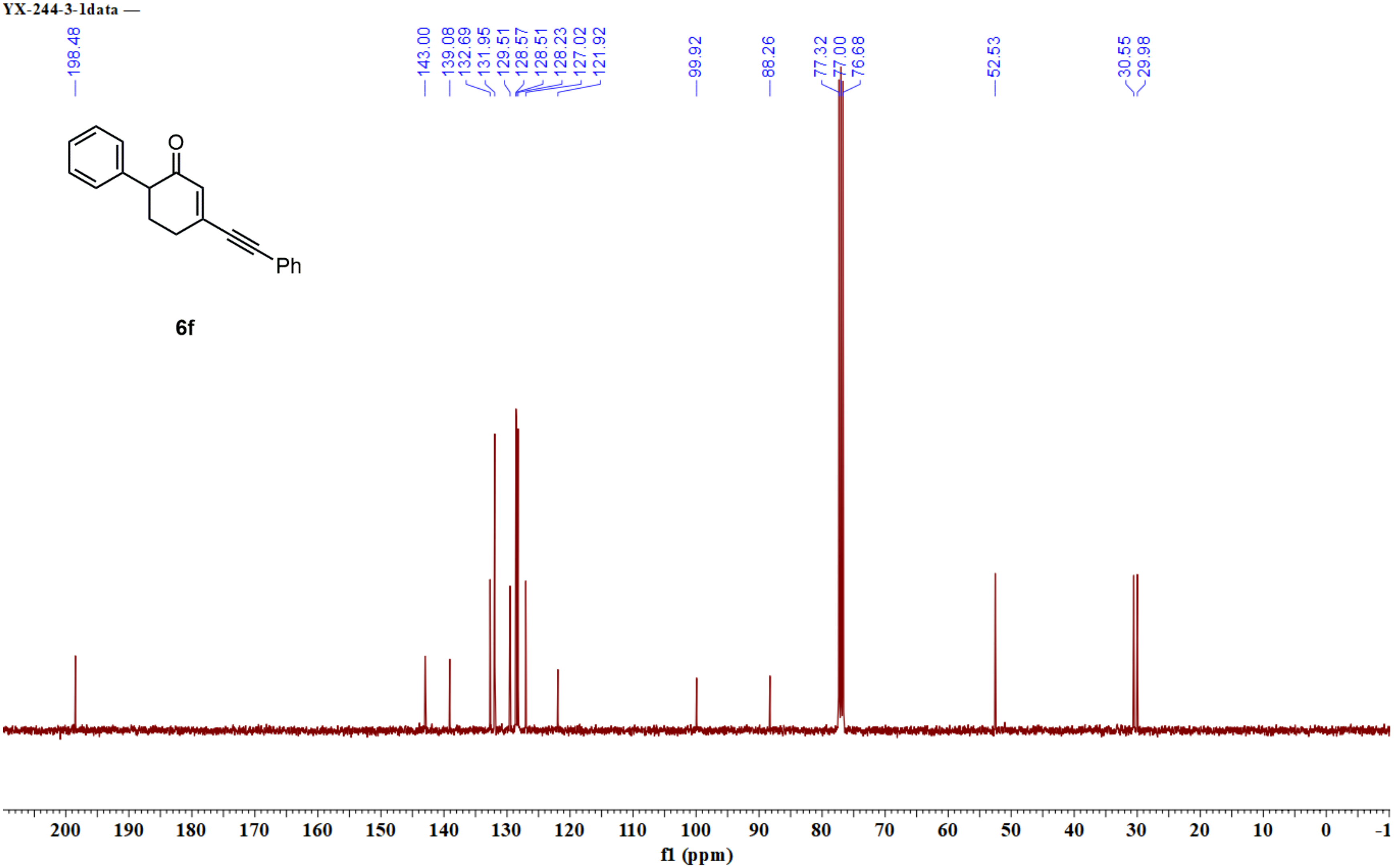

^{13}C NMR spectrum of compound 6f
